# Comparative genomics using teleost fish helps to systematically identify target gene bodies of functionally defined human enhancers

**DOI:** 10.1186/1471-2164-14-122

**Published:** 2013-02-23

**Authors:** Nazia Parveen, Ayesha Masood, Nouman Iftikhar, Bushra F Minhas, Rashid Minhas, Uzma Nawaz, Amir Ali Abbasi

**Affiliations:** 1 National Center for Bioinformatics, Program of Comparative and Evolutionary Genomics, Faculty of Biological Sciences, Quaid-i-Azam University, Islamabad 45320, Pakistan; 2 Department of Statistics, Govt: Post Graduate College For Women, Kutchary Road, Multan 60000, Pakistan

**Keywords:** CNEs, Human genome, Enhancers, Teleost fish, Synteny, Central nervous system, Transcriptional factors

## Abstract

**Background:**

Human genome is enriched with thousands of conserved non-coding elements (CNEs). Recently, a medium throughput strategy was employed to analyze the ability of human CNEs to drive tissue specific expression during mouse embryogenesis. These data led to the establishment of publicly available genome wide catalog of functionally defined human enhancers. Scattering of enhancers over larger regions in vertebrate genomes seriously impede attempts to pinpoint their precise target genes. Such associations are prerequisite to explore the significance of this *in vivo* characterized catalog of human enhancers in development, disease and evolution.

**Results:**

This study is an attempt to systematically identify the target gene-bodies for functionally defined human CNE-enhancers. For the purpose we adopted the orthology/paralogy mapping approach and compared the CNE induced reporter expression with reported endogenous expression pattern of neighboring genes. This procedure pinpointed specific target gene-bodies for the total of 192 human CNE-enhancers. This enables us to gauge the maximum genomic search space for enhancer hunting: 4 Mb of genomic sequence around the gene of interest (2 Mb on either side). Furthermore, we used human-rodent comparison for a set of 159 orthologous enhancer pairs to infer that the central nervous system (CNS) specific gene expression is closely associated with the cooperative interaction among at least eight distinct transcription factors: SOX5, HFH, SOX17, HNF3β, c-FOS, Tal1beta-E47S, MEF and FREAC.

**Conclusions:**

In conclusion, the systematic wiring of *cis*-acting sites and their target gene bodies is an important step to unravel the role of *in vivo* characterized catalog of human enhancers in development, physiology and medicine.

## Background

One of the main emerging challenges for genomics research is to discover all functional regions in the human genome. The completion of human genome sequencing/assembly and its annotation using computational and comparative genomic approaches has led to the cataloging of ~25,000 protein-coding genes. Key questions now relate to understanding how the spatial and temporal expression patterns of these human genes are established at cellular and organismal level [1]. In eukaryotes, transcriptional regulation tends to involve combinatorial interactions between several transcription factors, which allow for a sophisticated response to multiple conditions in the cellular environment [2,3]. In metazoans the precise spatial and temporal patterns of gene’s expression also require enhancer elements, distant regions of DNA that can loop back to the promoter [4]. To comprehend the molecular mechanism that governs specific expression patterns, it is important to identify the distant acting transcriptional regulatory elements (enhancers) associated with each predicted gene [5]. Furthermore, the ability to identify such elements is an essential step toward understanding how gene expression is altered in pathological conditions [1]. However, this task remains difficult due to lack of knowledge of the vocabulary controlling gene regulation and the vast genomic search space, with many of such distantly acting enhancers are positioned remotely from their target gene bodies [6].

Metazoan genes hold extremely intricate regulatory sequences that direct complex patterns of expression in diverse cell types, tissues and development phases [7]. Expression of a typical animal gene is likely to be governed by several distinct enhancer elements that can be located in 5^′^ and 3^′^ genomic regions, as well as within intronic intervals [8]. Metazoan *cis*-regulatory sequences are modular with each enhancer is responsible for a subset of the total gene expression pattern and usually mediate expression within a specific tissue/cell type or developmental phase/domain [9]. These elements are typically up to 500 bp long and contains binding sites for sequence-specific several distinct transcription factors [8].

The origin of organismal complexity is often thought to be the consequence of evolution of novel gene functions subsequent to gene duplication events [10]. According to the classical model (describing evolutionary fate of duplicate genes) one copy of the duplicated gene pair often degenerates by accumulating deleterious mutations, whereas the other copy keeps the ancestral function. This model further predicts that very rarely, one gene copy may obtain a novel adaptive function, resulting in the preservation of both duplicates, one copy with the new function and the other preserving the ancestral function. However, in numerous cases, empirical data suggest that the fraction of genes preserved subsequent to duplication events is much higher than predicted by the classic model. Keeping in view the regulatory complexity of eukaryotic genes, it was proposed that complementary degenerative mutations in distinct regulatory elements of duplicated genes can assist the preservation of both copies, thus providing long-term opportunities for the evolution of novel gene functions [11]. Numerous duplicate genes have been confirmed to evolve following this model of regulatory subfunctionalization. For instance, zebrafish engrailed-1 and engrailed-1b is a pair of transcription factor genes generated by a duplication event specifically in the lineage of teleost fish [11]. Expression pattern analysis revealed distinct expression domains for zebrafish engrailed paralogs with engrailed-1 is expressed in the pectoral appendage bud, whereas engrailed-1b is expressed in the hindbrain/spinal cord region [11]. The mouse genome harbor single ortholog (engrailed-1) for both genes of the zebrafish, which is expressed in both pectoral appendage bud and hindbrain/spinal cord. Complementary changes in gene expression domains after gene duplication events appear to be a general rule rather than exception and such changes usually happen rapidly after gene duplication [12].

Genomic comparison of diverse set of vertebrate species revealed many genomic intervals that have remained conserved throughout the vertebrate lineage [13]. Some of these sequences correspond to coding genes and non-coding RNAs, however two third of them are unlikely to produce a functional transcript [14]. These sequences fall in the new category of elements, which we collectively call as conserved non-coding elements (CNEs) [14]. These elements are experimentally characterized to harbor transcriptional regulatory elements, so involved in gene expression regulation [15]. Therefore, comparative genomics based strategies are now being employed to predict genomic regions harboring transcriptional regulatory elements even in the absence of knowledge about the specific characteristics of individual *cis*-regulatory element [16].

To explore the functional significance of conserved non-coding genomic elements, Pennacchio and coworkers (2006 & 2008) carried out *in vivo* enhancer analysis of hundreds of human CNEs in transgenic mice assay by using *LacZ* as a reporter gene [15,17,18]. This data confirmed the gene regulatory function for ~1000 of these sequences, directing reproducibly the reporter expression in diverse set of body tissues at mouse embryonic day 11.5 [17]. To elucidate the significance of this *in vivo* characterized catalog of human enhancers in organismal development, physiology and medicine it is essential to pinpoint the precise target gene for each of these elements. Noteworthy, Pennacchio and coworkers (2006 & 2008) associated the gene regulatory potential of these human enhancers with the genes harboring them (intragenic) or their immediate flanking genes (intergenic) [17]. However, the empirical evidence showed that enhancers regions are often located at large distances from transcriptional start site of the genes upon which they act [19]. They may be located upstream or downstream of target gene, within introns, in introns of unrelated neighboring genes or can be found at a distance of 1 Mb or even greater and are still able to regulate the gene expression in tissue specific manner [20]. Scattering of enhancer elements over larger regions in vertebrate genomes, impede attempts to assign precise target gene bodies to functionally characterized enhancer elements.

In the present study, through combined application of comparative synteny analyses and reported endogenous expression pattern investigation we systematically hunt for the precise target gene bodies of experimentally verified catalog of human gene enhancers [15]. We concentrate on deeply conserved human enhancers; by restricting to 192/975 *cis-*regulatory regions that show sequence conservation between mammals and teleost fish (last common ancestor existed 450 million years ago) (Table 1, for detailed list see Additional file 1: Table S1). By assuming that syntenic relationship between an enhancer and concerned target gene remains preserved subsequent to speciation (orthologous loci) and duplication events (paralogous loci) [20], we associated explicitly 85/192 enhancers to single target gene body (Table 1 and Additional file 1: Table S1). In those cases where more than one gene maintained conserve linkage (human-teleost fish orthologous loci) with single enhancer the pattern of reporter expression induced by an enhancer was compared manually (via image data) with the reported endogenous expression pattern of syntenically conserved genes to establish the enhancer-gene relationship. This strategy assisted further in assigning the activity of 57/192 enhancers to single gene, 43/192 enhancers to 2 target genes and 7/192 enhancers to 3 target genes (Table 1, for detailed list see Additional file 1: Table S1).

**Table 1:** The association of the human CNE-enhancers with their target gene bodies by comparative syntenic analyses and through comparison of the reporter expression induced by CNE-enhancers with the reported endogenous expression patterns of the neighboring genes

**No. of enhancers**	**No. of target genes**	** Technique used**		** Minimal evidence for association**	
		**Paralogy mapping (duplication)**	**Orthology mapping (synteny)**	**Only synteny**	**Synteny along with expression (MGI *in-situ*)**
39	1	√	√	√	¯
2	2	√	√	¯	√
1	1	√	√	¯	√
1	1	√	√	√	*
41	2	¯	√	¯	√
7	3	¯	√	¯	√
44	1	¯	√	√	¯
56	1	¯	√	¯	√
1	1	¯	√	√	*

This table provides the following information about human CNE-enhancers.

(i) The number of target genes associated with each CNE-enhancer analyzed.

(ii) Whether they are duplicated or not.

(iii) Their association with target gene is supported by either syntenic analysis or by endogenous expression analysis or by both.

* Expression pattern is not known.

MGI: Mouse Genome Informatics.

CNE: Conserved non-coding element.

Once the enhancers were assigned to their probable targets the next, we sought to use these associations as a training dataset to gauge the maximum distance at which an enhancer can act upon its target gene body. Furthermore this study aims to define the central nervous system (brain and spinal cord) specific transcription factor code.

## Results and discussion

### Predicting target genes for human CNE-enhancers

We devised a rule-based procedure to associate CNE-enhancers with their respective target gene (see methods). The human CNE-enhancers opted for this purpose is conserved over longer evolutionary distance, i.e. between human and teleost fish. Pennachio and coworkers has confirmed the gene regulatory potential of these deeply conserved human elements by employing transgenic mice assay [15]. Combined employment of comparative genomics and expression pattern analysis has assisted us to explicitly assign these human enhancers to their target gene bodies.

**Figure 1:**
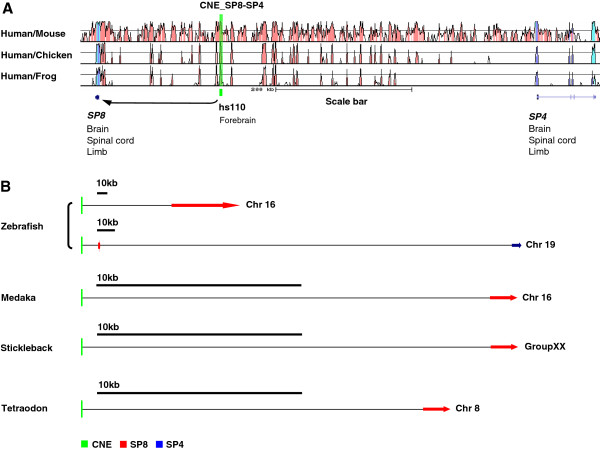
**Human CNE-enhancers associated with target genes using orthology mapping.** (**A**) Comparative syntenic analysis of human, mouse, chicken and frog orthologous loci depicts the conserved presence of both the paralogous *SP4* and *SP8* in the nearest vicinity of the CNE-enhancer (hs110: light green vertical line). Analogy in the expression pattern of the CNE-enhancer and both of these paralogs suggest the association of this CNE-enhancer with both *SP4* and *SP8*. (**B**) Increasing the depth of our synteny comparison by including orthologous loci from teleost fish lineage, it became evident that only *SP8* maintains its physical linkage with the CNE-enhancer in fish. Based on uninterrupted physical proximity over longer period of evolutionary time (450 Myr) *SP8* was considered as probable target for this CNE-enhancer. Genes are color-coded. Direction of arrow depicts the direction of gene transcription. Light green vertical line depicts the position of CNE-enhancer. Horizontal black line depicts scale.

Teleost fish are preferred for syntenic comparison because synteny comparison of human with closely related vertebrate species (mammals) often exhibits the conservation of large number of genes in the vicinity of the CNE-enhancer that reflects the short evolutionary distance and slow rate of neutral divergence among mammals, making it difficult to pinpoint the target gene body of functionally defined human enhancer elements. For instance, comparative analysis of an enhancer region (VISTA enhancer ID: hs110) residing on Homo sapiens autosome 7 (Hsa7: 21,003,280-21,004,750) revealed its conserved syntenic association with paralogous genes *SP4* and *SP8* in mouse, chicken and frog (Figure 1A). Furthermore, reported endogenous expression pattern (MGI:RNA *in-situ* hybridization) of both *SP4* (brain and spinal-cord) and *SP8* (brain and spinal-cord) exhibits harmony with the CNE-enhancer induced *LacZ* expression in transgenic mice assay (Figure 1A). Thus, tetrapod specific synteny comparison and endogenous expression pattern inspection of bracketing genes fails to assign the precise target gene body. To solve the puzzle, orthologous teleost fish loci were included in the synteny comparison (Figure 1B). Careful inspection of genes in the neighborhood of fish CNE_*SP8*-*SP4* revealed that only *SP8* gene maintains the syntenic association with this enhancer in zebrafish, medaka, stickleback and tetraodon (Figure 1B). It therefore appears that SP8 gene is an evolutionary conserved target of human CNE_*SP8*-*SP4* (Figure 1B). By using this systematic methodology we pinpointed specific target genes for the total of 192 human CNE-enhancer elements (Table 1, for detailed list see Additional file 1: Table S1).

**Figure 2:**
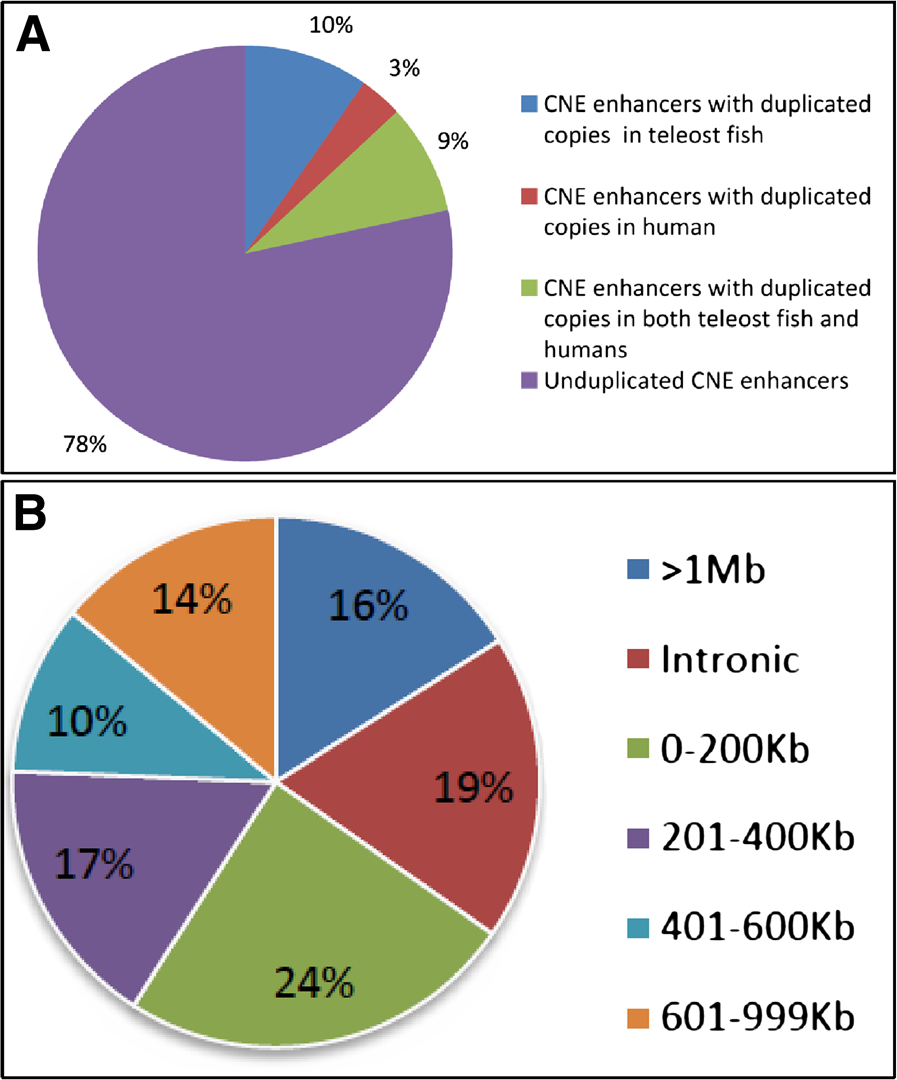
**Human CNE-enhancers duplication history and their genomic range of action.** (**A**) This pie chart shows the extent of duplication events in our selected subset of CNE-enhancers (192 enhancers in total). The majority of our selected enhancers are unduplicated. About 10% of the enhancers have duplicated copies only in teleost fishes, 9% are duplicated before teleost-tetrapod split and 3% enhancers have duplicated copies only in human/tetrapod. (**B**) This pie chart represents the distribution of distances between CNE-enhancers and target gene bodies to estimate the optimal distance at which an enhancer can access its concerned promoter. About 19% of enhancers are within the introns of their predicted target gene bodies, 24% are within a range of 0-200 kb, 17% are at a distance of 201-400 bp, 10% lie in the range of 401-600 kb, 14% in the range of 601-999 kb and 16% of the enhancers are located at distance of >1 Mb from their target genes.

**Figure 3:**
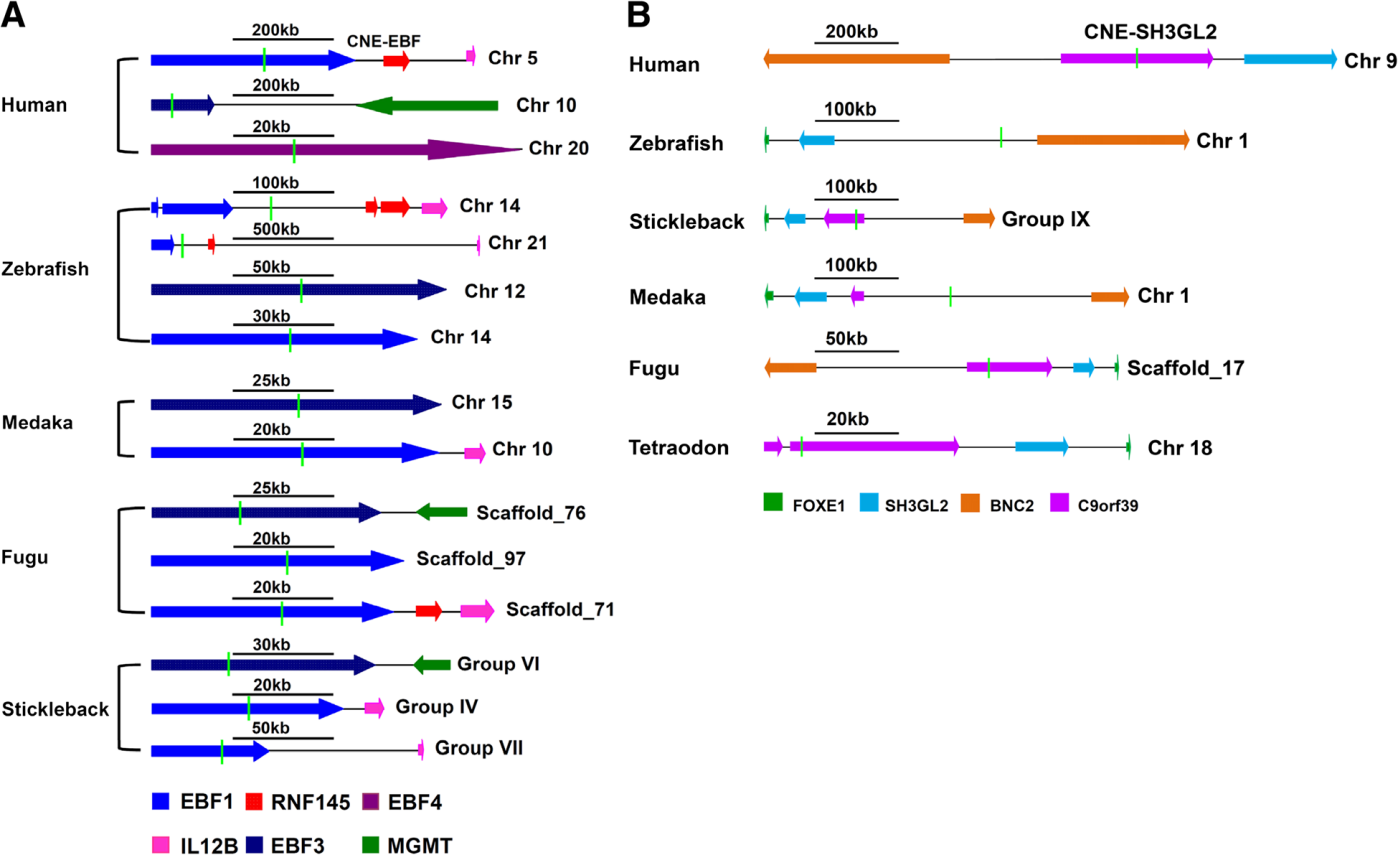
**Predicting the target genes of human enhancers by paralogy and orthology mapping of locus architecture.** (**A**) CNE-enhancers when duplicated are retained with their target genes. Comparing the genic content of *CNE-EBF* containing paralogous loci in human genome and their orthologous loci in multiple fish lineages unmistakably suggests that duplicated copies of human *CNE*-*EBF* enhancer (hs230) are associated with the regulation of paralogous copies of *EBF* family members, i.e. *EBF1, EBF3* and *EBF4*. (**B**) Unduplicated CNE-enhancer (hs529) is associated with its target gene *SH3GL2* by tracing the differential loss of unrelated bystander genes among orthologous loci. Genes are color-coded. Direction of arrow depicts the direction of gene transcription. Light green vertical line depicts the position of CNE-enhancer. Horizontal black line depicts scale.

Gene duplication is thought to be a major driving force in evolutionary innovation by providing raw material from which novel gene functions and expression patterns may arise [21]. Estimation of duplication pattern of selected set of human CNE-enhancers (192) revealed that 17/192 (8.85%) CNE-enhancers have duplicated copies in both teleost fish and tetrapod representatives. 20/192 (10.42%) have duplicated copies only in teleost fish representatives whereas 6/192 (3.12%) CNE-enhancers have duplicated copies only in human/tetrapods (Figure 2A). Duplicated CNEs enable us to unambiguously associate the total of 42/192 human enhancers to their respective target gene only on the basis of synteny comparison (Table 1), because a gene undergoes duplication with all of its *cis*-regulatory elements making genomic regulatory blocks (GRBs) which also harbor some bystander genes which get depleted from GRBs over evolutionary time (Figure 2A and Figure 3A). Thus only duplicated enhancers retain with paralogous copies of their target genes (Figure 2A and Figure 3A). For instance, in a BLAST search of a CNE-enhancer (hs230) residing on Hsa5 (158,340,962-158,342,611) significant hits were found on two other human chromosomal locations, i.e. Hsa10 and Hsa20. While comparing the human paralogous loci architecture, 2 Mb on each side of the CNE, triplicate CNE copies were found to exhibit syntenic association with three paralogous genes of EBF family, i.e. *EBF1* (Hsa5), *EBF3* (Hsa10), and *EBF4* (Hsa20) (Figure 3A). Resuming the analysis we searched out orthologous counterparts of these human triplicated CNE-enhancers in teleost fish genome (zebrafish, *Fugu*, medaka and stickleback). Intriguingly, like human genome this enhancer element was found to occur in multiple copies in each of the teleost genome analyzed: four copies in zebrafish, three copies in *Fugu*/stickleback and two copies in medaka (Figure 3A). Careful inspection of genic contents around paralogous copies of fish CNEs revealed that like human genome only *EBF* family members maintain physical linkage with each copy of this enhancer in teleost fish. Differential loss of bystander genes like *MGMT*, *IL128* and *RNF* from the enhancer containing orthologous and paralogous loci explicitly leads to the conclusion that this human CNE-enhancer (CNE_*EBF*) is controlling the spatio-temporal expression of human *EBF* family members (Figure 3A).

Among the unduplicated set of CNE-enhancers (149/192) 45 regions were assigned to a single target gene only on the basis of synteny comparison (Table 1, for detailed list see Additional file 1: Table S1). For example, BLAST based search of a CNE-enhancer (hs529) residing on Hsa9 (17,322,200-17,324,371) identified putative orthologs of this human interval in zebrafish, medaka, *Fugu*, stickleback and tetraodon genomes (Figure 3B). Synteny comparison of human, zebrafish, medaka, *Fugu*, stickleback and tetraodon orthologous loci revealed the differential loss of *BNC2*, *C9orf39* and *FOXE1* genes (positioned in the vicinity of CNE-enhancer) from the corresponding loci suggesting them as bystanders. *SH3GL2* was the only gene in this human locus which maintains physical linkage with the CNE-enhancer in all examined genomes. Therefore, the gene regulatory potential of this CNE-enhancer (CNE_ *SH3GL2)* was assigned to human *SH3GL2* gene (Figure 3B).

**Figure 4:**
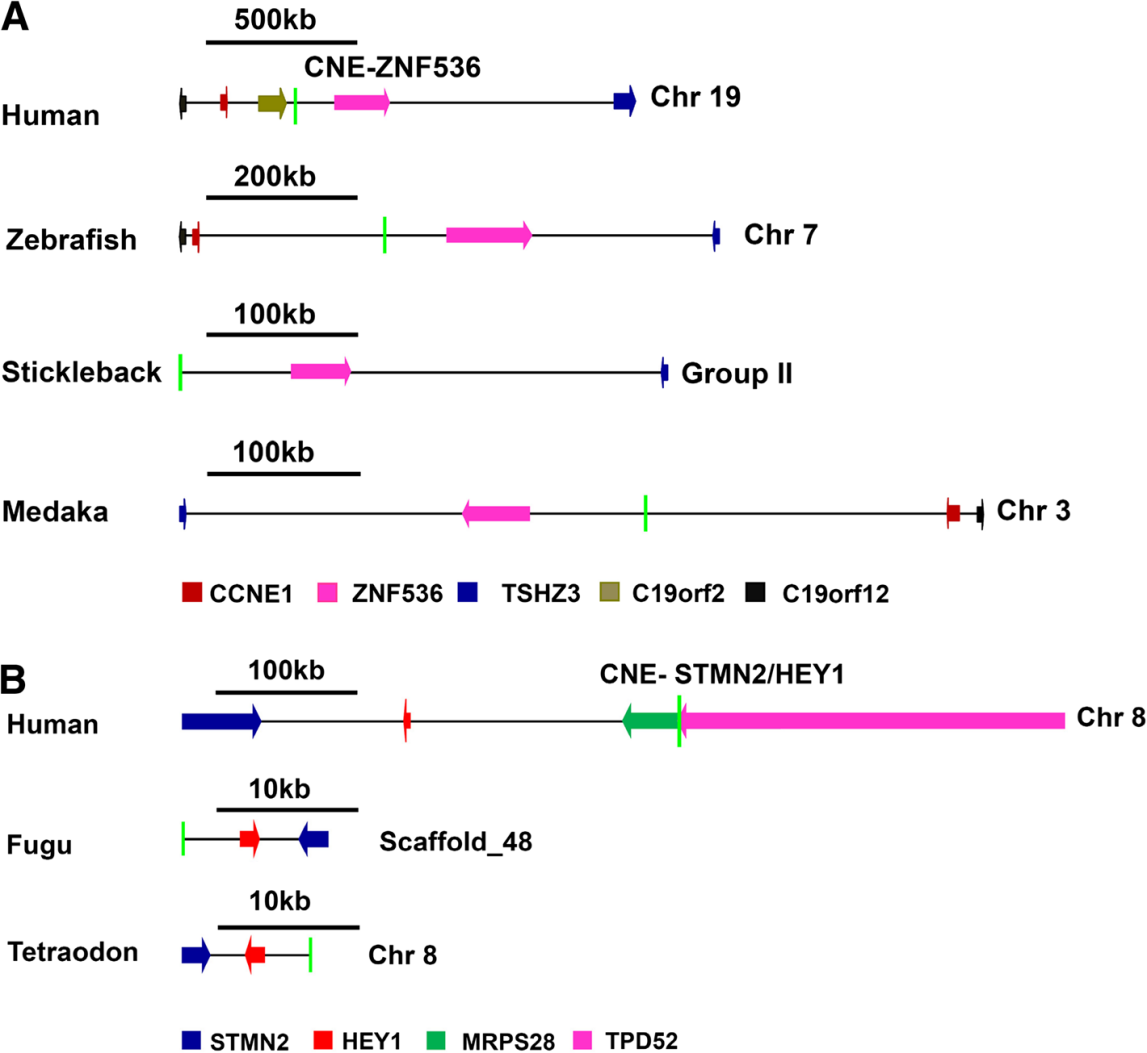
**CNE-enhancer induced reporter expression was compared with the endogenous expression of neighboring genes to find their probable target.** In those cases where comparative synteny analysis alone was not sufficient to establish unambiguous associations among CNE-enhancers and their target gene bodies, we carefully compared the CNE-enhancer induced reporter expression pattern with the reported endogenous expression pattern of neighboring genes. (**A**) Both *TSHZ3* and *ZNF536* genes depict conserved syntenic associations with a human CNE-enhancer (hs858)*,* suggesting either or both of these genes are under the regulatory control of this enhancer. However comparing the reporter expression induced by this CNE-enhancer with the reported endogenous expression pattern of *TSHZ3* and *ZNF536* depicts a precise analogy in the expression domain of *ZNF536* and this enhancer interval. (**B**) CNE-enhancer (hs1305) within an intron of human *MRPS28* gene is not associated with the promoter of the same gene, rather applying the same strategy discussed above in panel A we infer that this CNE-enhancer is acting at a distance of ~150.8 kb on *HEY1* and *STMN2* genes. Genes are color-coded. Direction of arrow depicts the direction of gene transcription. Light green vertical line depicts the position of CNE-enhancer. Horizontal black line depicts scale.

Very often the situation arose when syntenic comparison alone was not sufficient to pinpoint single target because more than one gene were showing conserved syntenic association with the CNE-enhancer element in all evaluated genomes (Additional file 2: Figure S1). In this situation, reporter expression pattern induced by CNE-enhancer was compared manually (via images) with the reported endogenous expression pattern of candidate neighboring genes. Analogy in the expression pattern of CNE-enhancer and one of the many conserved genes solved the puzzle (Additional file 1: Table S1 and Additional file 3: Table S2). This strategy enabled us to assign a single target gene to 56/149 unduplicated CNE-enhancers (Table 1). For instance, during the syntenic comparison of the orthologous loci of human CNE-enhancer (hs858) on Hsa19 (30,747,057-30,748,648), two genes *TSHZ3* and *ZNF536* are considered as putative target genes because both of them showed conserved syntenic association with the CNE-enhancer (Figure 4A). However, when expression pattern of *TSHZ3* and *ZNF536* was monitored only *ZNF536* showed harmony in expression with the CNE-enhancer (Figure 4A).

In 48/149 unduplicated CNE-enhancers we noticed that more than one syntenically conserved gene (2 or 3) are showing analogy in expression pattern with the CNE-enhancer. In this case, two or more genes are declared as target for the relevant CNE-enhancer (Table 1). For instance, a human CNE-enhancer (hs1305) on Has8 (80,874,361-80,876,746), revealed conserved syntenic association with *HEY1* and *STMN2* in all the compared orthologous loci (Figure 4B). Furthermore, reported endogenous expression pattern of both of these genes matches with the reporter expression pattern induced by the CNE-enhancer (Additional file 3: Table S2). Therefore, we assigned both *HEY1* and *STMN2* as target genes for this human enhancer region (Figure 4B).

### Range of action of human cis-acting sites

**Figure 5:**
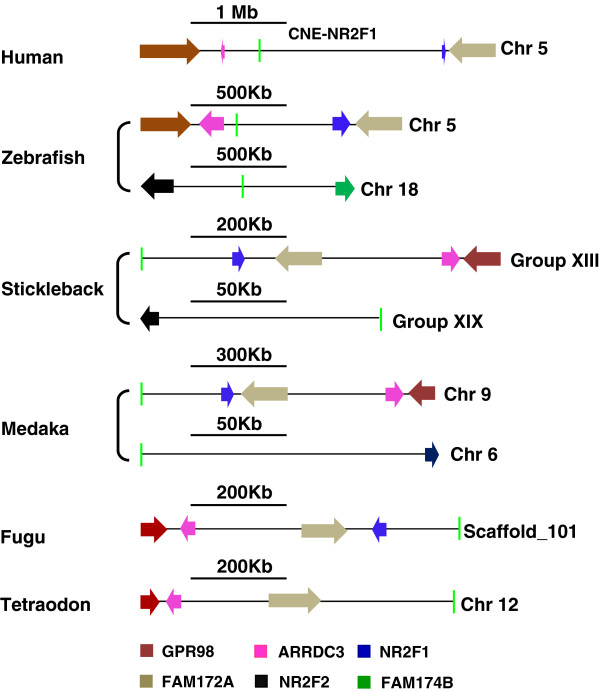
**Human enhancer regions can access their target promoters from a distance of ~2 MB.** Evolutionary conserved syntenic association between a CNE-enhancer (hs191) and human *NR2F1* and harmony in their expression pattern domains in mice infers the functional association among them. The distance between the transcription start site of human *NR2F1* and concerned *cis*-acting site is ~2 MB. Genes are color-coded. Direction of arrow depicts the direction of gene transcription. Light green vertical line depicts the position of CNE-enhancer. Horizontal black line depicts scale.

Once the enhancers were assigned to their probable targets we then set out to use these associations as a training dataset to gauge the maximum distance at which an enhancer can act upon its target gene body. Distance between the enhancer and its target gene is of significant importance because it is an important aspect to understand regulatory mechanism of CNEs and there are also several human genetic disorders that are associated with the disruption in the genomic distance between enhancer and its associated gene [20]. We estimated the percentage of human CNE-enhancers whose target gene bodies are positioned within the ranges, e.g. 0-200 kb, 201-400 kb, 401-600 kb, 601-999 kb and even >1 Mb (Figure 2B). We also counted the number of CNEs positioned within the intronic intervals of their target genes. These results show that a substantial proportion of human enhancers (36/192) reside within the intronic interval of genes they regulate (intragenic) (Figure 2B). Our data further shows that more than a third of human enhancers (77/192) are > 400 kb away from their assigned gene body (Figure 2B). Intriguingly, 40% of these long-range enhancers (31/77) have predicted targets at a distance of >1 Mb. Among these extreme long-range enhancers (31 enhancers) nine are positioned >1.5 Mb away from their target genes (Additional file 4: Table S3). For instance, comparative syntenic analysis of a human CNE-enhancer (hs191) residing on Chr5q14.3 (91,036,888-91,038,899) and comparison of reported endogenous expression pattern of neighboring genes revealed that this *cis*-acting site regulates the hindbrain specific expression of human *NR2F1* gene from a distance of 1.9 Mb (Figure 5).

It is often assumed that *cis*-acting regulatory sites act on the nearest neighboring genes and therefore the conventional search space for enhancers includes immediate upstream or downstream intervals of genes of interest. However, some examples exist where enhancers skip nearby promoters and specifically act on distantly located genes [19]. For instance, tissue specific expression of developmentally important *SHH*[20] and *SOX9*[22] genes were found to be regulated by enhancers positioned ~1 MB away from their transcription start site. Intriguingly, in this study substantial proportion (31/192) of enhancer-target gene associations are established at ≥ 1 Mb genomic interval and thus firmly establishing the fact that long-range spatial interaction among regulatory sites and their target gene bodies are not rare exceptions but occur on pervasive scale (Figure 2B and Additional file 4: Table S3). Consequently, this would imply that assignment of full complement of regulatory sites that act upon a single gene of interest can be seriously impeded by extreme site separation and this could in turn hinder attempts to associate disease causing non-coding mutations with their concerned gene bodies. Rigorously defined enhancer-target gene associations established in this study enable us to underscore the idea that linear proximity (among regulatory sites and target genes) rule is inadequate for the identification of *cis*-regulatory regions and thus extends the previously established maximum search space for enhancers from 2 Mb DNA template around the gene of interest to 4 Mb DNA template (2 Mb on either side) around the gene of interest (Additional file 4: Table S3).

### CNS specific transcriptional factor code

In higher eukaryotes the cell type specific or temporal specific influence of enhancer on their target genes is implemented through interactions of these *cis*-regulatory modules with TFs. The co-occurrence of distinct set of TFs (heterotypic clustering) with each type of binding site represented many times within the same regulatory region (homotypic clustering) are the key features of TF interaction networks in complex metazoan [23]. An important feature of homotypic site clustering is that it facilitates cooperative binding of factors that interacts to moderate or weaker sites [24]. Numerous transcription factors work in concert to regulate target genes in a developmental, cell, or tissue-specific manner. Typically, specific type of cooperativity (similar set of distinct TFs) is required to regulate diverse set of genes exhibiting temporally and spatially synchronized expression (co-expressed genes) [25,26]. For example, skeletal-muscle-specific expression of distinct gene sets has been associated with the cooperative interactions among at least five TFs: Mef-2, Myf, Sp1, SRF, and Tef [27]. Prediction of transcription factor cooperativity has been carried out in yeast and human but unfortunately our current knowledge about combinations of TFs that contributes to the tissue specificity of *cis*-regulatory modules is limited. This in turn has limited the large scale bioinformatics study of tissue-specific gene regulation.

Here we seek to identify TFs that act cooperatively to define CNS (central nervous system) specificity of an enhancer. For this purpose, among the selected subset of distant acting developmental enhancers we choose the one for which reproducible CNS-specific activity has been shown *in vivo* in E11.5 mouse embryos [15]. This data set consists of 159/192 elements, majority (118/159) of which are explicitly associated with single target gene (Table 1). Given the fact that a typical binding motif for TF can be as short as 5-8 bp, *in silico* matches to such short motifs occur frequently by chance alone, with many of these predicted sites presumably non-functional. Therefore, a major challenge in computational identification of such motifs that must be overcome is distinguishing functional TFBSs from spurious motif matches. In order to better define biologically relevant combinations of TFs, while analyzing each brain specific *cis*-regulatory module for an input set of known TFs we focused on: (i) evolutionary conservation of each enhancer across human and mouse lineages; (ii) conserved binding motifs that occurred more than once in an enhancer. This stringent criterion combining the technique of phylogenetic foot printing and possibilities of occurrence of homotypic interactions within typical metazoan enhancers reveals that the brain specific *cis*-regulatory modules have evolutionary conserved binding site preferences for SOX5, HFH, SOX17, c-FOS, HNF3β, c-REL, MEF2, nMYC, USF, FREAC, Tal1beta-E47S, NF-kappaB, AML1 and ARNT, the fourteen transcriptional factors (Additional file 5: Table S4).

The CNE enhancers across the 14 TFs of training data set are then developed as a matrix ${\underline{X}}_{159\times 14}$ and non-conserved/non-coding elements (Additional file 6: Table S5) across the 14 TFs of the control data set developed as a matrix ${\underline{Y}}_{100\times 14}$. The two correlation matrices of the training and control data sets developed as *R*_*X*_ = [*r*_*ij*_^2^ ] and *R*_*Y*_ = [*r*_*ij*_^2^ ] and revealed the pattern of TFs existing in the two comparative data sets (Additional file 7: Table S6). In the training data set a specific pattern of TF interactions is exposed by the formation of three distinctive groups of TFs with strong within and poor or no correlation among the groups, leaving aside two TFs: Tal1beta-E47S and MEF. These two are neither correlated between nor with any other member of the three groups formed. Here the findings of *R*_*X*_ = [*r*_*ij*_^2^ ] may be grouped into three clusters configuration as,

➢ Cluster-1: n-MYC, ARNT, USF (each *r*_*ij*_^2^ higher than 69%)

➢ Cluster-2: c-REL, NF-kappaB (*r*^2^ is 30%)

➢ Cluster-3: FREAC, AML-1, c-FOS, HNF3-β, SOX17, HFH, SOX5 (12*%* ≤ *r*_*ij*_^2^ ≤ 54*%*)

The *R*_*Y*_ = [*r*_*ij*_^2^ ] present findings in contrast with the above three cluster structure found from the *R*_*X*_ = [*r*_*ij*_^2^ ]. Apart from a highly correlated group of three TFs similar to cluster-1, not any other group structure is seen with the remaining TFs (Additional file 7: Table S6) in the control data set. A presumption of a non-interactive pattern of TFs in the control data set may be considered here.

The probability table (Table 2) provides a similar perception of a group structure of TFs present in the ${\underline{X}}_{159\times 14}$ and non co-occurrence pattern of TFs in ${\underline{Y}}_{100\times 14}$. It is readily seen that the least of an *P*(*TF*)_*i*_ in group-2 (the above cluster-3) of the training data set is not less than 59% (Table 2). Higher the *P*(*TF*)_*i*_ more likely is the co-occurrence of TFs in the elements (the CNS enhancers in the training and non-conserved/non-coding elements in the control data sets). In contrast, a lesser *P*(*TF*)_*i*_ may not be attributed to a less likely co-occurrence pattern of a TF. Since a *P*(*TF*)_*i*_ simply defines a degree that a TF is likely to occur in an element, so lower *P*(*TF*)_*i*_ are not mere reflections of less likely co-occurrence of TFs within the elements. Yet the likelihood of the co-occurrence pattern of these TFs cannot be overruled, since any TF that is less frequent across the elements may be co-occurring with another TF or TFs. The occurrence of all the fourteen TFs in the control data set are less frequent with 0.06 ≤ *P*(*TF*)_*i*_ ≤ 0.51 compared with the proportion of TFs in group-2 of the training data set (Table 2). This quantifies the significance of likely co-occurrence of TFs in group-2 of the training data set.

**Table 2:** Probability table of transcription factor binding sites in training and control data sets

**Sr. No**	**TFs**	***P*(*TF***_***i***_**) in training data set**$\left({\underline{X}}_{159\times 14}\right)$	***P*(*TF***_***i***_**) in control data set**$\left({\underline{Y}}_{100\times 14}\right)$
1	Tal1b-E47S	54/159=0.34	6/100=0.06
2	NF-kappaβ	29/159=0.18	10/100=0.10
3	n-MYC	74/159=0.46	7/100=0.07
4	ARNT	61/159=0.38	17/100=0.17
5	USF	61/159=0.38	7/100=0.07
6	c-REL	58/159=0.36	20/100=0.20
7	MEF	55/159=0.35	10/100=0.10
8	FREAC	94/159=0.59*	14/100=0.14
9	AML-1	94/159=0.59*	25/100=0.25
10	c-FOS	119/159=0.74*	23/100=0.23
11	HNF-3beta	119/159=0.74*	31/100=0.31
12	SOX17	136/159=0.85*	44/100=0.44
13	HFH	150/159=0.94*	51/100=0.51*
14	SOX-5	151/159=0.95*	44/100=0.44

* TFs with *P*(*TF*_*i*_) greater than 0.50 defined as group-2. The table present Sr. No 8 to 14 in Training data set as the only group of TFs with high probability of TFs so the most likely co-occurring TFs in CNS brain specific enhancers compared to TFs with *P*(*TF*_*i*_) less than 0.5 in training and control data sets.

**Figure 6:**
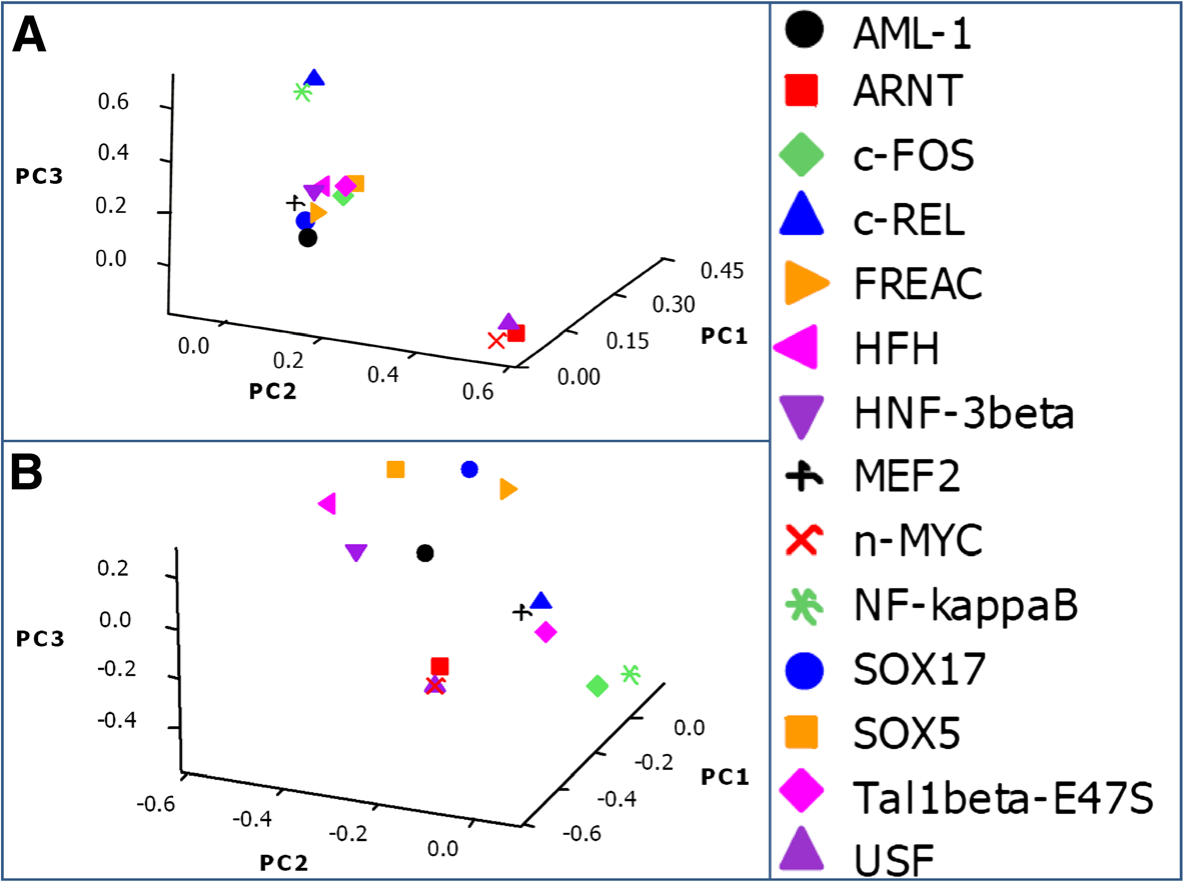
**3D loading plots. A**: Central nervous system specific human enhancers exhibit three distinctive and internally compact clusters exposing the interactive pattern of TFBSs. **B**: The control data set of human non-conserved and non-coding elements do not present any vivid cluster structure for the fourteen TFBSs that formed internally closely packed clusters in CNS specific enhancers (A), thus elucidating the significance of clusters in panel **A**. TFBSs are color coded.

Final identification of the cluster structures in the binding site preferences of the training and control data set is achieved with the application of Principal Component Analysis (PCA). The PCA on ${\underline{X}}_{159\times 14}$ and ${\underline{X}}_{100\times 14}$ using *R*_*X*_ = [*r*_*ij*_] and *R*_*Y*_ = [*r*_*ij*_] respectively validated the heterotypic clustering pattern of TFs perceived so far in the training data set, in a 3D loading plot of the first three principal components (PCs). The first three PCs explained 60% of the total variation present in the patterned correlated data set of ${\underline{X}}_{159\times 14}$. The 3D loading plot (Figure 6B) of the first 3 PCs derived from PCA on control data exposed a scenario of non-interactive pattern of TFs across the non-conserved and non-coding elements, a wide contrast to the interactive pattern of TFs across the CNS enhancers in the training data set (Figure 6A). The 3D loading plot (Figure 6A) exhibits clarity of three distinct sets of closely packed TFs, i.e. three cluster configurations of TFs. The cluster configuration presented coincide with the cluster configuration perceived in *R*_*X*_ = [*r*_*ij*_^2^ ], in addition to merging the two insignificant TFs: Tal1beta-E47S and MEF in cluster-3. The two TFs with *P*(*TF*)_*i*_ less than 0.36 are co-occurring with the TFs packed in cluster-3 so are merged in it. Thus cluster-3 in the training data set takes the form,

❖ Cluster-3: Talb-E47S, MEF, FREAC, AML-1, c-FOS, HNF3-β, SOX17, HFH, SOX5.

We may conclude that there are three distinct clusters of TF internally interactive and showing brain specific *cis-*regulatory modules binding site preferences in the training data set. The statistical significance of pattern of TFs interactions in the training data set exposed are well supported by the probability table (Table 2), the correlation matrix (Additional file 7: Table S6) interpreted as *R* = [*r*_*ij*_^2^ ] and are further statistically significant with the control results of non-conserved and non-coding elements.

To validate *in silico* predicted tissue specific TF code, an essential prerequisite is to depend upon experimentally determined relevance of TFs to the tissue of interest. Therefore, we consulted the literature and MGI [28] Gene Expression Database (GXD) project, and investigated the reported RNA *in-situ* hybridization based endogenous gene expression pattern for each of the predicted TF. Intriguingly, we found that our predictions are enriched in CNS specific expression during early mouse development (Table 3). Thus biological knowledge provides a crucial support to the quality of *in silico* predicted cooperation among specific set of transcription factors to regulate the CNS specific co-expressed genes. This analysis not only provides the list of TFs that may play a crucial role in CNS specific gene regulation but also provides information about cooperation between distinct set of factors (Figure 6). Furthermore, CNS specific transcription factor code defined in this study can act as a priori knowledge in large scale bioinformatics studies aimed at identifying *cis*-regulatory modules involved in neural tube specific gene regulation.

**Table 3:** **List of transcription factors having over-representative occurrence in brain specific *cis*-acting sites**

**Transcription factor**	**Protein-related information**	**Known endogenous expression pattern**	**Source**
AML1	RUNT-type transcription factor	spinal cord, hindbrain	[29,30]
ARNT	Helix-loop-helix DNA-binding	spinal cord, forebrain, midbrain, hindbrain	[31,32]
cFOS	Basic-leucine zipper (bZIP) transcription factor	hindbrain	[33,34]
c-REL	Immunoglobulin-like fold	CNS (components not defined yet)	[35,36]
FREAC	Winged helix-turn-helix transcription repressor	CNS (components not defined yet)	[37,38]
HFH	Winged helix-turn-helix transcription repressor DNA	CNS (components not defined yet)	[39,40]
HNF3B	Winged helix-turn-helix transcription repressor DNA	Spinal-cord, forebrain midbrain, hindbrain	[41,42]
MEF2	myocyte-specific enhancer factor 2A	forebrain, hindbrain	[43,44]
NF-KappaB	Immunoglobulin-like fold	spinal cord, forebrain, midbrain, hindbrain	[45,46]
nMYC	Helix-loop-helix DNA-binding	spinal cord, forebrain, midbrain, hindbrain	[47,48]
SOX17	High mobility group, HMG1/HMG2	spinal cord, forebrain, midbrain, hindbrain	[49]
SOX5	High mobility group, HMG1/HMG2	spinal cord, hindbrain, midbrain	[50,51]
Talbeta-E47S	Helix-loop-helix DNA-binding	spinal cord, forebrain, midbrain, hindbrain	[52,53]
USF	Helix-loop-helix DNA-binding	spinal cord, forebrain, hindbrain	[38,54]

This table also provides references of literature (last column) that addresses the type/function and endogenous expression pattern of transcription factors.

## Conclusion

Metazoan *cis*-regulatory landscape is complex, with modular organization and widespread spatial distribution around the target genes. This complexity hampers attempt to localize and catalog the entire repertoire of enhancers that orchestrate spatially and temporally diverse expression patterns for single gene of interest. Recent relatively high throughput transgenic mice assay based studies generated the genome-wide experimentally validated data set for hundreds of human enhancers. Regulatory activities of each of these enhancers were confirmed through enhancer induced *LacZ* reporter expression in E11.5 mice embryos and tissue specificity of expression was also described. Such genome-wide collections of enhancer data set are expected to contribute immensely in understanding i) structural anatomy of metazoan enhancers ii) mechanisms of enhancer-target gene interactions iii) comprehensively catalog the genetic regulatory circuits for developmentally critical genes iv) role of *cis*-regulatory mutations/alterations in development and disease. However, the utility of these *in vivo* characterized enhancers for variety of biological applications requires their systematic association with target gene bodies. This study is an attempt to systematically associate subset of these functionally defined enhancers with their target genes and thus establishing regulatory interactions for dozens of human genes. These explicit associations would enable the screening of this subset of enhancers for those pathogenic mutations not affecting the coding sequences of concerned genes but disrupting the functionality of these *cis*-acting regulatory sites and thus altering temporal, spatial and quantitative aspects of gene expression. Furthermore, assigning enhancers to *bona fide* target genes assisted us to gauge maximum range at which an enhancer can access its target promoter and delineated the fact that in human genome long-range regulatory interactions occur more frequently and involve longer distances than was previously anticipated. Maximum range of enhancer action reported in this study should serve as a guideline when analyzing the chromosomal deletions/rearrangements associated disorders; such as locus alternations are known to disrupt communication among distant regulatory sites and their target genes.

Gene regulation is exerted by cooperative interactions among TFs that binds to clusters of sites within *cis*-regulatory regions. Distinct *cis*-regulatory modules direct reporter expressions selectively in a particular cell/tissue specific manner are likely to interact with similar set of TFs and thus defining the TFBSs code for co-expressed genes. In this respect, given the abundant set of experimentally defined regulatory sequences, which is sufficient to direct expression of a reporter gene in a cell-specific pattern and list of TFs that are known to be relevant to that tissue, it is possible to construct biologically relevant, tissue specific, complex heterotypic TFs cluster model. Under this assumption, among the CNE-enhancers linked to their target genes we analyzed the large subset of regions that direct gene expression selectively to CNS and defined combinatorial heterotypic interactions of multiple TFs that are likely to bind to typical CNS specific *cis*-regulatory module. This analysis not only figures out the generalized structure of typical CNS specific enhancer, but has established TF interaction network that can be used as training data set for large scale identification of CNS specific enhancers.

## Methods

### In vivo dataset

Experimentally verified catalog of human enhancers which is the basis of this study was obtained from VISTA Enhancer Browser [17]. The core dataset of the VISTA Enhancer Browser consists of experimental *in vivo* data of human and mouse tissue-specific enhancers [17]. These enhancer regions were initially identified by evolutionary sequence conservation or by ChIP-seq [17]. Subsequently these putative enhancer sequences are tested in a transgenic mouse assay to validate their *in vivo* function and to determine their tissue specificity [17]. Elements that show reproducible and consistent *LacZ* reporter gene expression among at least three mouse embryos are presented as positive enhancers elements, whereas elements for which no reporter expression is observed among a minimum of five transgenic embryos are defined as negative [17]. The dataset of positive embryos is reported comprehensively in terms of sequence coordinates, flanking genes, annotated expression patterns and details of reproducibility of each structure, images of individual embryos, and series of histological sections [17]. Currently this database host experimentally confirmed 975 human enhancer sequences, directing reproducibly the reporter expression in diverse set of embryonic domains at embryonic day 11.5. We restricted our analyses to 192 human-teleost fish conserved sequences (Table 1, for detailed list see Additional file 1: Table S1). For each of the candidate enhancer element, we retrieved from VISTA enhancer browser information such as, genomic sequence, VISTA enhancer ID, conservation depth, and name of neighboring genes, tissue specificity and image data (Additional file 3: Table S2).

### Assigning the target gene to human CNE-enhancers

In order to associate each of the selected subset of human CNE-enhancer with their *bona fide* target gene, we analyzed the neighboring genomic context using the UCSC [55] and Ensembl genome browsers [56] and drafted a locus map depicting the flanking genes spanning at least 2 MB interval on either side of CNE-enhancer (Additional file 2: Figure S1). The sequence similarity of selected subset of CNEs between the human and the teleost fish genome suggests that they are functional in both lineages. It would be appropriate then to speculate that the target genes would also be the same in both species. Given this assumption, comparative picture of these CNE-enhancers bearing human synteny maps was observed in currently available teleost genome (zebrafish, tetraodon, stickleback, medaka and *Fugu*) by using Multi-species view option at Ensembl genome browser [56]. This allowed us to map carefully the genomic context of evolutionary conserved human enhancers in corresponding zebrafish, tetraodon, medaka, stickleback and *Fugu* loci (Additional file 2: Figure S1). Among these anciently diverged genomes (human-teleost fish, >450 Mya) uninterrupted physical linkage between CNE-enhancer and one or more neighboring genes was taken as an evidence of functional association (Additional file 1: Table S1 and Additional file 2: Figure S1). To further confirm these associations, for one or more genes depicting evolutionary conserved physical association with CNE-enhancer, the endogenous expression pattern of the mouse ortholog was obtained from MGI [28]. We preferred available gene expression obtained by RNA *in-situ* hybridization. Reporter gene expression induced by the selected CNE-enhancer is also captured from the VISTA enhancer browser database [17]. We manually compared the image data of transgenic mouse embryos expressing *LacZ* reporter gene under the influence of CNE-enhancer element with the RNA *in-situ* hybridization based endogenous expression data of genes residing in the neighborhood of enhancer sequence (Additional file 3: Table S2).

Duplicated copies of selected subset of CNE-enhancers (dCNEs) were searched through BLAST based similarity searches at Ensemble and UCSC genome browsers [55,56]. We categorized the duplicated enhancers into those, with duplicated copies only in fish lineage (only a single counterpart in human), duplicated copies only in human (only a single counterpart in fish), and the ones that contains duplicated copies in both fish and human lineages (Figure 2A). Duplicated CNE-enhancer facilitated further, to link them explicitly with their target gene through paralogy mapping, i.e. by identifying the genes that have paralogs in the genomic regions that harbor at least two dCNEs from the same family. Paralogy relationship among target genes of duplicated set of enhancers was generated by using paralogy prediction pipeline of Ensembl genome browser where maximum likelihood phylogenetic gene trees (generated by TreeBeST) play a central role [57].

### Estimation of range of action of human CNE-enhancers

Orthology mapping, paralogy mapping and expression pattern analysis helped in assigning *bona fide* target genes to total of 192 human CNE-enhancers. These large numbers of enhancer-target gene associations enables us to define the genomic range of regulatory activity for human enhancer sequences. For this purpose we calculated the distance between the CNE-enhancers and transcriptional start site of their predicted target genes and then examined the distribution of distances. We partitioned the range of enhancer action as, CNE-enhancers embedded within intronic intervals of target gene (intragenic), CNE-enhancers whose target gene lies within the ranges, e.g. 0-200 kb, 201-400 kb, 401-600, 601-999 and >1 Mb (Figure 2B). Our data shows that 36/192 (18.75%) enhancers are located within the intronic interval of genes they regulate, 47/192 (24.48%) enhancers are within a range of 0-200 kb from their assigned gene, 32/192 (16.67%) enhancers are within a distance of 201-400 kb, 19/192 (9.89%) positioned within 401-600 kb from their associated gene, 27/192 (14.06%) separated by a distance of 601-999 kb from their target. Intriguingly, 31/192 (16.14%) enhancers were found to act on their concerned gene body from a distance of >1 Mb (Figure 2B and Additional file 4: Table S3).

### Transcription factor analysis

To establish the central nervous system (CNS) specific transcriptional factor (TF) code we selected 159/192 the subset of human CNE-enhancers that were shown to drove expression in various domains mouse CNS (Additional file 5: Table S4). For this purpose the technique of phylogenetic foot printing was employed on human and mouse orthologous enhancer regions to track the occurrence of evolutionary conserved grouping of transcription factor binding sites (TFBSs) in experimentally verified subset of brain specific enhancers (Additional file 5: Table S4).

Mouse orthologs of human enhancers were obtained through BLAST based similarity searches. Human-mouse conserved transcription factor binding sites in each CNE-enhancer were detected with computer program ConSite [58]. The ConSite screen for conserved TFBSs was performed against the JASPAR database with 85% conservation cutoff, 60 bp window size and 75% transcription factor score threshold settings.

To track cooperative heterotypic interaction among distinct set of TFs within brain specific enhancers, suitable statistical methodologies were employed for their identification and verification. We formulated a multivariate data matrix with *n* (rows) as the sample of enhancers and *p* (columns) the number of TFs for training and control data sets (for control data set see Additional file 6: Table S5). For the materialization of the known biological background that occurrences of TFs in sample of enhancers are not mutually exclusive, the repeated occurrence of a TF is determined by finding the individual probability of the occurrence of a TF (*P*(*TF*)_*i*_ in a sample). Looking for the patterns and structures in TFs, primarily the training data matrix of 159 enhancers across 14 TFs $\left({\underline{X}}_{159\times 14}\right)$ and control data matrix of non-conserved/non-coding elements $\left({\underline{Y}}_{100\times 14}\right)$ are subjected to a two step exploratory data analysis. Computation of probabilities of TFs in (Table 2) and correlation matrices *R*_*X*_ = [*r*_*ij*_^2^ ] (lower diagonal in Additional file 7: Table S6) and *R*_*Y*_ = [*r*_*ij*_^2^ ] (upper diagonal in Additional file 7: Table S6) complete the two steps employed for the initial exploration of patterns of TFs in the control and training data sets respectively. The probability table (Table 2) is a classified presentation of *P*(*TF*)_*i*_ with *P*(*TF*)_*i*_ < 0.5 as members of group-1 and for *P*(*TF*)_*i*_ ≥ 0.5 members of group-2 in the training and control data sets.

The correlation matrices of the data sets are desirable to define clusters of TFs that may covary together among all possible pairs of TFs. For the purpose, the squared correlation coefficient (*R* = [*r*_*ij*_^2^ ]) is interpreted as it indicates a meaningful and practical co-variation among the variables (Additional file 7: Table S6) [59,60].

Principal Component Analysis (PCA), a powerful multivariate exploratory tool is used to identify patterns in specifically high (*P*) dimension, interrelated data sets and express the data sets by highlighting their similarities and differences. For multivariate data sets that are interrelated, appropriate application of PCA is using *R* = [*r*_*ij*_] matrix for eigen analysis. Therefore, PCA will be used as a means of constructing an informative graphical representation of the data set by projecting the data onto a lower dimensional space. In the study, control and training data will be presented in a three dimensions (3D) subspace of the first three PCs [61,62]. The PCs derived by the eigen analysis of correlation matrix (*R* = [*r*_*ij*_]) is a linear combination of the original *p* variables (the TFs) and each PC uncorrelated with the other, meaning these are the new transformed data expressed in terms of the patterns existing in the original data set. The total PCs derived are equal to the number of original variables present in the dataset. The *p* PCs formed are with decreasing order of magnitude of variance of the total variation in the data sets. Thus the first three PCs capturing most of the variation in the data set is visualized in a 3D representation. The coefficient of the variables in each of the linear combination, i.e. the PC is defined as loadings. The magnitude of these loadings represents the importance of each variable present. Thus a 3D representation of the loadings of the first three PCs will identify any cluster structure present in the variables (the TFs), exhibiting the co-occurring pattern of TFs in the control and training data sets.

The comparative analysis of control and training is of major significance in the validation of clusters of known TFs highly represented in human brain specific enhancers.

## Competing interest

Authors declare that they have no competing interest.

## Authors’ contributions

AAA conceived the project and designed the experiments. NP, AM, NI, BFM, RM, UN and AAA performed the experiments. AAA, UN, NP and AM analyzed the data. AAA, NP and UN wrote the paper. All authors read and approved the final manuscript.

## Supplementary Material

Additional file 1: Table S1 Evidences for CNE-enhancer and Target gene Association. Click here for file

Additional file 2: Figure S1 Comparative synteny analysis of genomic regions containing human CNE-enhancers. Click here for file

Additional file 3: Table S2 Describes the names of CNE enhancers as given by (http://enhancer.lbl.gov/), human chromosome location, coordinates (hg19), status of conservation location with respect to putative associated genes. Molecular function/biological process and endogenous expression pattern(as described in Mgi (http://www.informatics.jax.org/) of candidate associated genes is also given. The last column describes the reporter expression induced by human CNE enhancer in transgenic mice assay as describes by (http://enhancer.lbl.gov/). Click here for file

Additional file 4: Table S3 List of CNE-enhancers which reside >1.5 Mb apart from their target gene. Click here for file

Additional file 5: Table S4 Transcription factor binding sites analysis. Click here for file

Additional file 6: Table S5 Transcription factor binding site analysis of control dataset of human non-coding and non-conserved elements. Click here for file

Additional file 7: Table S6 *R* = [*r*_*ij*_^2^] A Correlation Matrix with Lower Diagonal for Training and Upper Diagonal for the Control data set. Click here for file
