# Supplementary material for: Comparative genomics using teleost fish helps to systematically identify target gene bodies of functionally defined human enhancers
Source: BMC Genomics. 2013 Feb 23;14:122. doi: 10.1186/1471-2164-14-122 (PMC3599049; doi:10.1186/1471-2164-14-122)
Supplement: Additional file 2: Figure S1 — Comparative synteny analysis of genomic regions containing human CNE-enhancers. [file 1471-2164-14-122-S2.pdf]

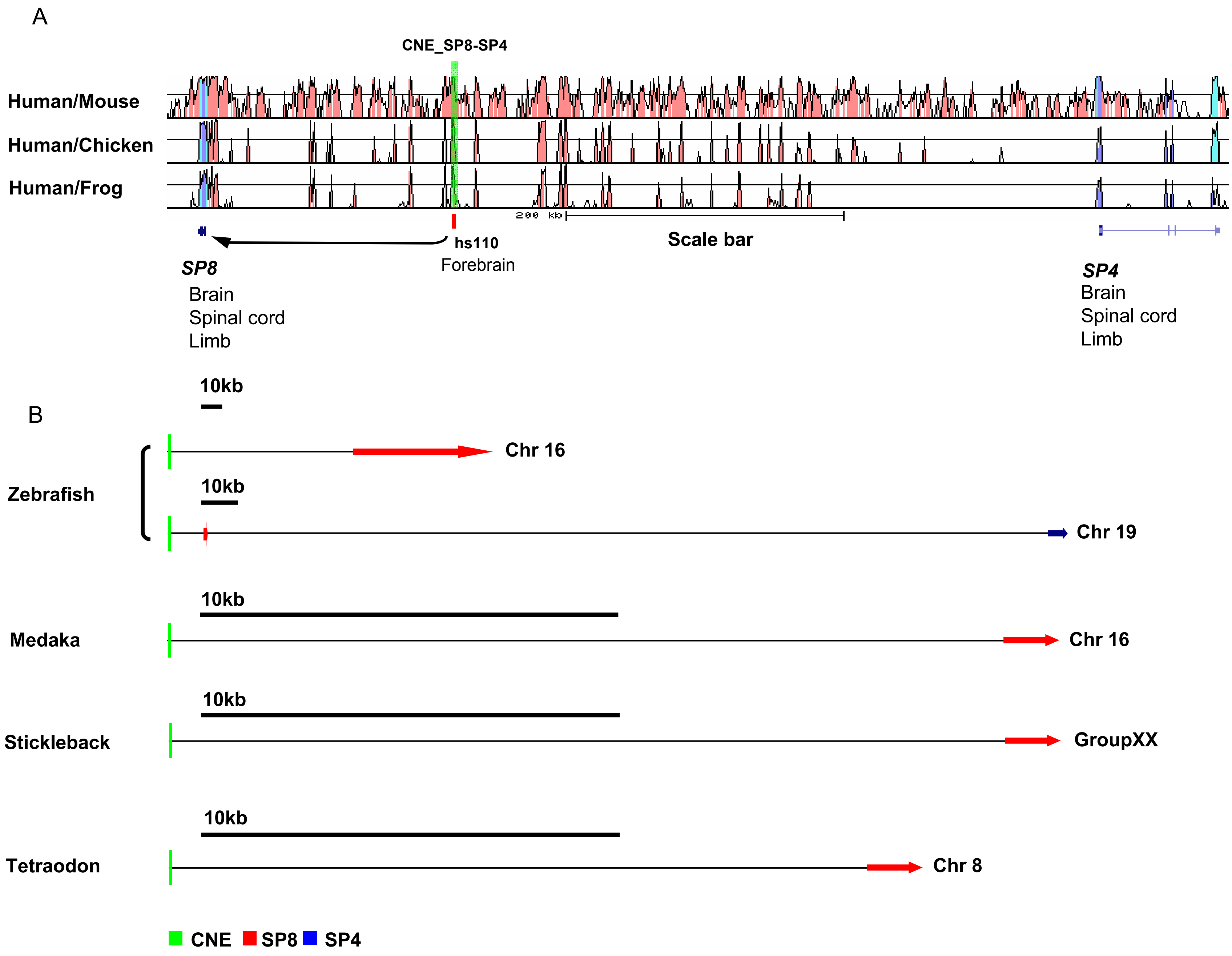

A
CNE_SP8-SP4
Human/Mouse
Human/Chicken
Human/Frog
200 kb
Scale bar
hs110
Forebrain
SP8
Brain
Spinal cord
Limb
SP4
Brain
Spinal cord
Limb
B
10kb
Zebrafish
Chr 16
10kb
Chr 19
10kb
Medaka
Chr 16
10kb
Stickleback
GroupXX
10kb
Tetraodon
Chr 8
CNE
SP8
SP4

Figure 1. Target gene identification of human CNE-enhancers through orthology mapping (hs110).

Extreme evolutionary conservation and spatio-temporal expression analysis of CNEs and their neighboring genes lead to association of CNE enhancers to their respective genes. **(A)** Comparative syntenic analysis of human and amphibian depicts the conservation of both the paralogs *SP4* (blue) and *SP8* (purple) in the nearest vicinity of the CNE (green). Analogy in the expression pattern of the CNE and both of these paralogs suggest the association of this CNE with both *SP4* and *SP8* gene. **(B)** When we increased the depth of our syntenic comparison up till the fishes it became evident that only *SP8* is conserved along with the CNE in fishes proposing it as a target gene for hs110.

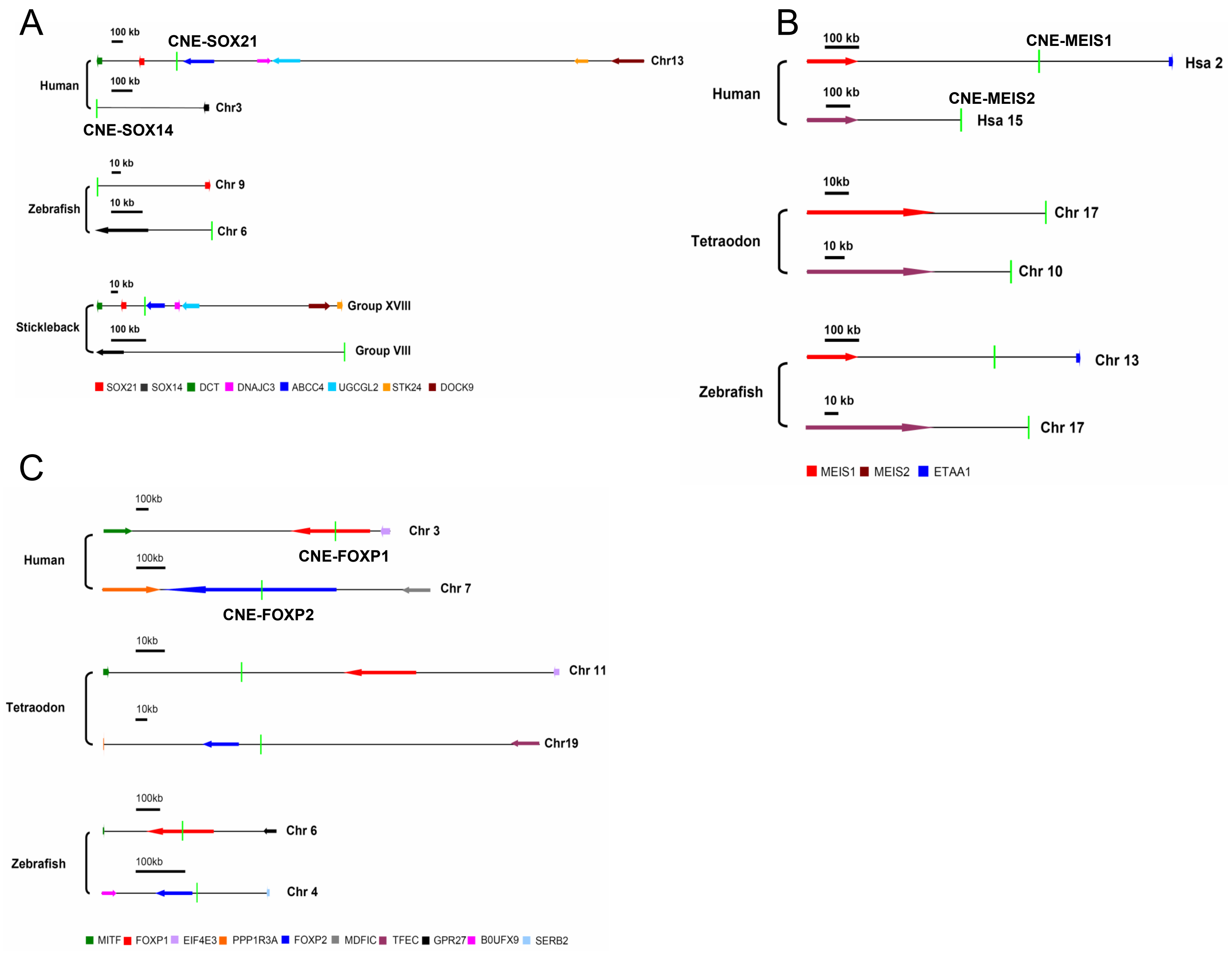
A
100 kb
CNE-SOX21
Chr13
Human
100 kb
Chr3
CNE-SOX14
10 kb
Chr 9
Zebrafish
10 kb
Chr 6
10 kb
Group XVIII
Stickleback
100 kb
Group VIII
SOX21 SOX14 DCT DNAJC3 ABCC4 UGCGL2 STK24 DOCK9
B
100 kb
CNE-MEIS1
Hsa 2
Human
100 kb
CNE-MEIS2
Hsa 15
10kb
Chr 17
Tetraodon
10 kb
Chr 10
100 kb
Chr 13
Zebrafish
10 kb
Chr 17
MEIS1 MEIS2 ETAA1
C
100kb
Chr 3
Human
CNE-FOXP1
100kb
Chr 7
CNE-FOXP2
10kb
Chr 11
Tetraodon
10kb
Chr19
100kb
Chr 6
Zebrafish
100kb
Chr 4
MITF FOXP1 EIF4E3 PPP1R3A FOXP2 MDFIC TFEC GPR27 B0UFX9 SERB2

Figure 2. Predicting the target genes of human enhancers by analyzing their genic environment within and across vertebrate genomes (hs189/hs831/hs187).

Careful analysis of genomic context of human enhancers helped in establishing an unambiguous association between them and target genes. **(A)** Human enhancer-target gene duplicated early in vertebrate history, before tetrapod-teleost split. Conserved association between duplicated enhancers and *SOX* paralogs indicate that these duplicated human enhancers (*CNE-SOX14* and *CNE-SOX21*) are specific to *SOX14* and *SOX21* genes. **(B)** Duplication of human enhancer at the root of vertebrate lineage and preservation of linkage of duplicated enhancers with *MEIS* family members both within and across genomes, unmistakably suggest the specificity of these duplicated cis-regulatory regions (*CNE-MEIS1* and *CNE-MEIS2*) towards *MEIS1* and *MEIS2* promoters. **(C)** Human *FOXP1* and *FOXP2* paralogs harbor duplicated set of enhancers within their introns. The association of these anciently conserved enhancers with *FOXP1* and *FOXP2* genes is confirmed by their physical proximity to teleost fish orthologs of human *FOXP1* and *FOXP2* genes.

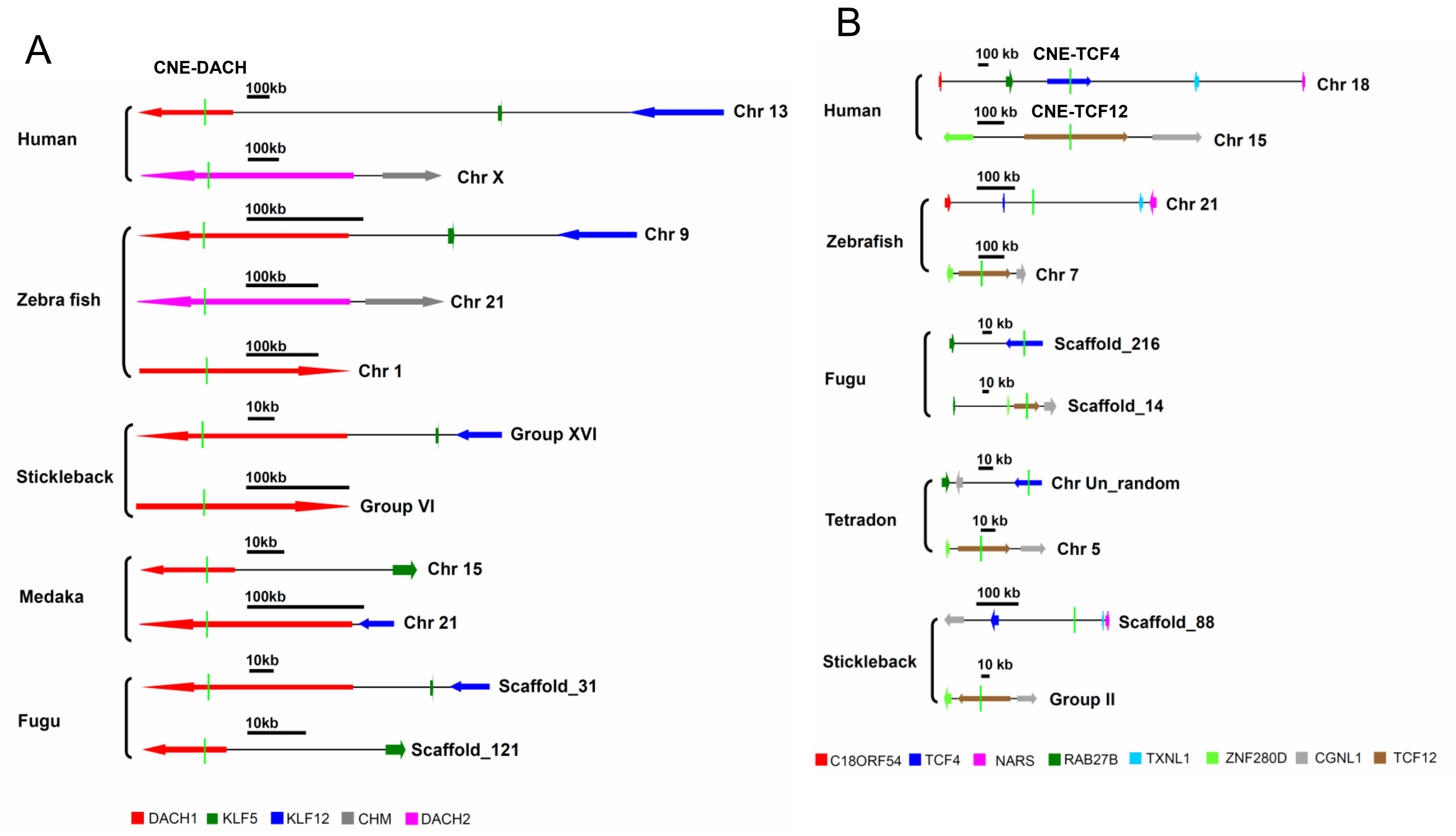
A
CNE-DACH
100kb
Human
Chr 13
Chr X
Zebra fish
Chr 9
Chr 21
Chr 1
10kb
Stickleback
Group XVI
Group VI
Medaka
Chr 15
Chr 21
Fugu
Scaffold_31
Scaffold_121
DACH1
KLF5
KLF12
CHM
DACH2
B
100 kb
CNE-TCF4
Chr 18
CNE-TCF12
Chr 15
Zebrafish
Chr 21
Chr 7
10 kb
Scaffold_216
Scaffold_14
Tetradon
Chr Un_random
Chr 5
Scaffold_88
Group II
C18ORF54
TCF4
NARS
RAB27B
TXNL1
ZNF280D
CGNL1
TCF12

Figure 3. Target gene identification of human CNE-enhancers through orthology mapping (hs137/hs376).

Analyzing the genic environment of human CNE-enhancers in teleost fish orthologous loci and expression pattern of reporter gene helps in identifying their target genes. **(A)** Human CNE-enhancer and target gene duplicated before tetrapod-teleost divergence. Comparative syntenic analysis of human duplicated loci in multiple fish lineages clearly suggest that these duplicated human enhancers are associated with the regulation of human *DACH* paralogs residing on human chromosome 13 and X. **(B)** Duplication of human enhancer at the root of vertebrate lineage and preserved linkage of duplicated enhancers with *TCF* family members both within and across genomes, suggest the specificity of these duplicated CNEs towards *TCF4* and *TCF12* human paralogs.

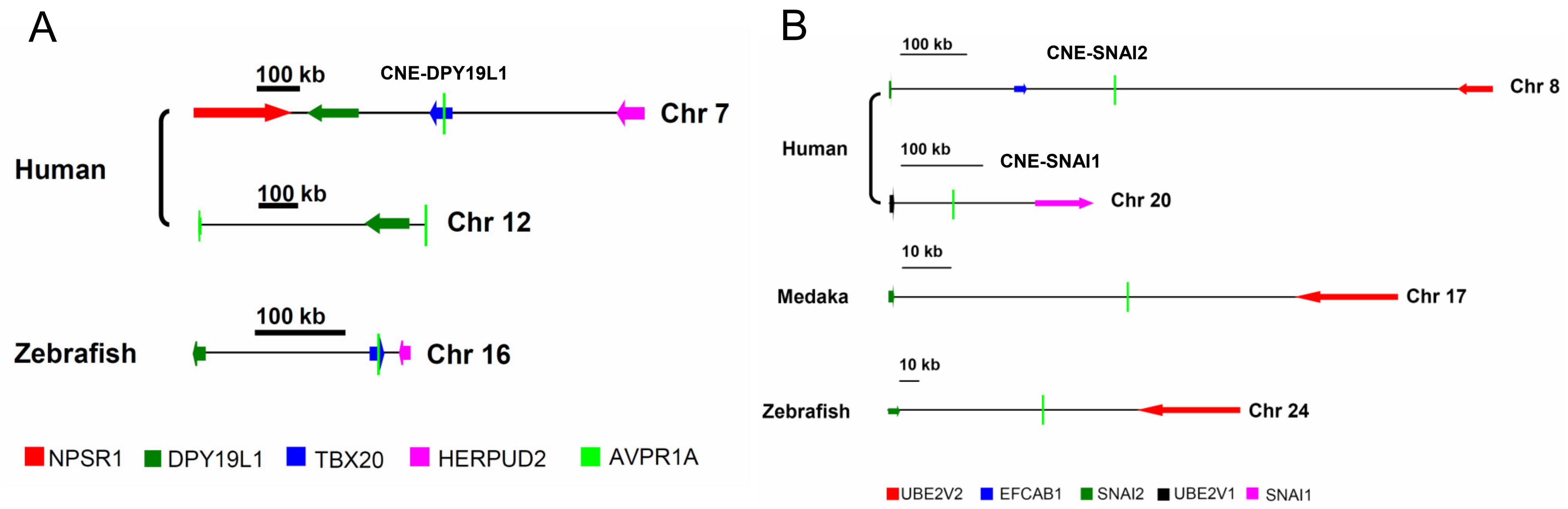
A
100 kb
CNE-DPY19L1
Chr 7
Human
100 kb
Chr 12
100 kb
Zebrafish
Chr 16
NPSR1
DPY19L1
TBX20
HERPUD2
AVPR1A
B
100 kb
CNE-SNAI2
Chr 8
Human
100 kb
CNE-SNAI1
Chr 20
10 kb
Medaka
Chr 17
10 kb
Zebrafish
Chr 24
UBE2V2
EFCAB1
SNAI2
UBE2V1
SNAI1

Figure 4. Target gene identification of human CNE-enhancers through orthology mapping (hs466 /mm466).

Comparative analysis help in assigning the CNE enhancer to its target genes. **(A)** The CNE-enhancer duplicated in human lineage. Careful analysis of genic context of orthologous regions of this element in teleost fish and its duplicated paralogous copy on human chromosome suggests the association of this enhancer with *DPY19L1*. **(B)** The localization of a CNE-enhancer in the intergenic space between *UBE2V2* and *EFCAB1* on human chromosome 8 suggest that this enhancer might be associated with one of these genes but not with *SNAI1* that is present ~ 200 kb upstream of *EFCAB1*. However the careful analysis of genic context of orthologous regions of this element in teleost fish and its duplicated paralogous copy on human chromosome 20 clearly indicates that these duplicated human enhancers (*CNE_SNAI1* and *CNE_SNAI1*) are associated with the regulation of human *SNAI* paralogs.

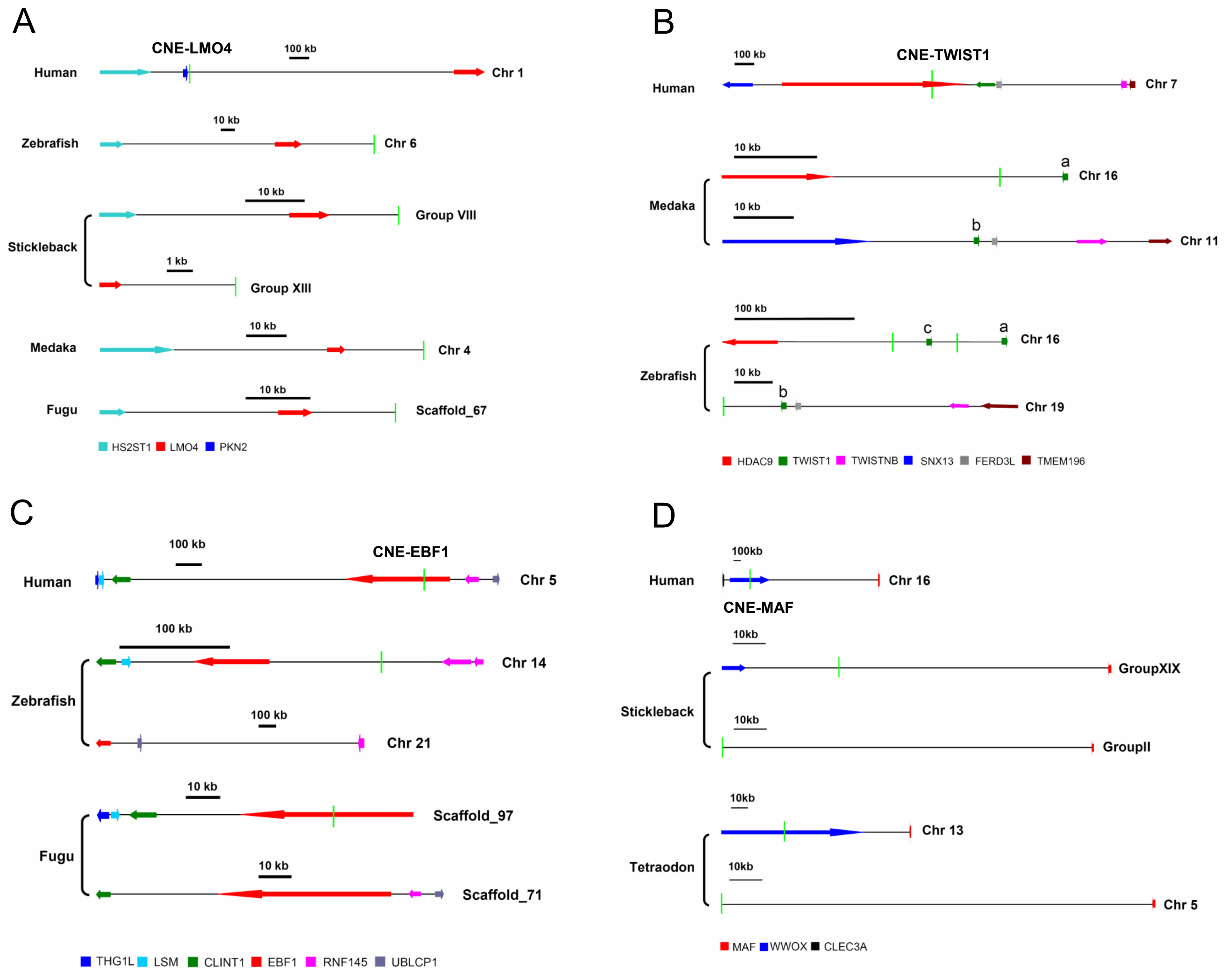
A
CNE-LMO4
100 kb
Human
Chr 1
10 kb
Zebrafish
Chr 6
10 kb
Group VIII
Stickleback
1 kb
Group XIII
10 kb
Medaka
Chr 4
10 kb
Fugu
Scaffold_67
HS2ST1 LMO4 PKN2
B
100 kb
CNE-TWIST1
Human
Chr 7
10 kb
a
Chr 16
Medaka
10 kb
b
Chr 11
100 kb
c
a
Chr 16
Zebrafish
10 kb
b
Chr 19
HDAC9 TWIST1 TWISTNB SNX13 FERD3L TMEM196
C
100 kb
CNE-EBF1
Human
Chr 5
100 kb
Chr 14
Zebrafish
100 kb
Chr 21
10 kb
Scaffold_97
Fugu
10 kb
Scaffold_71
THG1L LSM CLINT1 EBF1 RNF145 UBLCP1
D
100kb
Human
Chr 16
CNE-MAF
10kb
GroupXIX
Stickleback
10kb
GroupII
10kb
Chr 13
Tetraodon
10kb
Chr 5
MAF WWOX CLEC3A

Figure 5. Target gene identification of human CNE-enhancers through orthology mapping (hs174/hs644/hs1022 /hs12) .

Human CNE-enhancers by tracing the genic context of their orthologous copies in teleost fish lineage. **(A)** Human cis-regulatory element positioned in the intergenic space between *PKN2* and *LMO4*, suggesting that this enhancer might be associated with one of these genes. Examining the neighboring genes of this enhancer in teleost fish suggest that *PKN2* is a bystander gene because in all teleost fish analyzed this gene is physically uncoupled from CNE enhancer. Another gene *HS2ST1* present in the neighborhood of human CNE-enhancer (upstream of *PKN2*) is similarly linked to this enhancer in all teleost fish analyzed. However the duplication of this locus in stickleback indicated that this gene is also a bystander gene as in one of the duplicated fragment (on Group XIII) *HS2ST1* ortholog is lost. The only gene in the human locus that preserved its association with this CNE-enhancer in all teleost fish analyzed (even in duplicated loci of stickleback) is *LMO4*. Therefore human *cis*-regulatory element positioned in the intergenic space between *PKN2* and *LMO4* is unambiguously associated with *LMO4* and named *CNE-LMO4*. **(B)** Human CNE-enhancer positioned within the intronic interval of *HDAC9* gene on chromosome 7. Approximately 2 Mb of human locus encompassing this enhancer (containing at least 6 genes) was analyzed for the maintenance of conserved gene contents in teleost fish. The locus appeared to be duplicated in medaka and zebrafish. The differential gene loss from teleost duplicated loci suggest that CNE-enhancer within intragenic interval of human *HDAC9* gene is associated with the regulation of *TWIST1*. It is noteworthy that in zebrafish in addition to locus duplication event an independent gene duplication event occurred that produced two tandem copies of *TWIST1* and associated *CNE-TWIST1*. **(C)** Human CNE-enhancer positioned within the intronic interval of *EBF1* gene on chromosome 5. Comparative analysis of this locus in teleost fish revealed that genomic interval encompassing this conserved enhancer is duplicated in zebrafish and Fugu. It appeared that after duplication of the locus, one copy of this CNE-enhancer had been lost in both fishes. Tracing the correlation between gene loss-enhancer loss/gene retention-enhancer retention in fish duplicated loci suggest that CNE-enhancer within the intragenic interval of human EBF1 might be associated with the regulation EBF1 gene. **(D)** Comparative analysis of a genic context around a CNE-enhancer within the intronic interval of human *WWOX* suggest that this enhancer act at a distance of ~ 2Mb on *MAF* gene.

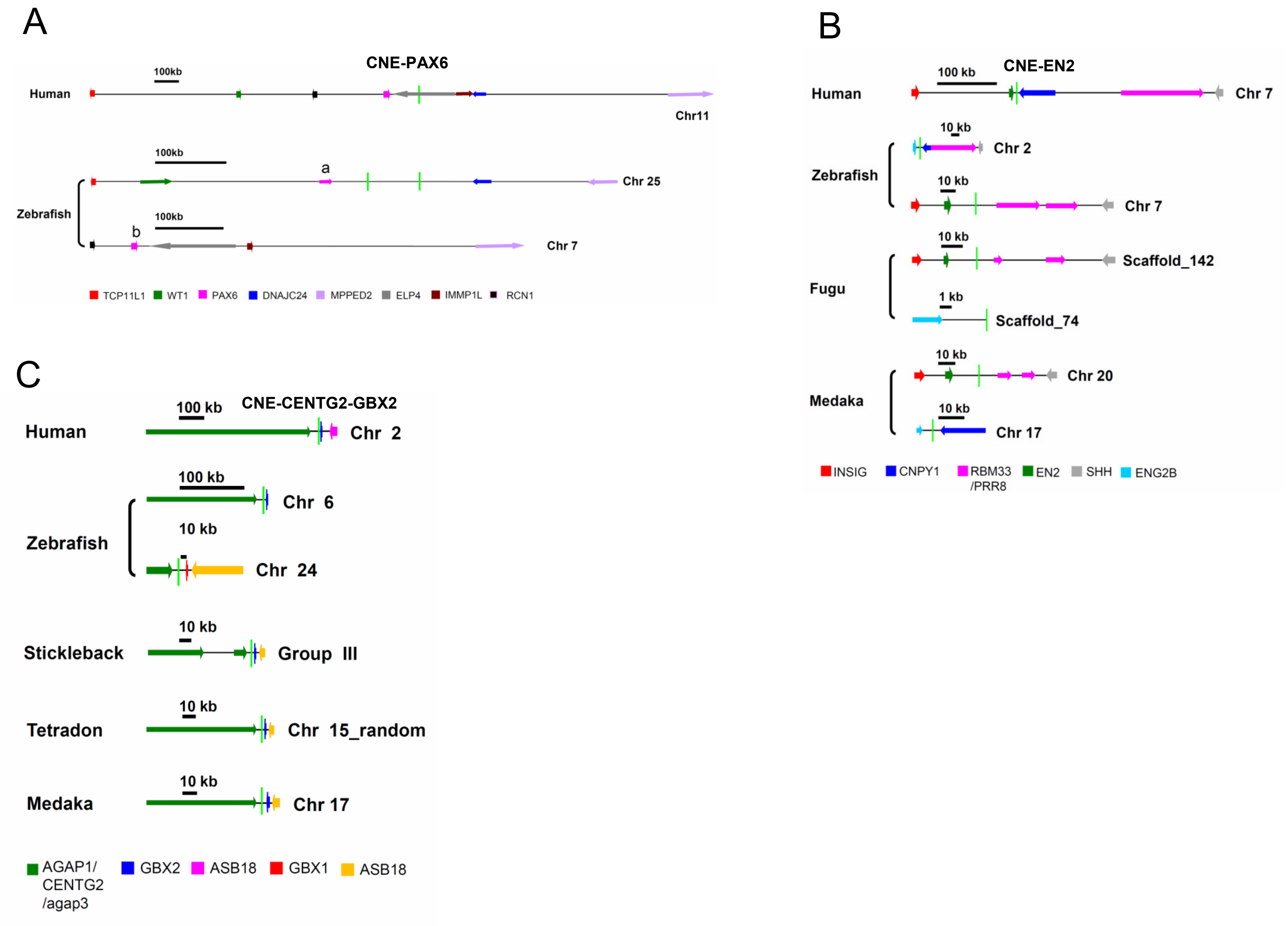
A
CNE-PAX6
100kb
Human
Chr11
100kb
a
Chr 25
Zebrafish
100kb
b
Chr 7
TCP11L1 WT1 PAX6 DNAJC24 MPPED2 ELP4 IMMP1L RCN1
B
CNE-EN2
100 kb
Human
Chr 7
10 kb
Zebrafish
Chr 2
10 kb
Chr 7
10 kb
Fugu
Scaffold_142
1 kb
Scaffold_74
10 kb
Medaka
Chr 20
10 kb
Chr 17
INSIG CNPY1 RBM33 /PRR8 EN2 SHH ENG2B
C
CNE-CENTG2-GBX2
100 kb
Human
Chr 2
100 kb
Zebrafish
Chr 6
10 kb
Chr 24
10 kb
Stickleback
Group III
10 kb
Tetradon
Chr 15_random
10 kb
Medaka
Chr 17
AGAP1/ CENTG2 /agap3
GBX2
ASB18
GBX1
ASB18

Figure 6. Target gene identification of human CNE-enhancers through orthology mapping (hs234/hs1418 /hs249) .

Analysis of genic environment of human CNE-enhancers in teleost fish orthologous genomic intervals helps in identifying their target genes. **(A)** CNE-enhancer within an intron of human *ELP4* gene is associated with neighboring *PAX6* gene. **(B)** Human CNE-enhancer positioned on chromosome 7 probably duplicated in the common ancestor of teleost fish. Comparative syntenic analysis of human locus in multiple fish lineages and redundancy in the CNE induced reporter expression pattern and endogenous expression pattern of *EN2* clearly suggest that this CNE enhancer is associated with the regulation of human *EN2*. **(C)** Conserved positioning of CNE-enhancer within an intergenic space between human *CENTG2* and *GBX2* and all other fish lineages and expression pattern studies suggest that this CNE is associated with either of flanking genes.

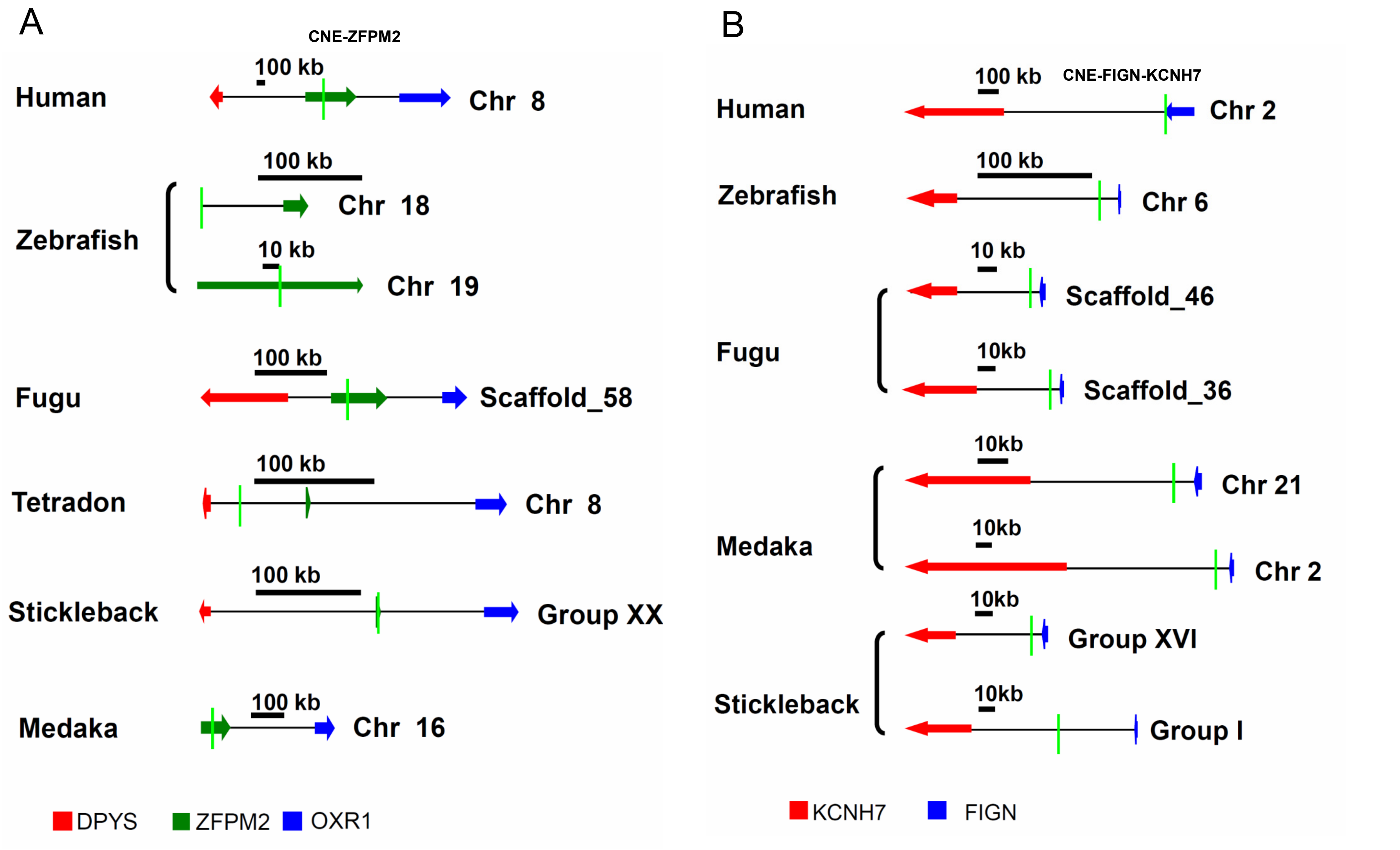

A
CNE-ZFPM2
100 kb
Human
Chr 8
100 kb
Zebrafish
Chr 18
10 kb
Chr 19
100 kb
Fugu
Scaffold_58
100 kb
Tetradon
Chr 8
100 kb
Stickleback
Group XX
100 kb
Medaka
Chr 16
DPYS
ZFPM2
OXR1
B
100 kb
CNE-FIGN-KCNH7
Human
Chr 2
100 kb
Zebrafish
Chr 6
10 kb
Fugu
Scaffold_46
10kb
Scaffold_36
10kb
Medaka
Chr 21
10kb
Chr 2
10kb
Stickleback
Group XVI
10kb
Group I
KCNH7
FIGN

Figure 7. Target gene identification of human CNE-enhancers through orthology mapping (hs195/hs170) .

Assigning the target genes to CNE-enhancers through comparative genomic analysis. **(A)** Human CNE-enhancer is positioned within the intronic interval of *ZFPM2* gene on chromosome 8. Comparative analysis of this locus in teleost fish revealed that genomic interval encompassing this conserved enhancer is duplicated in zebrafish. The conserved linkage of CNE with *ZFPM2* in all lineages and its endogenous expression unmistakably links this CNE with the regulation of *ZFPM2* gene. **(B)** Human CNE enhancer positioned on chromosome 2 duplicated in the common ancestor of teleost fish. Human-fish conserved positioning of CNE-enhancer within an intergenic space between human *FIGN* and *KCNH7* and redundant expression pattern of these two genes suggest the association of this CNE enhancer with either of neighboring genes.

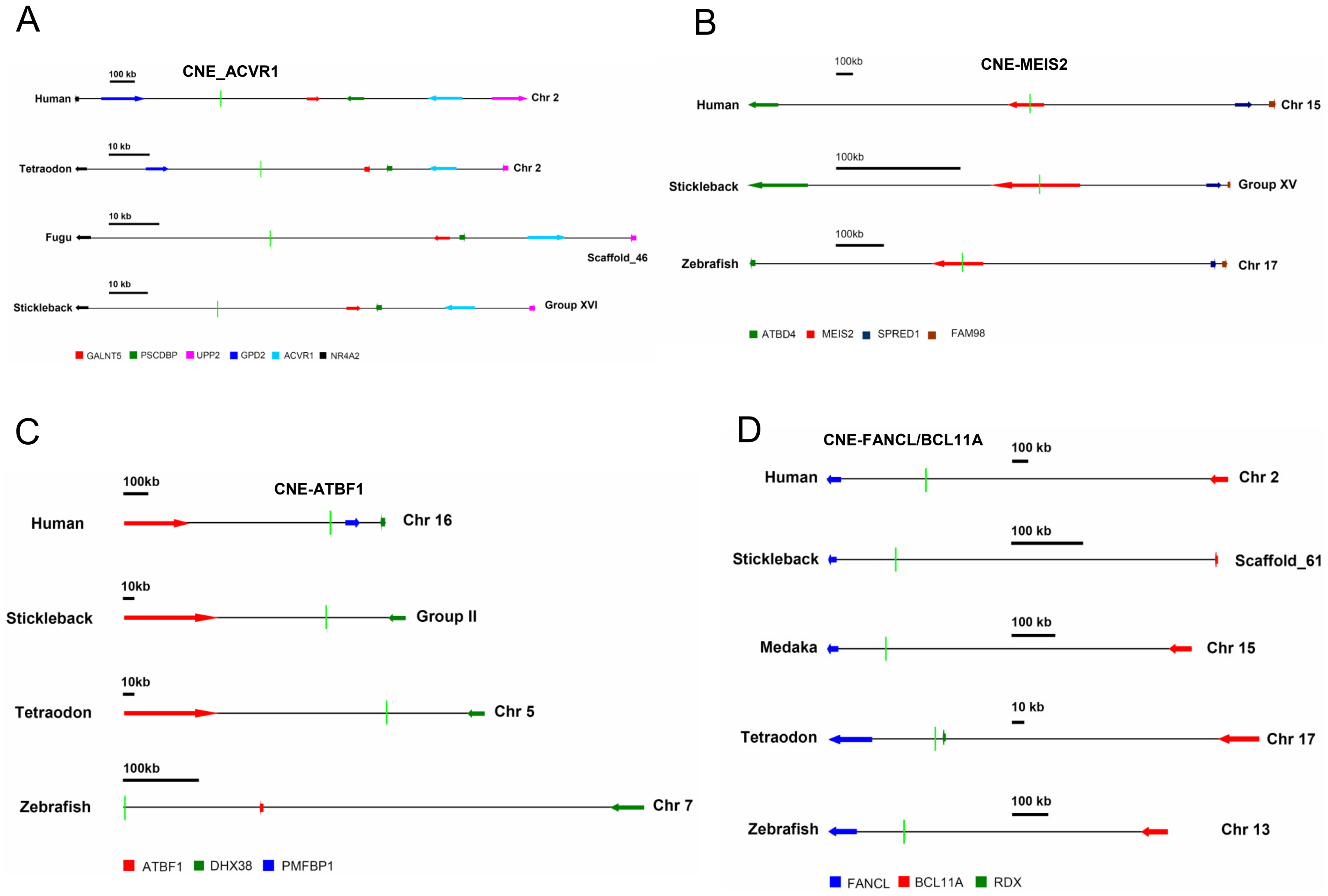

A
CNE_ACVR1
100 kb
Human
Chr 2
10 kb
Tetraodon
Chr 2
10 kb
Fugu
Scaffold_46
10 kb
Stickleback
Group XVI
GALNT5 PSCDBP UPP2 GPD2 ACVR1 NR4A2
B
CNE-MEIS2
100kb
Human
Chr 15
100kb
Stickleback
Group XV
100kb
Zebrafish
Chr 17
ATBD4 MEIS2 SPRED1 FAM98
C
CNE-ATBF1
100kb
Human
Chr 16
10kb
Stickleback
Group II
10kb
Tetraodon
Chr 5
100kb
Zebrafish
Chr 7
ATBF1 DHX38 PMFBP1
D
CNE-FANCL/BCL11A
100 kb
Human
Chr 2
100 kb
Stickleback
Scaffold_61
100 kb
Medaka
Chr 15
10 kb
Tetraodon
Chr 17
100 kb
Zebrafish
Chr 13
FANCL BCL11A RDX

Figure8.Target gene identification of human CNE-enhancers through orthology mapping (hs413/hs181/hs22/ hs1067).

Assigning the target genes to CNE-enhancers through comparative genomic analysis. **(A)** CNE-enhancer within an intergenic space between human *GALNT5* and *GPD2* is associated with *ACVR1*. **(B)** CNE-enhancer within an intron of human *MEIS2* gene is associated with the promoter of the same gene. Other human-fish conserved genes within this locus appeared to be bystanders as their expression pattern is unrelated to the activity of this cis-acting region. **(C)** CNE-enhancer within an intergenic space between human *ATBF1* and *PMFBP1* is associated with *ATBF1* gene. **(D)** Human-fish conserved positioning of a CNE-enhancer within an intergenic space between human *FANCL* and *BCL11A* and redundant expression pattern of these two genes suggest that a single enhancer could interact with more than one neighboring genes.

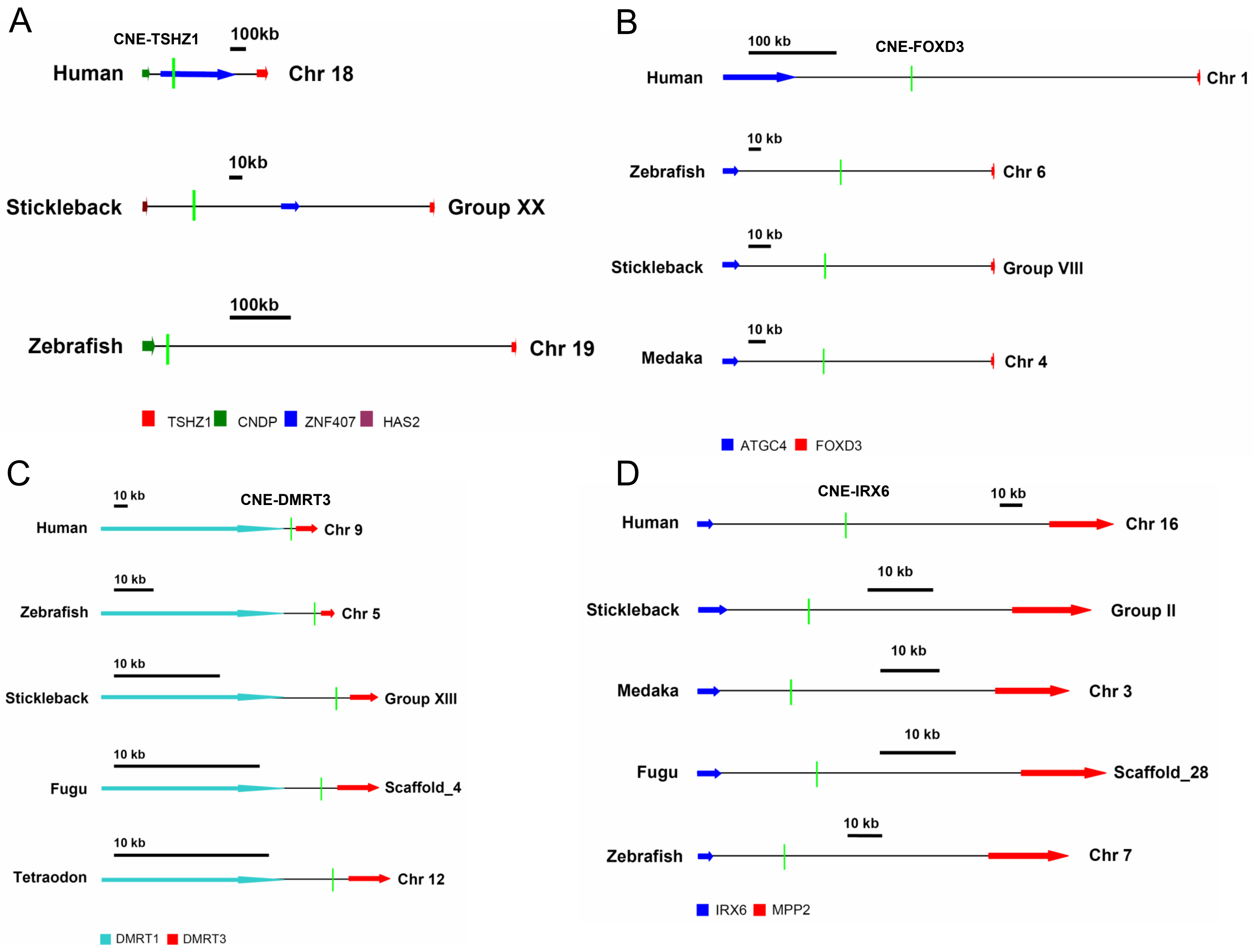
A
CNE-TSHZ1
100kb
Human
Chr 18
10kb
Stickleback
Group XX
100kb
Zebrafish
Chr 19
TSHZ1
CNDP
ZNF407
HAS2
B
100 kb
CNE-FOXD3
Human
Chr 1
10 kb
Zebrafish
Chr 6
10 kb
Stickleback
Group VIII
10 kb
Medaka
Chr 4
ATGC4
FOXD3
C
10 kb
CNE-DMRT3
Human
Chr 9
10 kb
Zebrafish
Chr 5
10 kb
Stickleback
Group XIII
10 kb
Fugu
Scaffold_4
10 kb
Tetraodon
Chr 12
DMRT1
DMRT3
D
CNE-IRX6
10 kb
Human
Chr 16
10 kb
Stickleback
Group II
10 kb
Medaka
Chr 3
10 kb
Fugu
Scaffold_28
10 kb
Zebrafish
Chr 7
IRX6
MPP2

Figure 9. Target gene identification of human CNE-enhancers through orthology mapping (hs378/hs169/Hs112/hs23).

Analyzing the genic architecture of human loci in teleost and comparing the reporter expression domains of CNE-enhancers with neighboring genes expression aided in assigning the *cis*-regulatory elements to human **(A)** *TSHZ1* **(B)** *FOXD3* **(C)** *DMRT3* and **(D)** *IRX6* genes**.**

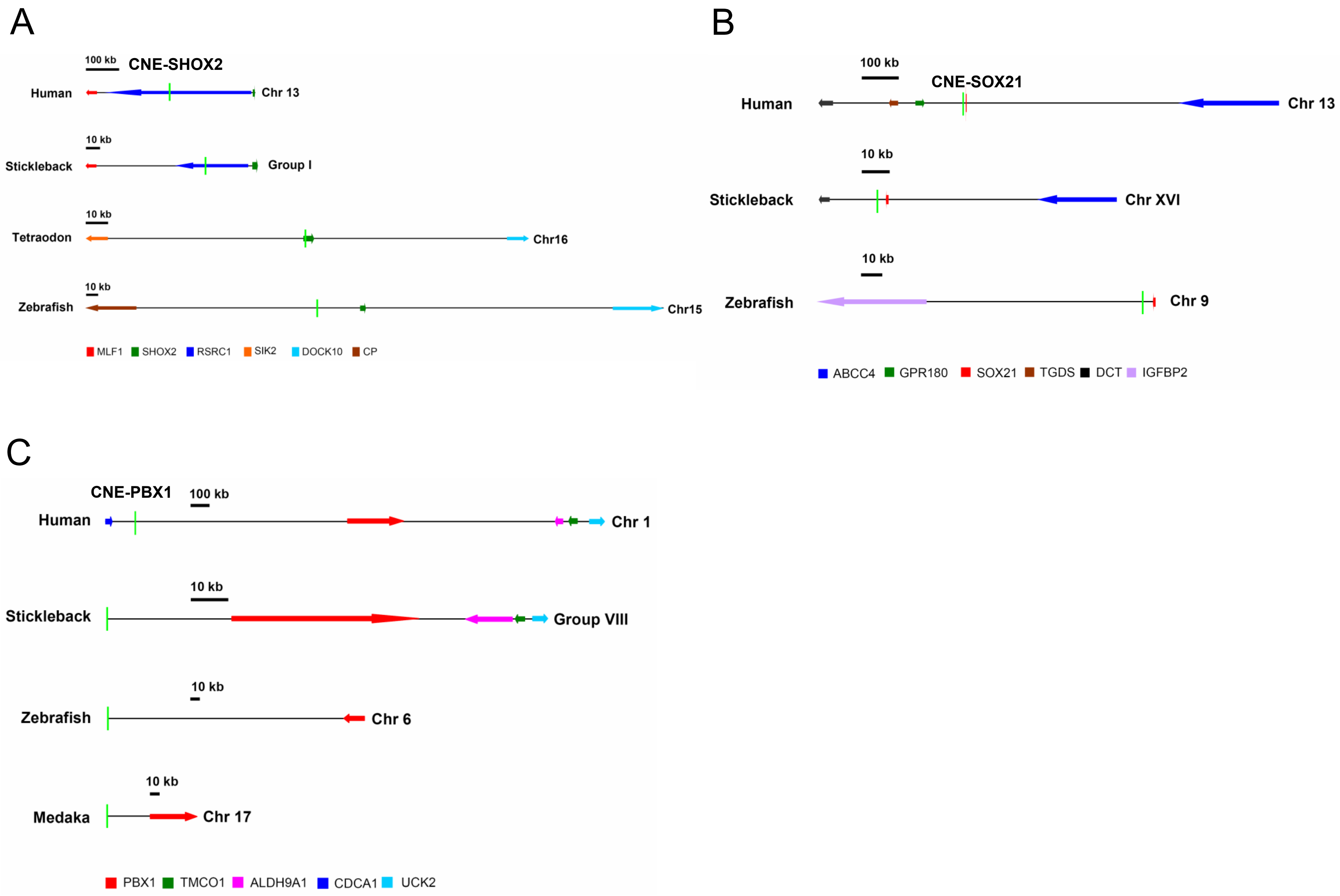
A
100 kb
CNE-SHOX2
Human
Chr 13
10 kb
Stickleback
Group I
10 kb
Tetraodon
Chr16
10 kb
Zebrafish
Chr15
MLF1
SHOX2
RSRC1
SIK2
DOCK10
CP
B
100 kb
CNE-SOX21
Human
Chr 13
10 kb
Stickleback
Chr XVI
10 kb
Zebrafish
Chr 9
ABCC4
GPR180
SOX21
TGDS
DCT
IGFBP2
C
CNE-PBX1
100 kb
Human
Chr 1
10 kb
Stickleback
Group VIII
10 kb
Zebrafish
Chr 6
10 kb
Medaka
Chr 17
PBX1
TMCO1
ALDH9A1
CDCA1
UCK2

Figure 10. Target gene identification of human CNE-enhancers through orthology mapping (hs741/hs488/hs762) .

Analysis of human CNE-enhancers loci in teleost fish orthologous genomic intervals helps in identifying their target genes. **(A)** CNE-enhancer within an intron of human *RSRC1* gene is associated with neighboring *SHOX2*. **(B)** CNE-enhancer within an intergenic space between human *SOX21* and *GPR180* is associated with *SOX21*. **(C)** CNE-enhancer within an intergenic space between human *CDCA1* and *PBX1* is associated with *PBX1*.

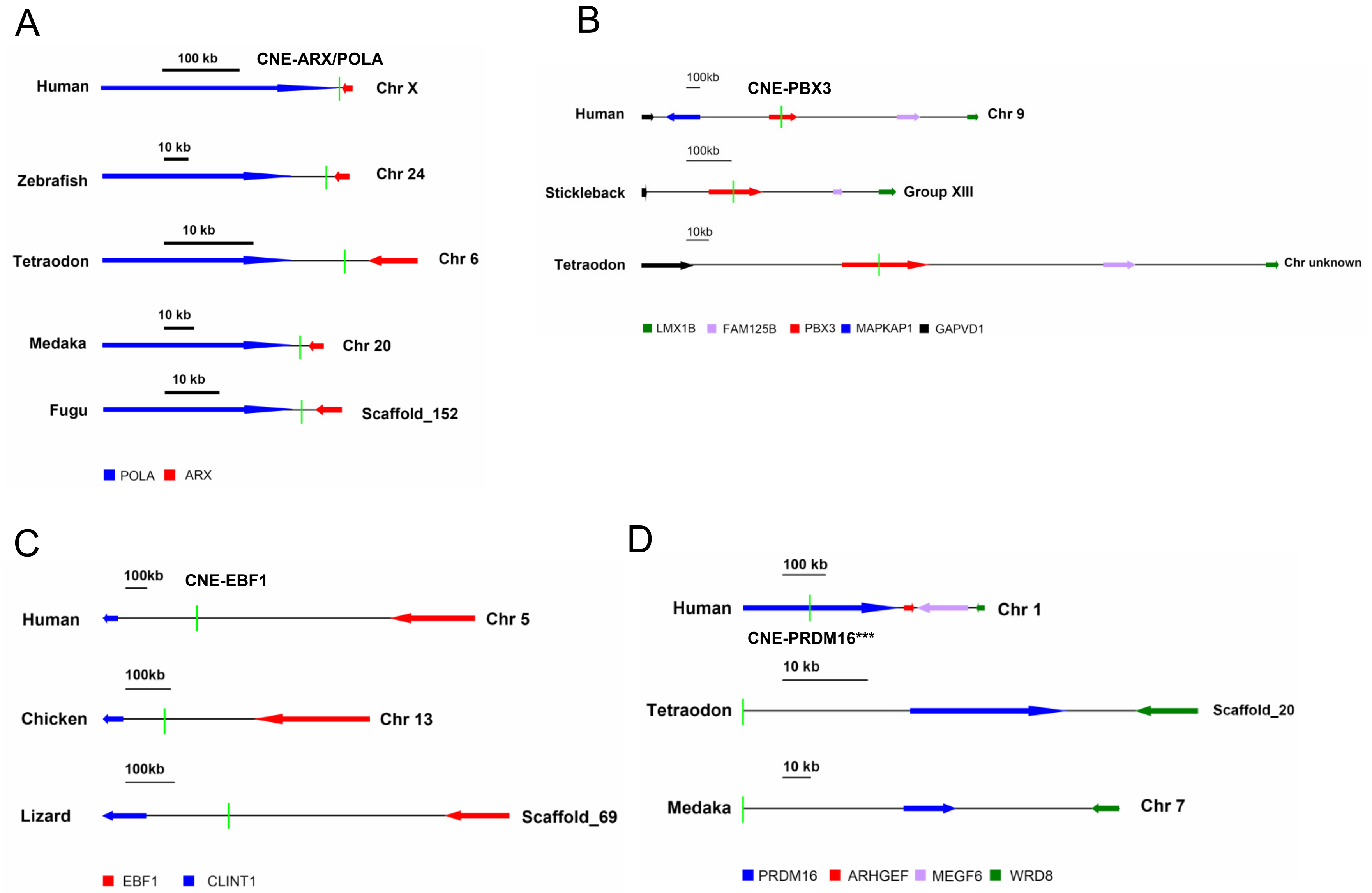
A
100 kb
CNE-ARX/POLA
Human
Chr X
10 kb
Zebrafish
Chr 24
10 kb
Tetraodon
Chr 6
10 kb
Medaka
Chr 20
10 kb
Fugu
Scaffold_152
POLA
ARX
B
100kb
CNE-PBX3
Human
Chr 9
100kb
Stickleback
Group XIII
10kb
Tetraodon
Chr unknown
LMX1B
FAM125B
PBX3
MAPKAP1
GAPVD1
C
100kb
CNE-EBF1
Human
Chr 5
100kb
Chicken
Chr 13
100kb
Lizard
Scaffold_69
EBF1
CLINT1
D
100 kb
Human
Chr 1
CNE-PRDM16***
10 kb
Tetraodon
Scaffold_20
10 kb
Medaka
Chr 7
PRDM16
ARHGEF
MEGF6
WRD8

Figure 11. Target gene identification of human CNE-enhancers through orthology mapping (hs122/hs818/ hs1130/hs705).

Human-fish comparative analysis of human loci and comparing the reporter expression domains induced by CNEs with neighboring genes expression pattern helped in assigning the target genes to CNE-enhancers. **(A)** An intergenic CNE-enhancer showed conserved positioning with respect to *ARX* and *POLA* genes in human and teleost fish lineages. The expression pattern of these genes is highly redundant and in accordance with CNE-enhancer induced reporter expression, and suggest that this enhancer might be influencing the expression of both of its neighboring genes. **(B)** The physical location of human CNE-enhancer within the intronic region of *PBX3* is conserved in its orthologous intervals in fish lineages, along with other bystander genes. However the endogenous expression pattern of *PBX3* matches with that of reporter gene expression, clearly inferring the association of enhancer with *PBX3*. **(C)** Human-fish comparative syntenic analysis revealed the conservation of CNE between *EBF1* and *CLINT1* along with these genes , moreover the expression pattern suggest specificity of this CNE enhancer to human *EBF1*. **(D)** Human-fish comparative syntenic analysis revealed the conservation of CNE and *PRDM16* linkage in both lineages, moreover the expression pattern also suggest specificity of this CNE enhancer to human *PRDM16*.

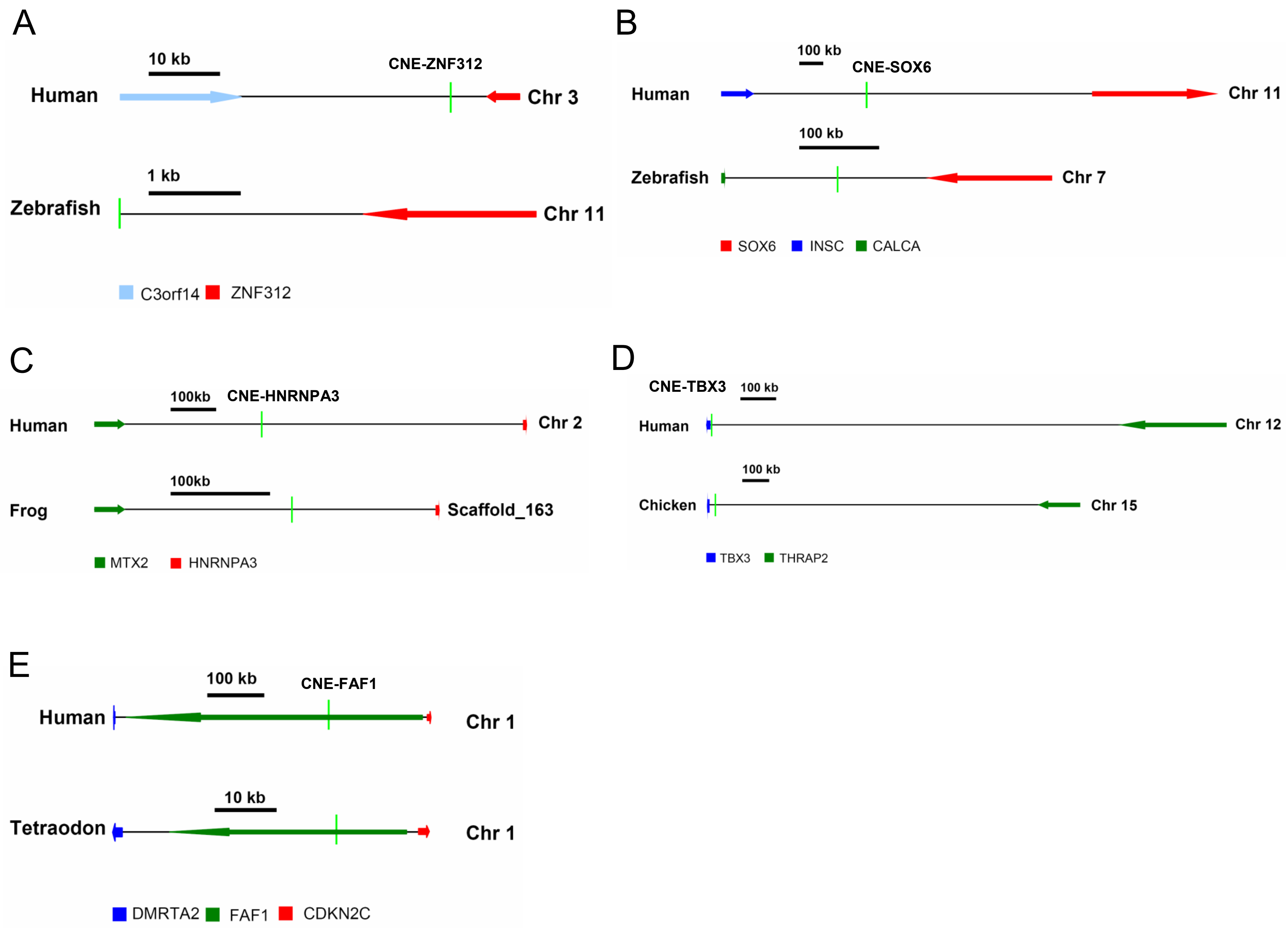
A
10 kb
CNE-ZNF312
Human
Chr 3
1 kb
Zebrafish
Chr 11
C3orf14
ZNF312
B
100 kb
CNE-SOX6
Human
Chr 11
100 kb
Zebrafish
Chr 7
SOX6
INSC
CALCA
C
100kb
CNE-HNRNPA3
Human
Chr 2
100kb
Frog
Scaffold_163
MTX2
HNRNPA3
D
CNE-TBX3
100 kb
Human
Chr 12
100 kb
Chicken
Chr 15
TBX3
THRAP2
E
100 kb
CNE-FAF1
Human
Chr 1
10 kb
Tetraodon
Chr 1
DMRTA2
FAF1
CDKN2C

Figure 12. Target gene identification of human CNE-enhancers through orthology mapping (hs434/hs692/ hs248/hs483/hs194).

Comparing the human fish genic content and their reporter gene expression lead to association of CNE enhancers to their respective genes. **(A)** Both synteny analysis and endogenous expression pattern clearly associate to human *ZNF312* gene. **(B)** A human CNE-enhancer positioned in the intergenic space between human *INSC* and *SOX6* gene is similarly associated with the zebrafish *SOX6* but not with *INSC* strongly suggesting that the human *SOX6* is under the regulatory control of this intergenic enhancer. **(C)** Human-fish conserved linkage and expression analysis associate a human intergenic enhancer with *HNRNPA3* gene. **(D)** The localization of CNE-enhancer in the intergenic space between *TBX3* and *THRAP2* and conservation of this arrangement down to chicken lineage prevents the unambiguous assignment of this CNE-enhancer to one of these genes. However comparing the endogenous expression patterns of both of these flanking genes with the reporter expression pattern induced by this CNE in transgenic mice assay clearly associate this CNE-enhancer to human *TBX3* gene. **(E)** Analyzing the expression pattern and conservation in fish lineage shows that an intragenic CNE-enhancer is regulating the expression of *FAF1*.

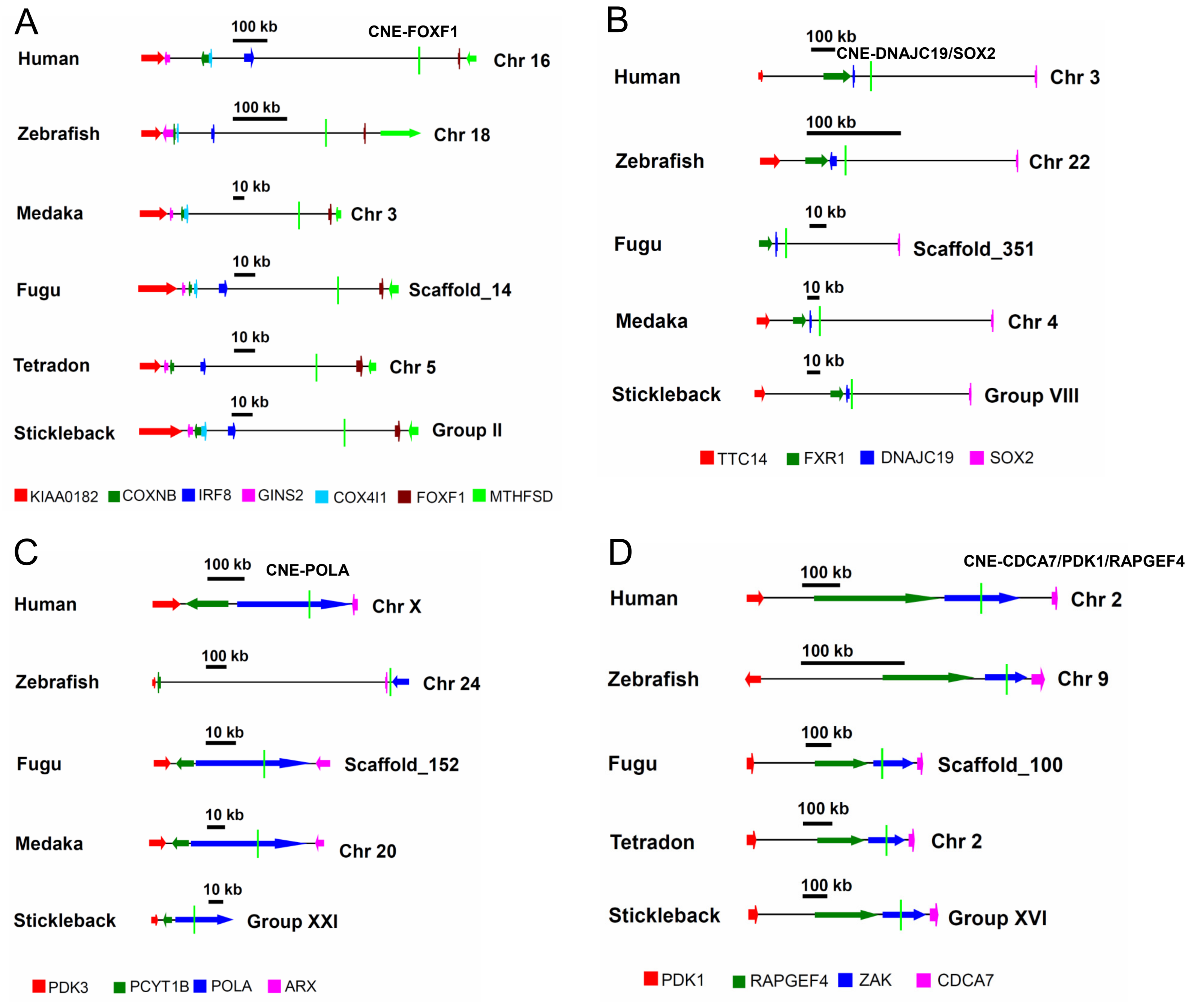

A
100 kb
CNE-FOXF1
Human
Chr 16
100 kb
Zebrafish
Chr 18
10 kb
Medaka
Chr 3
10 kb
Fugu
Scaffold_14
10 kb
Tetradon
Chr 5
10 kb
Stickleback
Group II
KIAA0182 COXNB IRF8 GINS2 COX4I1 FOXF1 MTHFSD
B
100 kb
CNE-DNAJC19/SOX2
Human
Chr 3
100 kb
Zebrafish
Chr 22
10 kb
Fugu
Scaffold_351
10 kb
Medaka
Chr 4
10 kb
Stickleback
Group VIII
TTC14 FXR1 DNAJC19 SOX2
C
100 kb
CNE-POLA
Human
Chr X
100 kb
Zebrafish
Chr 24
10 kb
Fugu
Scaffold_152
10 kb
Medaka
Chr 20
10 kb
Stickleback
Group XXI
PDK3 PCYT1B POLA ARX
D
CNE-CDCA7/PDK1/RAPGEF4
100 kb
Human
Chr 2
100 kb
Zebrafish
Chr 9
100 kb
Fugu
Scaffold_100
100 kb
Tetradon
Chr 2
100 kb
Stickleback
Group XVI
PDK1 RAPGEF4 ZAK CDCA7

Figure 13. Target gene identification of human CNE-enhancers through orthology mapping (hs1/hs192/ hs121/hs242).

Analysis of genic environment of human CNE-enhancers in teleost fish orthologous genomic intervals and observing their expression patterns helps in identifying their target genes. **(A)** The conserved localization of CNE in the intergenic region near *FOXF1* and harmony of their redundant endogenous expression pattern with that of reporter expression induced by this CNE-enhancer in transgenic mice assay suggest the association of this enhancer with *FOXF1* gene. **(B)** A human fish positioning of CNE enhancer between *DNAJC19* and *SOX2* genes and redundancy in the endogenous expression domains of these two genes and CNE induces reporter expression pattern hinders the unambiguous association of this CNE enhancer with either of flanking human genes. **(C)** Human fish comparative analysis of CNE containing locus and comparison of expression domains of CNE enhancer induced reporter expression with endogenous expression pattern of genes present in the human locus suggest that this intragenic human CNE enhancer is associated with regulation of *POLA* genes. **(D)** The conserved physical location of CNE-enhancer within intronic region of *ZAK* in fish and human lineages suggest its association with *ZAK* gene, however the expression studies of neighboring conserved genes infers that this CNE might be regulating the expression of *CDCA7, PDK1* and *RAPGEF4* rather than *ZAK*.

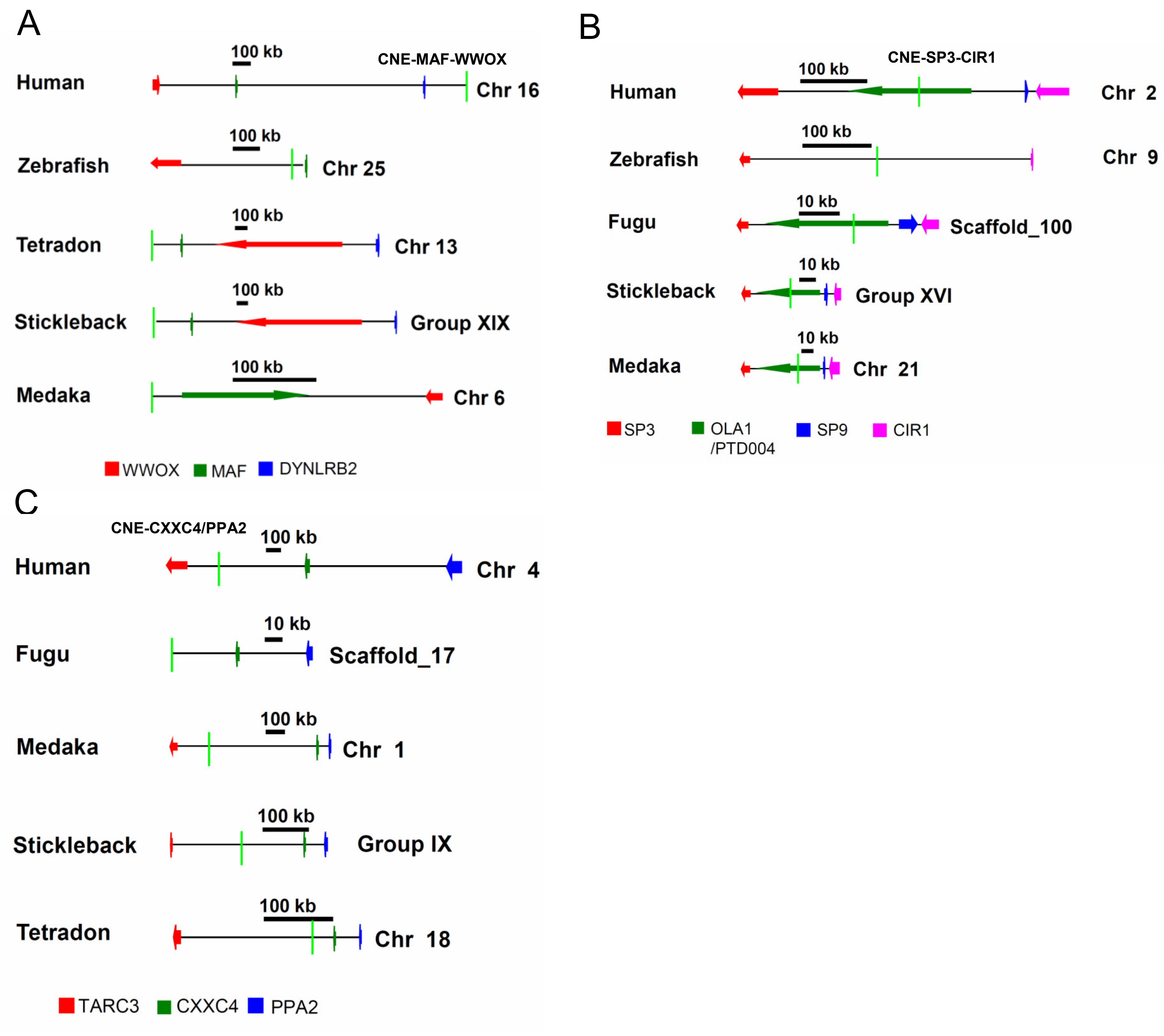
A
100 kb
CNE-MAF-WWOX
Human
Chr 16
Zebrafish
Chr 25
Tetradon
Chr 13
Stickleback
Group XIX
Medaka
Chr 6
WWOX
MAF
DYNLRB2
B
CNE-SP3-CIR1
Human
Chr 2
Zebrafish
Chr 9
10 kb
Fugu
Scaffold_100
Stickleback
Group XVI
Medaka
Chr 21
SP3
OLA1
/PTD004
SP9
CIR1
C
CNE-CXXC4/PPA2
Human
Chr 4
Fugu
Scaffold_17
Medaka
Chr 1
Stickleback
Group IX
Tetradon
Chr 18
TARC3
CXXC4
PPA2

Figure 14. Target gene identification of human CNE-enhancers through orthology mapping (hs4/hs244/ hs260).

Comparative analysis and expression pattern studies help in associating the enhancer to its target gene. **(A)** Comparative analysis of CNE enhancer in fish and human lineages show conserved localization of enhancer along with *MAF* and *WWOX* genes. Moreover comparing the endogenous expression of these genes with reporter gene expression of CNE also suggest the associates this enhancer with *WWOX* or *MAF*. **(B)** The CNE-enhancer is located within intronic region of *PTD004* in human and some fish lineages. The comparative syntenic analysis of human locus with multiple fish lineages suggest its association with *SP3* or *CIR* ,moreover expression pattern studies also favors this association. **(C)** Comparative syntenic analysis of CNE enhancer containing region suggest the association of CNE with either *CXXC4* or *PPA2*.

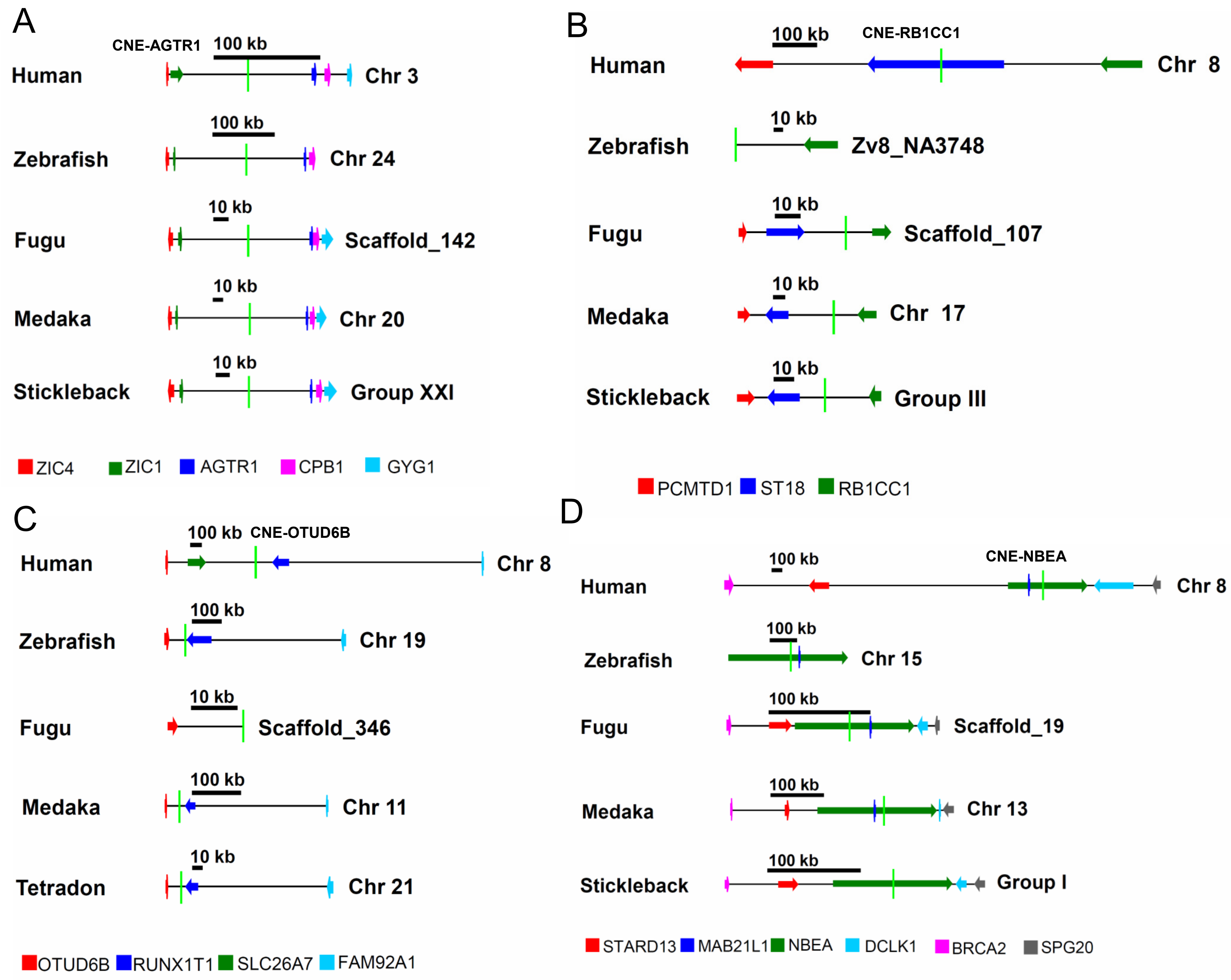
A
CNE-AGTR1
100 kb
Human
Chr 3
Zebrafish
Chr 24
10 kb
Fugu
Scaffold_142
Medaka
Chr 20
Stickleback
Group XXI
ZIC4
ZIC1
AGTR1
CPB1
GYG1
B
CNE-RB1CC1
Human
Chr 8
Zebrafish
Zv8_NA3748
Fugu
Scaffold_107
Medaka
Chr 17
Stickleback
Group III
PCMTD1
ST18
RB1CC1
C
CNE-OTUD6B
Human
Chr 8
Zebrafish
Chr 19
Fugu
Scaffold_346
Medaka
Chr 11
Tetradon
Chr 21
OTUD6B
RUNX1T1
SLC26A7
FAM92A1
D
CNE-NBEA
Human
Chr 8
Zebrafish
Chr 15
Fugu
Scaffold_19
Medaka
Chr 13
Stickleback
Group I
STARD13
MAB21L1
NBEA
DCLK1
BRCA2
SPG20

Figure 15. Target gene identification of human CNE-enhancers through orthology mapping (hs654/hs698/hs669/hs1330).

Comparative analysis and expression pattern studies help in finding the target gene for CNE enhancer. **(A)** The CNE-enhancer is located in the intergenic space between *ZIC1* and *AGTR1* and this positioning is conserved in human-fish comparison, however comparing the endogenous expression pattern of flanking genes with reporter expression induces by this CNE enhancer suggest that this CNE enhancer might be associated with regulation of human *AGTR1* gene. **(B)** Human CNE enhancer is located in the intronic region of *ST18* on chromosome 8. Comparative analysis of this locus in fish lineage clearly link this CNE enhancer with the regulation of *RB1CC1* gene moreover endogenous expression pattern of *RB1CC1* also suggest it as a target gene for this enhancer. **(C)** Human fish comparative analysis of CNE enhancer containing region shows conservation of CNE near *OTUD6B* suggesting it to be the target gene for this CNE. **(D)** The conserved localization of CNE-enhancer within intronic region of *NBEA* in human and all fish lineages, and its redundant expression in accordance with CNE enhancer induced reporter gene expression unmistakably links this CNE enhancer with the regulation of *NBEA* gene.

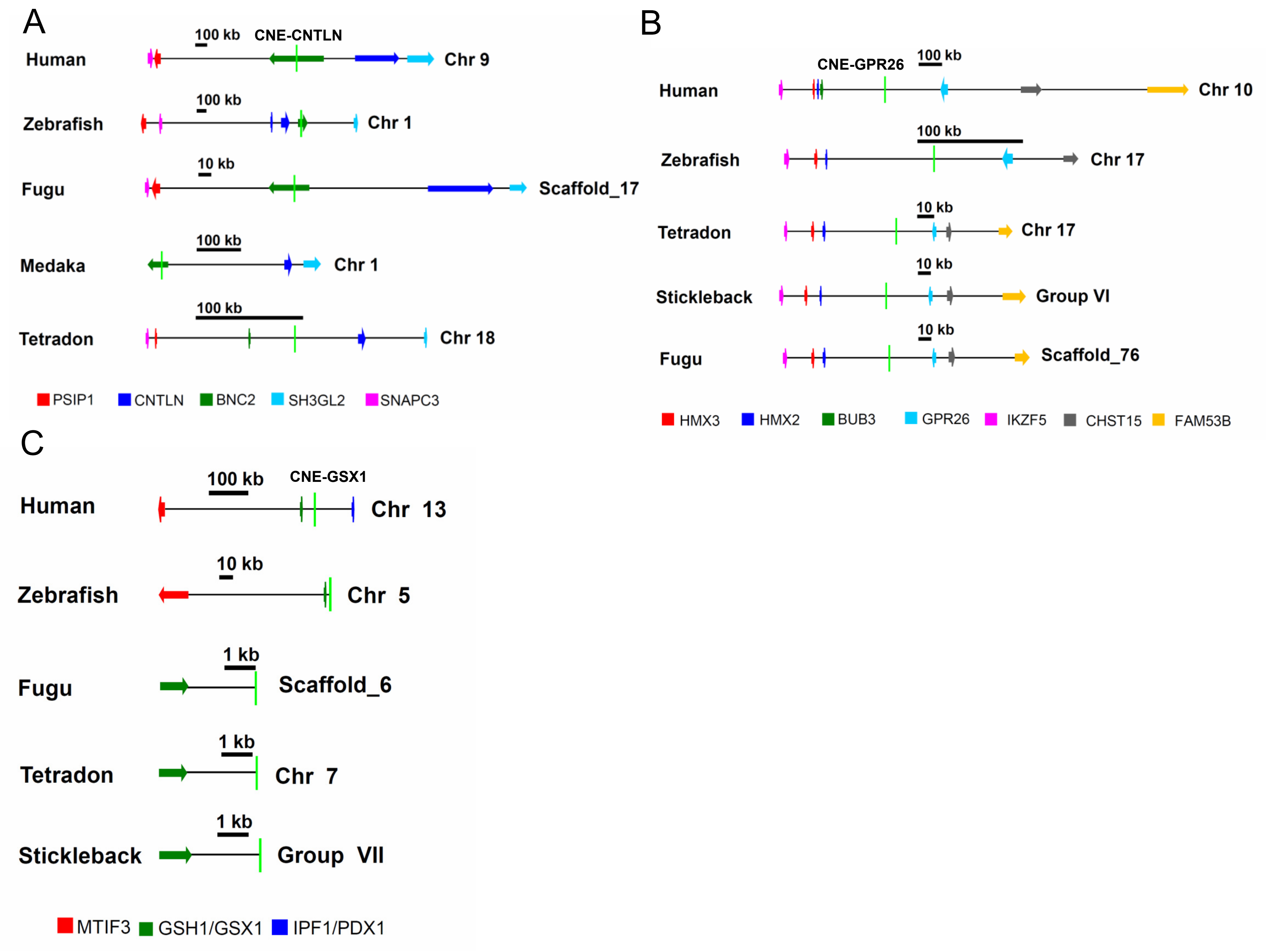
A
100 kb
CNE-CNTLN
Human
Chr 9
100 kb
Zebrafish
Chr 1
10 kb
Fugu
Scaffold_17
100 kb
Medaka
Chr 1
100 kb
Tetradon
Chr 18
PSIP1
CNTLN
BNC2
SH3GL2
SNAPC3
B
CNE-GPR26
100 kb
Human
Chr 10
100 kb
Zebrafish
Chr 17
10 kb
Tetradon
Chr 17
10 kb
Stickleback
Group VI
10 kb
Fugu
Scaffold_76
HMX3
HMX2
BUB3
GPR26
IKZF5
CHST15
FAM53B
C
100 kb
CNE-GSX1
Human
Chr 13
10 kb
Zebrafish
Chr 5
1 kb
Fugu
Scaffold_6
1 kb
Tetradon
Chr 7
1 kb
Stickleback
Group VII
MTIF3
GSH1/GSX1
IPF1/PDX1

Figure 16. Target gene identification of human CNE-enhancers through orthology mapping (hs307/hs578/ Hs532).

(A) The conserved positioning of CNE-enhancer within intronic region of *BNC2* in human and some fish lineages suggest its association with *BCN2* whereas human fish comparative syntenic analysis and expression pattern studies favors its association with *CNTLN* rather than *BNC2*. **(B)** Human CNE enhancer is located in the intergenic space between *BUB3* and *GPR26*.Comparative syntenic analysis of the human locus with orthologous region of this element in teleost fish lineage reveals the association of this CNE with *GPR26*, also the expression pattern of *GPR26* is in accordance with that of reporter gene expression leading to the unambiguous association of *GPR26* as the target gene for this CNE. **(C)** Conserved positioning of the enhancer near *GSH1* gene in human and fish lineages along with endogenous expression of *GSH1* in accordance with CNE induced reporter gene expression clearly suggests that this enhancer is associated with regulation of *GSH1* gene.

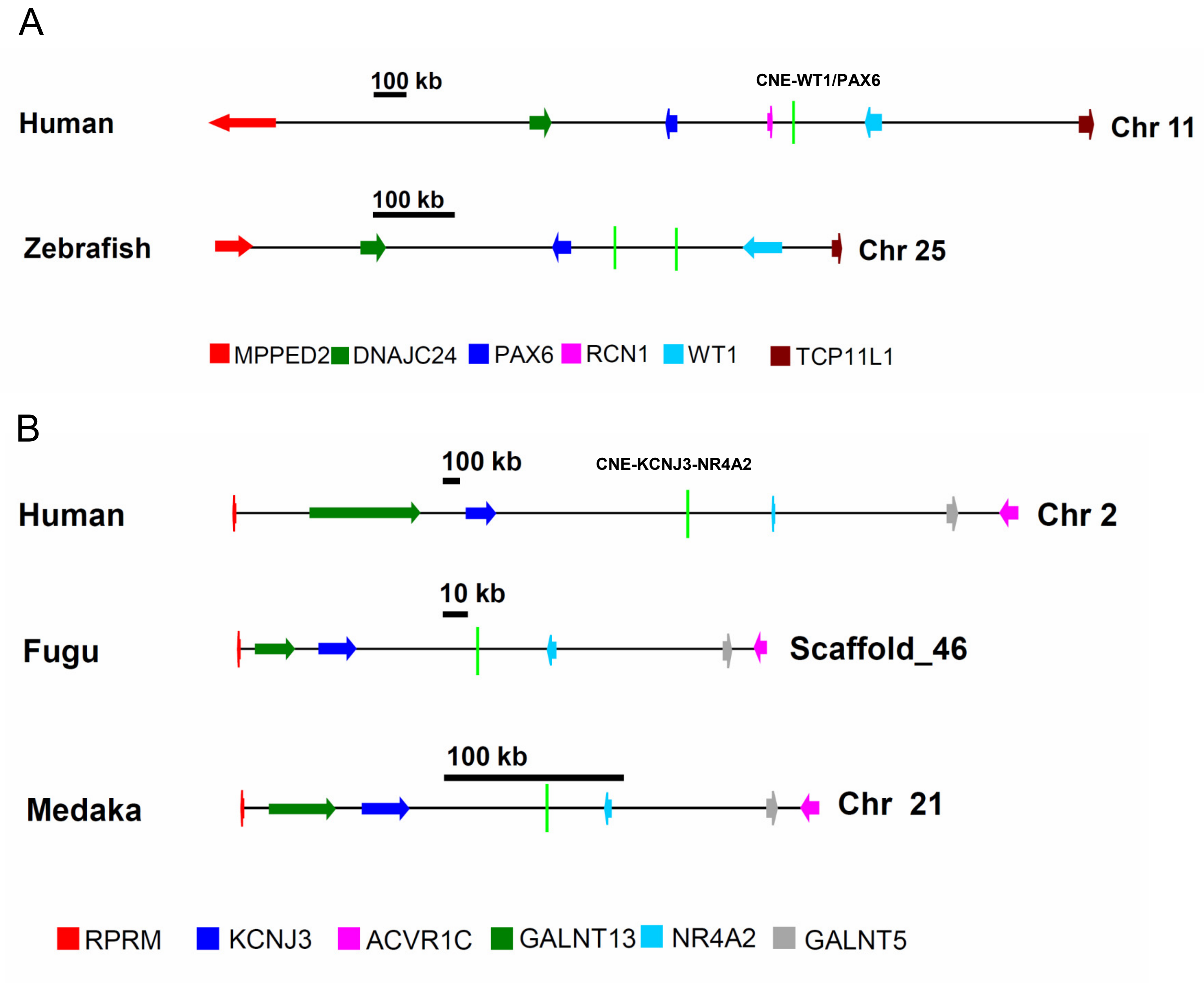

A
100 kb
CNE-WT1/PAX6
Human
Chr 11
100 kb
Zebrafish
Chr 25
MPPED2
DNAJC24
PAX6
RCN1
WT1
TCP11L1
B
100 kb
CNE-KCNJ3-NR4A2
Human
Chr 2
10 kb
Fugu
Scaffold_46
100 kb
Medaka
Chr 21
RPRM
KCNJ3
ACVR1C
GALNT13
NR4A2
GALNT5

Figure 17. Target gene identification of human CNE-enhancers through orthology mapping (hs113 /hs411).

Analysis of CNE-enhancers in humans and its orthologous genomic intervals teleost fish lineage helps in associating CNE to their target genes. **(A)** CNE-enhancer is positioned within an intergenic space between human *RCN1* and *WT1* on chromosome 11 .Comparative syntenic analysis in fish lineage and expression pattern studies suggest the association of this CNE with *PAX6* or *WT1*. **(B)** Conserved localization of CNE-enhancer in the intergenic space between human *KCNJ3* and *NR4A2* and all other fish lineages and expression pattern of these genes in accordance with CNE enhancer suggest that this CNE is associated with either of flanking genes.

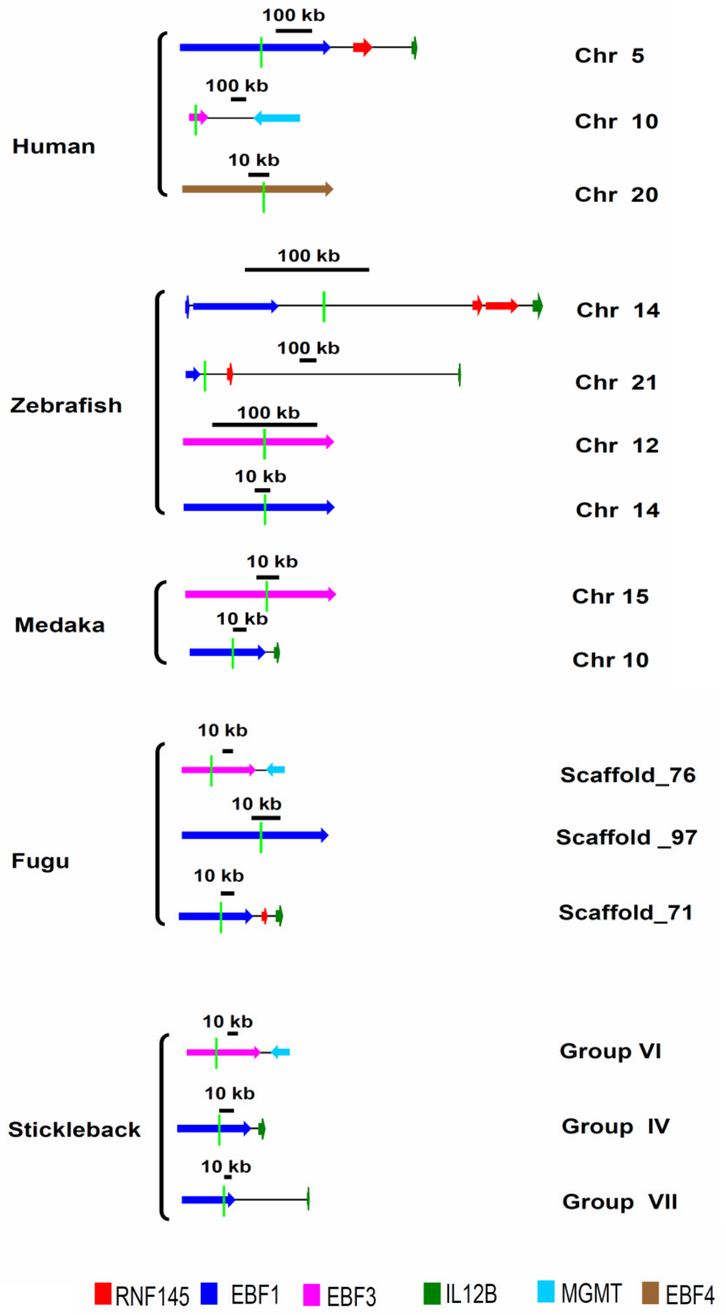

Human
100 kb
Chr 5
100 kb
Chr 10
10 kb
Chr 20
Zebrafish
100 kb
Chr 14
100 kb
Chr 21
100 kb
Chr 12
10 kb
Chr 14
Medaka
10 kb
Chr 15
10 kb
Chr 10
Fugu
10 kb
Scaffold_76
10 kb
Scaffold _97
10 kb
Scaffold_71
Stickleback
10 kb
Group VI
10 kb
Group IV
10 kb
Group VII
RNF145
EBF1
EBF3
IL12B
MGMT
EBF4

Figure 18. Target gene identification of human CNE-enhancers through orthology mapping (hs230).

Human enhancer target gene duplicated early in vertebrate history before mammal fish divergence. Comparing the genic content of *CNE-EBF* paralogous loci in human genome and their orthologous loci in multiple fish lineages clearly suggests that duplicated copies of human *CNE-EBF* enhancer are associated with the regulation of paralogous copies of *EBF* family members i.e.; *EBF1, EBF3* and *EBF4*. The examination of fish loci also suggest the lineage specific duplication of *CNE-EBF* enhancer in teleost fish.

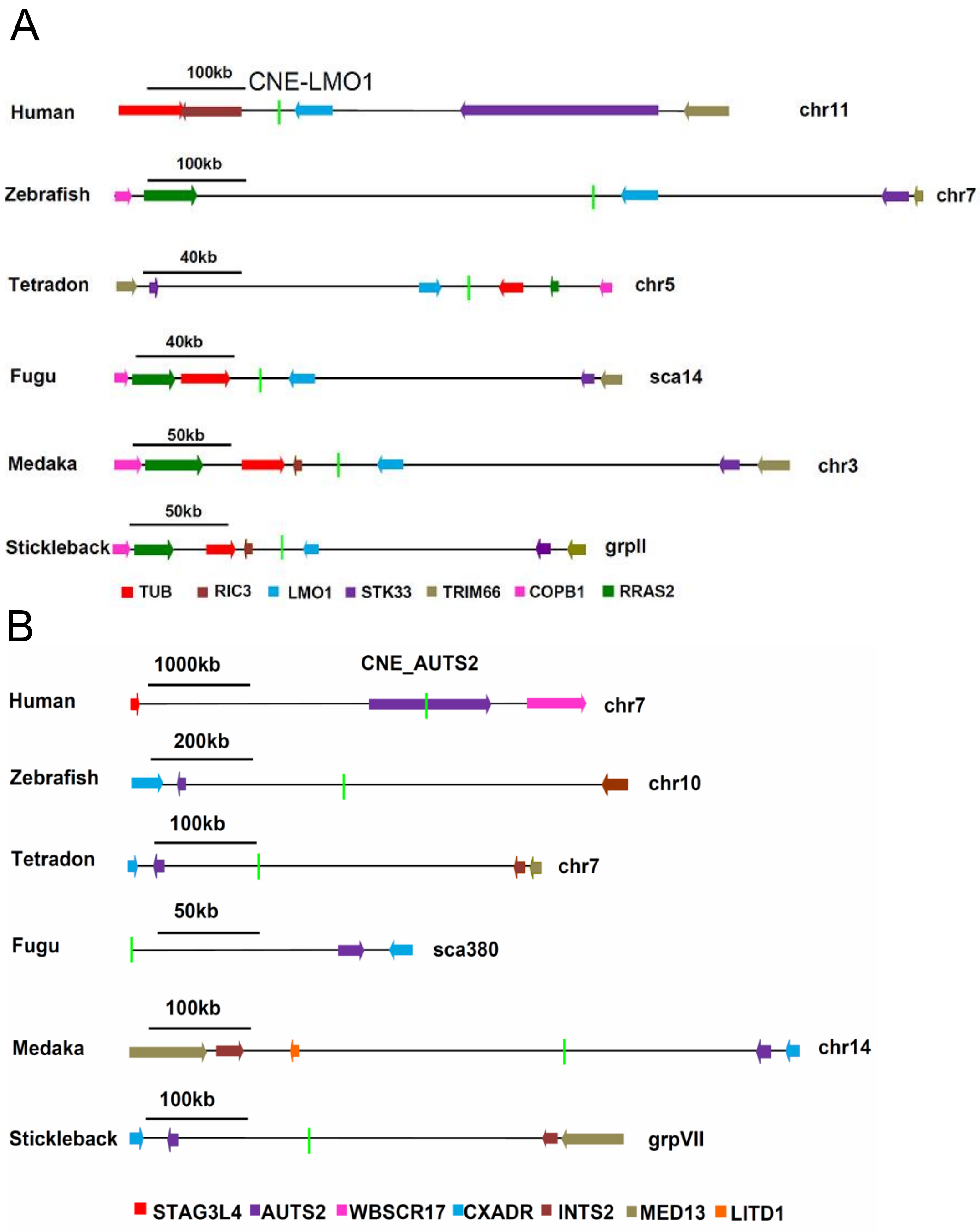
A
100kb
CNE-LMO1
Human
chr11
Zebrafish
100kb
chr7
Tetradon
40kb
chr5
Fugu
40kb
sca14
Medaka
50kb
chr3
Stickleback
50kb
grpII
TUB
RIC3
LMO1
STK33
TRIM66
COPB1
RRAS2
B
1000kb
CNE_AUTS2
Human
chr7
Zebrafish
200kb
chr10
Tetradon
100kb
chr7
Fugu
50kb
sca380
Medaka
100kb
chr14
Stickleback
100kb
grpVII
STAG3L4
AUTS2
WBSCR17
CXADR
INTS2
MED13
LITD1

Figure 19. Analysis of genic environment of human CNE-enhancers in teleost fish orthologous genomic intervals helps in identifying their target genes (hs1859/hs1425).

Systematic analysis of the surrounding region of CNE enhancers, conserved across the tetrapod-teleost lineages, carved a pathway to associate the CNE enhancer to its target genes. **(A)** Comparative syntenic analysis of human and teleost fishes and the expression pattern analysis clearly depicts the connection between the CNE and *LMO1*, because of their conservation throughout the verteberate genome. **(B)** Analyzing the expression pattern and conservation in tetrapod and fish lineages show that an intragenic CNE-enhancer is regulating the expression of *AUTS2*.

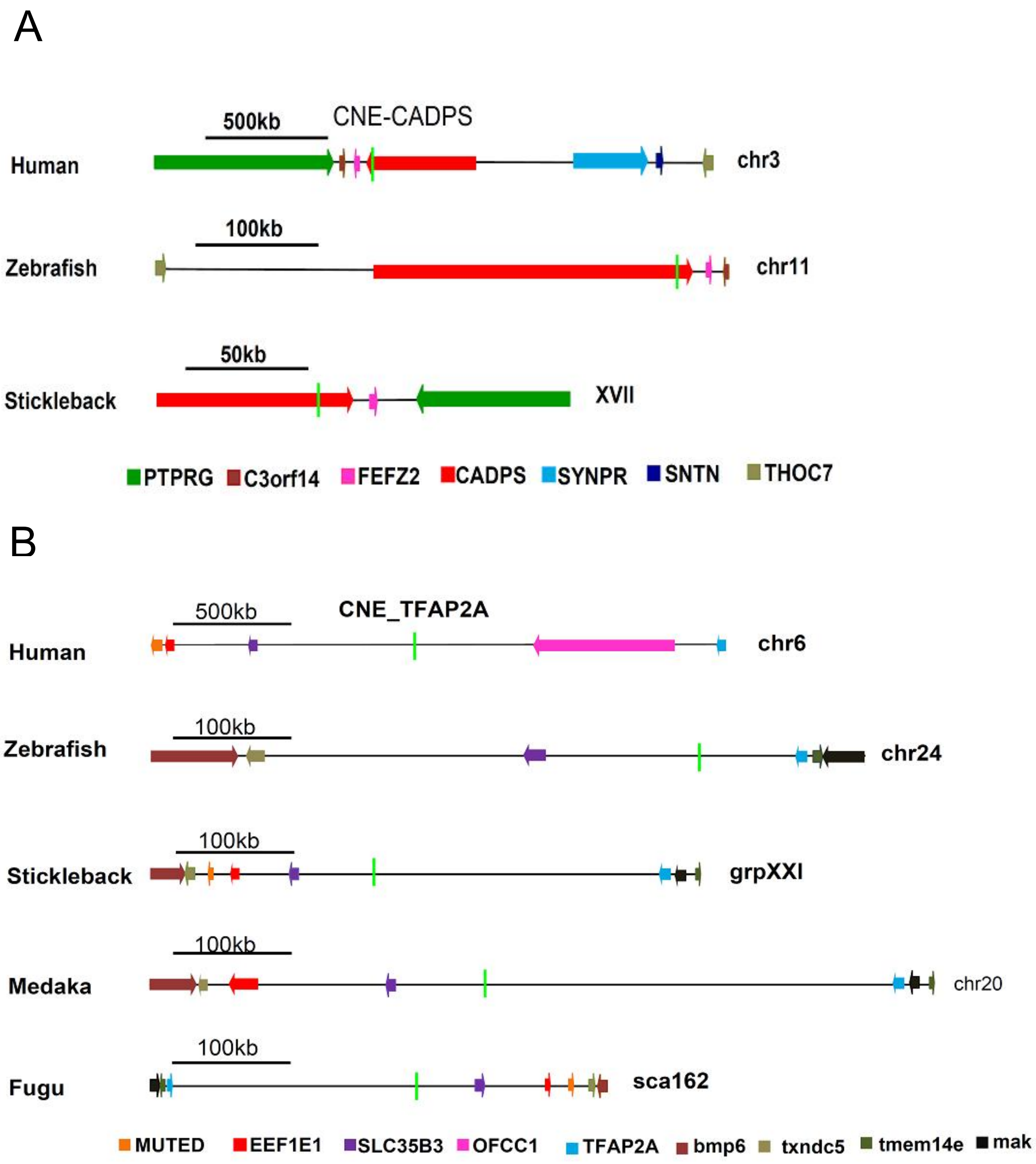

A
500kb
CNE-CADPS
Human
chr3
100kb
Zebrafish
chr11
50kb
Stickleback
XVII
PTPRG
C3orf14
FEFZ2
CADPS
SYNPR
SNTN
THOC7
B
500kb
CNE_TFAP2A
Human
chr6
100kb
Zebrafish
chr24
100kb
Stickleback
grpXXI
100kb
Medaka
chr20
100kb
Fugu
sca162
MUTED
EEF1E1
SLC35B3
OFCC1
TFAP2A
bmp6
txndc5
tmem14e
mak

Figure 20. Assigning the target genes to CNE-enhancers through comparative genomic analysis (hs1316/ hs1315).

Through Comparative genomic analysis of functionally defined CNE enhancers, across tetrapod-teleost lineages, cis regulatory networks of human genes are defined. **(A)** Not only the CNE but also its intronic location in *CADPS*, conserved in both human and teleost lineages, suggests the link exists between CNE and *CADPS* which is further confirmed by endogenous expression pattern analysis of both the CNE and the gene. **(B)** Studying the orthologous genomic content in the neighboring region of CNE enhancer in tetrapod-teleost lineages demonstrate the relationship between CNE and its target gene *TFAP2A*.

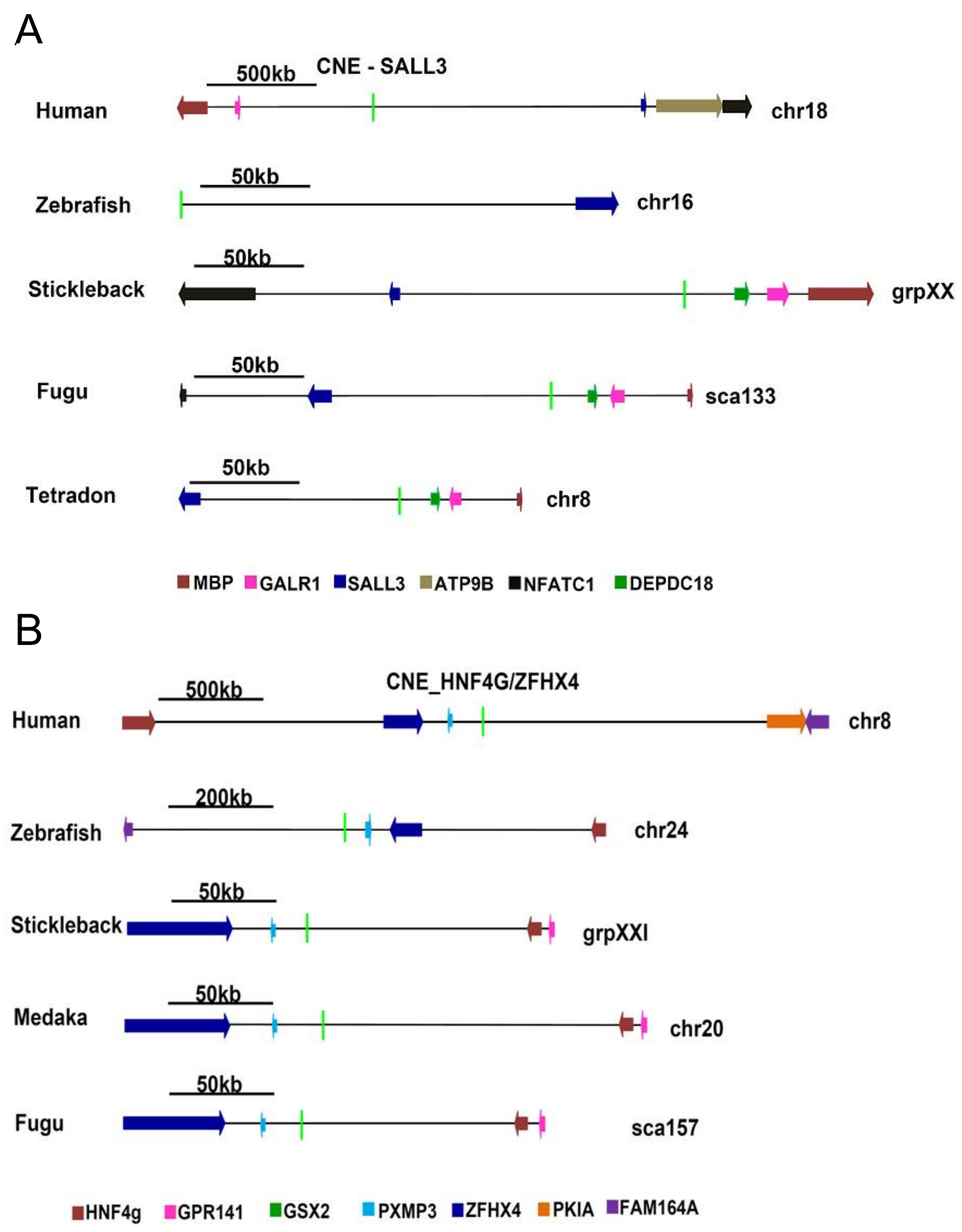
A
500kb
CNE - SALL3
Human
chr18
50kb
Zebrafish
chr16
50kb
Stickleback
grpXX
50kb
Fugu
sca133
50kb
Tetradon
chr8
MBP
GALR1
SALL3
ATP9B
NFATC1
DEPDC18
B
500kb
CNE_HNF4G/ZFHX4
Human
chr8
200kb
Zebrafish
chr24
50kb
Stickleback
grpXXI
50kb
Medaka
chr20
50kb
Fugu
sca157
HNF4g
GPR141
GSX2
PXMP3
ZFHX4
PKIA
FAM164A

Figure 21. Comparing the human-fish genic content and their reporter gene expression lead to association of CNE enhancers to their respective genes (hs1186/hs1318).

Target genes of CNE enhancers are identified through orthology mapping in Human and teleost fishes. **(A)** By tracing the genomic content around the CNE in tetrapod and fish lineages it becomes evident that the CNE is regulatory element of *SALL3*. Redundant expression pattern of the gene and CNE is also in accordance with each other. **(B)** Conservation of *ZFHX4* and *HNF4G* through out the vertebrate genome and endogenous expression pattern analysis of CNE matches with both these genes proposes them as target genes of CNE.

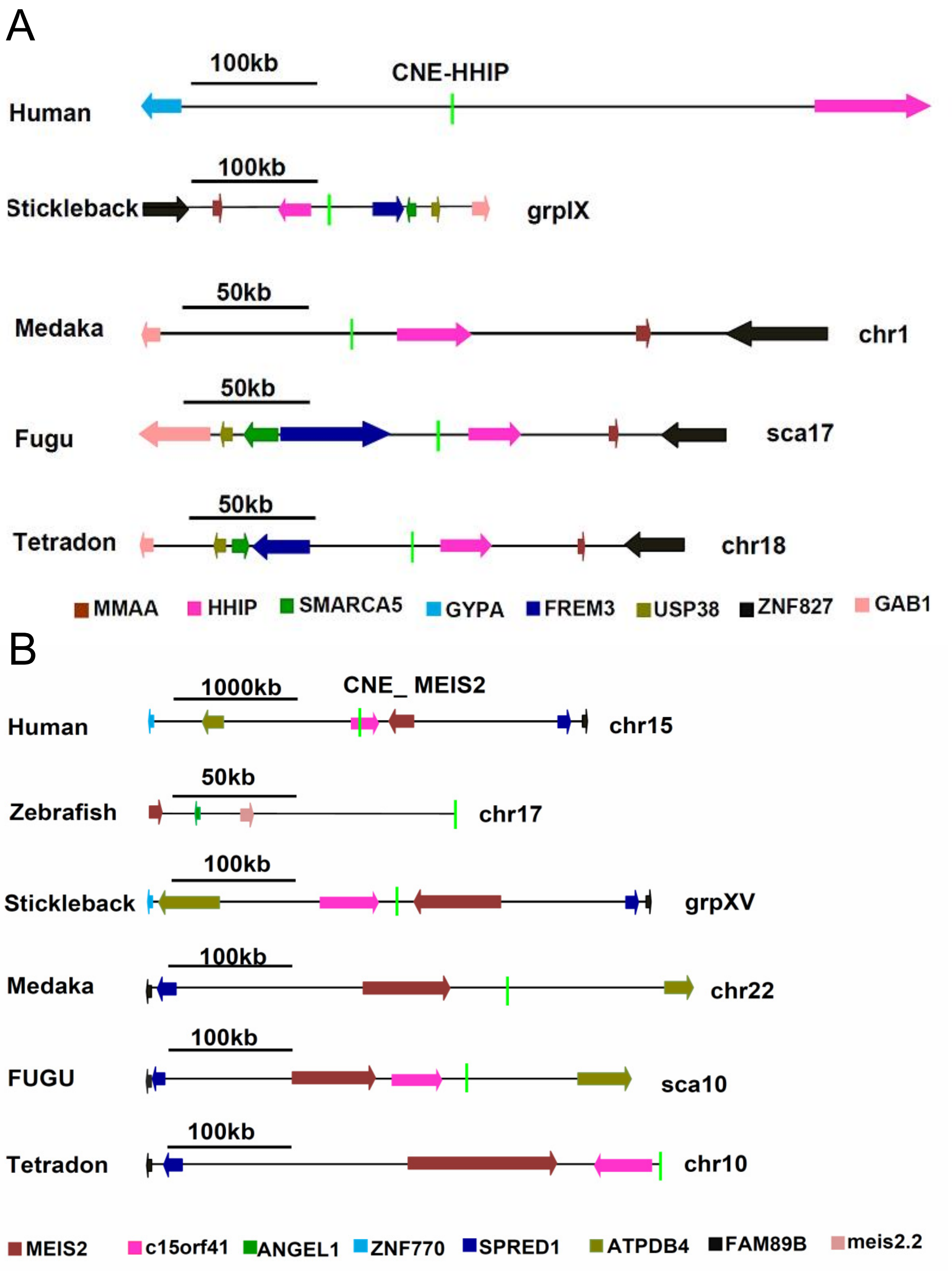
A
100kb
CNE-HHIP
Human
chr4
100kb
Stickleback
grpIX
50kb
Medaka
chr1
50kb
Fugu
sca17
50kb
Tetradon
chr18
MMAA
HHIP
SMARCA5
GYPA
FREM3
USP38
ZNF827
GAB1
B
1000kb
CNE_ MEIS2
Human
chr15
50kb
Zebrafish
chr17
100kb
Stickleback
grpXV
100kb
Medaka
chr22
100kb
FUGU
sca10
100kb
Tetradon
chr10
MEIS2
c15orf41
ANGEL1
ZNF770
SPRED1
ATPDB4
FAM89B
meis2.2

Figure 22. Analysis of genic environment of human CNE-enhancers in teleost fish orthologous genomic intervals and observing their expression patterns helps in identifying their target genes (hs906/hs828).

Gene, controlled by specific regulatory elements, are identified through the systematic analysis of orthologous genomic content, which harbors functionally identified CNE enhancers, of tetrapod-teleost lineages. **(A)** Conserved localization of CNE and *HHIP* in tetrapod-teleost lineages demonstrate the linkage between them. Redundant expression pattern analysis of CNE and *HHIP* also emphasizes this idea. **(B)** Genic architecture analysis of the orthologous loci holding the CNE enhancers in vertebrate genome recommends the association of CNE with *MEIS2.*

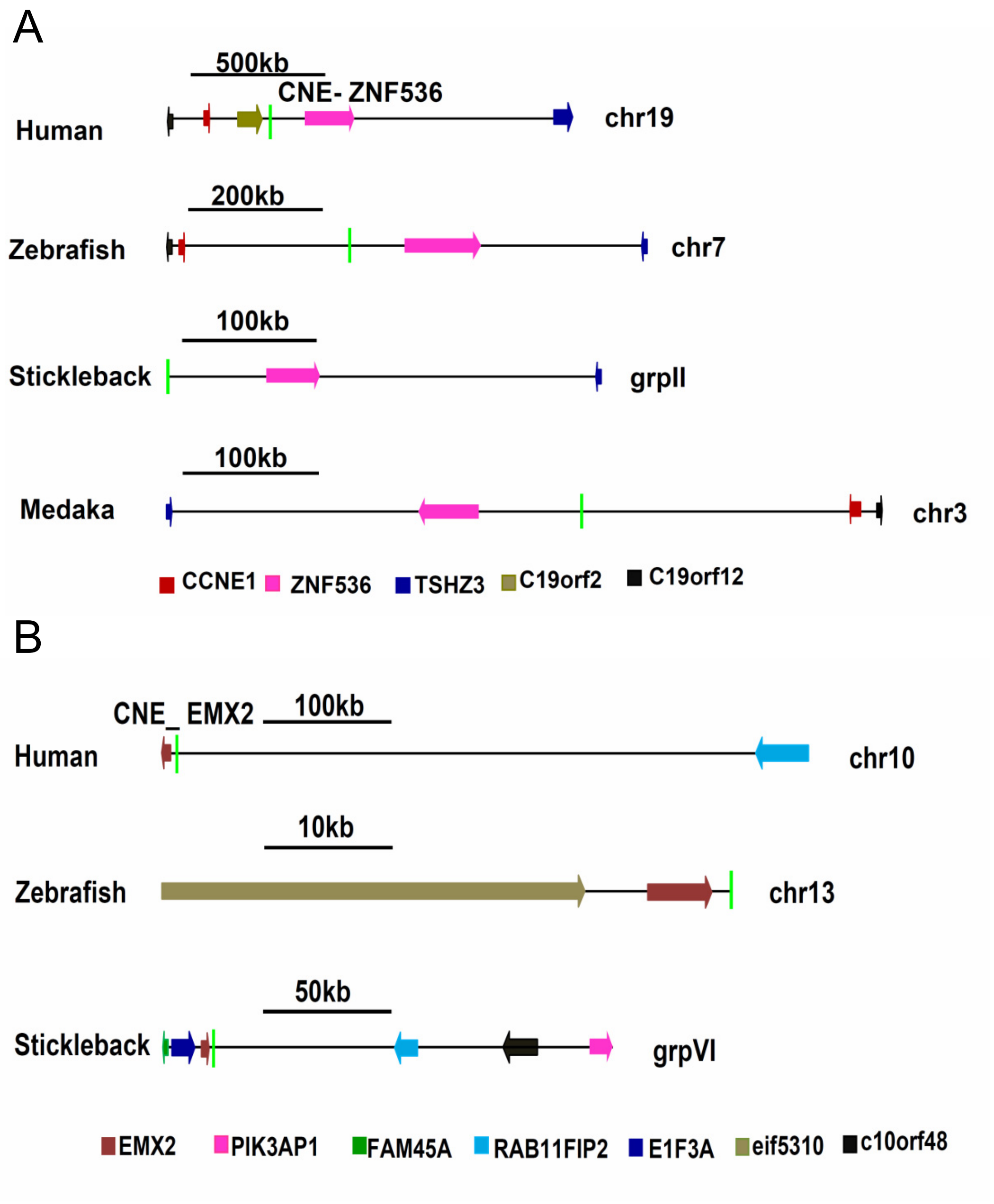
A
500kb
CNE- ZNF536
Human
chr19
200kb
Zebrafish
chr7
100kb
Stickleback
grpII
100kb
Medaka
chr3
CCNE1
ZNF536
TSHZ3
C19orf2
C19orf12
B
CNE_ EMX2
100kb
Human
chr10
10kb
Zebrafish
chr13
50kb
Stickleback
grpVI
EMX2
PIK3AP1
FAM45A
RAB11FIP2
E1F3A
eif5310
c10orf48

Figure 23. Analysis of genic environment of human CNE-enhancers in teleost fish orthologous genomic intervals helps in identifying their target genes (hs858/hs935).

Analysis of CNE-enhancers in humans and its orthologous genomic intervals teleost fish lineage helps in associating CNE to their target genes. **(A)** Both synteny analysis and endogenous expression pattern clearly associate the CNE to human *ZNF536* gene. **(B)** CNE residing between *EMX2* and *RAB11FIP2* in humans is also conserved along with *EMX2,* with differential loss and gain of bystander genes, in teleost fishes. Comparison of reporter gene expression of CNE enhancer and *EMX2* also confers *EMX2* as target gene of CNE.

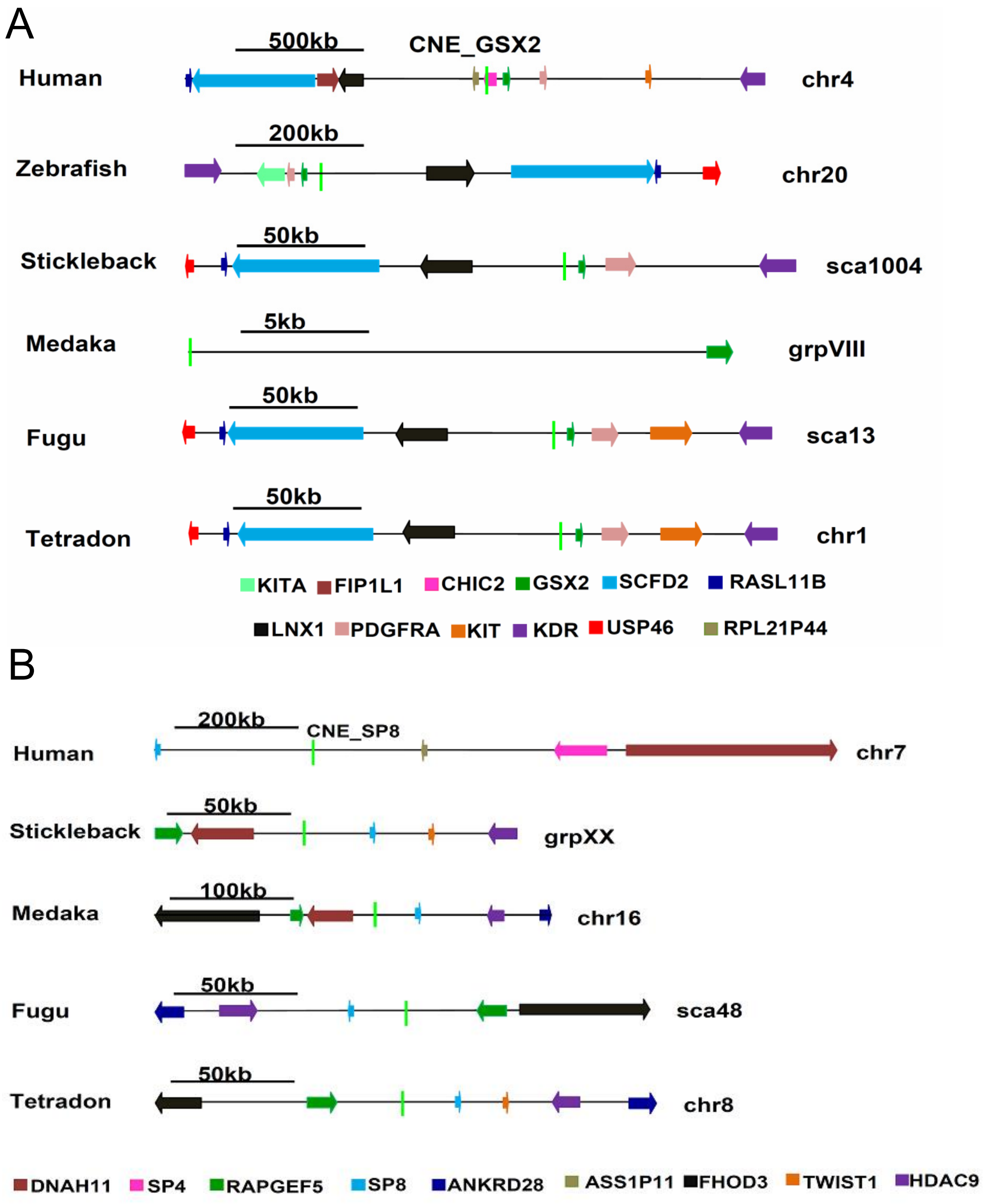

A
500kb
CNE_GSX2
Human
chr4
200kb
Zebrafish
chr20
50kb
Stickleback
sca1004
5kb
Medaka
grpVIII
50kb
Fugu
sca13
50kb
Tetradon
chr1
KITA FIP1L1 CHIC2 GSX2 SCFD2 RASL11B
LNX1 PDGFRA KIT KDR USP46 RPL21P44
B
200kb
CNE_SP8
Human
chr7
50kb
Stickleback
grpXX
100kb
Medaka
chr16
50kb
Fugu
sca48
50kb
Tetradon
chr8
DNAH11 SP4 RAPGEF5 SP8 ANKRD28 ASS1P11 FHOD3 TWIST1 HDAC9

Figure 24. Assigning the target genes to CNE-enhancers through comparative genomic analysis (hs687/ hs701).

Systematic analysis of the surrounding region of CNE enhancers, conserved across the tetrapod-teleost lineages, carved a pathway to associate the CNE enhancer to its target genes. **(A)** Comparative syntenic analysis of human and teleost fishes and the expression pattern analysis clearly depicts the connection between the CNE and *GSX2,* because of their conservation throughout the vertebrate genome. **(B)** Analyses of expression pattern and conservation in tetrapod and fish lineages shows that CNE-enhancer is regulating the expression of *SP8*.

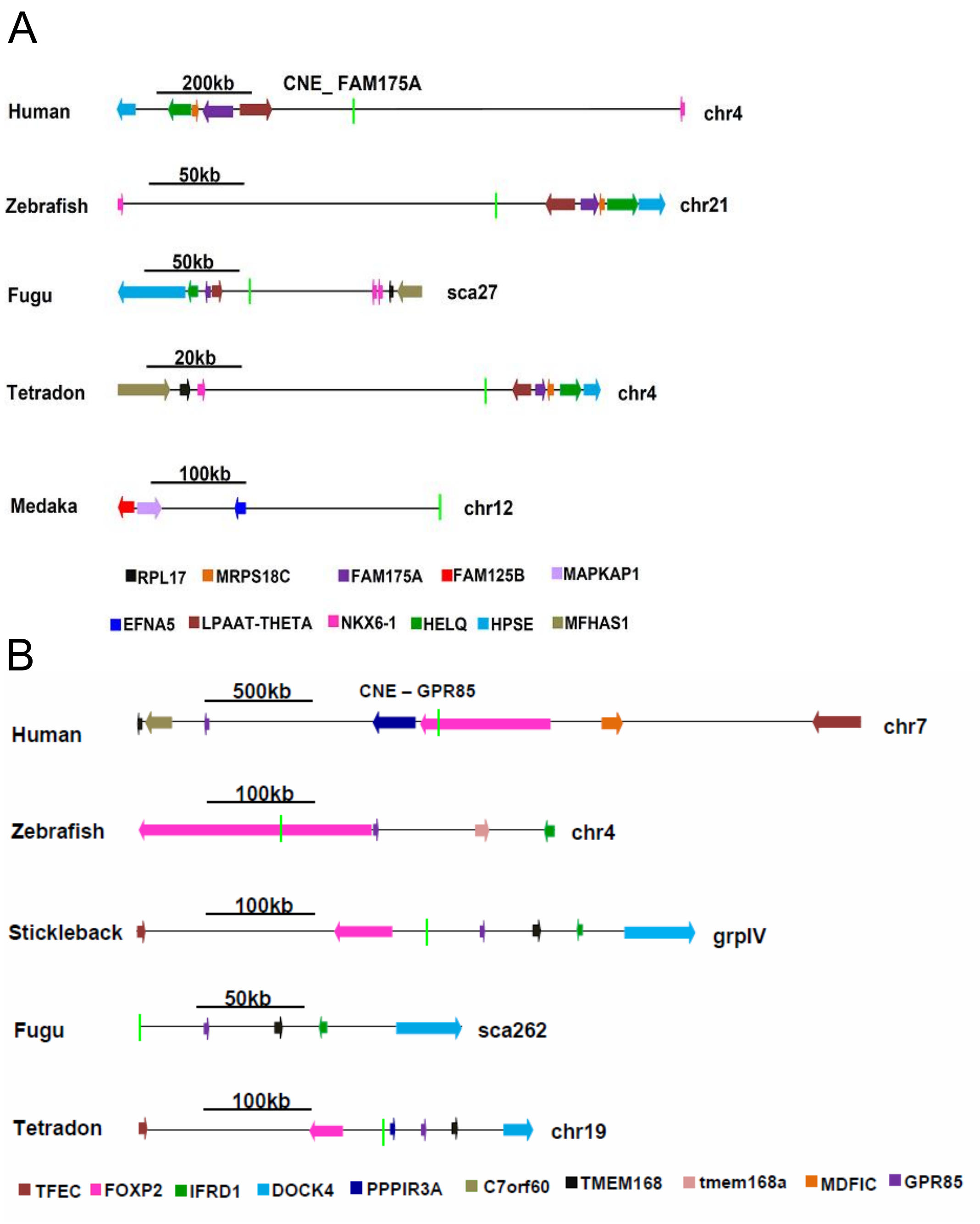

A
200kb
CNE_ FAM175A
Human
chr4
50kb
Zebrafish
chr21
50kb
Fugu
sca27
20kb
Tetradon
chr4
100kb
Medaka
chr12
RPL17
MRPS18C
FAM175A
FAM125B
MAPKAP1
EFNA5
LPAAT-THETA
NKX6-1
HELQ
HPSE
MFHAS1
B
500kb
CNE – GPR85
Human
chr7
100kb
Zebrafish
chr4
100kb
Stickleback
grpIV
50kb
Fugu
sca262
100kb
Tetradon
chr19
TFEC
FOXP2
IFRD1
DOCK4
PPPIR3A
C7orf60
TMEM168
tmem168a
MDFIC
GPR85

Figure 25. Analysis of genic environment of human CNE-enhancers in teleost fish orthologous genomic intervals and observing their expression patterns helps in identifying their target genes (hs712/ hs720).

Through Comparative genomic analysis of functionally defined CNE enhancers, across tetrapod and fish lineages, cis regulatory networks of human genes are defined. **(A)** Conserved localization of CNE and *FAM175A* in tetrapod-teleost lineages demonstrate the linkage between them. Redundant expression pattern analysis of CNE and *FAM175A* also emphasizes this idea. **(B)** Studying the orthologous genomic content in the neighboring region of CNE enhancer in human and teleost lineages demonstrate the relationship between CNE and its target gene *GPR85*.

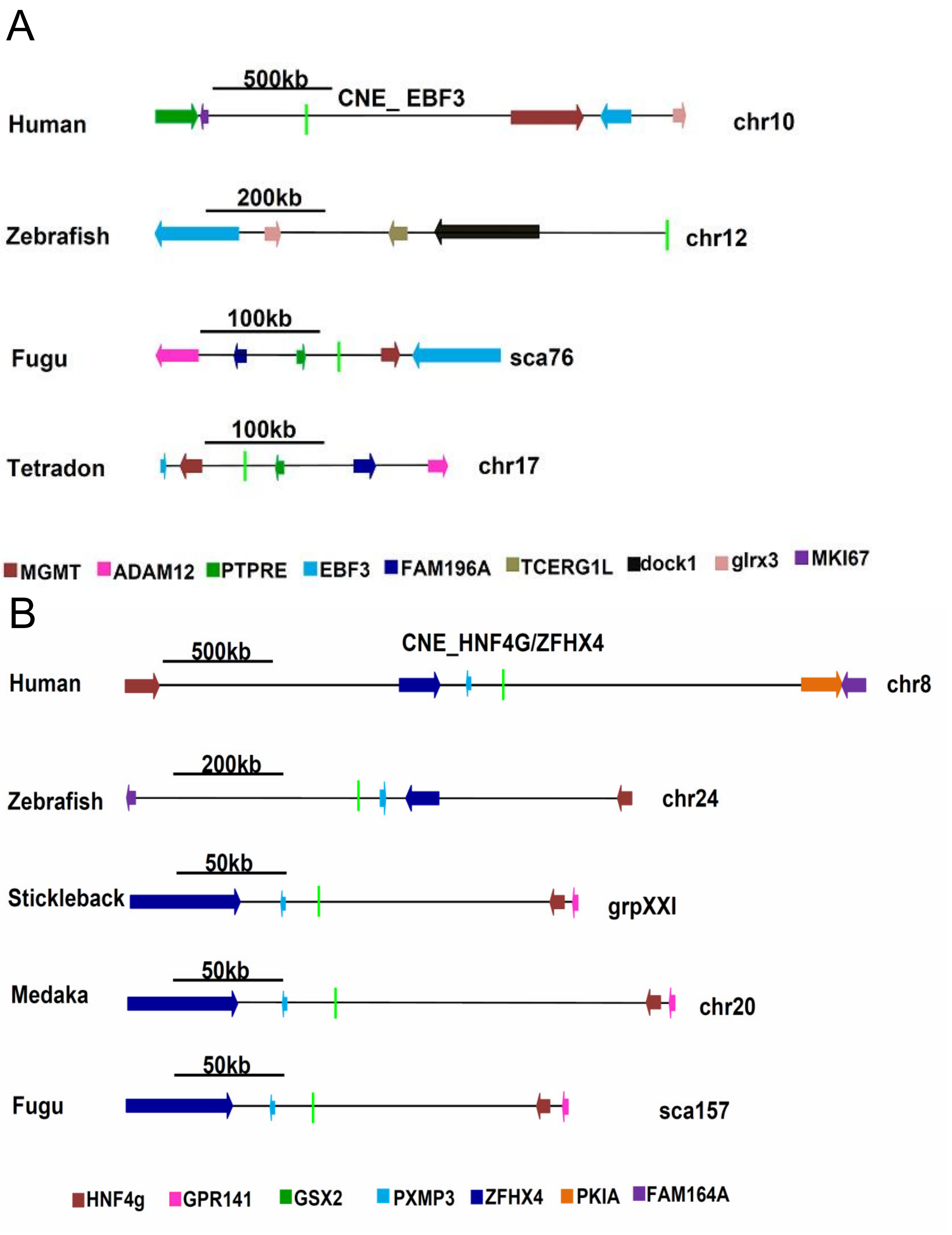
A
500kb
CNE_ EBF3
Human
chr10
200kb
Zebrafish
chr12
100kb
Fugu
sca76
100kb
Tetradon
chr17
MGMT
ADAM12
PTPRE
EBF3
FAM196A
TCERG1L
dock1
glrx3
MKI67
B
500kb
CNE_HNF4G/ZFHX4
Human
chr8
200kb
Zebrafish
chr24
50kb
Stickleback
grpXXI
50kb
Medaka
chr20
50kb
Fugu
sca157
HNF4g
GPR141
GSX2
PXMP3
ZFHX4
PKIA
FAM164A

Figure 26. **Analysis of genic environment of human CNE-enhancers in teleost fish orthologous genomic intervals helps in identifying their target genes (hs737/hs742).**

Target genes of CNE enhancers are identified through orthology mapping in Human and teleost fishes. **(A)** By tracing the genomic content around the CNE in tetrapod and fish lineages it becomes evident that the CNE is regulatory element of *EBF3*. Redundant expression pattern of the gene and CNE is also in accordance with each other. **(B)** Conservation of *ZFHX4* and *HNF4G* through out the vertebrate genome and endogenous expression pattern analysis of CNE matches with both these genes proposes them as target genes of CNE.

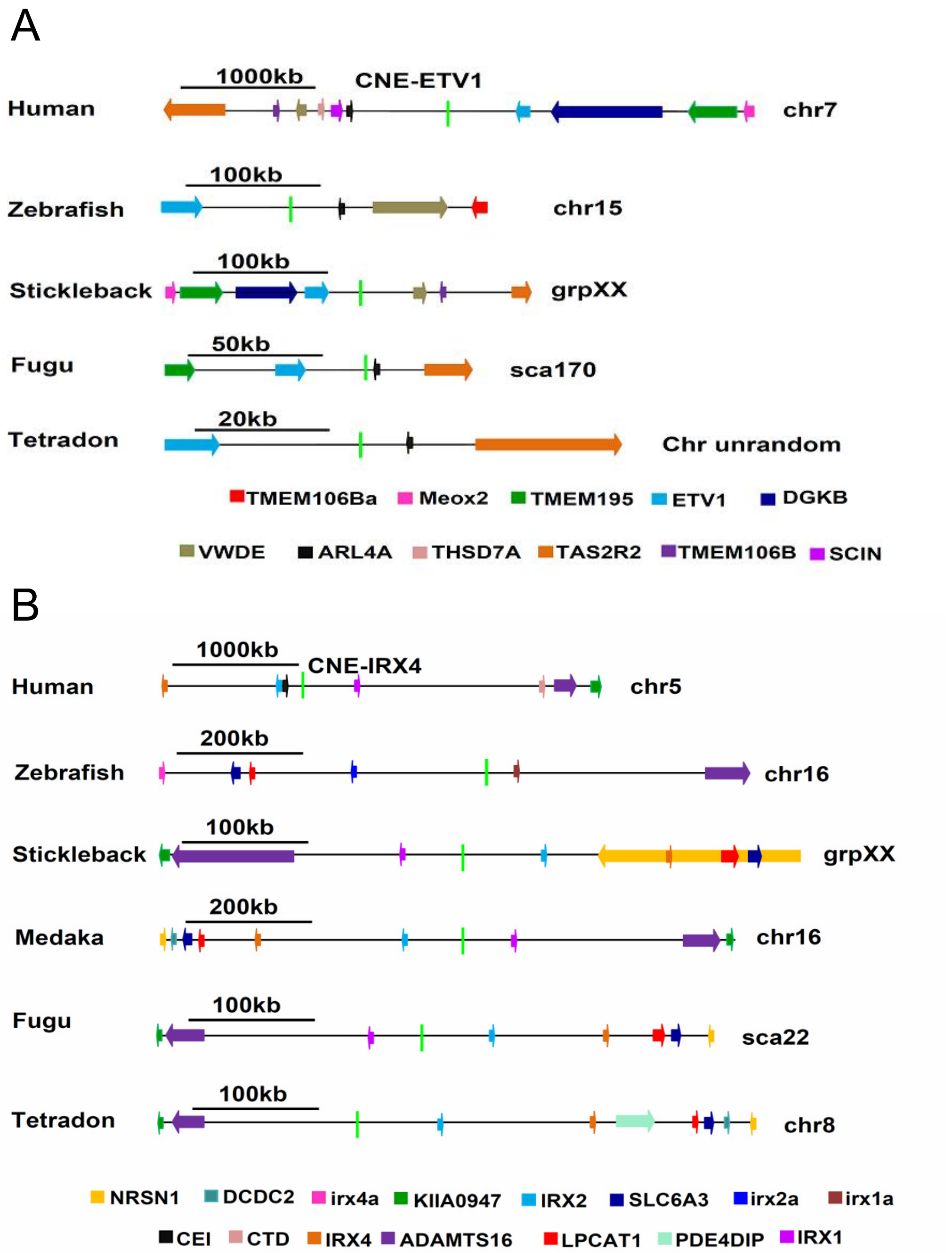
A
1000kb
CNE-ETV1
Human
chr7
100kb
Zebrafish
chr15
100kb
Stickleback
grpXX
50kb
Fugu
sca170
20kb
Tetradon
Chr unrandom
TMEM106Ba
Meox2
TMEM195
ETV1
DGKB
VWDE
ARL4A
THSD7A
TAS2R2
TMEM106B
SCIN
B
1000kb
CNE-IRX4
Human
chr5
200kb
Zebrafish
chr16
100kb
Stickleback
grpXX
200kb
Medaka
chr16
100kb
Fugu
sca22
100kb
Tetradon
chr8
NRSN1
DCDC2
irx4a
KIIA0947
IRX2
SLC6A3
irx2a
irx1a
CEI
CTD
IRX4
ADAMTS16
LPCAT1
PDE4DIP
IRX1

Figure 27. Analysis of genic environment of human CNE-enhancers in teleost fish orthologous genomic intervals helps in identifying their target genes (hs749/hs754).

Gene, controlled by specific regulatory elements, are identified through the systematic analysis of orthologous genomic content, which harbors functionally identified CNE enhancers, of tetrapod-teleost lineages. **(A)** CNE residing between *ETV1* and *ARL4A* in humans is also conserved along with *ETV1* , with differential loss and gain of bystander genes, in teleost fishes. Comparison of reporter gene expression of CNE enhancer and *ETV1* also confers *ETV1* as target gene of CNE. **(B)** Genic architecture analysis of the orthologous loci holding the CNE enhancers in vertebrate genome recommends the association of CNE with *IRX4*.

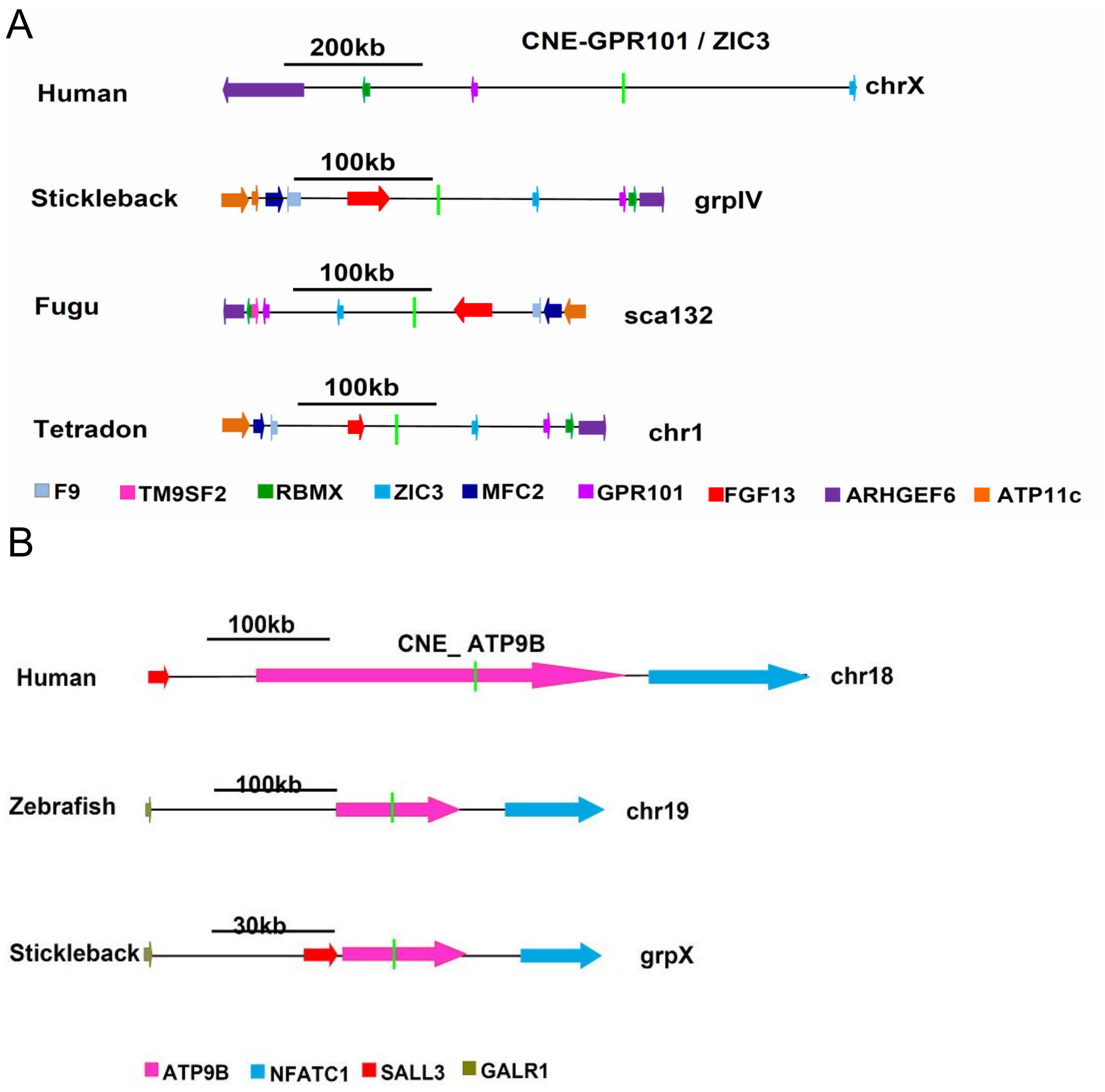
A
200kb
CNE-GPR101 / ZIC3
Human
chrX
100kb
Stickleback
grpIV
100kb
Fugu
sca132
100kb
Tetradon
chr1
F9
TM9SF2
RBMX
ZIC3
MFC2
GPR101
FGF13
ARHGEF6
ATP11c
B
100kb
CNE_ ATP9B
Human
chr18
100kb
Zebrafish
chr19
30kb
Stickleback
grpX
ATP9B
NFATC1
SALL3
GALR1

Figure 28: **Assigning the target genes to CNE-enhancers through comparative genomic analysis (hs755/ hs775).**

Analysis of CNE-enhancers in humans and its orthologous genomic intervals teleost fish lineage helps in associating CNE to their target genes**. (A)** Conservation of *GPR101* and *ZIC3* through out the vertebrate genome and endogenous expression pattern analysis of CNE matches with both these genes proposes them as target genes of CNE. **(B)** Conserved intronic localization of CNE and *ATP9B* in tetrapod-teleost lineages demonstrate the linkage between them. Redundant expression pattern analysis of CNE and *ATP9B* also emphasizes this idea.

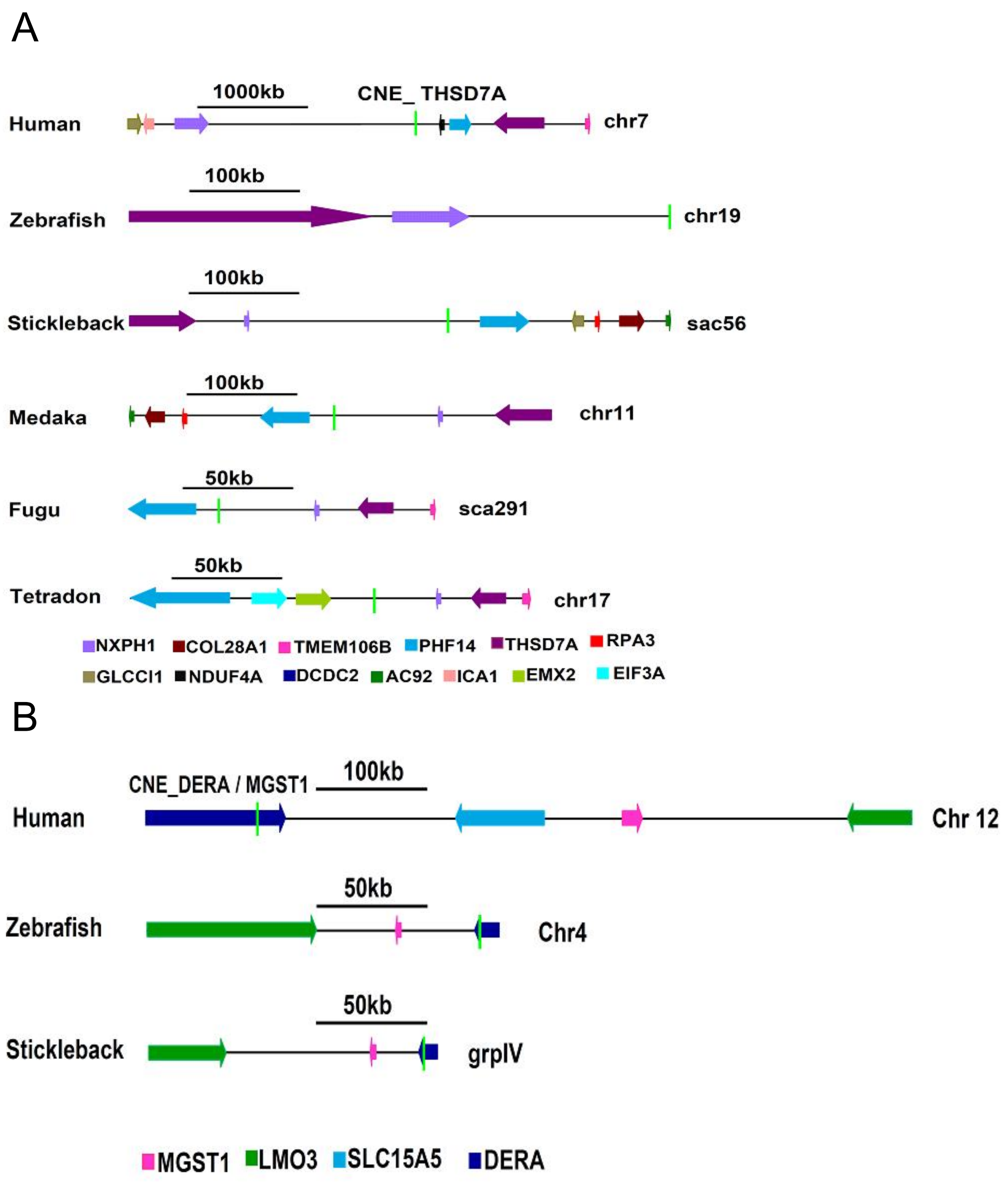
A
1000kb
CNE_ THSD7A
Human
chr7
100kb
Zebrafish
chr19
100kb
Stickleback
sac56
100kb
Medaka
chr11
50kb
Fugu
sca291
50kb
Tetradon
chr17
NXPH1
COL28A1
TMEM106B
PHF14
THSD7A
RPA3
GLCCI1
NDUF4A
DCDC2
AC92
ICA1
EMX2
EIF3A
B
CNE_DERA / MGST1
100kb
Human
Chr 12
50kb
Zebrafish
Chr4
50kb
Stickleback
grpIV
MGST1
LMO3
SLC15A5
DERA

Figure 29. Comparing the human-fish genic content and their reporter gene expression lead to association of CNE enhancers to their respective genes (hs793/hs798).

Target genes of CNE enhancers are identified through orthology mapping in Human and teleost fishes. **(A)** By tracing the genomic content around the CNE in tetrapod and fish lineages it becomes evident that the CNE is regulatory element of *THSD7A*.Redundant expression pattern of the gene and CNE is also in accordance with each other. **(B)** Conservation of *DERA* and *MGST1* through out the vertebrate genome and endogenous expression pattern analysis of CNE matches with both these genes proposes them as target genes of CNE.

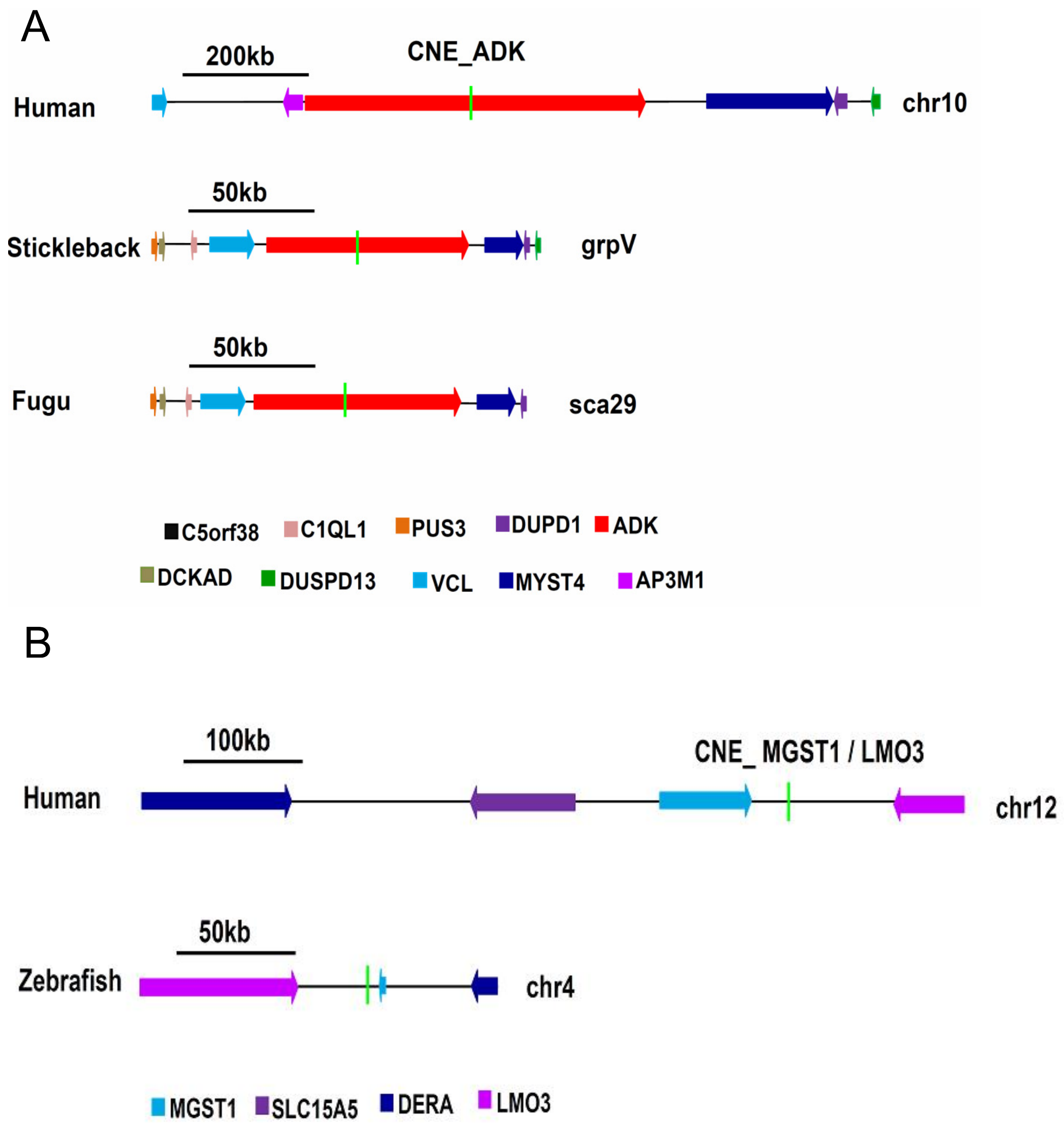
A
200kb
CNE_ADK
Human
chr10
50kb
Stickleback
grpV
50kb
Fugu
sca29
C5orf38
C1QL1
PUS3
DUPD1
ADK
DCKAD
DUSPD13
VCL
MYST4
AP3M1
B
100kb
CNE_ MGST1 / LMO3
Human
chr12
50kb
Zebrafish
chr4
MGST1
SLC15A5
DERA
LMO3

Figure 30. Analysis of genic environment of human CNE-enhancers in teleost fish orthologous genomic intervals helps in identifying their target genes (hs595/hs607).

Through Comparative genomic analysis of functionally defined CNE enhancers, across tetrapod-teleost lineages, cis regulatory networks of human genes are defined. **(A)** Not only the CNE but also its intronic location in *ADK,* conserved in both human and teleost lineages, suggests the link exists between CNE and *ADK* which is further confirmed by endogenous expression pattern analysis of both the CNE and the gene. **(B)** Conservation of *LMO3* and *MGST1* through out the vertebrate genome and endogenous expression pattern analysis of CNE matches with both these genes proposes them as target genes of CNE.

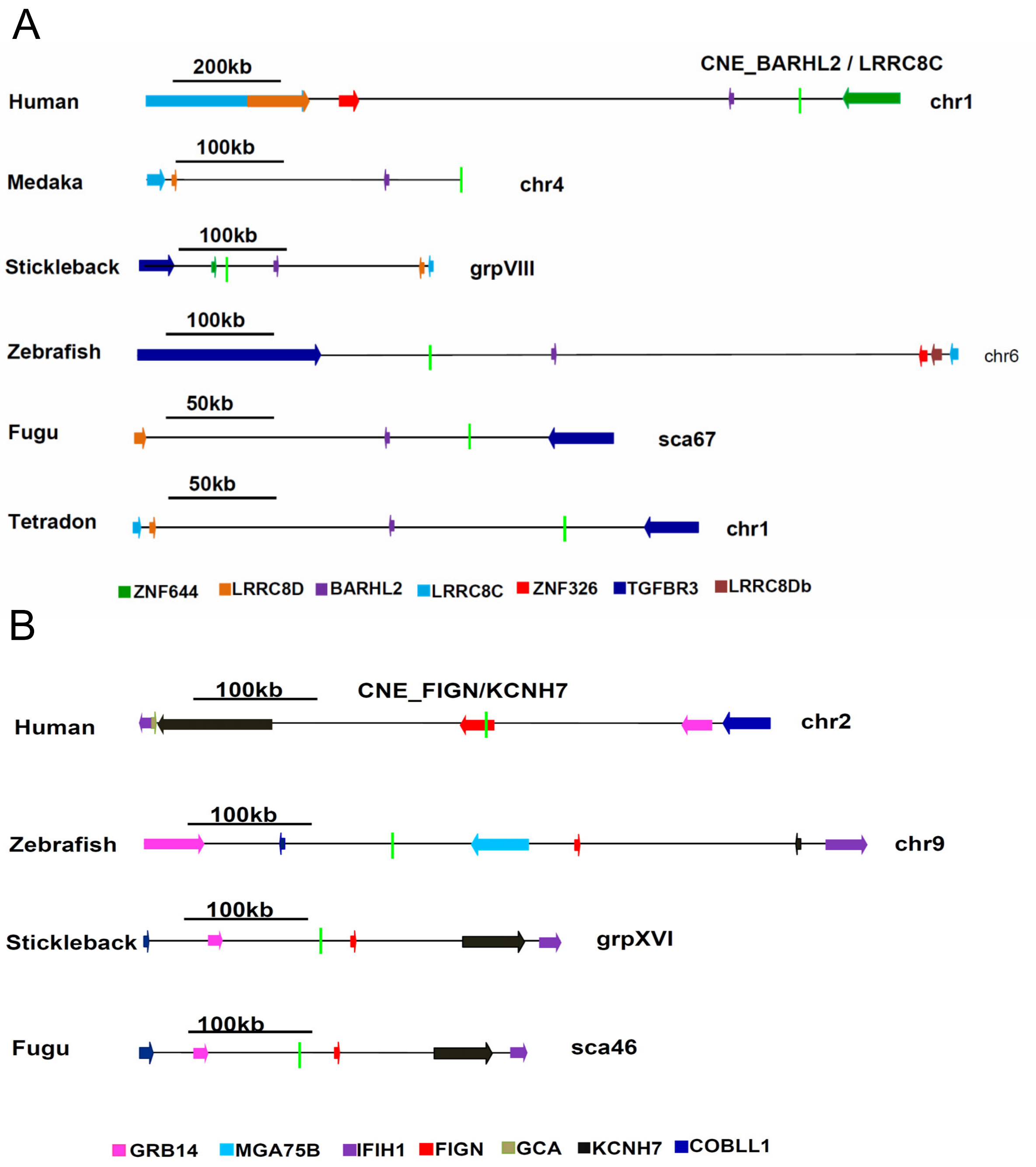
A
CNE_BARHL2 / LRRC8C
200kb
Human
chr1
100kb
Medaka
chr4
100kb
Stickleback
grpVIII
100kb
Zebrafish
chr6
50kb
Fugu
sca67
50kb
Tetradon
chr1
ZNF644 LRRC8D BARHL2 LRRC8C ZNF326 TGFBR3 LRRC8Db
B
100kb
CNE_FIGN/KCNH7
Human
chr2
100kb
Zebrafish
chr9
100kb
Stickleback
grpXVI
100kb
Fugu
sca46
GRB14 MGA75B IFIH1 FIGN GCA KCNH7 COBLL1

Figure 31. Assigning the target genes to CNE-enhancers through comparative genomic analysis (hs612/ hs640).

Target genes of CNE enhancers are identified through orthology mapping in Human and teleost fishes. **(A)** Conservation of *LBARHL2* and *LRRC8C* through out the vertebrate genome and endogenous expression pattern analysis of CNE matches with both these genes proposes them as target genes of CNE. **(B)** Conservation of *FIGN* and *KCNH7* through out the vertebrate genome and endogenous expression pattern analysis of CNE matches with both these genes proposes them as target genes of CNE.

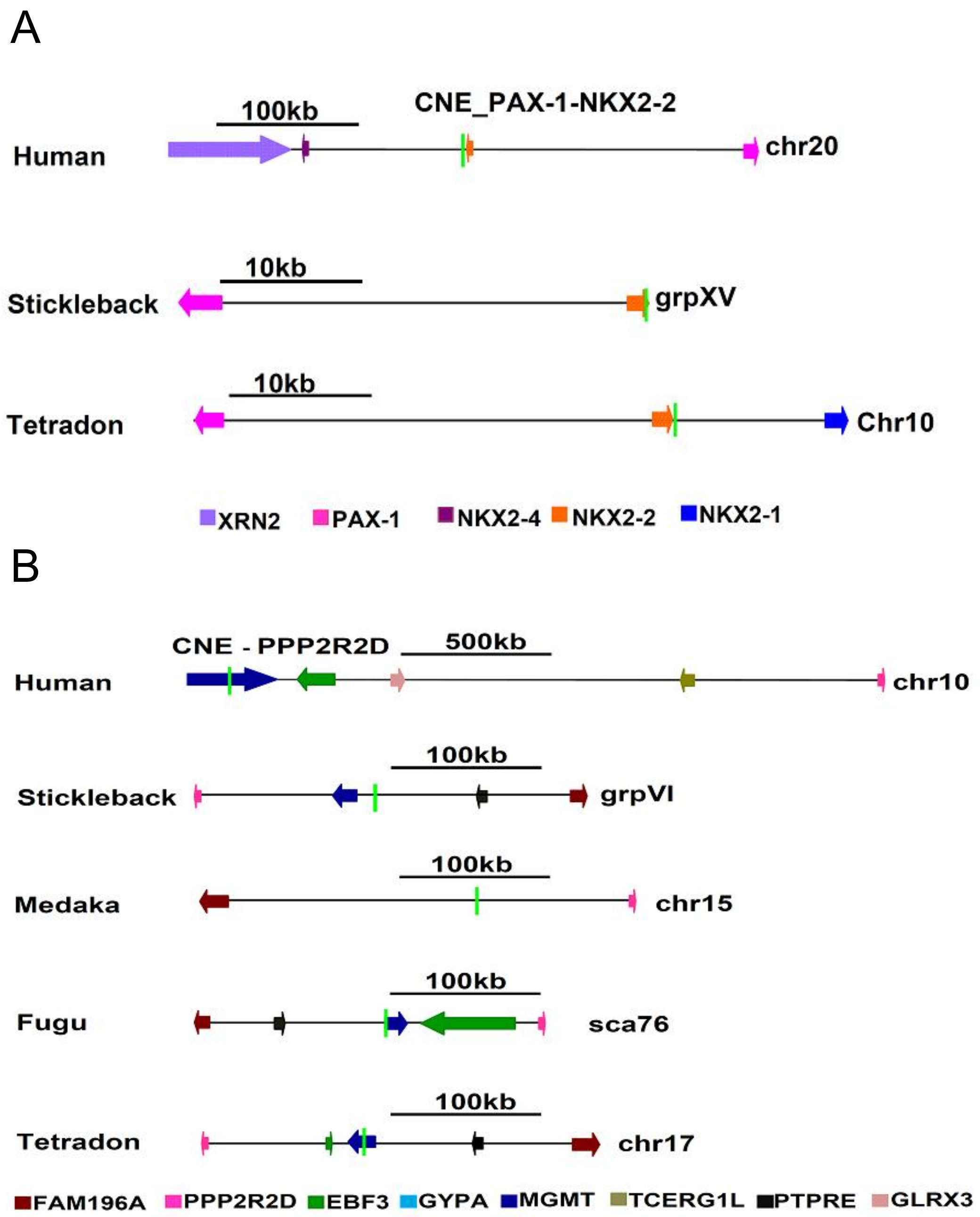
A
100kb
CNE_PAX-1-NKX2-2
Human
chr20
10kb
Stickleback
grpXV
10kb
Tetradon
Chr10
XRN2
PAX-1
NKX2-4
NKX2-2
NKX2-1
B
CNE - PPP2R2D
500kb
Human
chr10
100kb
Stickleback
grpVI
100kb
Medaka
chr15
100kb
Fugu
sca76
100kb
Tetradon
chr17
FAM196A
PPP2R2D
EBF3
GYPA
MGMT
TCERG1L
PTPRE
GLRX3

Figure 32: Comparing the human-fish genic content and their reporter gene expression lead to association of CNE enhancers to their respective genes (hs1205/hs656).

Systematic analysis of the surrounding region of CNE enhancers, conserved across the tetrapod-teleost lineages, carved a pathway to associate the CNE enhancer to its target genes. **(A)** Conservation of *PAX-1* and *NKX2-2* through out the vertebrate genome and endogenous expression pattern analysis of CNE matches with both these genes proposes them as target genes of CNE. **(B)** Analyses of expression pattern and conservation in tetrapod and fish lineages shows that CNE-enhancer is regulating the expression of *PPP2R2D*.

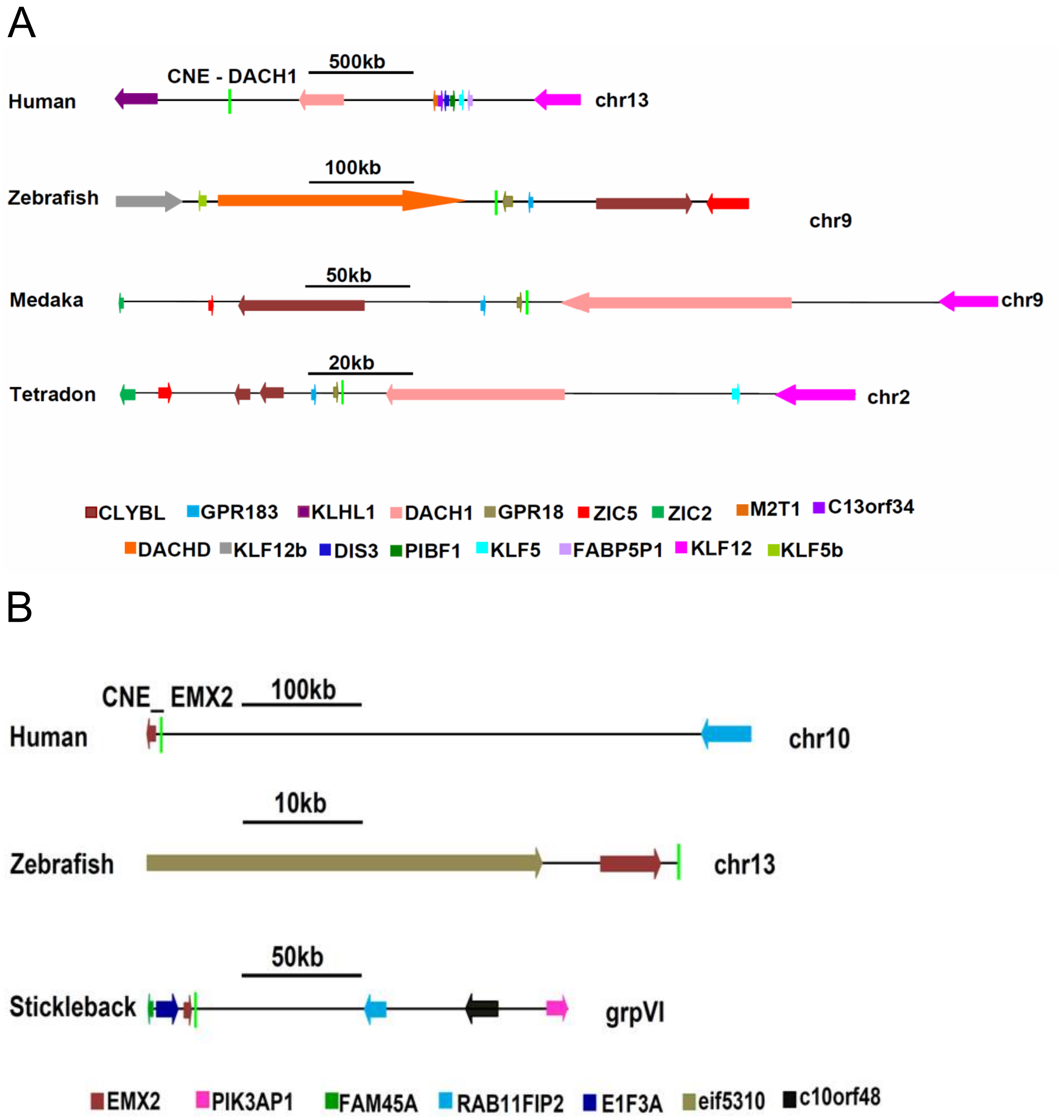
A
CNE - DACH1
500kb
Human
chr13
100kb
Zebrafish
chr9
50kb
Medaka
chr9
20kb
Tetradon
chr2
CLYBL
GPR183
KLHL1
DACH1
GPR18
ZIC5
ZIC2
M2T1
C13orf34
DACHD
KLF12b
DIS3
PIBF1
KLF5
FABP5P1
KLF12
KLF5b
B
CNE_ EMX2
100kb
Human
chr10
10kb
Zebrafish
chr13
50kb
Stickleback
grpVI
EMX2
PIK3AP1
FAM45A
RAB11FIP2
E1F3A
eif5310
c10orf48

Figure 33. **Human CNE-enhancers by tracing the genic context of their orthologous copies in teleost fish lineage (hs540/hs672).**

Analysis of CNE-enhancers in humans and its orthologous genomic intervals teleost fish lineage helps in associating CNE to their target genes**.** **(A)** Analyses of expression pattern and conservation in tetrapod and fish lineages shows that CNE-enhancer is regulating the expression of *DACH1.* **(B)** Genic architecture analysis of the orthologous loci holding the CNE enhancers in vertebrate genome recommends the association of CNE with *EMX2*.

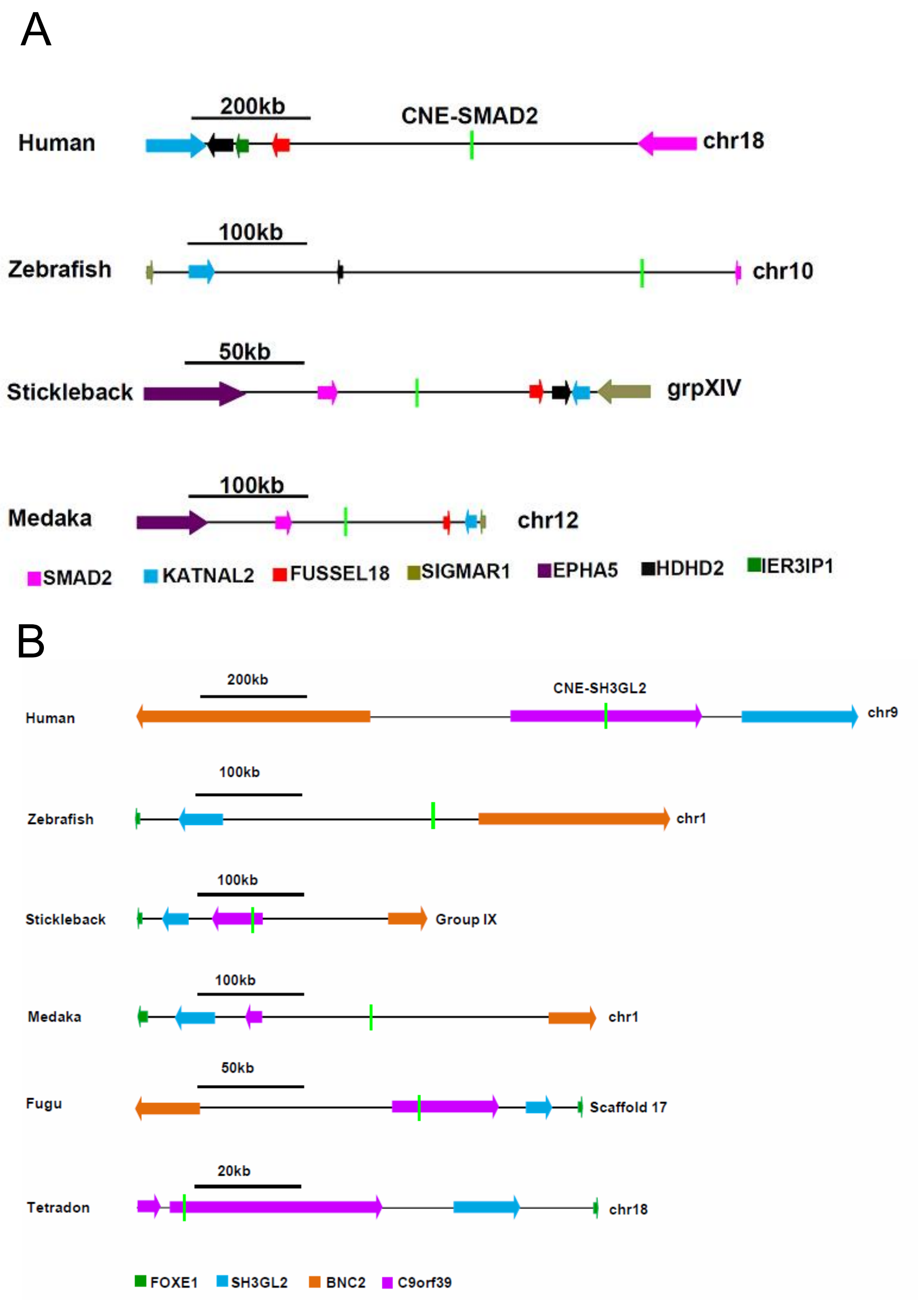
A
Human
200kb
CNE-SMAD2
chr18
Zebrafish
100kb
chr10
Stickleback
50kb
grpXIV
Medaka
100kb
chr12
SMAD2
KATNAL2
FUSSEL18
SIGMAR1
EPHA5
HDHD2
IER3IP1
B
Human
200kb
CNE-SH3GL2
chr9
Zebrafish
100kb
chr1
Stickleback
100kb
Group IX
Medaka
100kb
chr1
Fugu
50kb
Scaffold 17
Tetradon
20kb
chr18
FOXE1
SH3GL2
BNC2
C9orf39

Figure 34. Analysis of genic environment of human CNE-enhancers in teleost fish orthologous genomic intervals helps in identifying their target genes (hs679/hs529).

Gene, controlled by specific regulatory elements, are identified through the systematic analysis of orthologous genomic content, which harbors functionally identified CNE enhancers, of tetrapod-teleost lineages. **(A)** CNE residing between *FUSSEL18* and *SMAD2* in humans is also conserved along with *SMAD2* , with differential loss and gain of bystander genes, in teleost fishes. Comparison of reporter gene expression of CNE enhancer and *SMAD2* also confers *SMAD2* as target gene of CNE. **(B)** By tracing the genomic content around the CNE in tetrapod and fish lineages it becomes evident that the CNE is regulatory element of *SH3GL2*. Redundant expression pattern of the gene and CNE is also in accordance with each other.

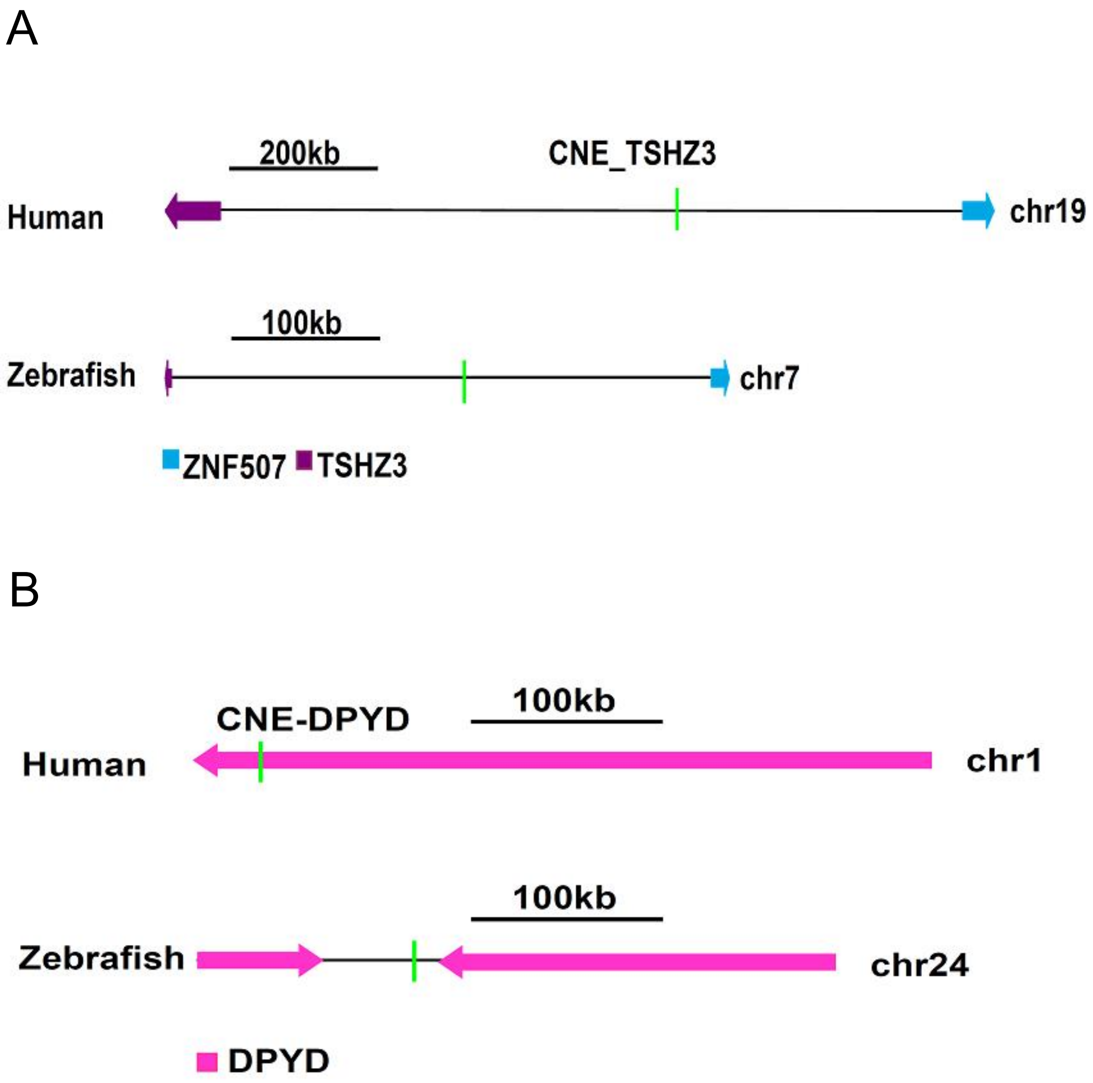
A
200kb
CNE_TSHZ3
Human
chr19
100kb
Zebrafish
chr7
ZNF507
TSHZ3
B
100kb
CNE-DPYD
Human
chr1
100kb
Zebrafish
chr24
DPYD

Figure 35. **Assigning the target genes to CNE-enhancers through comparative genomic analysis (hs536/ hs671).**

Through Comparative genomic analysis of functionally defined CNE enhancers, across tetrapod-teleost lineages, cis regulatory networks of human genes are defined. **(A)** By tracing the genomic content around the CNE in tetrapod and fish lineages it becomes evident that the CNE is regulatory element of *TSHZ3*. Redundant expression pattern of the gene and CNE is also in accordance with each other. **(B)** Analyses of expression pattern and conservation in tetrapod and fish lineages shows that CNE-enhancer is regulating the expression of *DPYD.*

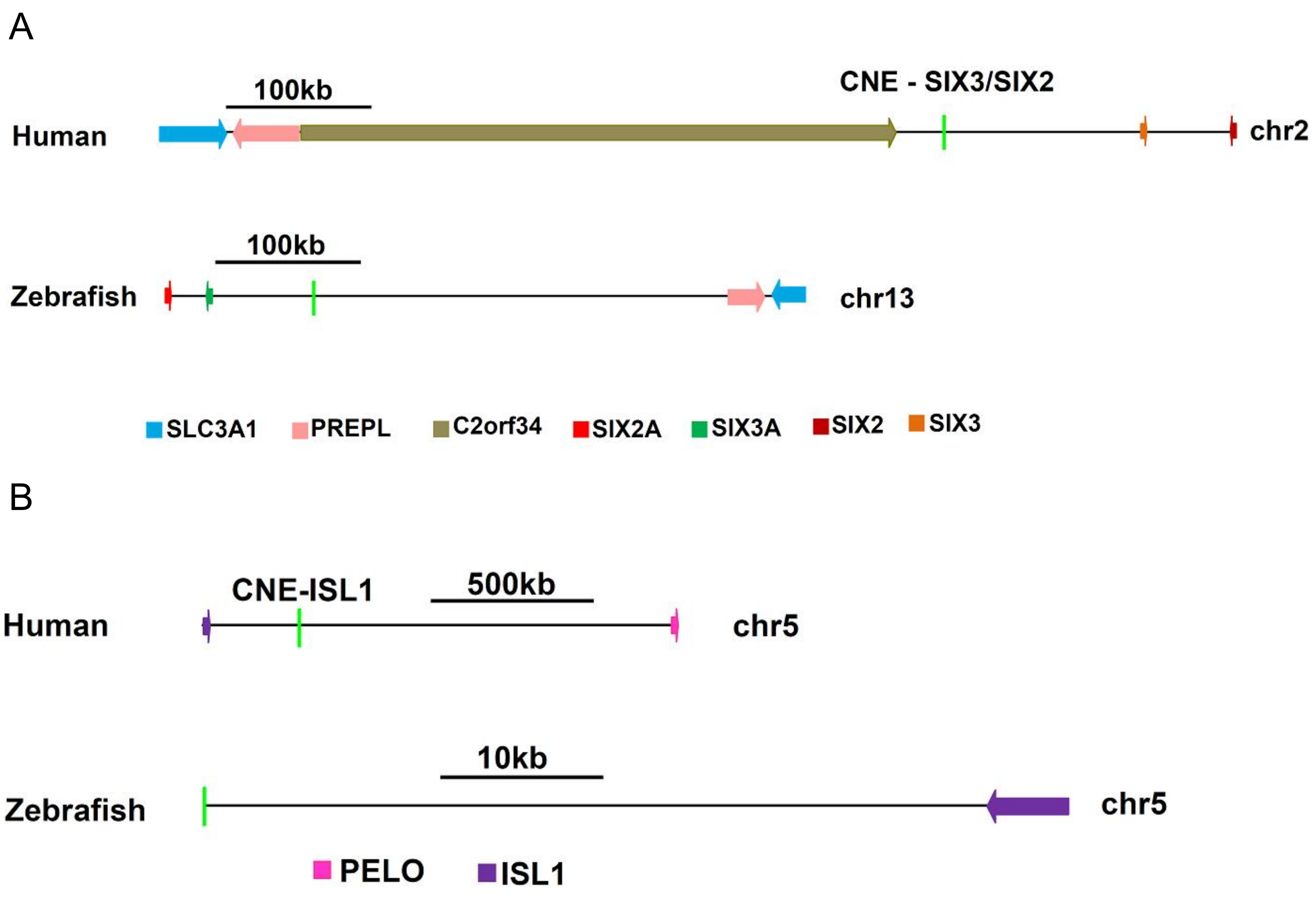

A
100kb
CNE - SIX3/SIX2
Human
chr2
100kb
Zebrafish
chr13
SLC3A1
PREPL
C2orf34
SIX2A
SIX3A
SIX2
SIX3
B
CNE-ISL1
500kb
Human
chr5
10kb
Zebrafish
chr5
PELO
ISL1

Figure 36. Comparing the human-fish genic content and their reporter gene expression lead to association of CNE enhancers to their respective genes (hs541/hs543).

Target genes of CNE enhancers are identified through orthology mapping in Human and teleost fishes. **(A)** Conservation of *SIX2* and *SIX3* through out the vertebrate genome and endogenous expression pattern analysis of CNE matches with both these genes proposes them as target genes of CNE. **(B)** Genic architecture analysis of the orthologous loci holding the CNE enhancers in vertebrate genome recommends the association of CNE with *ISL1*.

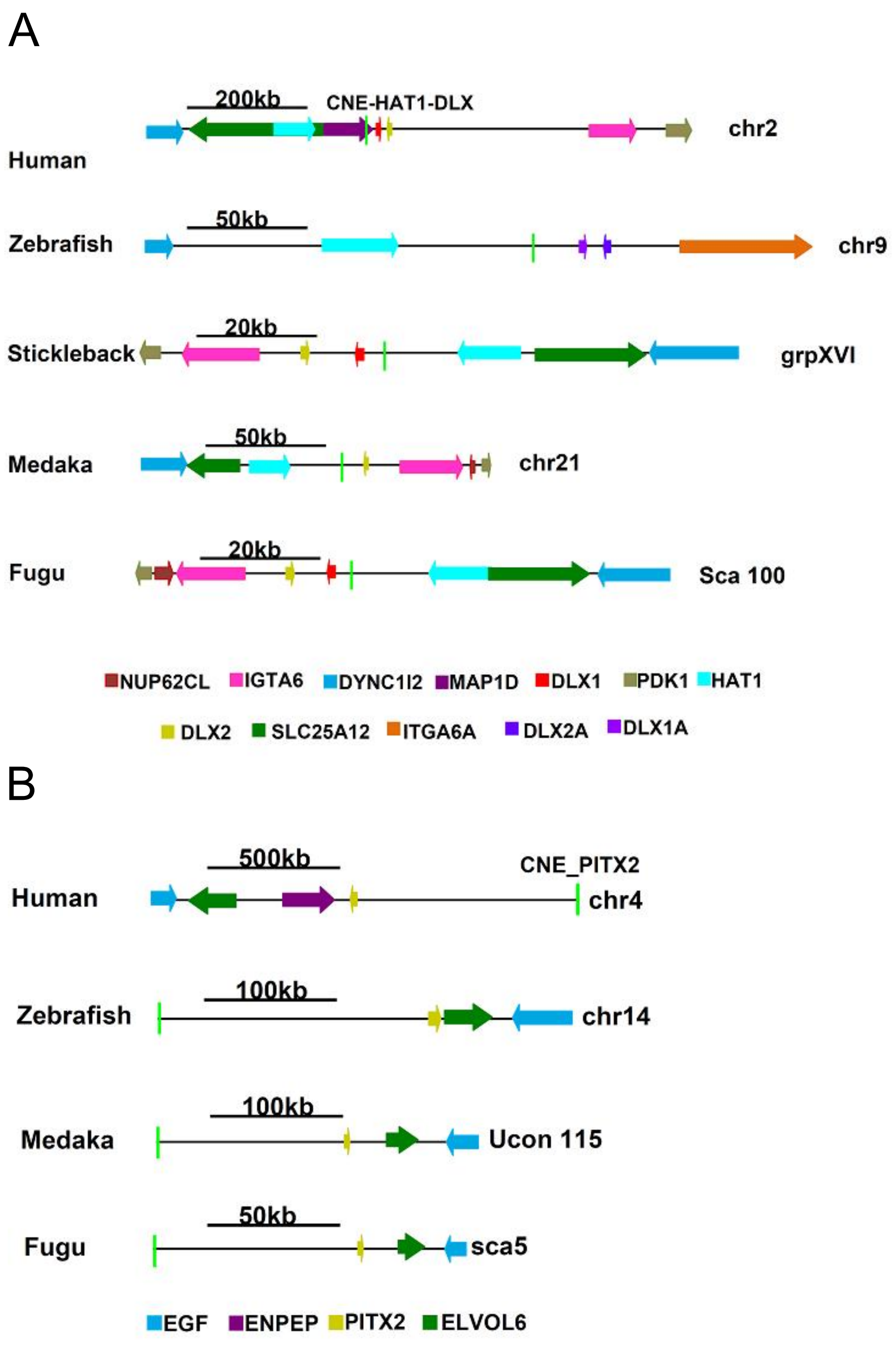

A
200kb
CNE-HAT1-DLX
Human
chr2
Zebrafish
50kb
chr9
Stickleback
20kb
grpXVI
Medaka
50kb
chr21
Fugu
20kb
Sca 100
NUP62CL
IGTA6
DYNC1I2
MAP1D
DLX1
PDK1
HAT1
DLX2
SLC25A12
ITGA6A
DLX2A
DLX1A
B
Human
500kb
CNE_PITX2
chr4
Zebrafish
100kb
chr14
Medaka
100kb
Ucon 115
Fugu
50kb
sca5
EGF
ENPEP
PITX2
ELVOL6

Figure 37. Human CNE-enhancers by tracing the genic context of their orthologous copies in teleost fish lineage (hs553/hs559).

Analysis of CNE-enhancers in humans and its orthologous genomic intervals teleost fish lineage helps in associating CNE to their target genes**.** **(A)** Conservation of *HAT1* and *DLX* through out the vertebrate genome and endogenous expression pattern analysis of CNE matches with both these genes proposes them as target genes of CNE. **(B)** Analysis of expression pattern and conservation in tetrapod and fish lineages shows that CNE-enhancer is regulating the expression of *PITX2.*

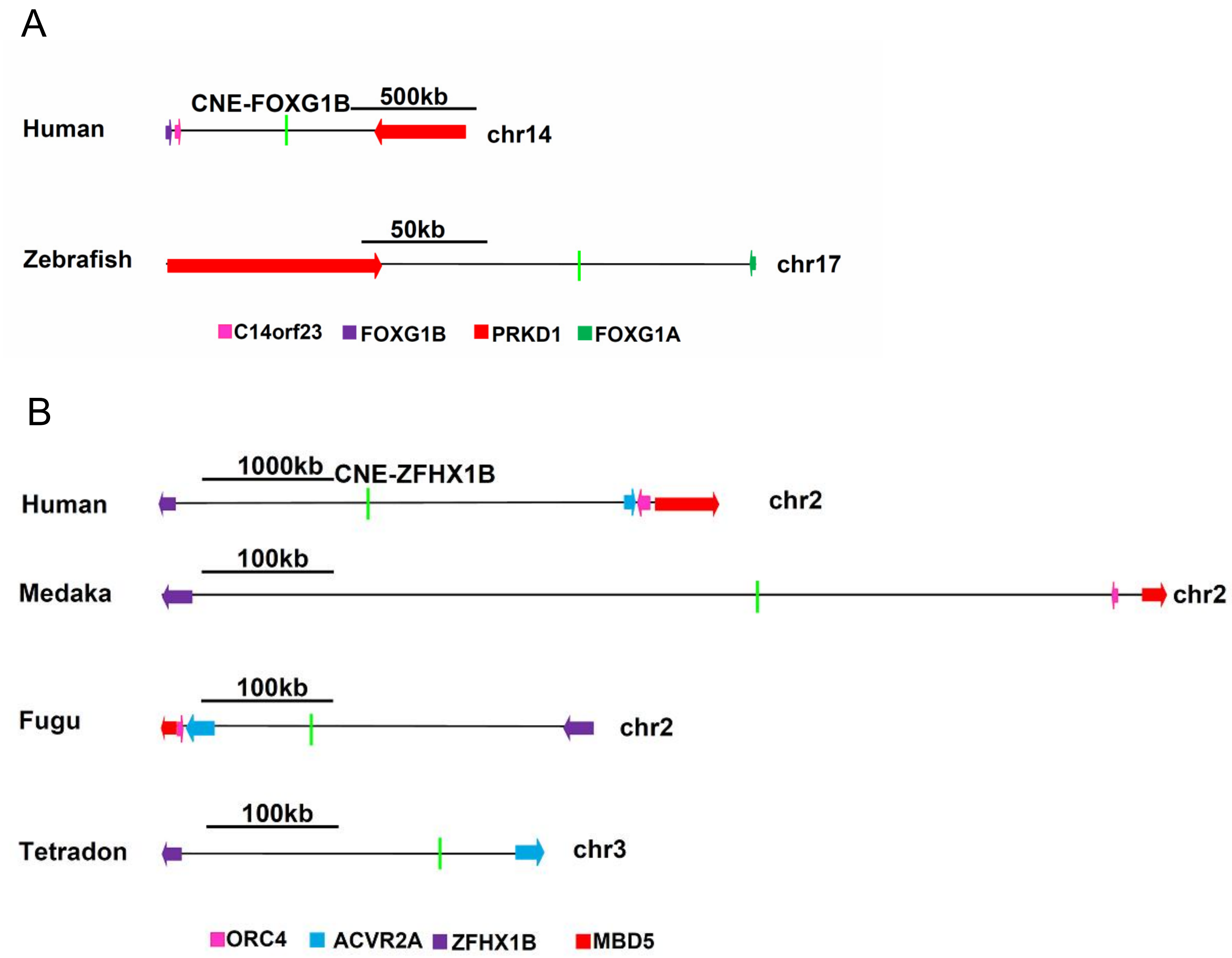

A
Human
CNE-FOXG1B
500kb
chr14
Zebrafish
50kb
chr17
C14orf23
FOXG1B
PRKD1
FOXG1A
B
1000kb
CNE-ZFHX1B
Human
chr2
Medaka
100kb
chr2
Fugu
100kb
chr2
Tetradon
100kb
chr3
ORC4
ACVR2A
ZFHX1B
MBD5

Figure 38. Analysis of genic environment of human CNE-enhancers in teleost fish orthologous genomic intervals helps in identifying their target genes (hs566/hs568).

Systematic analysis of the surrounding region of CNE enhancers, conserved across the tetrapod-teleost lineages, carved a pathway to associate the CNE enhancer to its target genes. **(A)** By tracing the genomic content around the CNE in tetrapod and fish lineages it becomes evident that the CNE is regulatory element of *FOXG1B*. Redundant expression pattern of the gene and CNE is also in accordance with each other. **(B)** Analysis of expression pattern and conservation in tetrapod and fish lineages shows that CNE-enhancer is regulating the expression of *ZFHX1B.*

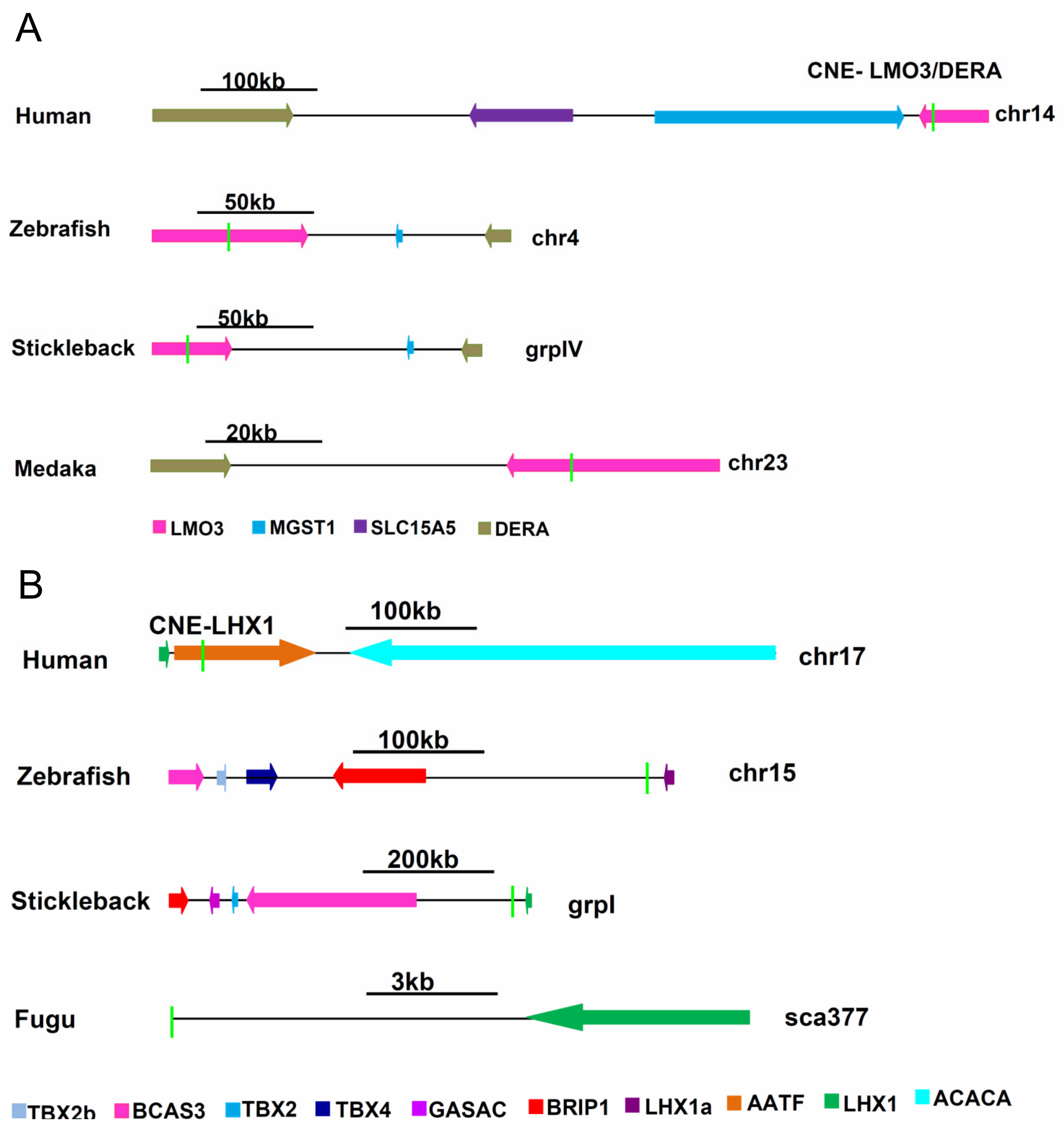
A
100kb
CNE- LMO3/DERA
Human
chr14
50kb
Zebrafish
chr4
50kb
Stickleback
grpIV
20kb
Medaka
chr23
LMO3
MGST1
SLC15A5
DERA
B
CNE-LHX1
100kb
Human
chr17
100kb
Zebrafish
chr15
200kb
Stickleback
grpI
3kb
Fugu
sca377
TBX2b
BCAS3
TBX2
TBX4
GASAC
BRIP1
LHX1a
AATF
LHX1
ACACA

Figure 39. Assigning the target genes to CNE-enhancers through comparative genomic analysis (hs336 /hs363).

Gene, controlled by specific regulatory elements, are identified through the systematic analysis of orthologus genomic content, which harbors functionally identified CNE enhancers, of tetrapod-teleost lineages. **(A)** Conservation of *LMO3* and *DERA* through out the vertebrate genome and endogenous expression pattern analysis of CNE matches with both these genes proposes them as target genes of CNE. **(B)** By tracing the genomic content around the CNE in tetrapod and fish lineages it becomes evident that the CNE is regulatory element of *LHX1*. Redundant expression pattern of the gene and CNE is also in accordance with each other.

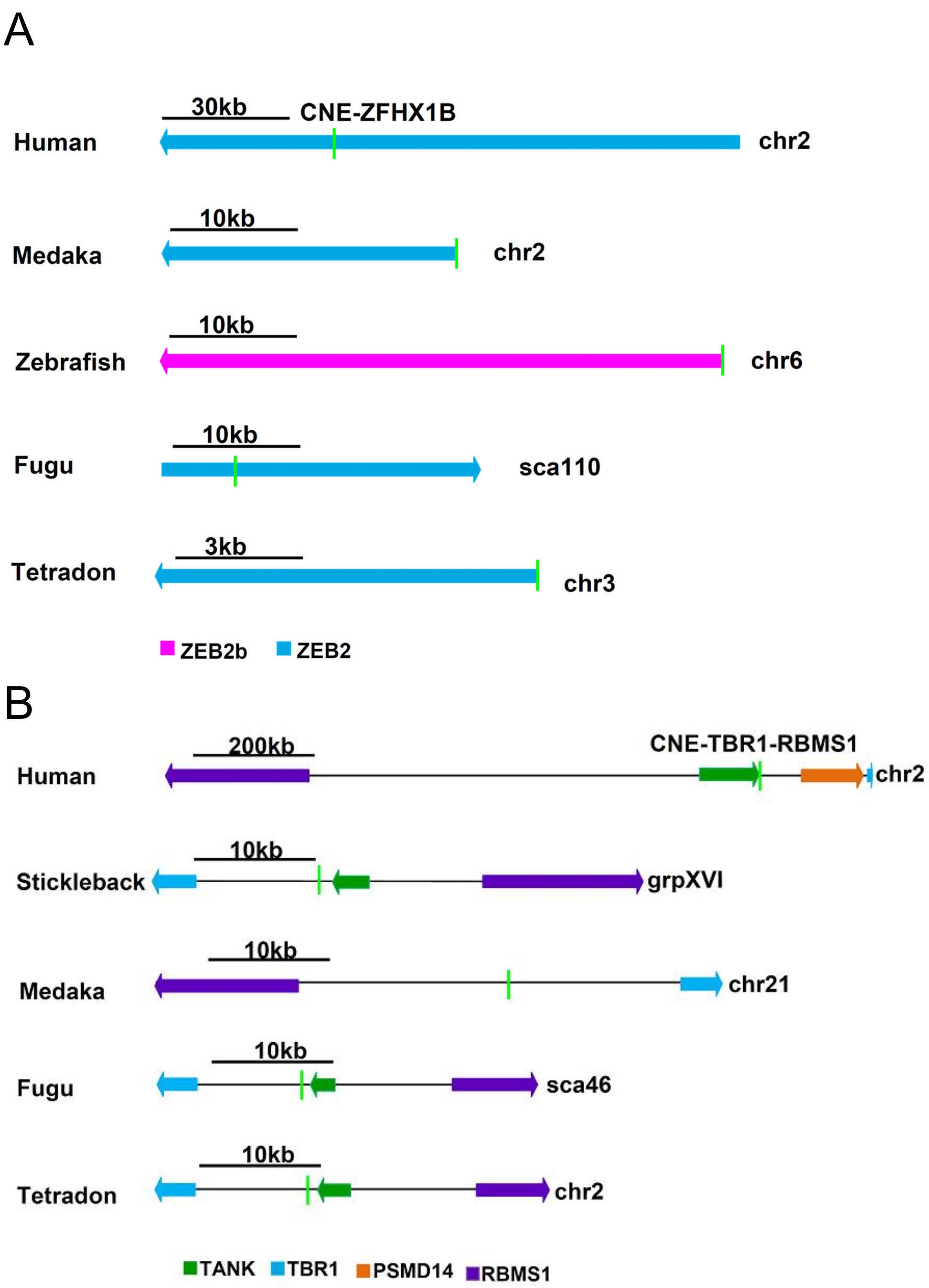
A
30kb
CNE-ZFHX1B
Human
chr2
10kb
Medaka
chr2
10kb
Zebrafish
chr6
10kb
Fugu
sca110
3kb
Tetradon
chr3
ZEB2b
ZEB2
B
200kb
CNE-TBR1-RBMS1
Human
chr2
10kb
Stickleback
grpXVI
10kb
Medaka
chr21
10kb
Fugu
sca46
10kb
Tetradon
chr2
TANK
TBR1
PSMD14
RBMS1

Figure 40. Comparing the human-fish genic content and their reporter gene expression lead to association of CNE enhancers to their respective genes (hs407/hs416).

Through Comparative genomic analysis of functionally defined CNE enhancers, across tetrapod-teleost lineages, cis regulatory networks of human genes are defined. **(A)** Analysis of expression pattern and conservation in tetrapod and fish lineages shows that CNE-enhancer is regulating the expression of *ZFHX1B*. **(B)** Conservation of *TBR1* and *RBMS1* through out the vertebrate genome and endogenous expression pattern analysis of CNE matches with both these genes proposes them as target genes of CNE.

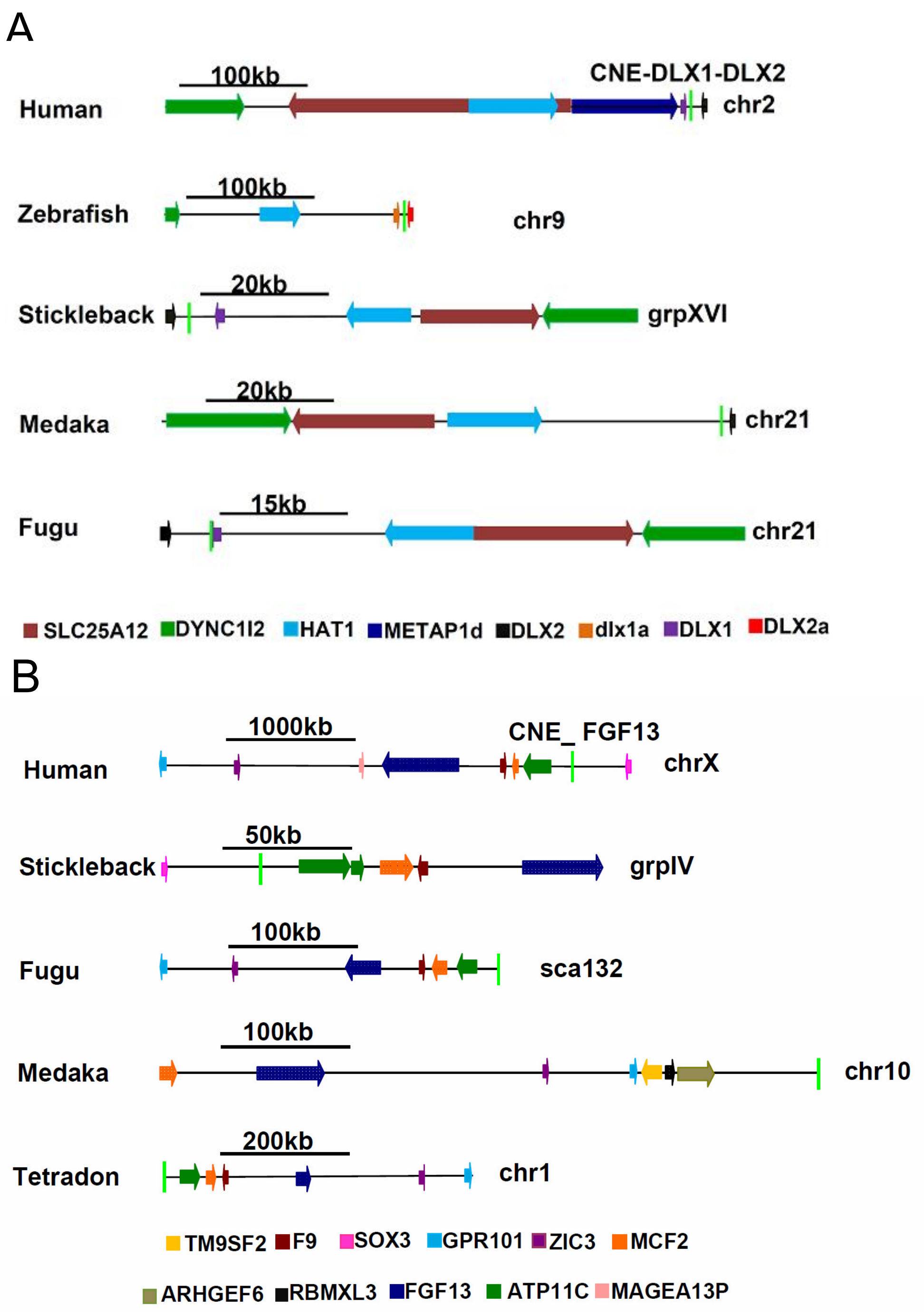
A
100kb
CNE-DLX1-DLX2
Human
chr2
100kb
Zebrafish
chr9
20kb
Stickleback
grpXVI
20kb
Medaka
chr21
15kb
Fugu
chr21
SLC25A12
DYNC1I2
HAT1
METAP1d
DLX2
dlx1a
DLX1
DLX2a
B
1000kb
CNE_ FGF13
Human
chrX
50kb
Stickleback
grpIV
100kb
Fugu
sca132
100kb
Medaka
chr10
200kb
Tetradon
chr1
TM9SF2
F9
SOX3
GPR101
ZIC3
MCF2
ARHGEF6
RBMXL3
FGF13
ATP11C
MAGEA13P

Figure 41. Human CNE-enhancers by tracing the genic context of their orthologous copies in teleost fish lineage (hs422/hs427).

Analysis of CNE-enhancers in humans and its orthologous genomic intervals teleost fish lineage helps in associating CNE to their target genes. **(A)** Conservation of *DLX1* and *DLX2* through out the vertebrate genome and endogenous expression pattern analysis of CNE matches with both these genes proposes them as target genes of CNE. **(B)** Genic architecture analysis of the orthologous loci holding the CNE enhancers in vertebrate genome recommends the association of CNE with *FGF13*.

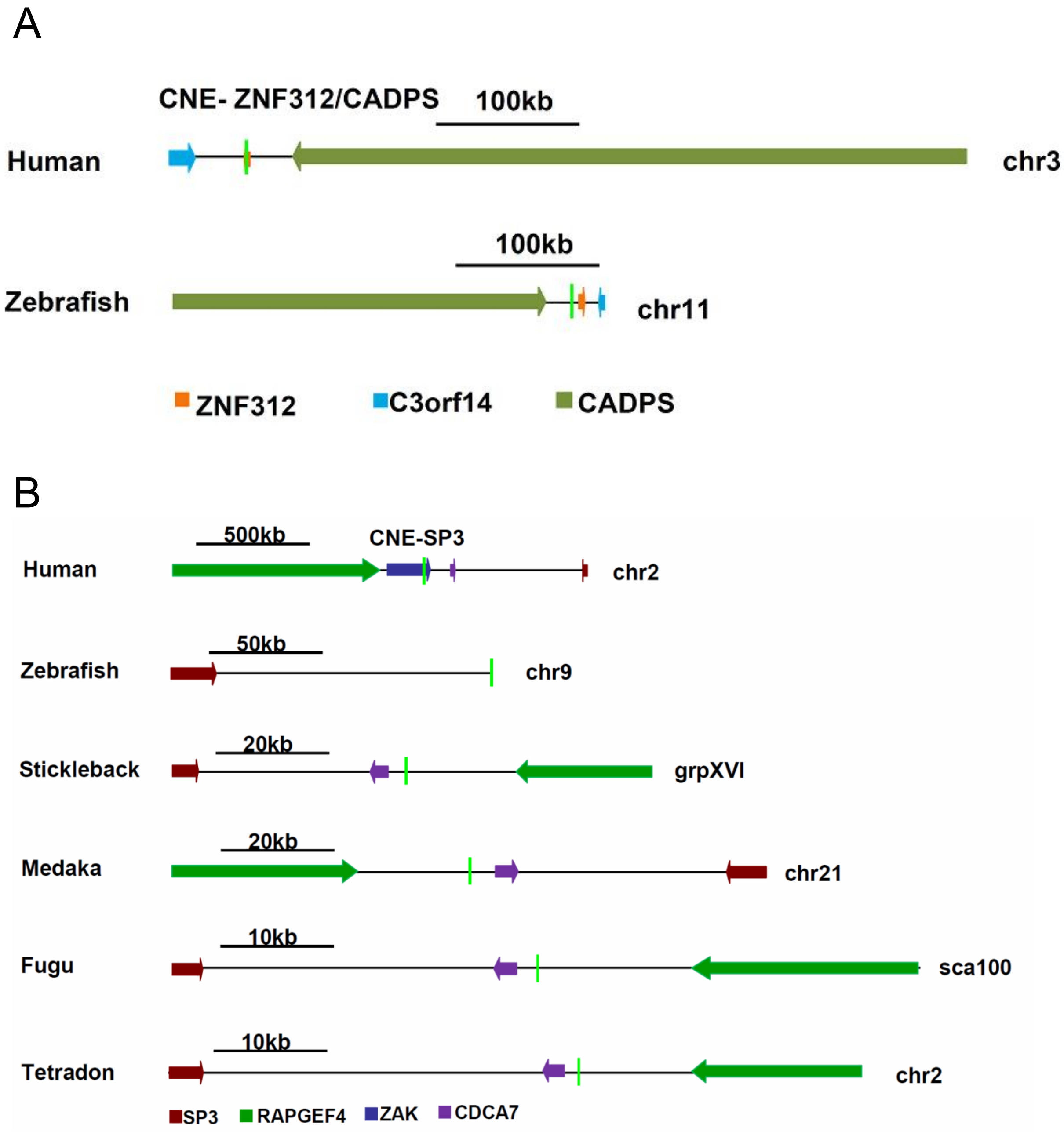
A
CNE- ZNF312/CADPS
100kb
Human
chr3
100kb
Zebrafish
chr11
ZNF312
C3orf14
CADPS
B
500kb
CNE-SP3
Human
chr2
50kb
Zebrafish
chr9
20kb
Stickleback
grpXVI
20kb
Medaka
chr21
10kb
Fugu
sca100
10kb
Tetradon
chr2
SP3
RAPGEF4
ZAK
CDCA7

Figure 42. Analysis of genic environment of human CNE-enhancers in teleost fish orthologous genomic intervals helps in identifying their target genes (hs435/hs242).

Systematic analysis of the surrounding region of CNE enhancers, conserved across the tetrapod-teleost lineages, carved a pathway to associate the CNE enhancer to its target genes. **(A)** Conservation of *ZNF312* and *CADPS* through out the vertebrate genome and endogenous expression pattern analysis of CNE matches with both these genes proposes them as target genes of CNE. **(B)** Analysis of expression pattern and conservation in tetrapod and fish lineages shows that CNE-enhancer is regulating the expression of *SP3*.

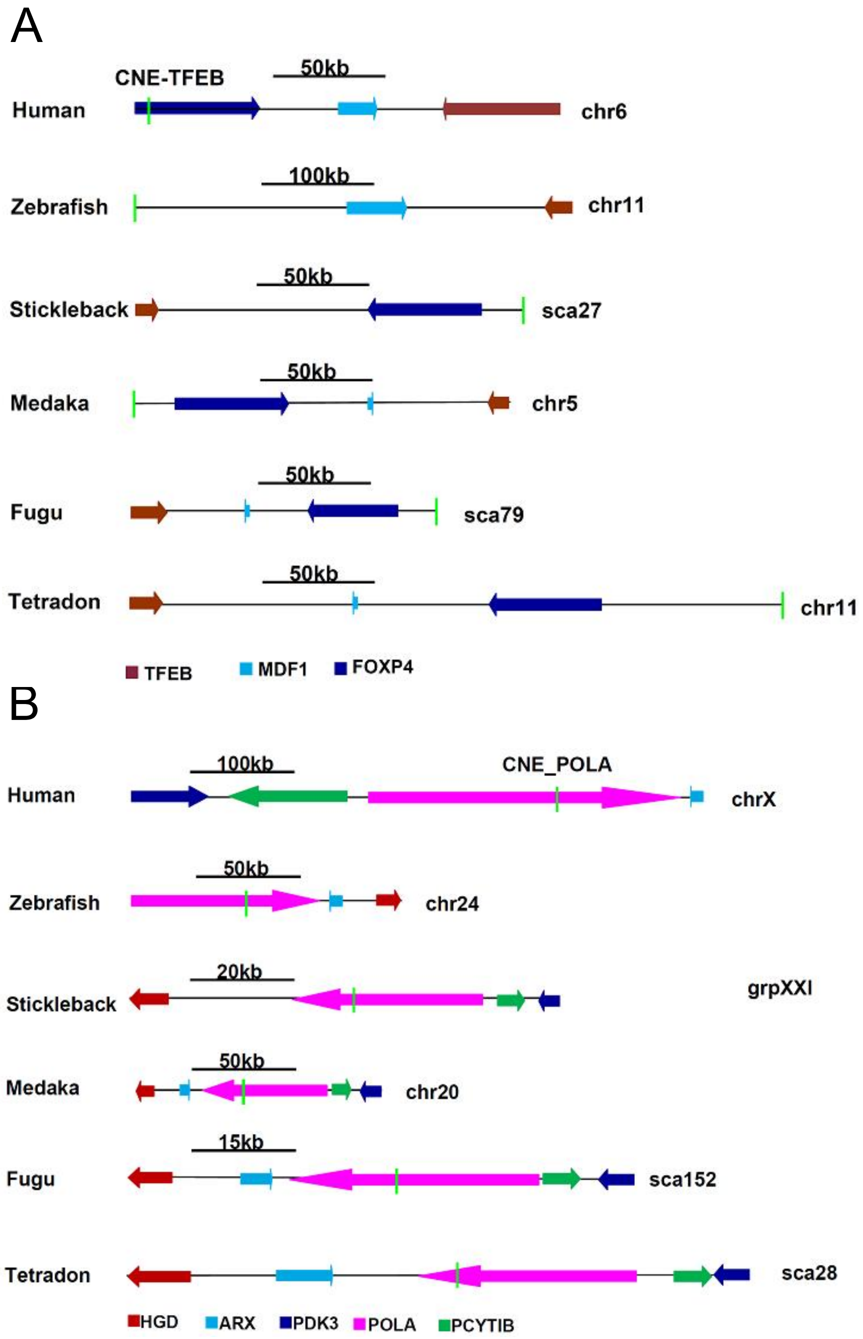
A
CNE-TFEB
50kb
Human
chr6
100kb
Zebrafish
chr11
50kb
Stickleback
sca27
50kb
Medaka
chr5
50kb
Fugu
sca79
50kb
Tetradon
chr11
TFEB
MDF1
FOXP4
B
100kb
CNE_POLA
Human
chrX
50kb
Zebrafish
chr24
20kb
Stickleback
grpXXI
50kb
Medaka
chr20
15kb
Fugu
sca152
Tetradon
sca28
HGD
ARX
PDK3
POLA
PCYTIB

Figure 43. Assigning the target genes to CNE-enhancers through comparative genomic analysis (hs281/hs118).

Gene, controlled by specific regulatory elements, are identified through the systematic analysis of orthologous genomic content, which harbors functionally identified CNE enhancers, of tetrapod-teleost lineages. **(A)** Genic architecture analysis of the orthologous loci holding the CNE enhancers in vertebrate genome recommends the association of CNE with *TFEB*. **(B)** By tracing the genomic content around the CNE in tetrapod and fish lineages it becomes evident that the CNE is regulatory element of *POLA*. Redundant expression pattern of the gene and CNE is also in accordance with each other.

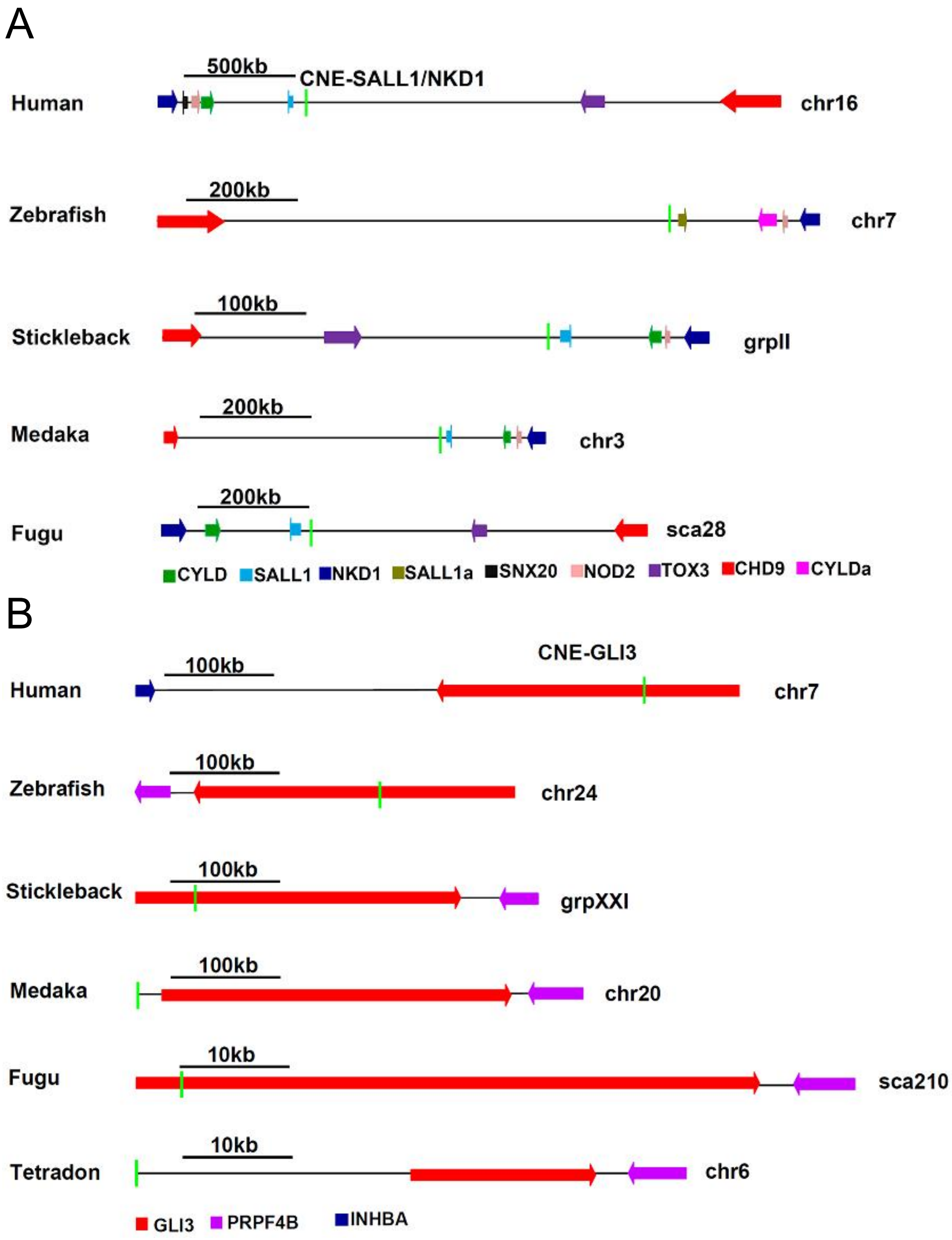
A
Human
500kb
CNE-SALL1/NKD1
chr16
Zebrafish
200kb
chr7
Stickleback
100kb
grpII
Medaka
200kb
chr3
Fugu
200kb
sca28
CYLD SALL1 NKD1 SALL1a SNX20 NOD2 TOX3 CHD9 CYLDa
B
Human
100kb
CNE-GLI3
chr7
Zebrafish
100kb
chr24
Stickleback
100kb
grpXXI
Medaka
100kb
chr20
Fugu
10kb
sca210
Tetradon
10kb
chr6
GLI3 PRPF4B INHBA

Figure 44. Comparing the human-fish genic content and their reporter gene expression lead to association of CNE enhancers to their respective genes (hs76/hs111).

Through Comparative genomic analysis of functionally defined CNE enhancers, across tetrapod-teleost lineages, cis regulatory networks of human genes are defined. **(A)** Conservation of *SALL1* and *NKD1* through out the vertebrate genome and endogenous expression pattern analysis of CNE matches with both these genes proposes them as target genes of CNE. **(B)** Analysis of expression pattern and conservation in tetrapod and fish lineages shows that CNE-enhancer is regulating the expression of *GLI3*.

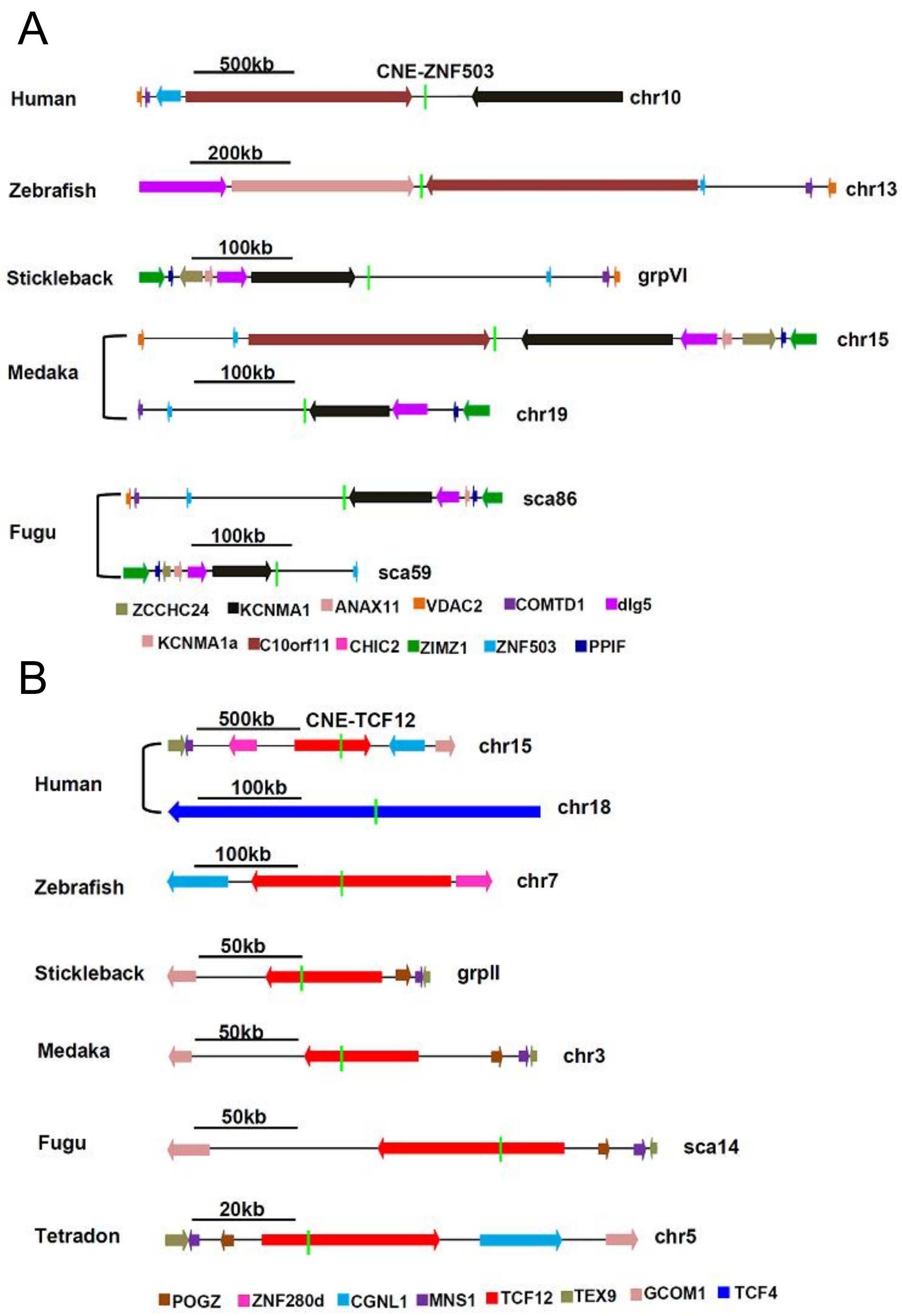
A
500kb
CNE-ZNF503
Human
chr10
200kb
Zebrafish
chr13
100kb
Stickleback
grpVI
Medaka
chr15
100kb
chr19
Fugu
sca86
100kb
sca59
ZCCHC24 KCNMA1 ANAX11 VDAC2 COMTD1 dlg5
KCNMA1a C10orf11 CHIC2 ZIMZ1 ZNF503 PPIF
B
500kb
CNE-TCF12
Human
chr15
100kb
chr18
100kb
Zebrafish
chr7
50kb
Stickleback
grpII
50kb
Medaka
chr3
50kb
Fugu
sca14
20kb
Tetradon
chr5
POGZ ZNF280d CGNL1 MNS1 TCF12 TEX9 GCOM1 TCF4

Figure 45. Predicting the target genes of human enhancers by analyzing their genic environment within and across vertebrate genomes (hs484/hs357).

Analysis of CNE-enhancers in humans and its orthologous genomic intervals teleost fish lineage helps in associating CNE to their target genes. **(A)** Analysis of expression pattern and conservation in tetrapod and fish lineages shows that CNE-enhancer is regulating the expression of *ZNF503*. Conservation of duplicated copy of this gene along with the CNE in fishes verifies this suggestion. **(B)** Duplication event of the CNE occurred only in humans and the gene entangled with the CNE, in duplicated copy, is TCF. Genic architecture analysis of the orthologous and paralogous loci holding the CNE enhancers in vertebrate genome recommends the association of CNE with *TCF12.*

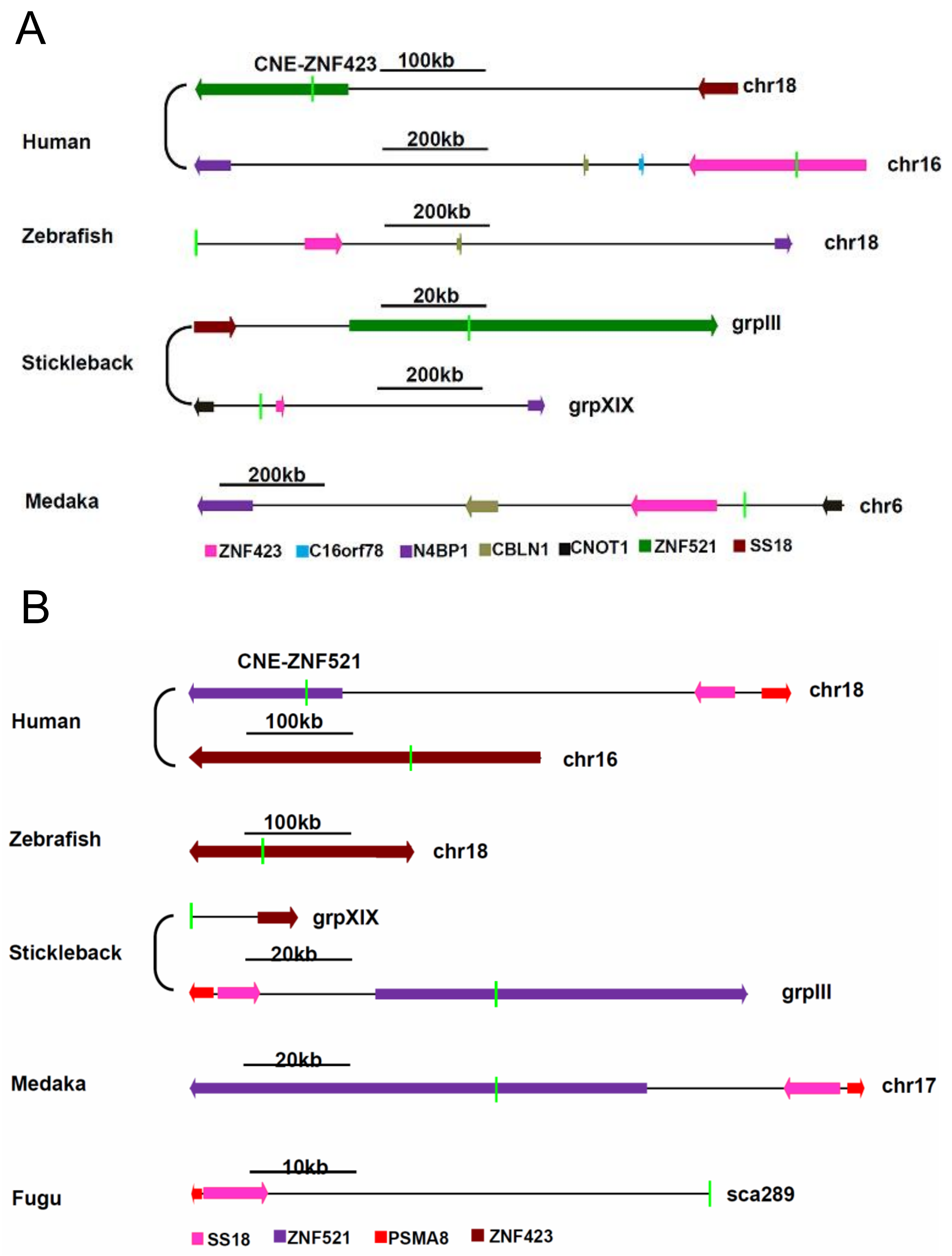
A
CNE-ZNF423
100kb
chr18
Human
200kb
chr16
Zebrafish
200kb
chr18
Stickleback
20kb
grpIII
200kb
grpXIX
Medaka
200kb
chr6
ZNF423
C16orf78
N4BP1
CBLN1
CNOT1
ZNF521
SS18
B
CNE-ZNF521
Human
chr18
100kb
chr16
Zebrafish
100kb
chr18
Stickleback
grpXIX
20kb
grpIII
Medaka
20kb
chr17
Fugu
10kb
sca289
SS18
ZNF521
PSMA8
ZNF423

Figure 46. Human CNE-enhancers by tracing the genic context of their orthologous copies in teleost fish lineage (hs625 /hs369).

Systematic analysis of the surrounding region of CNE enhancers, conserved across the tetrapod-teleost lineages, carved a pathway to associate the CNE enhancer to its target genes. **(A)** Duplication is occurred in both human and fish lineages. Careful analysis of genic context of orthologous regions of this element in teleost fish and its duplicated paralogous copy in human and fishes suggests the association of this enhancer with *ZNF423*. **(B)** Duplication is occurred in both human and fish lineages. Careful analysis of genic context of orthologous regions of this element in teleost fish and its duplicated paralogous copy in human and fishes suggests the association of this enhancer with *ZNF521*.

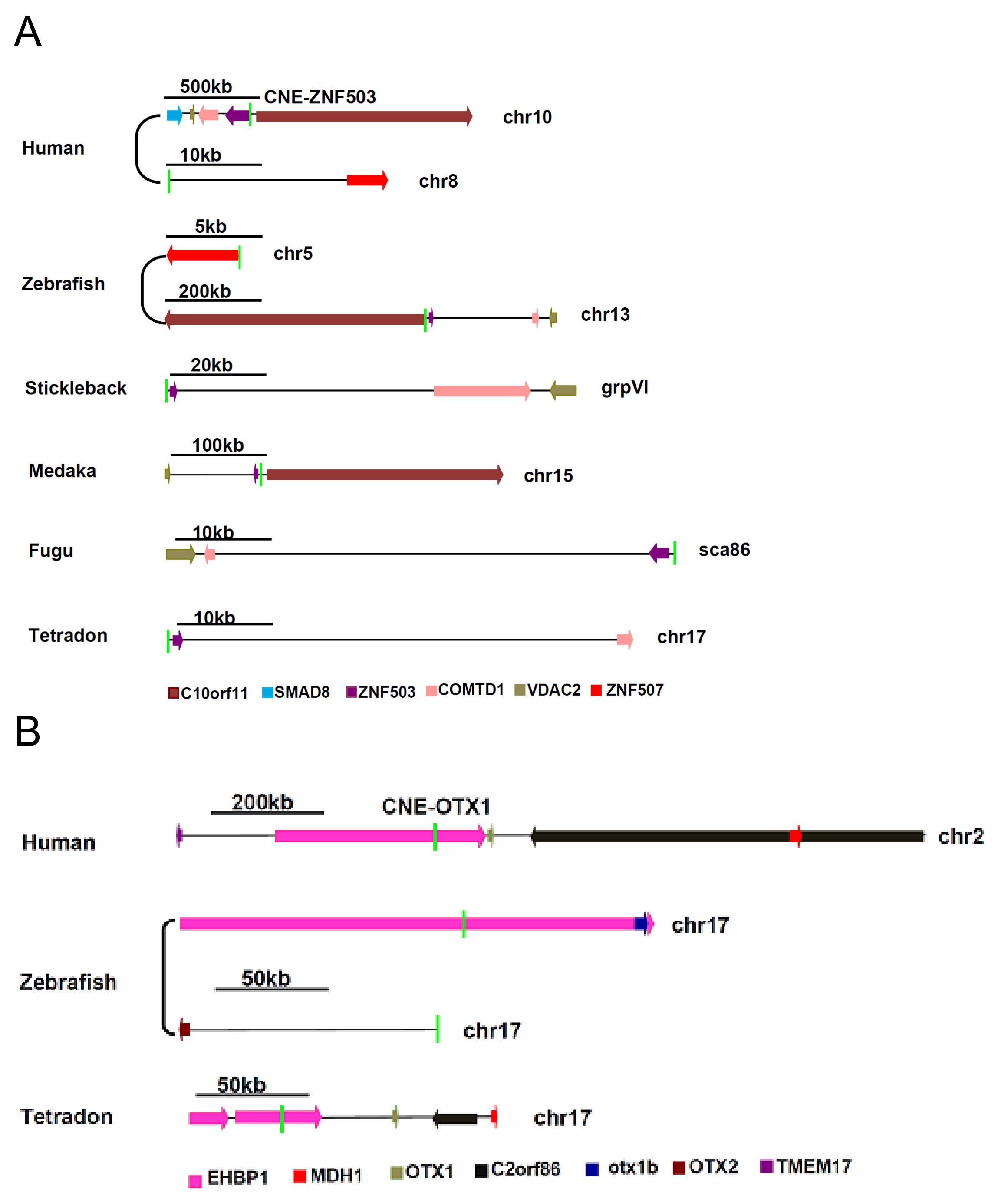
A
Human
500kb
CNE-ZNF503
chr10
10kb
chr8
Zebrafish
5kb
chr5
200kb
chr13
Stickleback
20kb
grpVI
Medaka
100kb
chr15
Fugu
10kb
sca86
Tetradon
10kb
chr17
C10orf11
SMAD8
ZNF503
COMTD1
VDAC2
ZNF507
B
Human
200kb
CNE-OTX1
chr2
Zebrafish
chr17
50kb
chr17
Tetradon
50kb
chr17
EHBP1
MDH1
OTX1
C2orf86
otx1b
OTX2
TMEM17

Figure 47. Predicting the target genes of human enhancers by analyzing their genic environment within and across vertebrate genomes (hs320/hs690).

Gene, controlled by specific regulatory elements, are identified through the systematic analysis of orthologous genomic content, which harbors functionally identified CNE enhancers, of tetrapod-teleost lineages. **(A)** Analysis of expression pattern and conservation in tetrapod and fish lineages shows that CNE-enhancer is regulating the expression of *ZNF503*. Conservation of duplicated copy of this gene along with the CNE in fishes verifies this suggestion. **(B)** Duplication is occurred in only in teleost fishes , and the gene entangled with the CNE, in duplicated copy, is *OTX1*. Genic architecture analysis of the orthologous and paralogous loci holding the CNE enhancers in vertebrate genome recommends the association of CNE with *OTX1*.

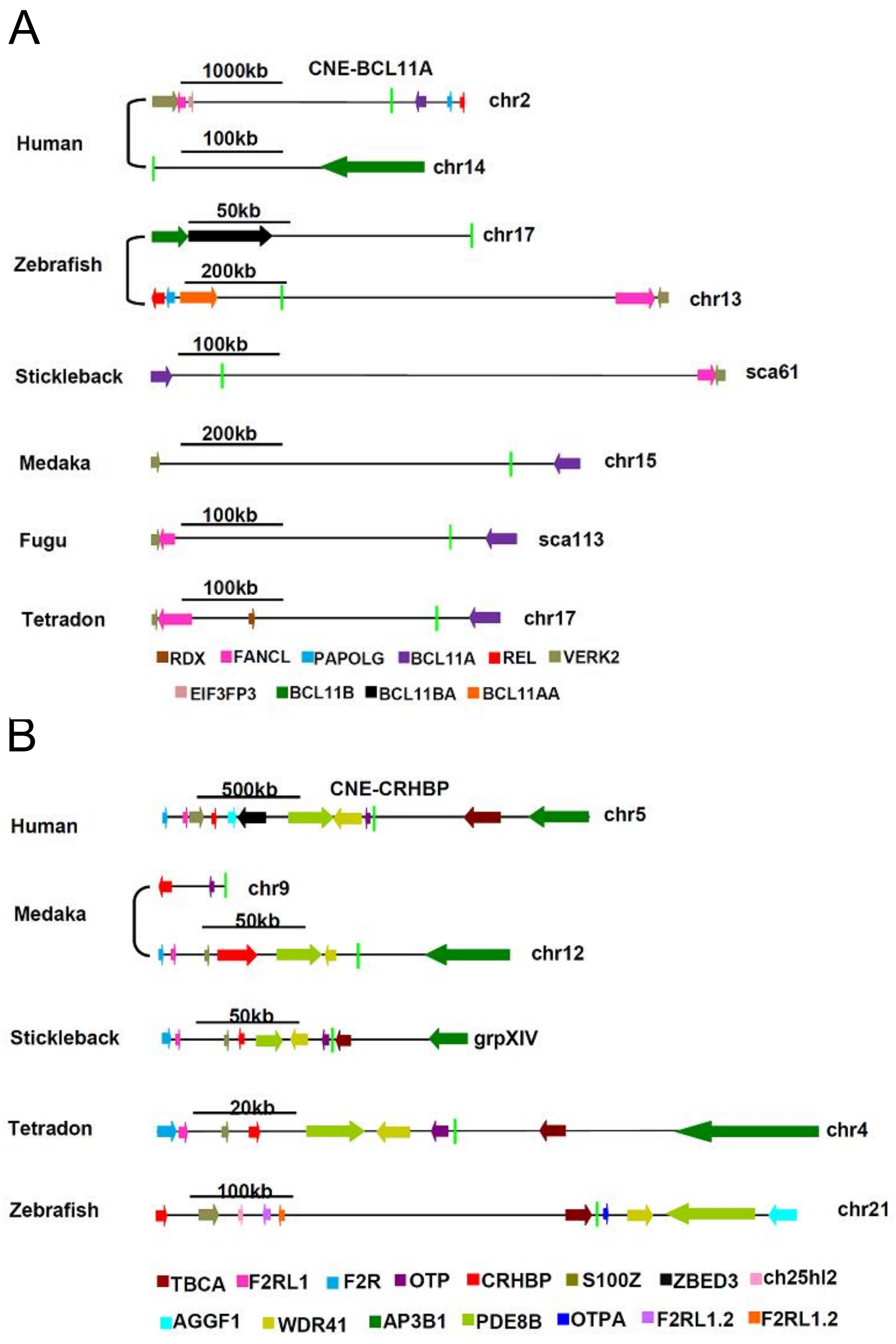
A
1000kb
CNE-BCL11A
chr2
Human
100kb
chr14
50kb
chr17
Zebrafish
200kb
chr13
100kb
Stickleback
sca61
200kb
Medaka
chr15
100kb
Fugu
sca113
100kb
Tetradon
chr17
RDX
FANCL
PAPOLG
BCL11A
REL
VERK2
EIF3FP3
BCL11B
BCL11BA
BCL11AA
B
500kb
CNE-CRHBP
Human
chr5
Medaka
chr9
50kb
chr12
50kb
Stickleback
grpXIV
20kb
Tetradon
chr4
100kb
Zebrafish
chr21
TBCA
F2RL1
F2R
OTP
CRHBP
S100Z
ZBED3
ch25hl2
AGGF1
WDR41
AP3B1
PDE8B
OTPA
F2RL1.2
F2RL1.2

Figure 48. Assigning the target genes to CNE-enhancers through comparative genomic analysis (hs399/hs262).

Through Comparative genomic analysis of functionally defined CNE enhancers, across tetrapod-teleost lineages, cis regulatory networks of human genes are defined. **(A)** Duplication is occurred in both human and fish lineages. Careful analysis of genic context of orthologous regions of this element in teleost fish and its duplicated paralogous copy in human and fishes suggests the association of this enhancer with *BCL11A*. **(B)** Duplication is occurred in fishes. Careful analysis of genic context of orthologous regions of this element in teleost fish and its duplicated paralogous copy in human and fishes suggests the association of this enhancer with *CRHBP.*

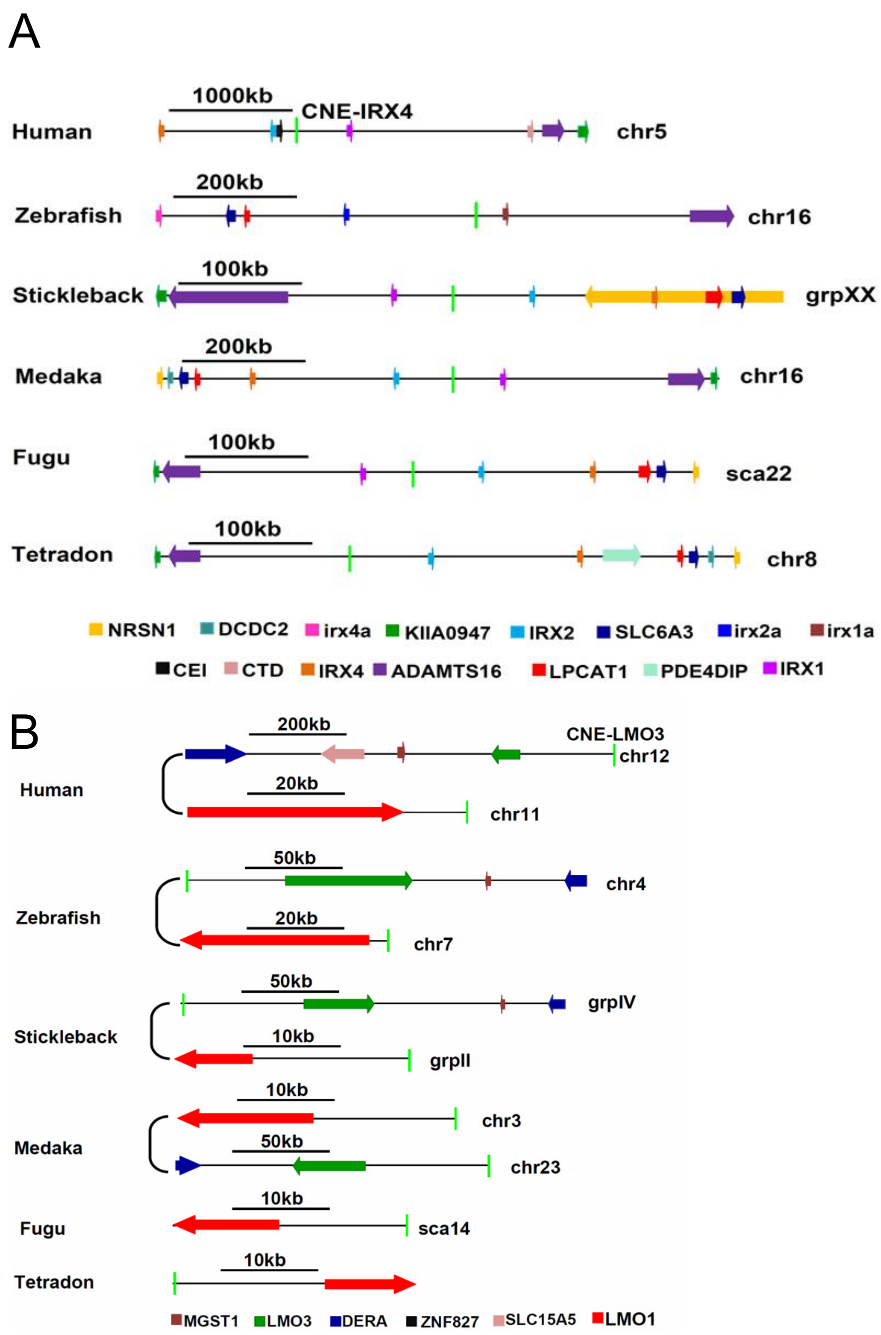
A
1000kb
CNE-IRX4
Human
chr5
200kb
Zebrafish
chr16
100kb
Stickleback
grpXX
200kb
Medaka
chr16
100kb
Fugu
sca22
100kb
Tetradon
chr8
NRSN1
DCDC2
irx4a
KIIA0947
IRX2
SLC6A3
irx2a
irx1a
CEI
CTD
IRX4
ADAMTS16
LPCAT1
PDE4DIP
IRX1
B
200kb
CNE-LMO3
chr12
Human
20kb
chr11
50kb
chr4
Zebrafish
20kb
chr7
50kb
grpIV
Stickleback
10kb
grpII
10kb
chr3
Medaka
50kb
chr23
10kb
Fugu
sca14
10kb
Tetradon
MGST1
LMO3
DERA
ZNF827
SLC15A5
LMO1

Figure 49. Predicting the target genes of human enhancers by analyzing their genic environment within and across vertebrate genomes (hs754/hs661).

Gene, controlled by specific regulatory elements, are identified through the systematic analysis of orthologous genomic content, which harbors functionally identified CNE enhancers, of tetrapod-teleost lineages. **(A)** Analysis of expression pattern and conservation in tetrapod and fish lineages shows that CNE-enhancer is regulating the expression of *IRX4*. Conservation of duplicated copy of this gene along with the CNE in fishes verifies this suggestion. **(B)** Duplication is occurred in both human and teleost fishes , and the gene entangled with the CNE, in duplicated copy, is *LMO3*. Genic architecture analysis of the orthologous and paralogous loci holding the CNE enhancers in vertebrate genome recommends the association of CNE with *LMO3*.

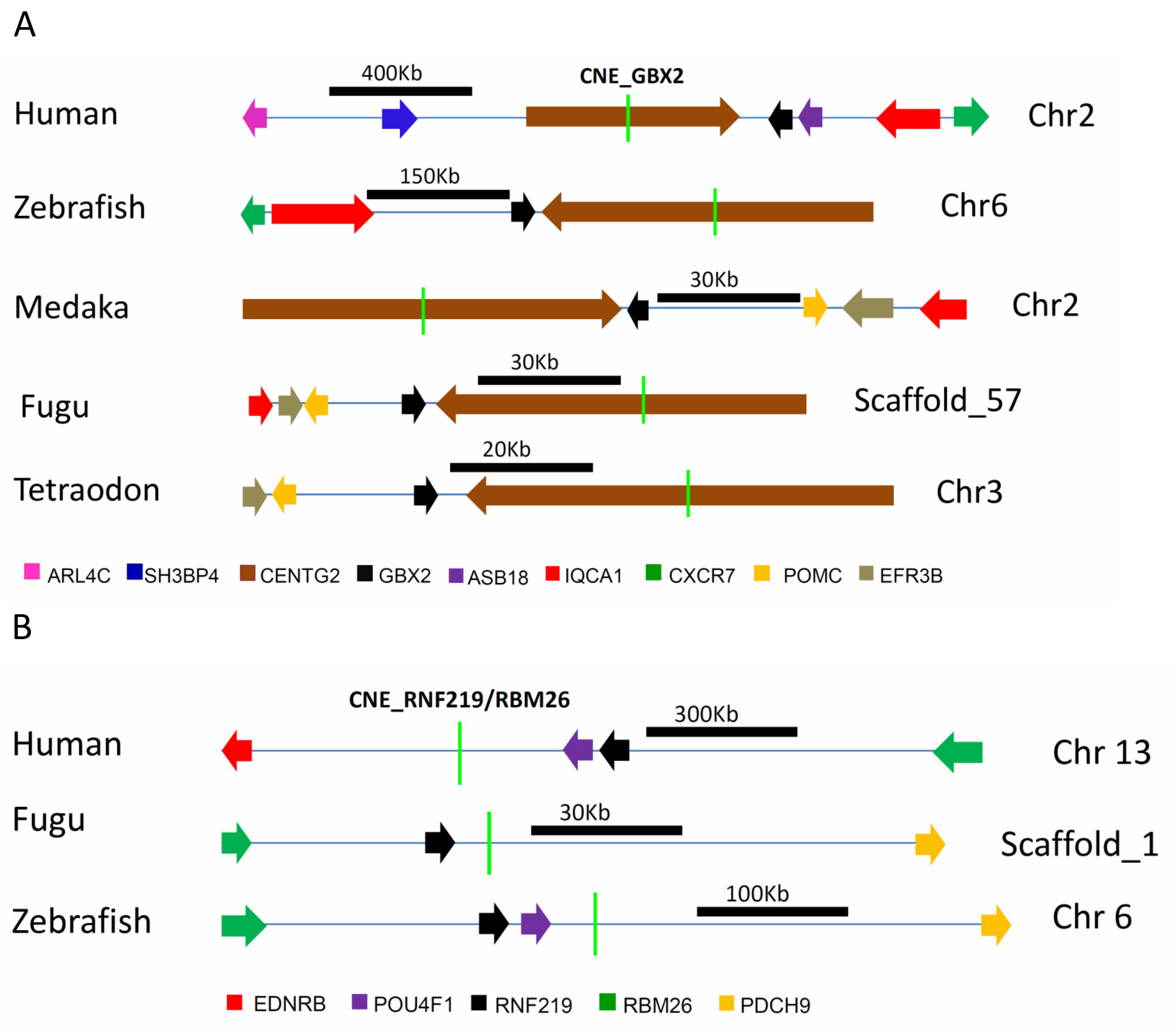
A
400Kb
CNE_GBX2
Human
Chr2
150Kb
Zebrafish
Chr6
30Kb
Medaka
Chr2
30Kb
Fugu
Scaffold_57
20Kb
Tetraodon
Chr3
ARL4C
SH3BP4
CENTG2
GBX2
ASB18
IQCA1
CXCR7
POMC
EFR3B
B
CNE_RNF219/RBM26
300Kb
Human
Chr 13
Fugu
30Kb
Scaffold_1
100Kb
Zebrafish
Chr 6
EDNRB
POU4F1
RNF219
RBM26
PDCH9

Figure 50 . Predicting the target genes of human enhancers by analyzing their genic environment within and across vertebrate genomes (hs921/hs915).

(A) CNE-enhancer within intron of human *CENTG2* gene is associated with neighboring *GBX2*. **(B)** A human CNE-enhancer positioned in the intergenic space between human *EDNRB* and *POU4F1* is associated with *RNF219* and *RBM26* according to the endogenous expression pattern analysis of genes and CNE-induced reporter gene expression.

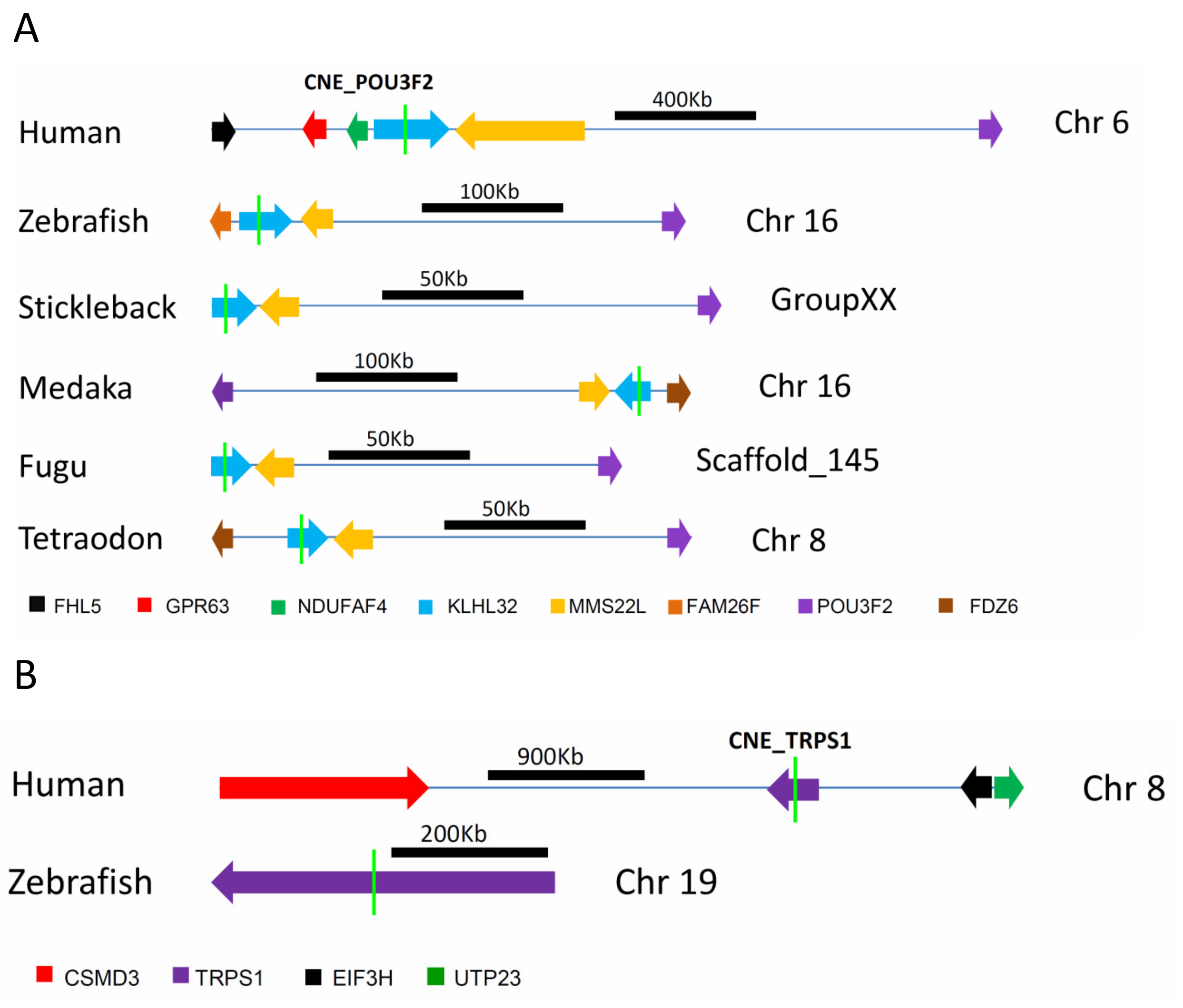
A
CNE_POU3F2
Human
400Kb
Chr 6
Zebrafish
100Kb
Chr 16
Stickleback
50Kb
GroupXX
Medaka
100Kb
Chr 16
Fugu
50Kb
Scaffold_145
Tetraodon
50Kb
Chr 8
FHL5
GPR63
NDUFAF4
KLHL32
MMS22L
FAM26F
POU3F2
FDZ6
B
Human
900Kb
CNE_TRPS1
Chr 8
200Kb
Zebrafish
Chr 19
CSMD3
TRPS1
EIF3H
UTP23

Figure 51 . Analyzing the genic environment of human CNE-enhancers in teleost fish orthologous loci and expression pattern of reporter gene helps in identifying their target genes (hs676/hs919).

(A) The physical location of human CNE-enhancer within the intronic region of *KIAA1900 (KLHL32*) is conserved in its orthologous intervals in fish lineages, along with other bystander genes. However the endogenous expression pattern of *KIAA1900* and *MMS22L* matches with that of reporter gene expression, clearly inferring the association of enhancer with *KIAA1900* and *MMS22L*. **(B)** Analyzing the expression pattern and conservation in fish lineage shows that an intragenic CNE-enhancer is regulating the expression of *TRPS1* gene.

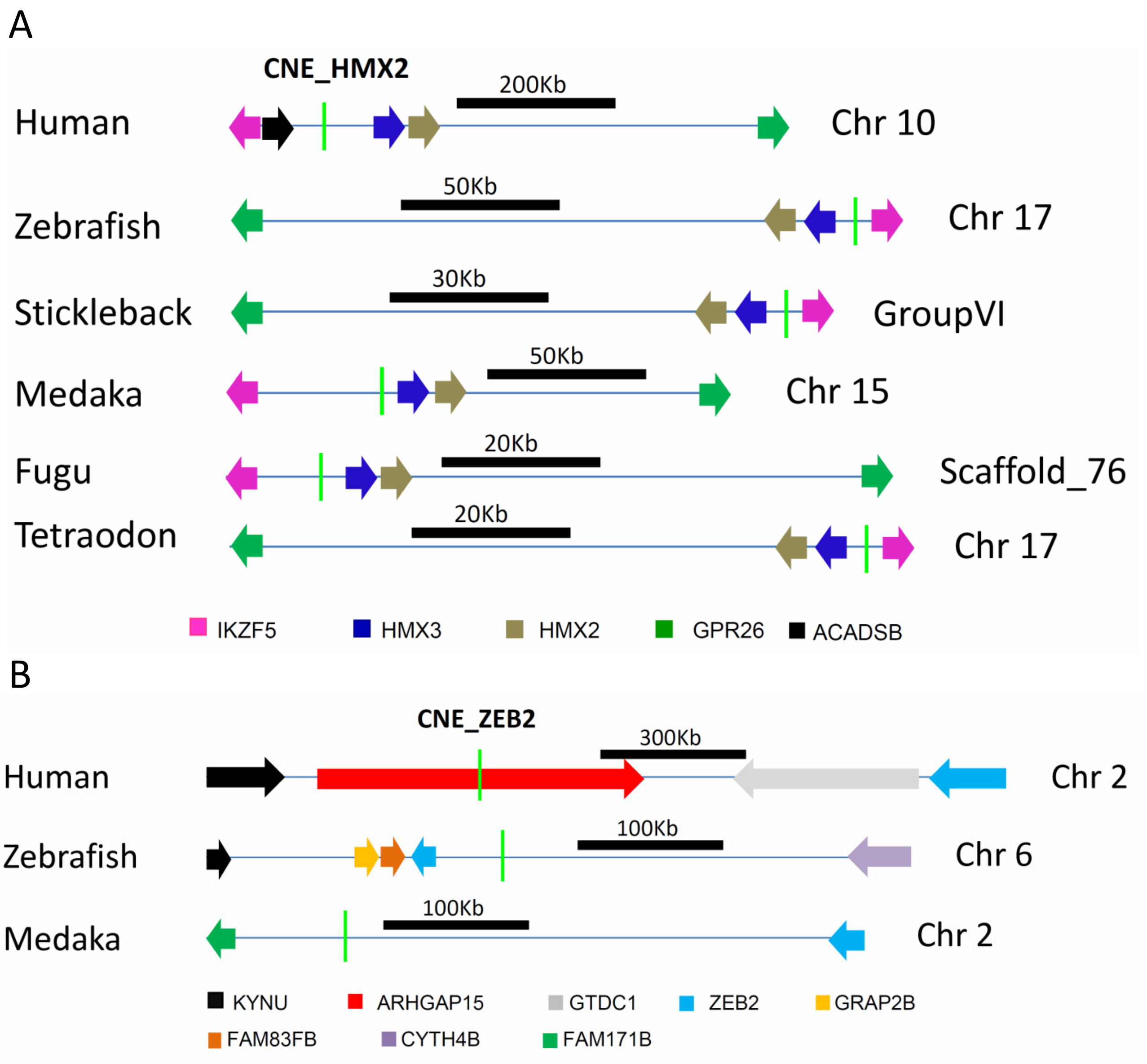
A
CNE_HMX2
200Kb
Human
Chr 10
50Kb
Zebrafish
Chr 17
30Kb
Stickleback
GroupVI
50Kb
Medaka
Chr 15
20Kb
Fugu
Scaffold_76
20Kb
Tetraodon
Chr 17
IKZF5
HMX3
HMX2
GPR26
ACADSB
B
CNE_ZEB2
300Kb
Human
Chr 2
100Kb
Zebrafish
Chr 6
100Kb
Medaka
Chr 2
KYNU
ARHGAP15
GTDC1
ZEB2
GRAP2B
FAM83FB
CYTH4B
FAM171B

Figure 52. Comparative analysis helps in assigning the CNE-enhancer to its target genes (hs329/hs675).

(A) A human CNE-enhancer positioned in the intergenic space between human *ACADSB* and *HMX3* is associated with *HMX2* according to the endogenous expression pattern analysis of genes and CNE-induced reporter gene expression. **(B)** CNE-enhancer within an intron of human *ARHGAP15* gene is associated with neighboring *ZEB2* gene. Syntenic and expression pattern analysis supports this association.

A

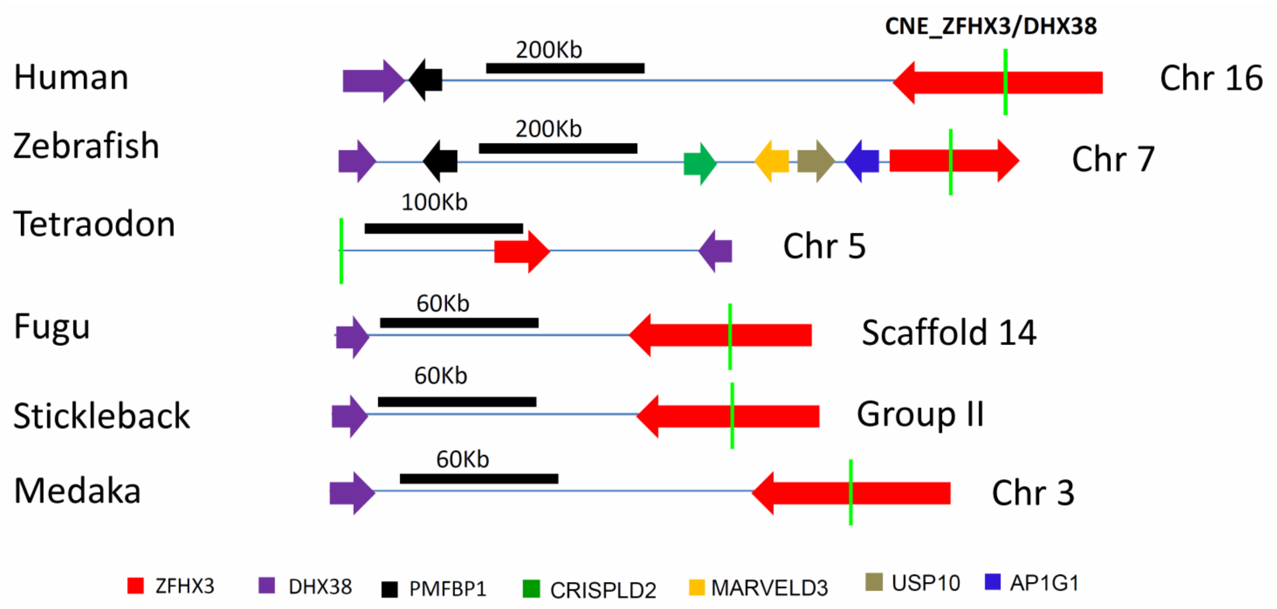
CNE_ZFHX3/DHX38
Human
200Kb
Chr 16
Zebrafish
200Kb
Chr 7
Tetraodon
100Kb
Chr 5
Fugu
60Kb
Scaffold 14
Stickleback
60Kb
Group II
Medaka
60Kb
Chr 3
ZFHX3
DHX38
PMFBP1
CRISPLD2
MARVELD3
USP10
AP1G1

B

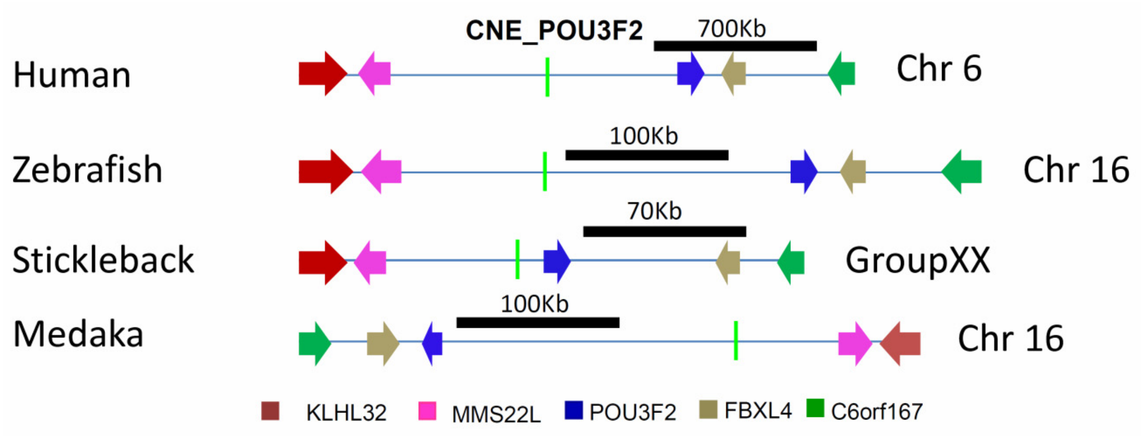
CNE_POU3F2
700Kb
Human
Chr 6
Zebrafish
100Kb
Chr 16
Stickleback
70Kb
GroupXX
Medaka
100Kb
Chr 16
KLHL32
MMS22L
POU3F2
FBXL4
C6orf167

Figure 53 . Target gene identification of human CNE-enhancers through orthology mapping (hs16/ hs282).

(A) Conserved localization of CNE within the intronic region of *ZFHX3*, across the vertebrate lineage (except Tetraodon) associates this CNE-enhancer with its host gene. Analysis of expression pattern of genes and CNE-induced reporter gene expression along with syntenic analysis also associates this CNE with *DHX38* gene. **(B)** A human CNE, present within the Intergenic region between *MMS22L* and *POU3F2* genes, is associated with *POU3F2* genes. Expression pattern of *MMS22L* is not matched with CNE-induced reporter gene expression.

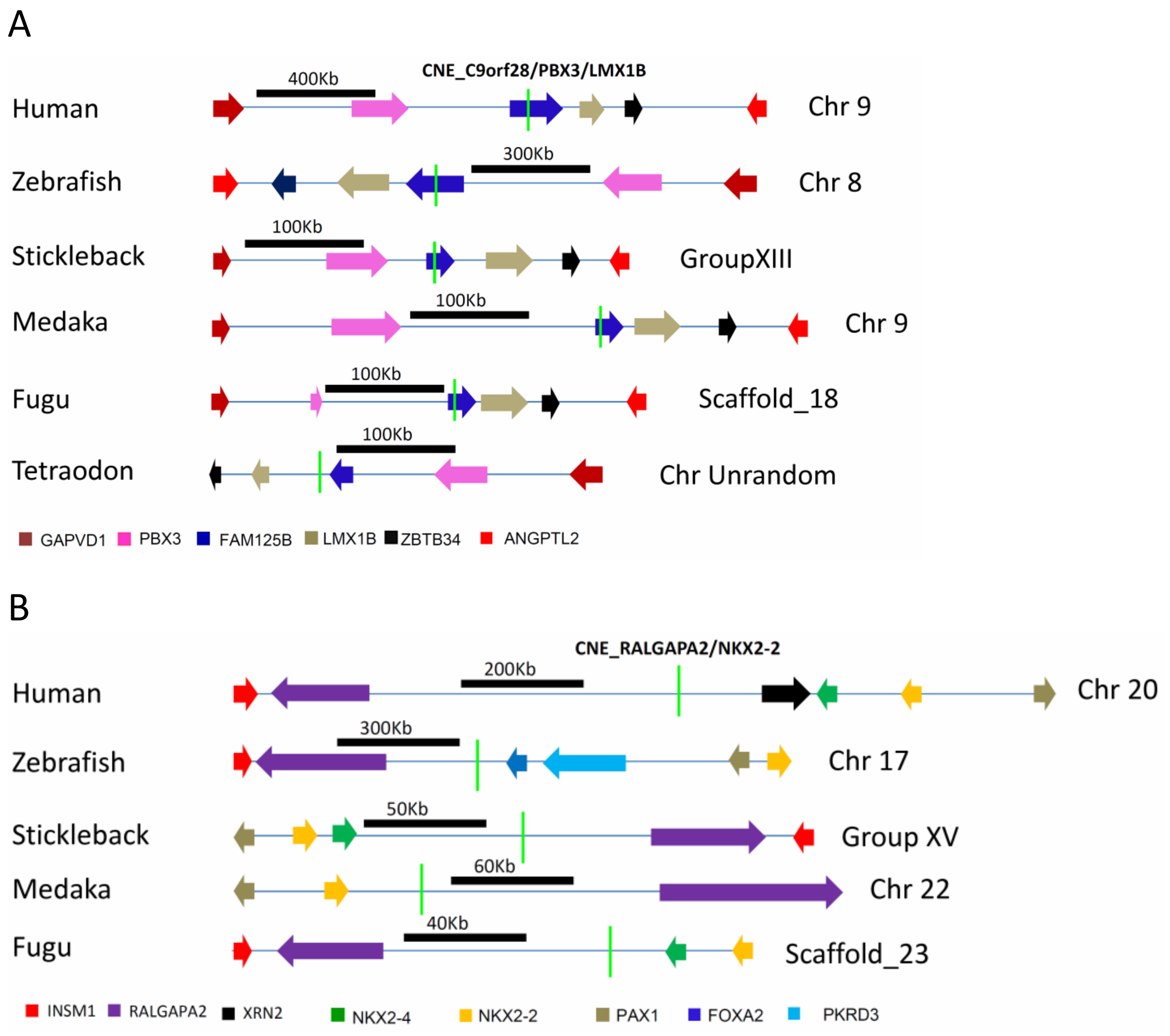
A
CNE_C9orf28/PBX3/LMX1B
400Kb
Human
Chr 9
300Kb
Zebrafish
Chr 8
100Kb
Stickleback
GroupXIII
100Kb
Medaka
Chr 9
100Kb
Fugu
Scaffold_18
100Kb
Tetraodon
Chr Unrandom
GAPVD1 PBX3 FAM125B LMX1B ZBTB34 ANGPTL2
B
CNE_RALGAPA2/NKX2-2
200Kb
Human
Chr 20
300Kb
Zebrafish
Chr 17
50Kb
Stickleback
Group XV
60Kb
Medaka
Chr 22
40Kb
Fugu
Scaffold_23
INSM1 RALGAPA2 XRN2 NKX2-4 NKX2-2 PAX1 FOXA2 PKRD3

Figure 54 . Analysis of genic environment of human CNE-enhancers in teleost fish orthologous genomic intervals helps in identifying their target genes (hs186/ hs914).

(A) An intragenic CNE is associated with its harboring gene *C9orf28* (*FAM125B*). Synteny and expression pattern analysis also associate this CNE with *PBX3* and *LMX1B* genes. **(B)** A human CNE is present between *RALGAPA2* and *XRN2*. Comparative syntenic analysis reveals that *XRN2* is lost in fish lineage. Expression pattern analysis and interspecies conservation supports the association of this CNE-enhancer with *RALGAPA2* and *NKX2-2* genes.

A

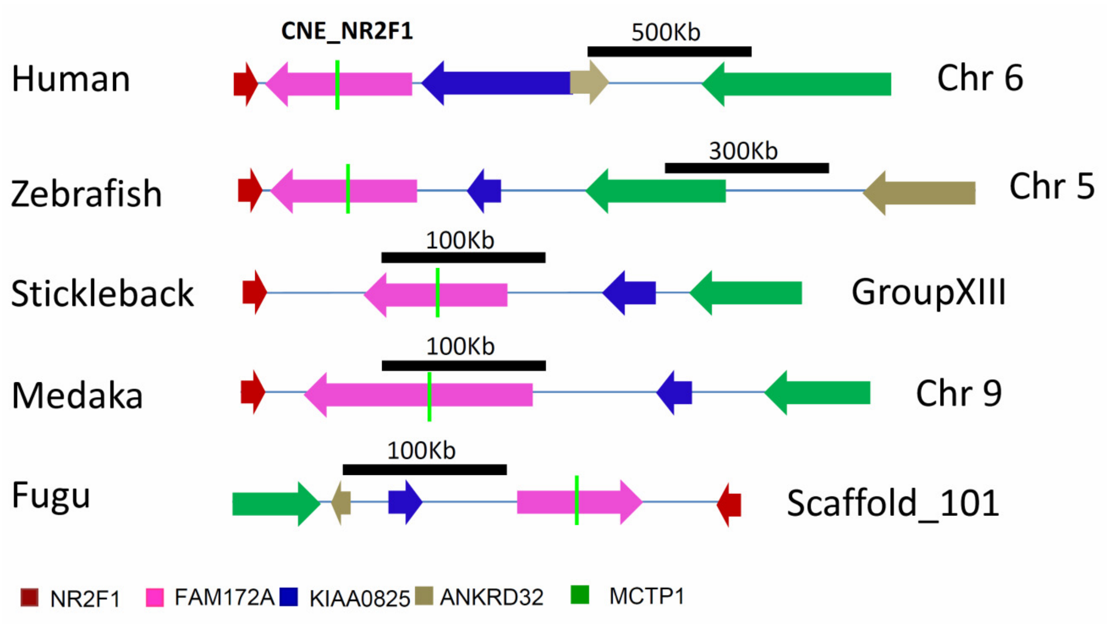

CNE_NR2F1
500Kb
Human
Chr 6
300Kb
Zebrafish
Chr 5
100Kb
Stickleback
GroupXIII
100Kb
Medaka
Chr 9
100Kb
Fugu
Scaffold_101
NR2F1
FAM172A
KIAA0825
ANKRD32
MCTP1

Figure 55. Assigning the target genes to CNE-enhancers through comparative genomic analysis (hs271).

(A) CNE-enhancer within an intron of human *FAM172A* gene is associated with neighboring *NR2F1* gene according to the expression pattern analysis.

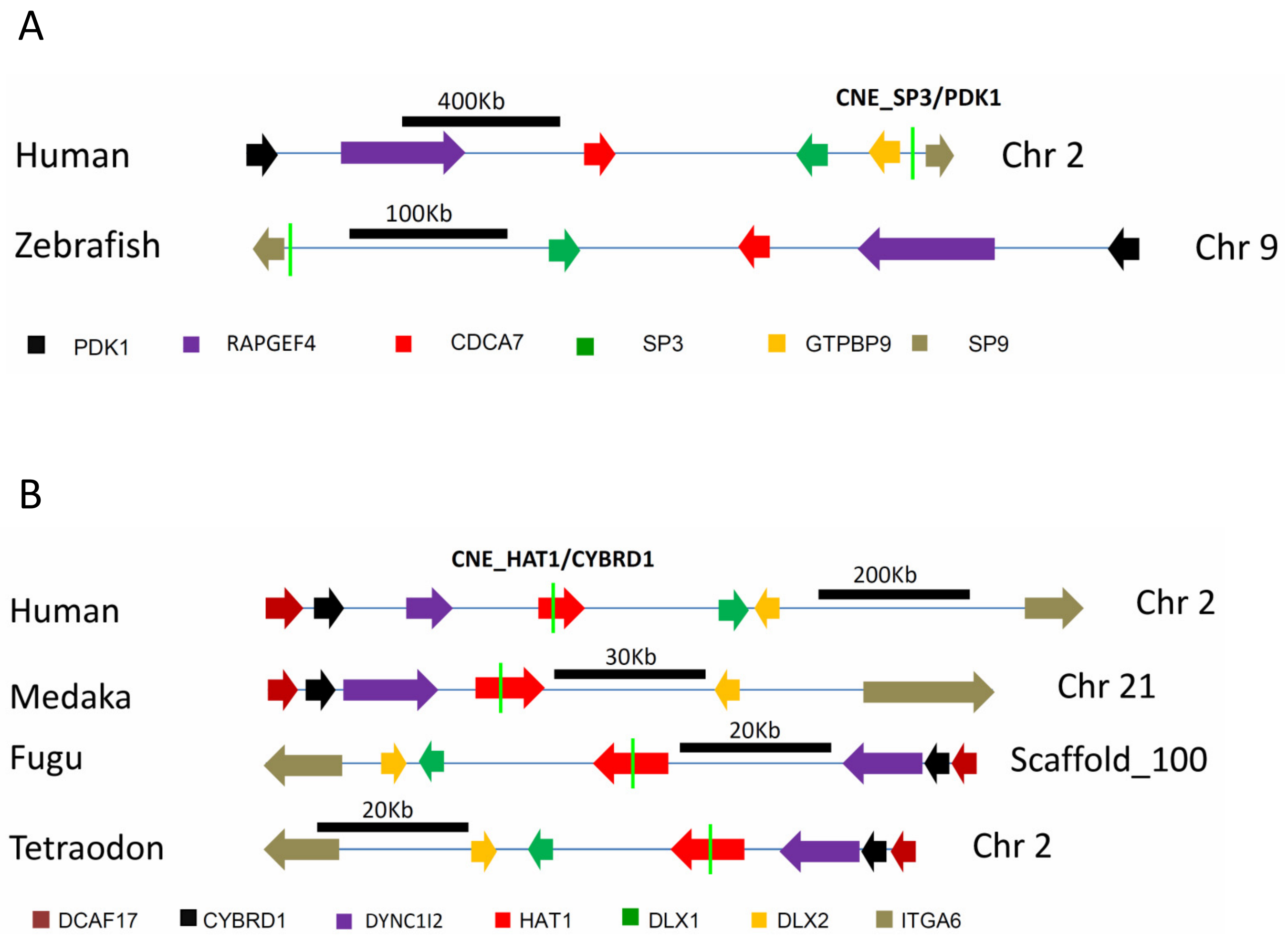
A
400Kb
CNE_SP3/PDK1
Human
Chr 2
100Kb
Zebrafish
Chr 9
PDK1
RAPGEF4
CDCA7
SP3
GTPBP9
SP9
B
CNE_HAT1/CYBRD1
Human
200Kb
Chr 2
Medaka
30Kb
Chr 21
Fugu
20Kb
Scaffold_100
20Kb
Tetraodon
Chr 2
DCAF17
CYBRD1
DYNC1I2
HAT1
DLX1
DLX2
ITGA6

Figure 56 . Assigning the target genes to CNE-enhancers through comparative genomic analysis (hs860/ hs646).

(A) A human CNE is present within intergenic region of *GTPBP9* and *SP9* genes. According to the expression pattern analysis and interspecies genic conservation, this CNE is associated with *SP3* and *PDK1* genes. **(B)** An intragenic CNE is associated with its harboring gene *HAT1*. Syntenic and expression pattern analysis also associate this CNE with *CYBRD1* gene.

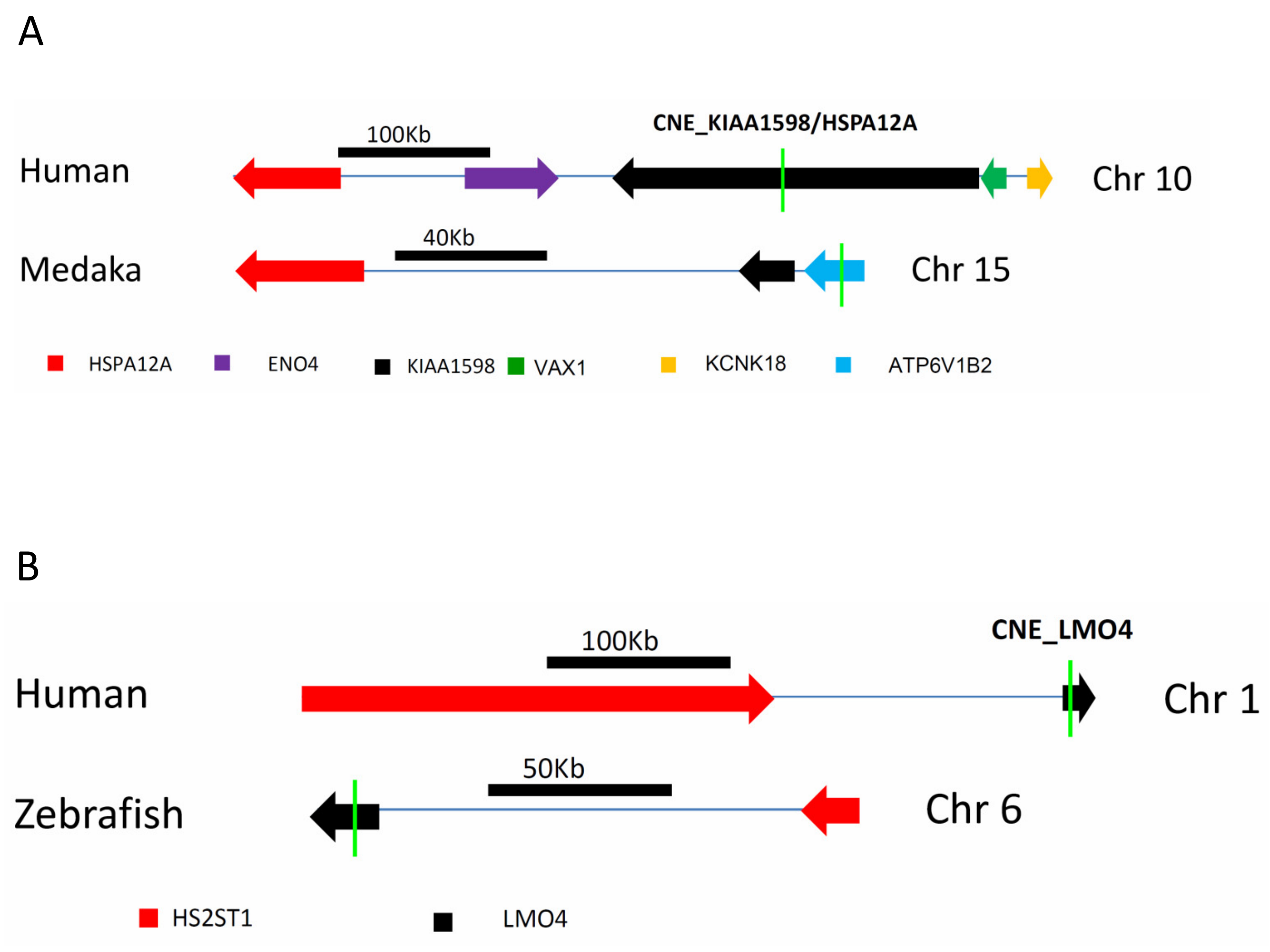
A
100Kb
CNE_KIAA1598/HSPA12A
Human
Chr 10
40Kb
Medaka
Chr 15
HSPA12A
ENO4
KIAA1598
VAX1
KCNK18
ATP6V1B2
B
CNE_LMO4
100Kb
Human
Chr 1
50Kb
Zebrafish
Chr 6
HS2ST1
LMO4

Figure 57 . Analyzing the genic architecture of human (hs841/hs809).

(A) A CNE is present within intron of *KIAA1598* gene. This CNE is associated with *KIAA1598* and *HSPA12A* genes. **(B)** Conserved intragenic position of CNE-enhancer and expression pattern analysis clearly associates this CNE with its harboring gene *LMO4*.

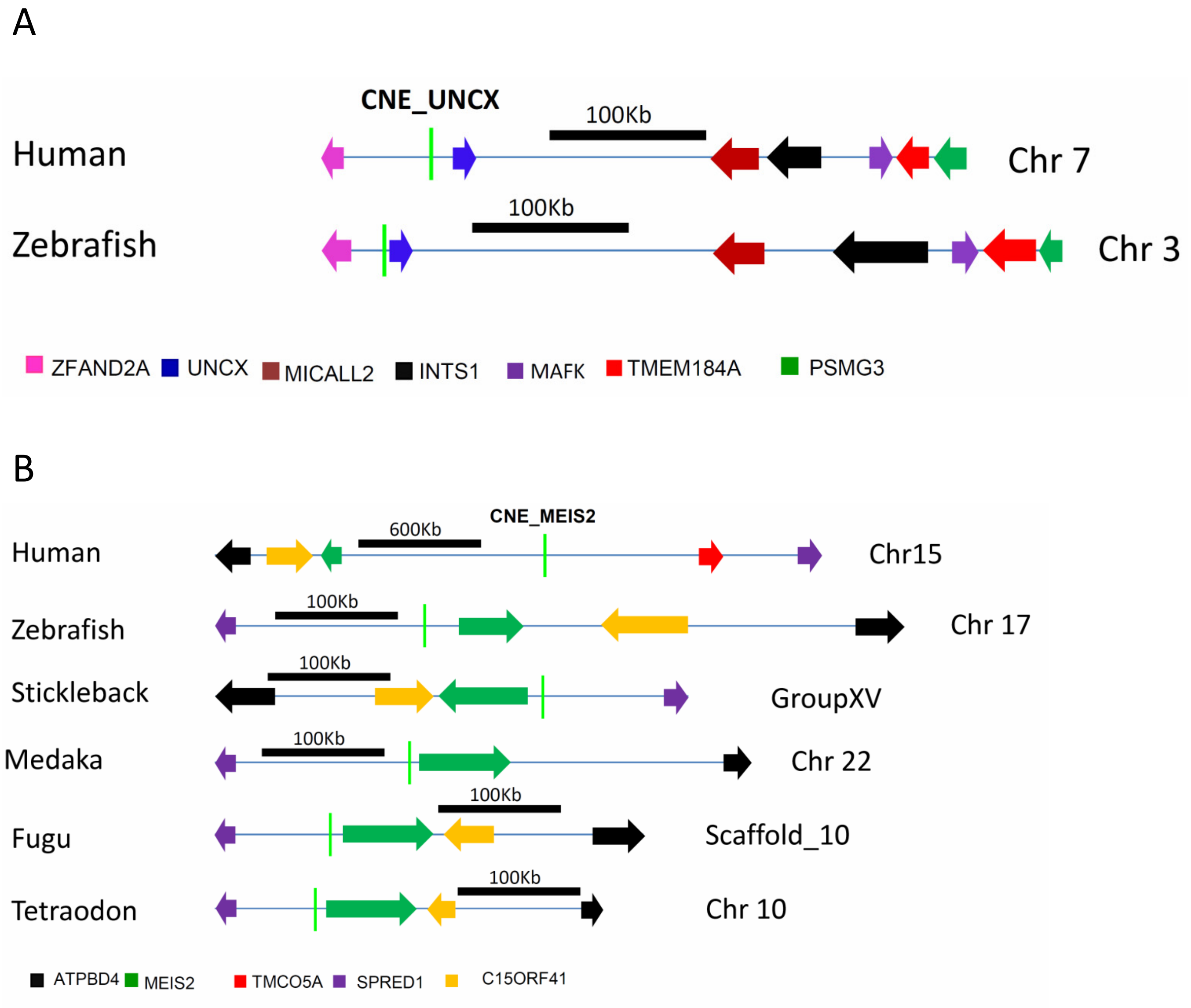
A
CNE_UNCX
Human
100Kb
Chr 7
Zebrafish
100Kb
Chr 3
ZFAND2A UNCX MICALL2 INTS1 MAFK TMEM184A PSMG3
B
CNE_MEIS2
Human
600Kb
Chr15
Zebrafish
100Kb
Chr 17
Stickleback
100Kb
GroupXV
Medaka
100Kb
Chr 22
Fugu
100Kb
Scaffold_10
Tetraodon
100Kb
Chr 10
ATPBD4 MEIS2 TMCO5A SPRED1 C15ORF41

Figure 58 . Analysis of human CNE-enhancers loci in teleost fish orthologous genomic intervals helps in identifying their target genes (hs293/hs572).

(A) A human CNE-enhancer is located within the intergenic region between *ZFAND2A* and *UNCX* genes. Endogenous expression pattern and CNE-induced reporter gene expression suggest the association between this CNE and *UNCX.* Expression pattern of *ZFAND2A* is unrelated to CNE expression pattern. **(B)** A CNE is positioned between *MEIS2* and *TMCO5* at human chromosome. Careful syntenic analysis shows that *TMCO5* is lost in teleost fish lineage, leaving the *MEIS2* best candidate for CNE association. Moreover, analysis of endogenous gene expression of *MEIS2* and CNE-induced reporter gene activity pattern clearly associates this CNE with *MEIS2*.

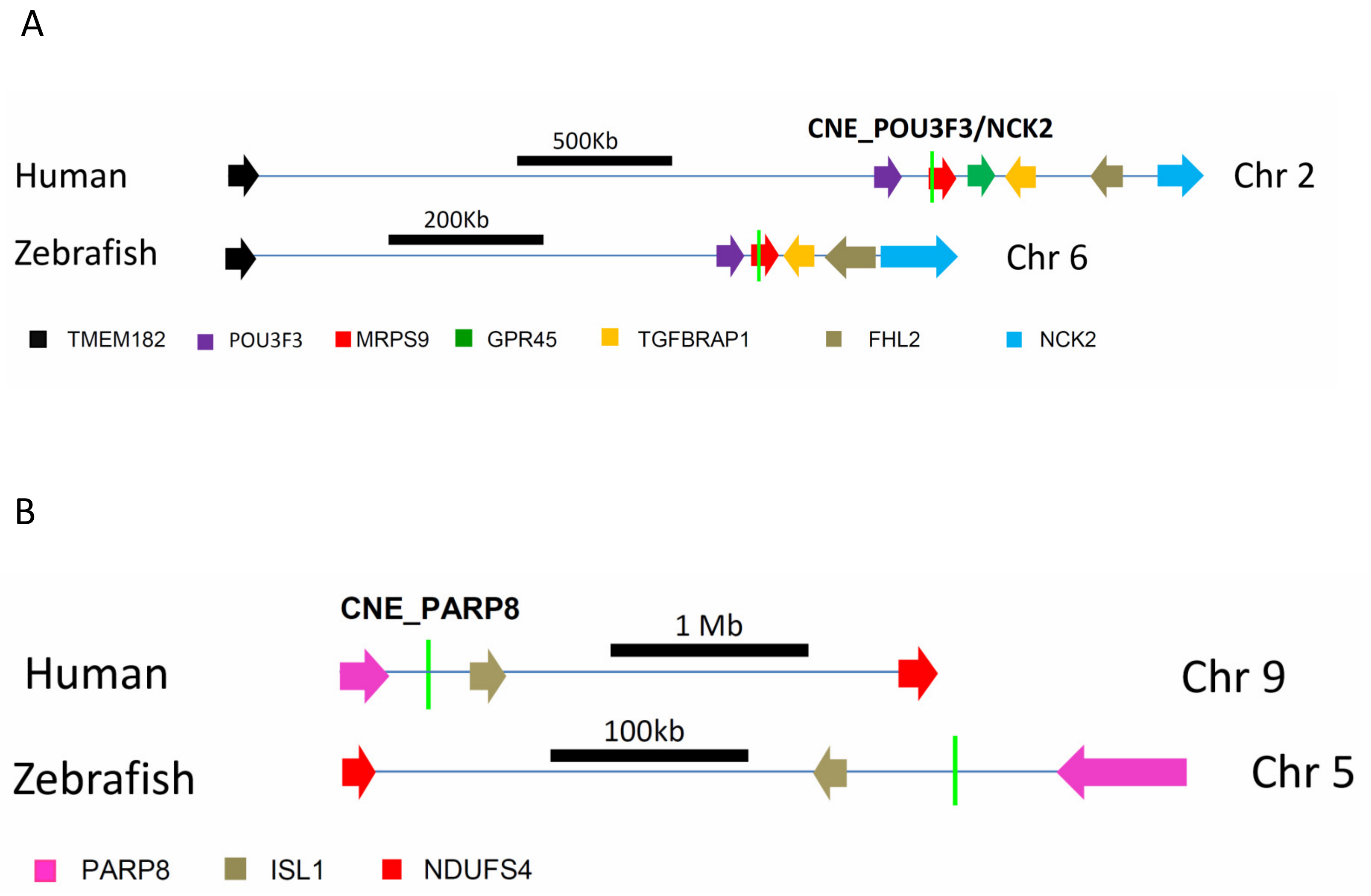
A
CNE_POU3F3/NCK2
500Kb
Human
Chr 2
200Kb
Zebrafish
Chr 6
TMEM182
POU3F3
MRPS9
GPR45
TGFBRAP1
FHL2
NCK2
B
CNE_PARP8
1 Mb
Human
Chr 9
100kb
Zebrafish
Chr 5
PARP8
ISL1
NDUFS4

Figure 59 . Human-fish comparative analysis of human CNEs (hs868/hs215).

(A) Conserved localization of human CNE within the intron of *MRPS9* gene, in human and teleost fish lineages, proposes the association between CNE and its harboring gene. But careful expression pattern analysis of both, the genes and CNE-enhancer suggests that this CNE is regulating the *POU3F3* and *NCK2* genes. **(B)** A human CNE is located within the intergenic region between *PARP8* and *ISL1* genes. As, expression pattern of *ISL1* is not in accordance with CNE-enhancer, this CNE is associated with *PARP8* gene.

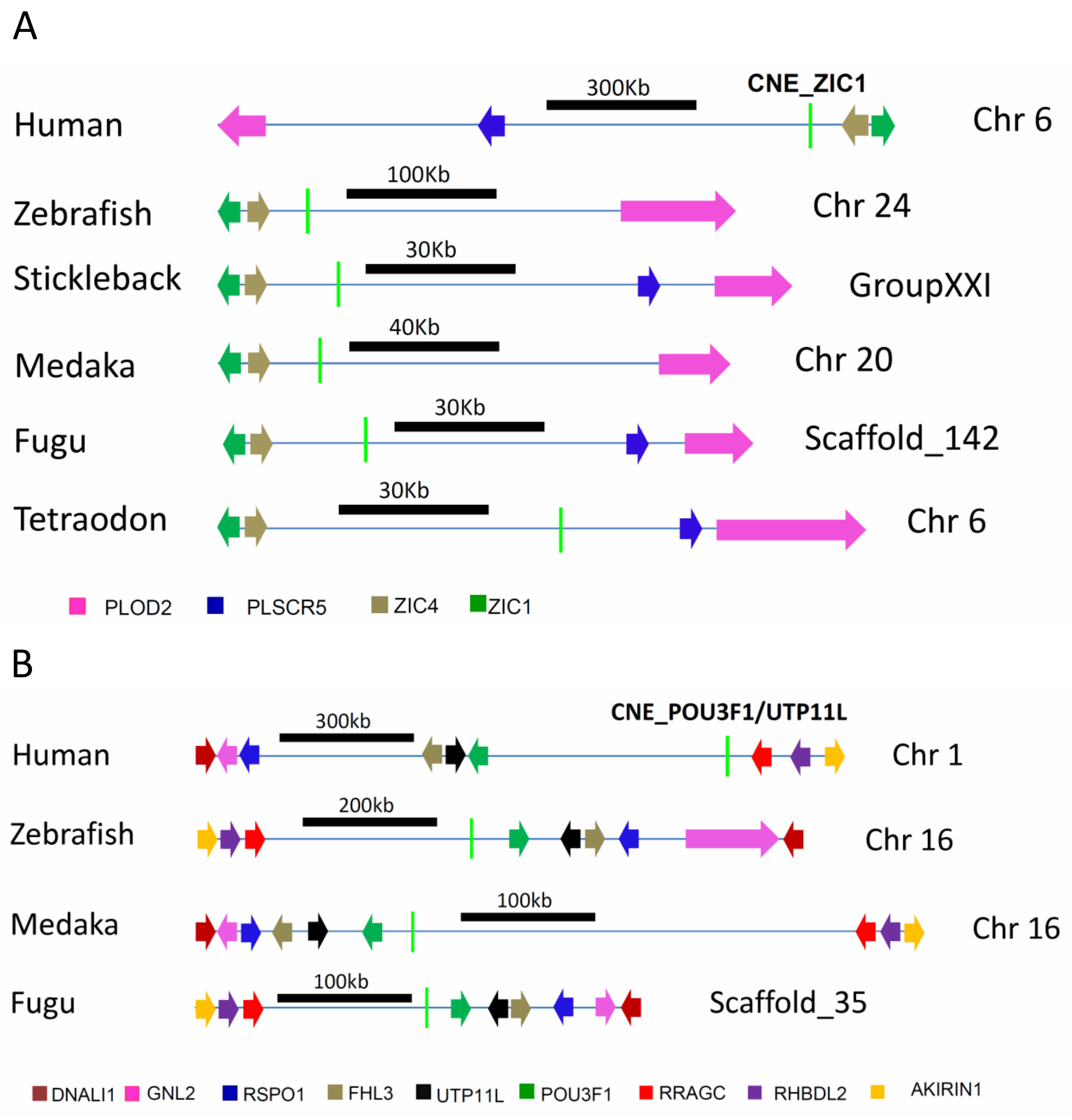
A
CNE_ZIC1
300Kb
Human
Chr 6
100Kb
Zebrafish
Chr 24
30Kb
Stickleback
GroupXXI
40Kb
Medaka
Chr 20
30Kb
Fugu
Scaffold_142
30Kb
Tetraodon
Chr 6
PLOD2
PLSCR5
ZIC4
ZIC1
B
CNE_POU3F1/UTP11L
300kb
Human
Chr 1
200kb
Zebrafish
Chr 16
100kb
Medaka
Chr 16
100kb
Fugu
Scaffold_35
DNALI1
GNL2
RSPO1
FHL3
UTP11L
POU3F1
RRAGC
RHBDL2
AKIRIN1

Figure 60 . Comparing the human-fish genic content and their reporter gene expression lead to association of CNE enhancers to their respective genes (hs213/hs238).

(A) Conserved positioning of human CNE between *PLSCR5* and *ZIC4* indicates that this CNE is associated with its flanking genes, but expression pattern supports *ZIC1* gene to be the target for this CNE. **(B)** A human CNE is associated with one of its flanking genes, *POU3F1*, and a neighboring gene *UTP11L*. The expression pattern of other flanking gene *RRAGC* is not in accordance with CNE-induced reporter gene expression.

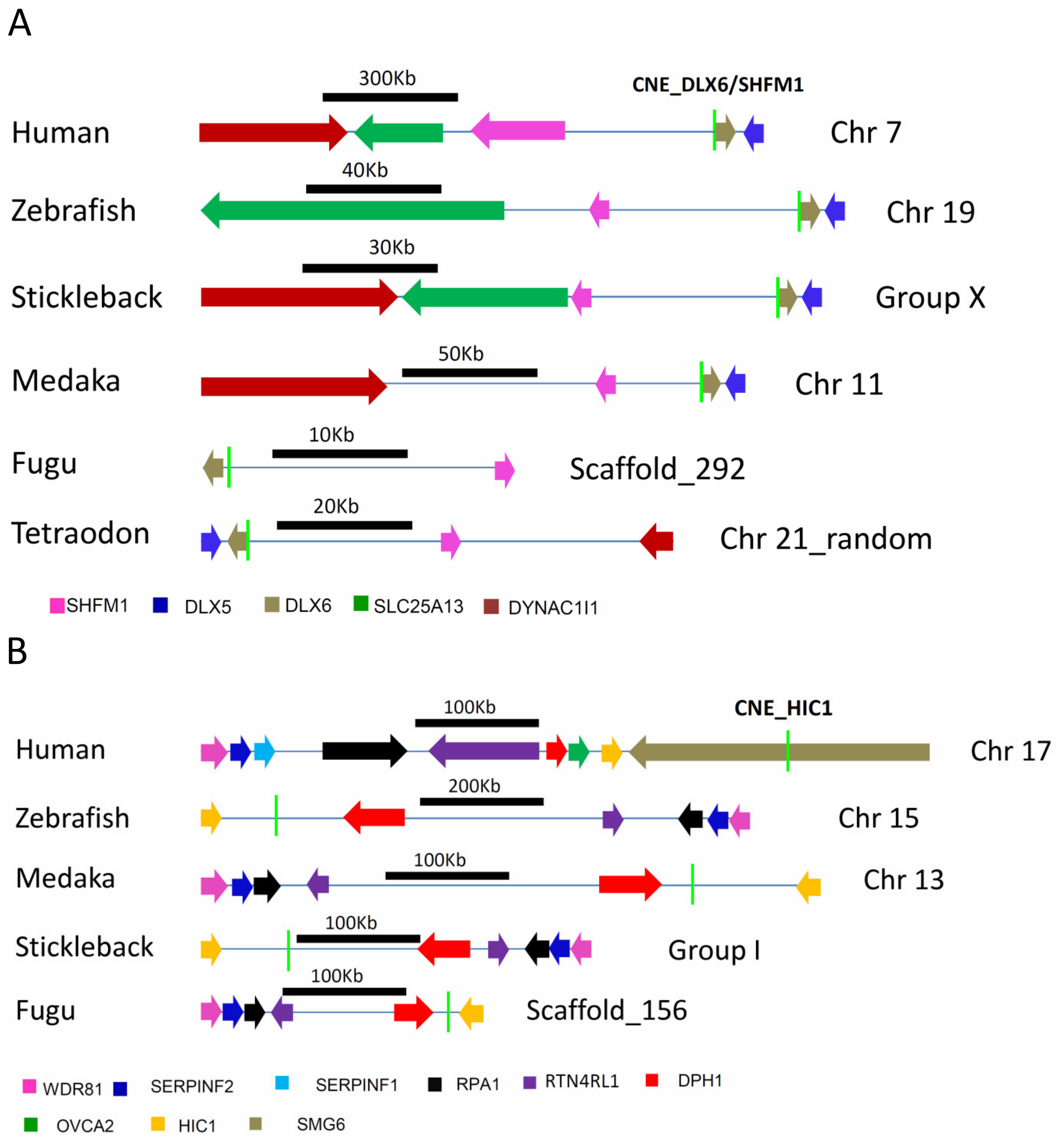
A
300Kb
CNE_DLX6/SHFM1
Human
Chr 7
40Kb
Zebrafish
Chr 19
30Kb
Stickleback
Group X
50Kb
Medaka
Chr 11
10Kb
Fugu
Scaffold_292
20Kb
Tetraodon
Chr 21_random
SHFM1
DLX5
DLX6
SLC25A13
DYNAC1I1
B
100Kb
CNE_HIC1
Human
Chr 17
200Kb
Zebrafish
Chr 15
100Kb
Medaka
Chr 13
100Kb
Stickleback
Group I
100Kb
Fugu
Scaffold_156
WDR81
SERPINF2
SERPINF1
RPA1
RTN4RL1
DPH1
OVCA2
HIC1
SMG6

Figure 61 . Analysis of genic environment of human CNE-enhancers in teleost fish orthologous genomic intervals and observing their expression patterns helps in identifying their target genes (hs298/ hs924).

(A) Conserved allotment of a human CNE between its flanking genes, *SHFM1* and *DLX6*, across human and teleost fish lineages, and endogenous expression pattern analysis clearly associates this CNE with its flanking genes. **(B)** A human CNE present within the intron of *SMG6* gene is associated with a neighbor gene *HIC1*.

A

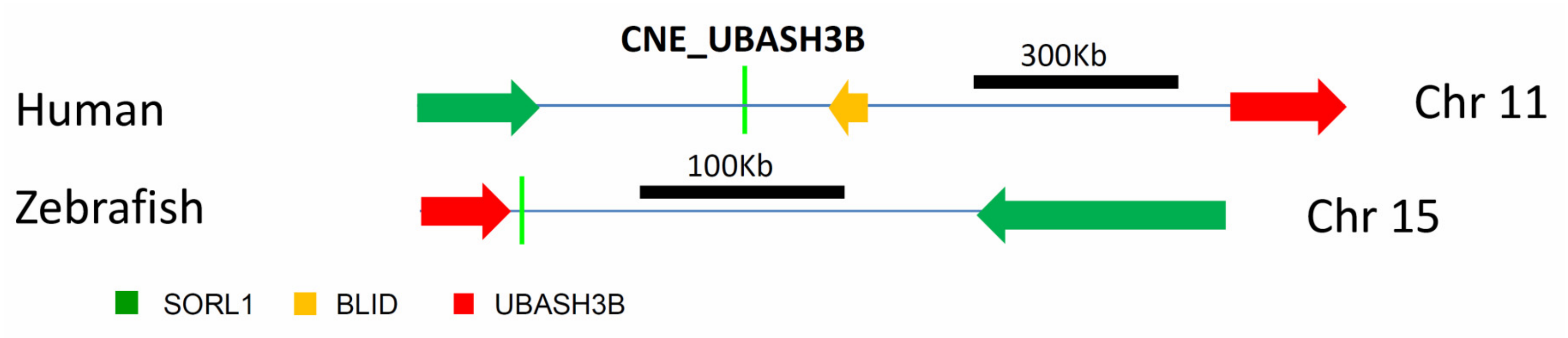
CNE_UBASH3B
300Kb
Human
Chr 11
100Kb
Zebrafish
Chr 15
SORL1
BLID
UBASH3B

B

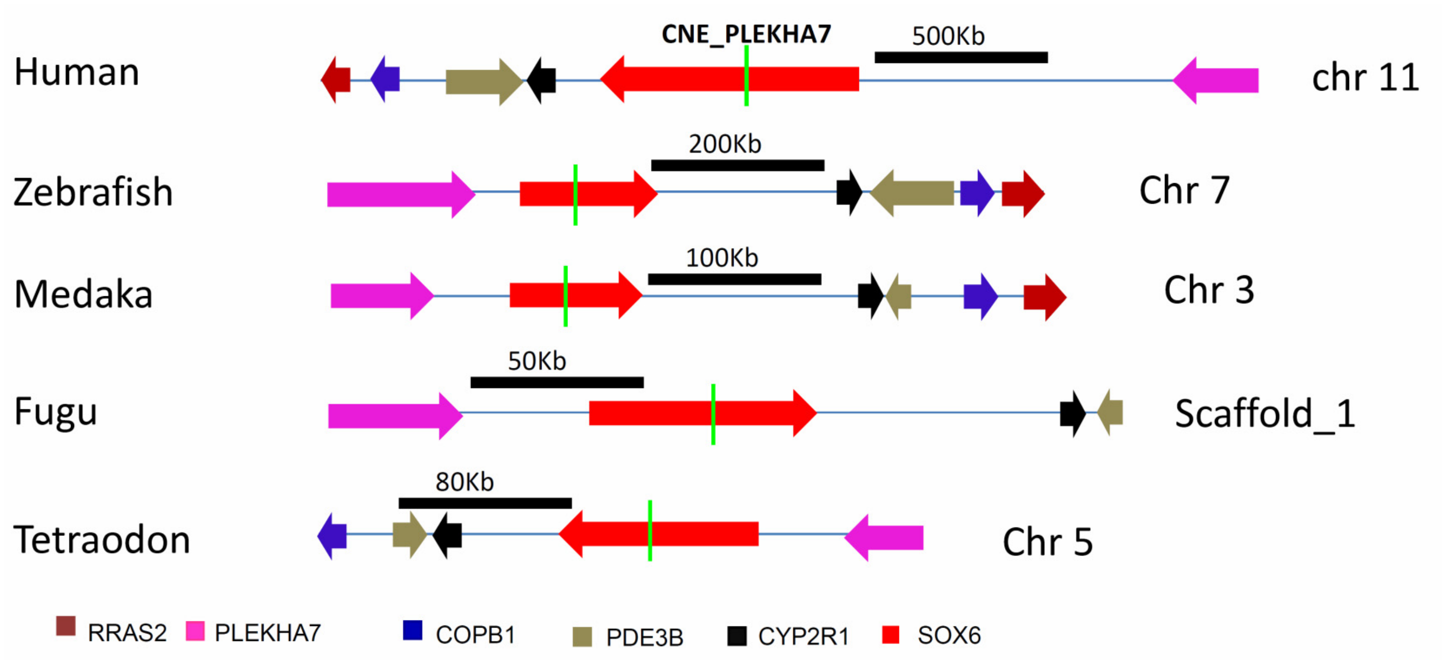
CNE_PLEKHA7
500Kb
Human
chr 11
200Kb
Zebrafish
Chr 7
100Kb
Medaka
Chr 3
50Kb
Fugu
Scaffold_1
80Kb
Tetraodon
Chr 5
RRAS2
PLEKHA7
COPB1
PDE3B
CYP2R1
SOX6

Figure 62 . Comparative analysis and expression pattern studies help in associating the enhancer to its target gene (hs872/hs236).

(A) A CNE-enhancer is located within the Intergenic region between *SORL1* and *BLID* genes in human genome. Careful syntenic analysis revealed that *BLID* is lost in fish genome. Analysis of expression pattern of genes and CNE-induced reporter gene activity associates this CNE with a neighboring gene, *UBASH3B*. **(B)** Although, a human CNE is having conserved localization within intron of *SOX6* gene, across human and teleost fish lineages, expression pattern analysis supports the association between this CNE and *PLEKHA7* gene.

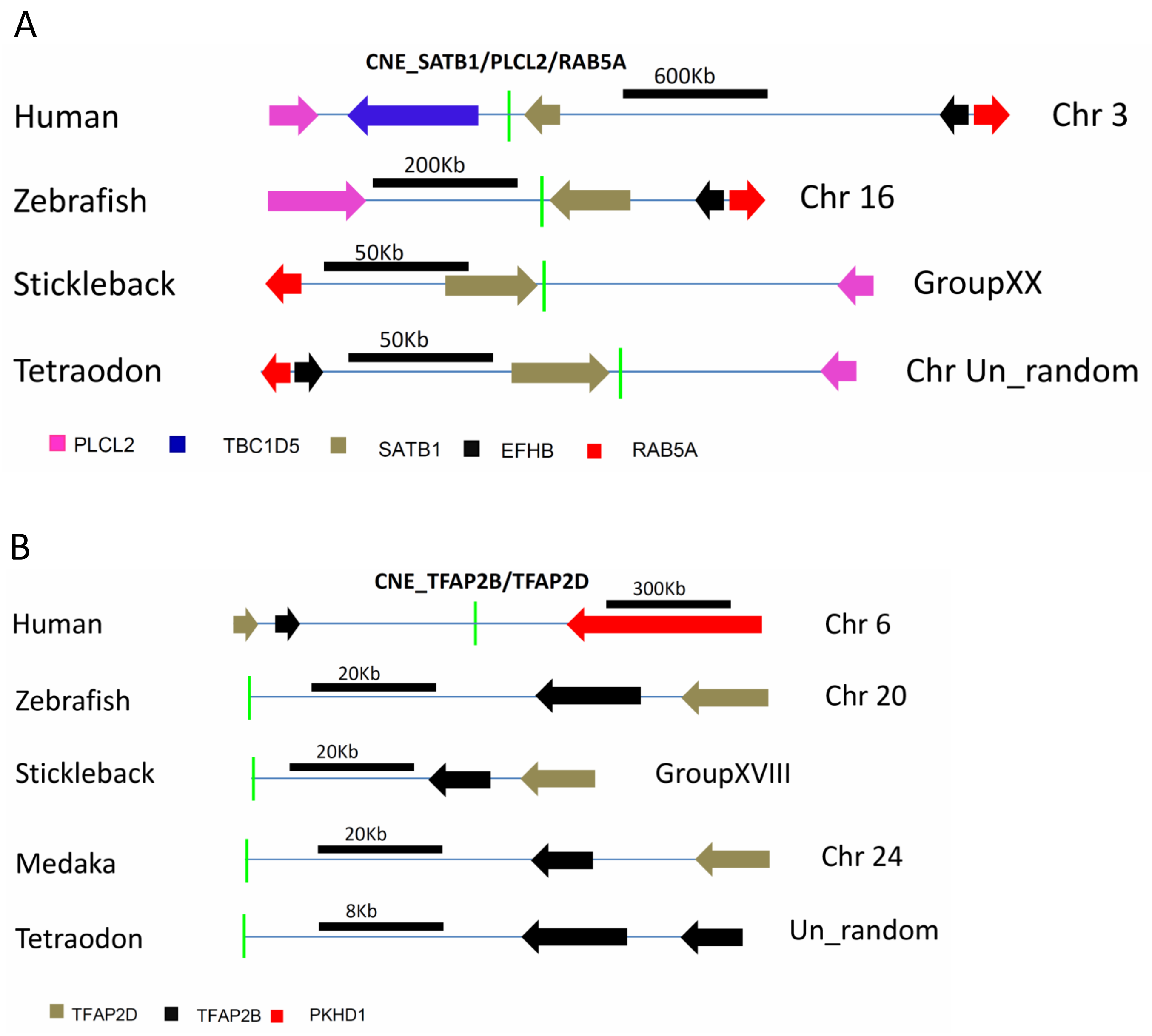
A
CNE_SATB1/PLCL2/RAB5A
600Kb
Human
Chr 3
200Kb
Zebrafish
Chr 16
50Kb
Stickleback
GroupXX
50Kb
Tetraodon
Chr Un_random
PLCL2
TBC1D5
SATB1
EFHB
RAB5A
B
CNE_TFAP2B/TFAP2D
300Kb
Human
Chr 6
20Kb
Zebrafish
Chr 20
20Kb
Stickleback
GroupXVIII
20Kb
Medaka
Chr 24
8Kb
Tetraodon
Un_random
TFAP2D
TFAP2B
PKHD1

Figure 63 . Comparative analysis and expression pattern studies help in finding the target gene for CNE-enhancer (hs250/hs217).

(A) Expression pattern analysis of conserved genes with CNE-induced reporter gene expression associates this CNE with *SATB1, PLCL2* and *RAB5A* genes. This association is also supported by conserved synteny across human and teleost fish genomes. **(B)** One of the flanking genes of a human CNE, *PKHD1*, is lost in fish genomes. There are two conserved genes near the CNE*, TFAP2B and TFAP2D*, which are suggested target genes of this CNE-enhancer.

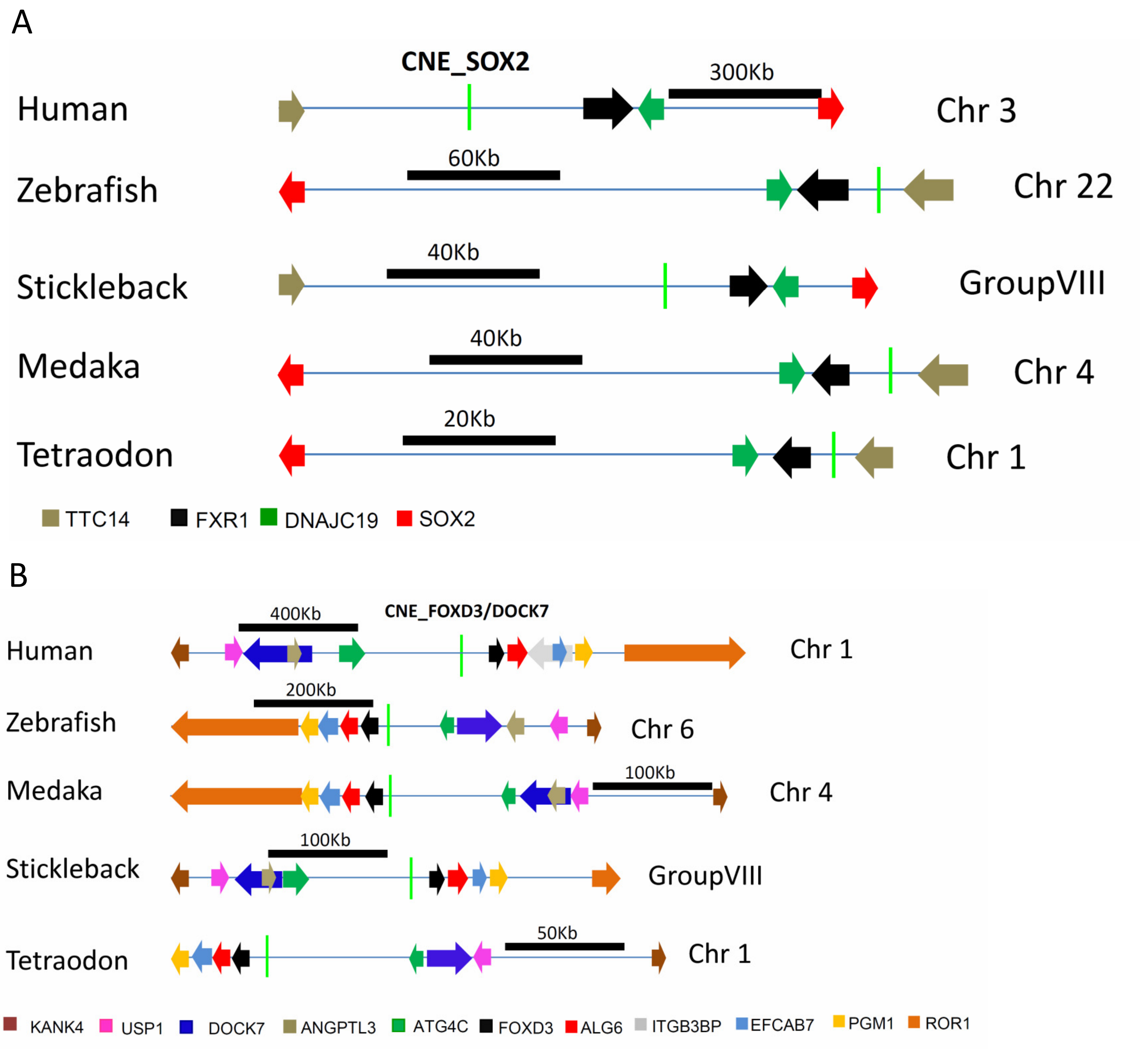

A
CNE_SOX2
300Kb
Human
Chr 3
60Kb
Zebrafish
Chr 22
40Kb
Stickleback
GroupVIII
40Kb
Medaka
Chr 4
20Kb
Tetraodon
Chr 1
TTC14
FXR1
DNAJC19
SOX2
B
400Kb
CNE_FOXD3/DOCK7
Human
Chr 1
200Kb
Zebrafish
Chr 6
100Kb
Medaka
Chr 4
100Kb
Stickleback
GroupVIII
50Kb
Tetraodon
Chr 1
KANK4
USP1
DOCK7
ANGPTL3
ATG4C
FOXD3
ALG6
ITGB3BP
EFCAB7
PGM1
ROR1

Figure 64 . Human CNE-enhancers by tracing the genic context of their orthologous copies in teleost fish lineage (hs258/hs764).

(A) Although, positioning of a human intragenic CNE in conserved across human and teleost fish lineages, expression pattern analysis indicates the *SOX2* as the target gene for this CNE. **(B)** A human CNE is located within the Intergenic region between *ATG4C* and *FOXD3* and this localization is conserved across human and teleost fish lineages. Expression pattern of *ATG4C* is different from that of CNE-induced reporter gene. Careful syntenic and expression pattern analysis reveals that this CNE is associated with *FOXD3* and *DOCK7* genes.

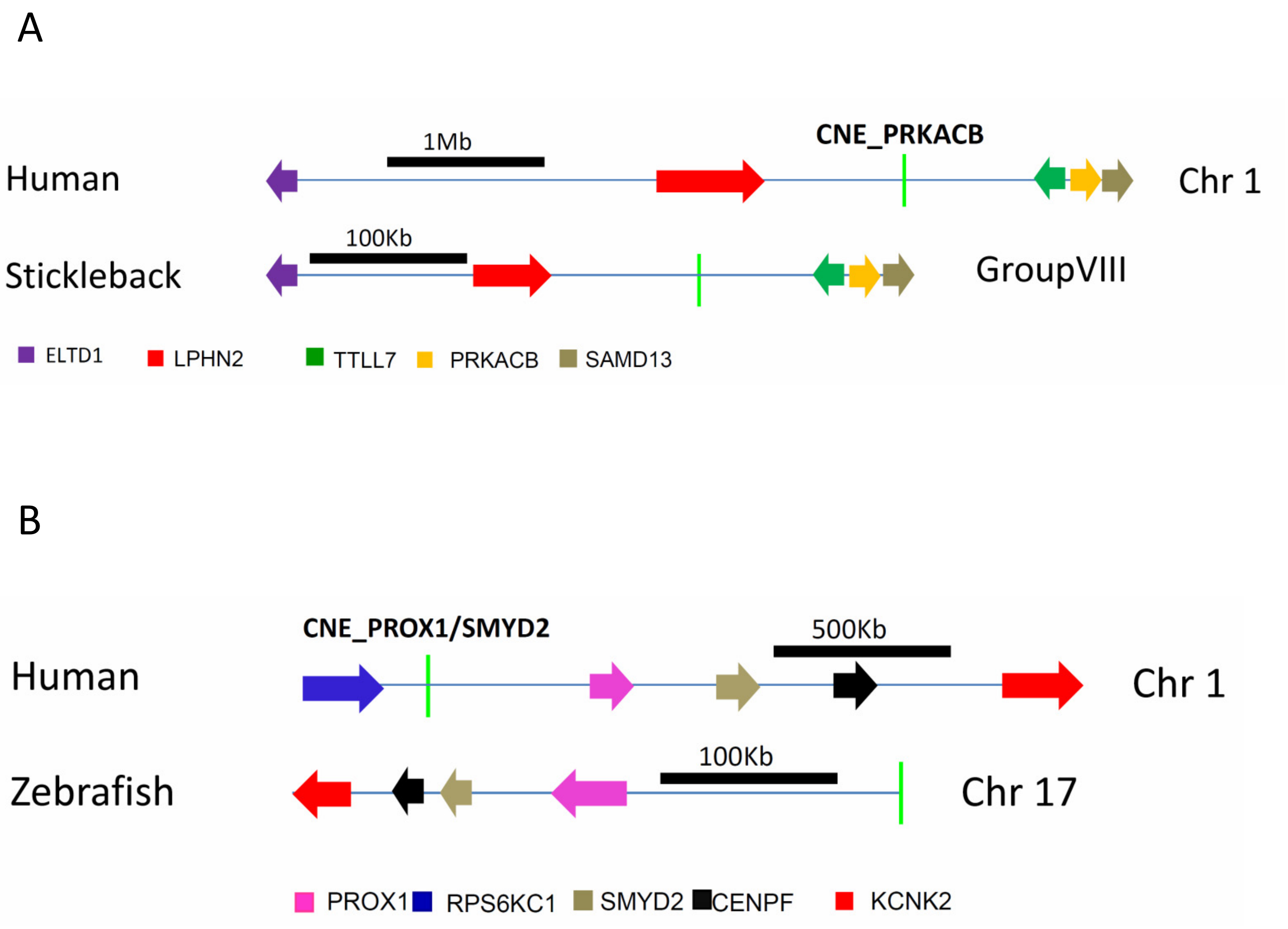
A
CNE_PRKACB
1Mb
Human
Chr 1
100Kb
Stickleback
GroupVIII
ELTD1
LPHN2
TTLL7
PRKACB
SAMD13
B
CNE_PROX1/SMYD2
500Kb
Human
Chr 1
100Kb
Zebrafish
Chr 17
PROX1
RPS6KC1
SMYD2
CENPF
KCNK2

Figure 65 . Analysis of CNE-enhancers in humans and its orthologous genomic intervals teleost fish lineage helps in associating CNE to their target genes (hs442/hs204).

(A) Intergenomic conserved synteny and expression pattern profile associates this human CNE with *PRKACB* gene. **(B)** A human CNE is associated with *PROX1* and *SMYD2* gene. One of the flanking genes, *RPS6KC1*, is lost in fish lineage.

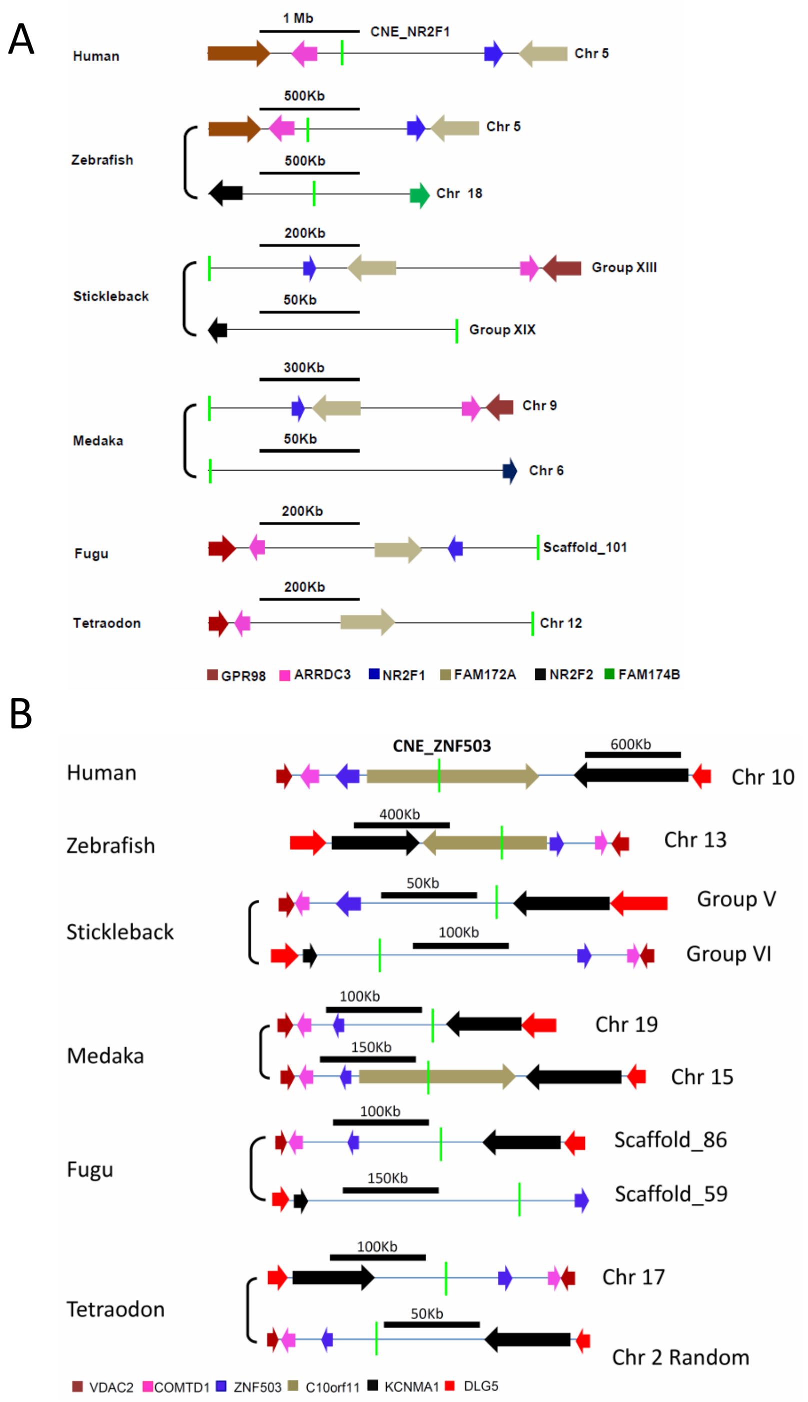

A
Human
1 Mb
CNE_NR2F1
Chr 5
Zebrafish
500Kb
Chr 5
500Kb
Chr 18
Stickleback
200Kb
Group XIII
50Kb
Group XIX
Medaka
300Kb
Chr 9
50Kb
Chr 6
Fugu
200Kb
Scaffold_101
Tetraodon
200Kb
Chr 12
GPR98 ARRDC3 NR2F1 FAM172A NR2F2 FAM174B
B
Human
CNE_ZNF503
600Kb
Chr 10
Zebrafish
400Kb
Chr 13
Stickleback
50Kb
Group V
100Kb
Group VI
Medaka
100Kb
Chr 19
150Kb
Chr 15
Fugu
100Kb
Scaffold_86
150Kb
Scaffold_59
Tetraodon
100Kb
Chr 17
50Kb
Chr 2 Random
VDAC2 COMTD1 ZNF503 C10orf11 KCNMA1 DLG5

Figure 66 . Predicting the target genes of human enhancers by analyzing their genic environment within and across vertebrate genomes (hs271/hs320).

(A) Careful syntenic analysis of CNE in human and fish lineage reveals that CNE is duplicated only in fish genomes. Conserved localization pattern of duplicated CNEs and their corresponding paralogous gene, and expression pattern analysis clearly refers *NR2F1* as the target gene for this CNE. **(B)** A CNE is duplicated only in fish lineage (Except Zebrafish). Conserved localization pattern of duplicated CNEs and their corresponding paralogous gene, and expression pattern analysis clearly refers *ZNF503* as the target gene for this CNE.

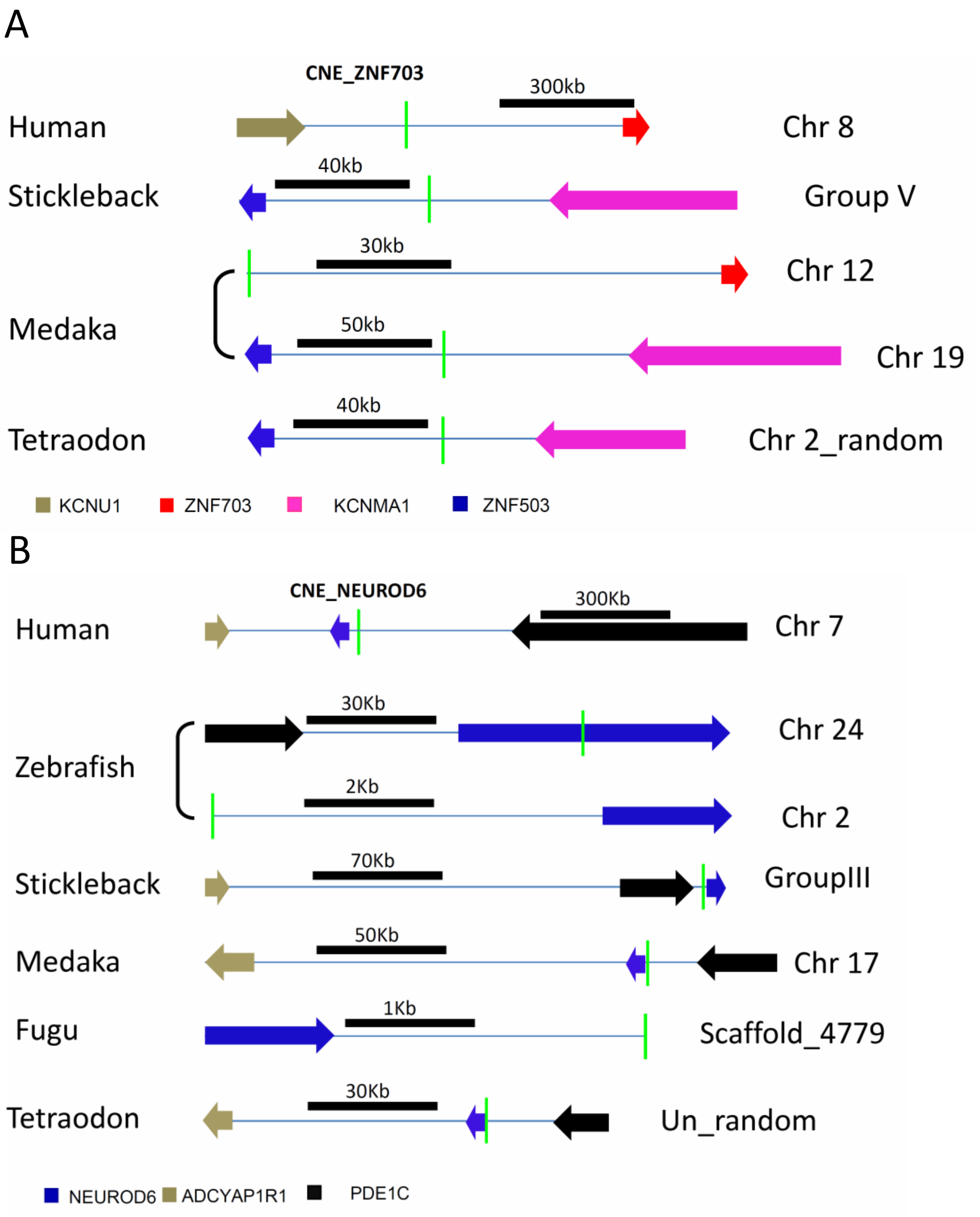

A
CNE_ZNF703
Human
300kb
Chr 8
Stickleback
40kb
Group V
30kb
Chr 12
Medaka
50kb
Chr 19
Tetraodon
40kb
Chr 2_random
KCNU1
ZNF703
KCNMA1
ZNF503
B
CNE_NEUROD6
Human
300Kb
Chr 7
Zebrafish
30Kb
Chr 24
2Kb
Chr 2
Stickleback
70Kb
GroupIII
Medaka
50Kb
Chr 17
Fugu
1Kb
Scaffold_4779
Tetraodon
30Kb
Un_random
NEUROD6
ADCYAP1R1
PDE1C

Figure 67 . Analyzing the genic environment of human CNE-enhancers in teleost fish orthologous loci and expression pattern of reporter gene helps in identifying their target genes (hs631/hs297).

(A) Syntenic analysis of a human CNE in teleost fish lineage reveals that this CNE is duplicated only in Medaka. CNE duplication pattern and endogenous expression profile suggests that this CNE is associated with *ZNF703* and dCNE is associated with paralog *ZNF503.* **(B)** A CNE is duplicated only in Zebrafish. Intergenomic conserved synteny, duplication pattern and expression profile refers *NEUROD6* as the target gene for this CNE.

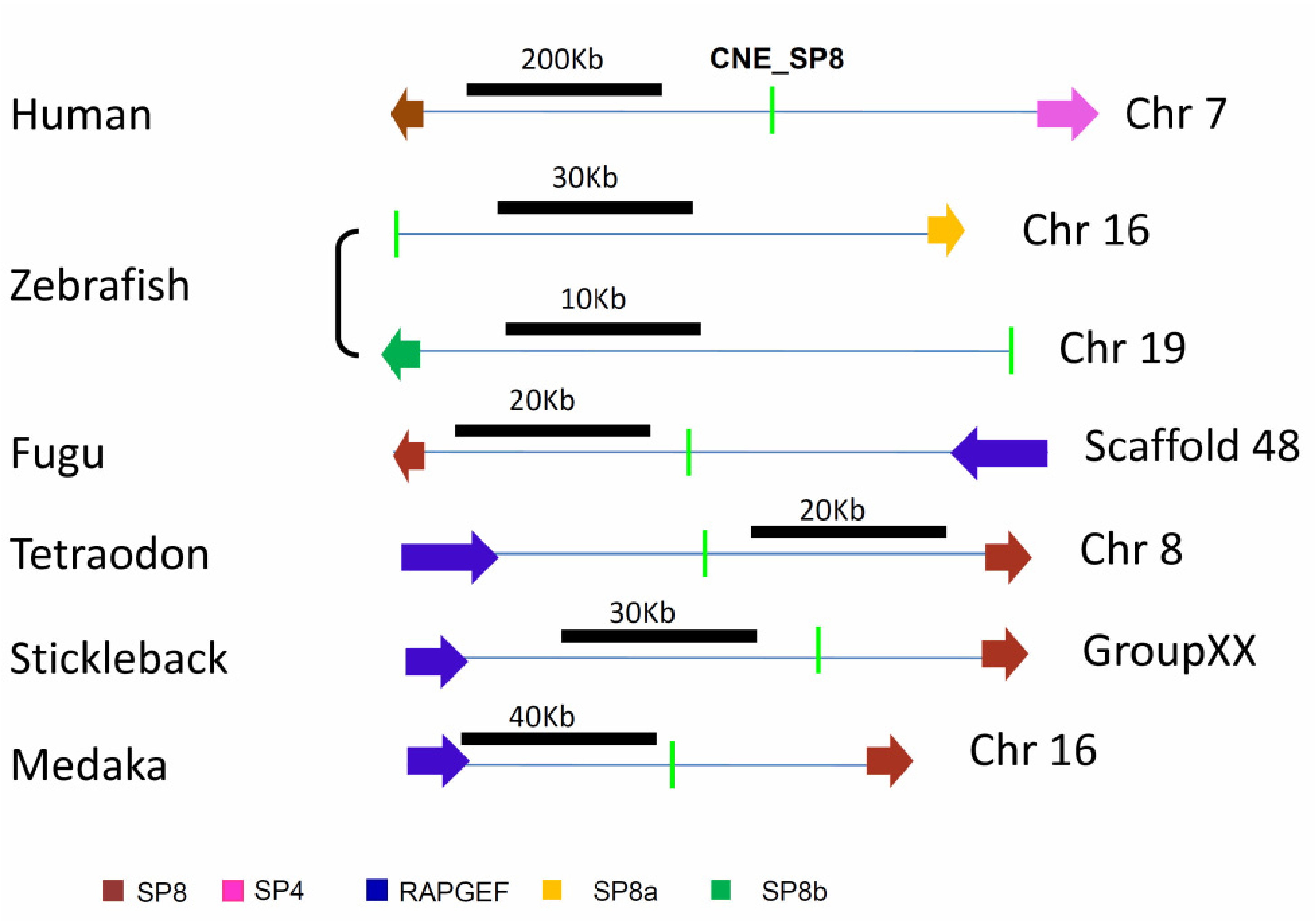

Human
200Kb
CNE_SP8
Chr 7
30Kb
Chr 16
Zebrafish
10Kb
Chr 19
20Kb
Fugu
Scaffold 48
20Kb
Tetraodon
Chr 8
30Kb
Stickleback
GroupXX
40Kb
Medaka
Chr 16
SP8
SP4
RAPGEF
SP8a
SP8b

Figure68 . Comparative analysis helps in assigning the CNE-enhancer to its target genes (hs110).

A CNE is duplicated only in Zebrafish. Intergenomic conserved synteny, duplication pattern and expression profile refers *SP8* as the target gene for this CNE.

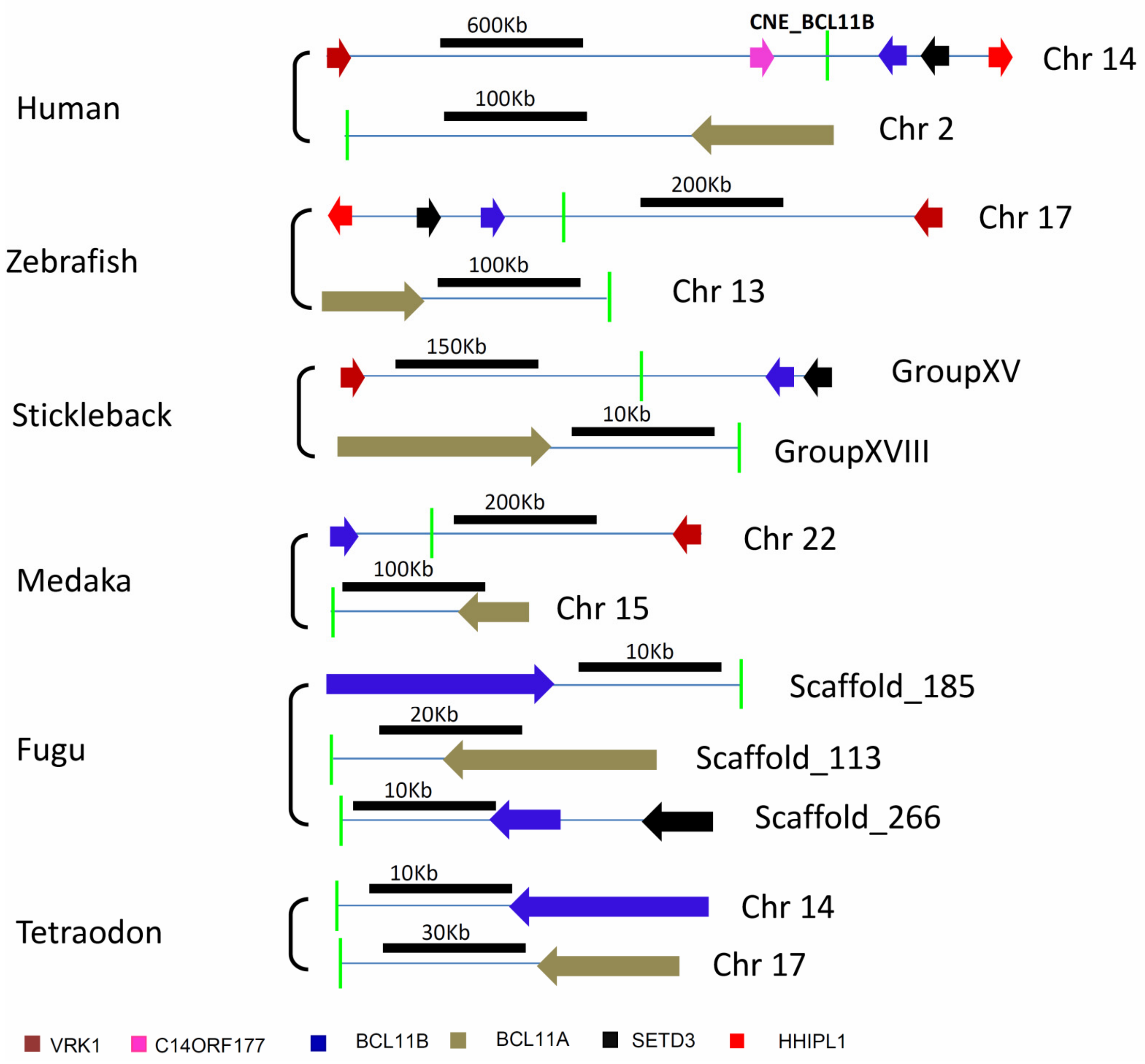

600Kb
CNE_BCL11B
Chr 14
100Kb
Chr 2
Human
200Kb
Chr 17
100Kb
Chr 13
Zebrafish
150Kb
GroupXV
10Kb
GroupXVIII
Stickleback
200Kb
Chr 22
100Kb
Chr 15
Medaka
10Kb
Scaffold_185
20Kb
Scaffold_113
10Kb
Scaffold_266
Fugu
10Kb
Chr 14
30Kb
Chr 17
Tetraodon
VRK1
C14ORF177
BCL11B
BCL11A
SETD3
HHIPL1

Figure 69 . Target gene identification of human CNE-enhancers through orthology mapping (hs622).

Duplication of human enhancer at the root of vertebrate lineage and preservation of linkage of duplicated enhancers with *BCL11* family members both within and across genomes, unmistakably suggest the specificity of these duplicated cis-regulatory regions towards *BCL11B* and *BCL11A* promoters.

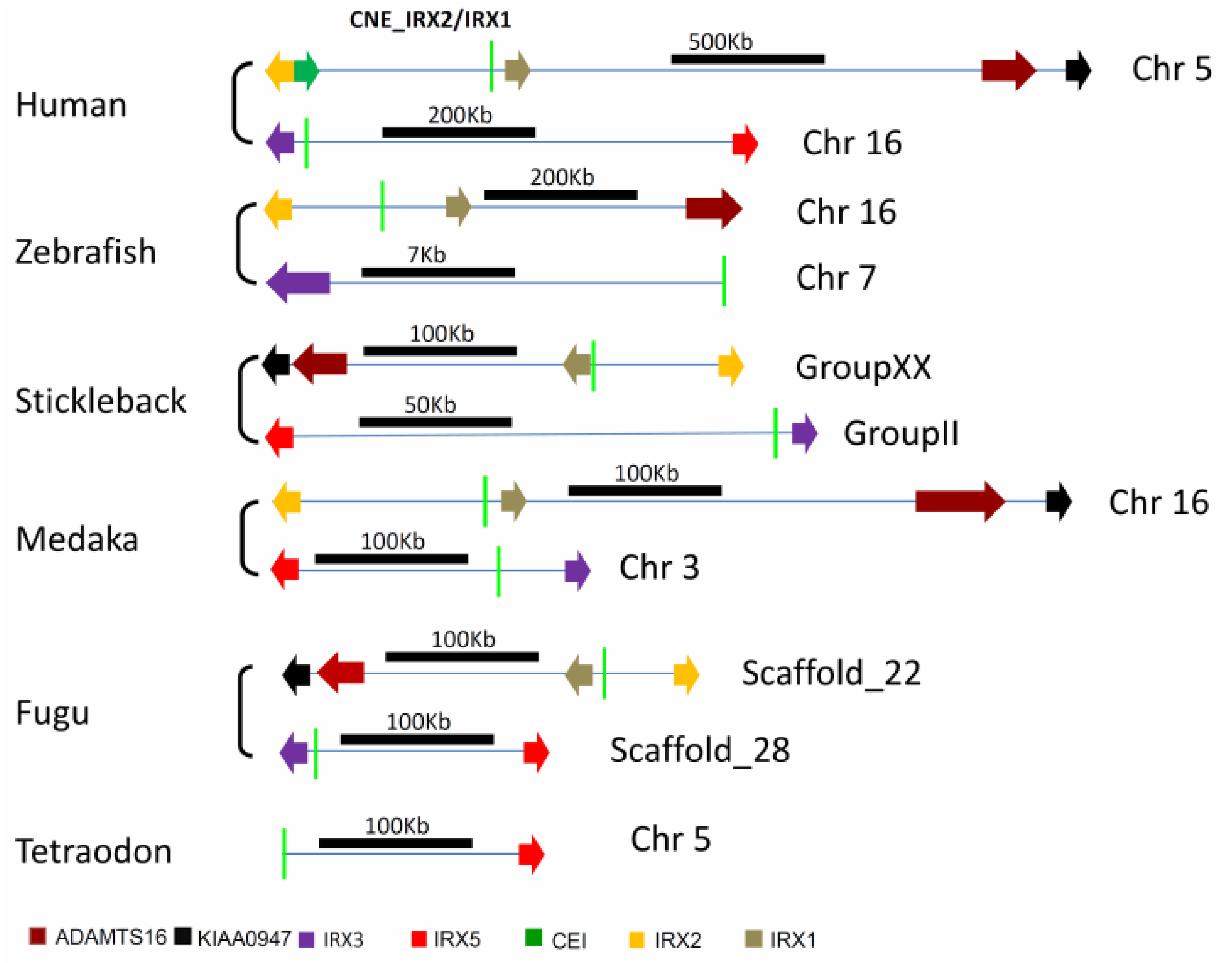
CNE_IRX2/IRX1
500Kb
Chr 5
Human
200Kb
Chr 16
200Kb
Chr 16
Zebrafish
7Kb
Chr 7
100Kb
GroupXX
Stickleback
50Kb
GroupII
100Kb
Chr 16
Medaka
100Kb
Chr 3
100Kb
Scaffold_22
Fugu
100Kb
Scaffold_28
Tetraodon
100Kb
Chr 5
ADAMTS16
KIAA0947
IRX3
IRX5
CEI
IRX2
IRX1

Figure 70 . Analysis of genic environment of human CNE-enhancers in teleost fish orthologous genomic intervals helps in identifying their target genes (hs261).

Human enhancer-target gene duplicated early in vertebrate history, before tetrapod-teleost split. Conserved association between duplicated enhancers and *IRX* paralogs indicate that these duplicated human enhancers (*CNE-IRX2, CNE-IRX1 and CNE-IRX5, CNE-IRX3*) are specific to *IRX2, IRX1, IRX5* and *IRX3* respectively.

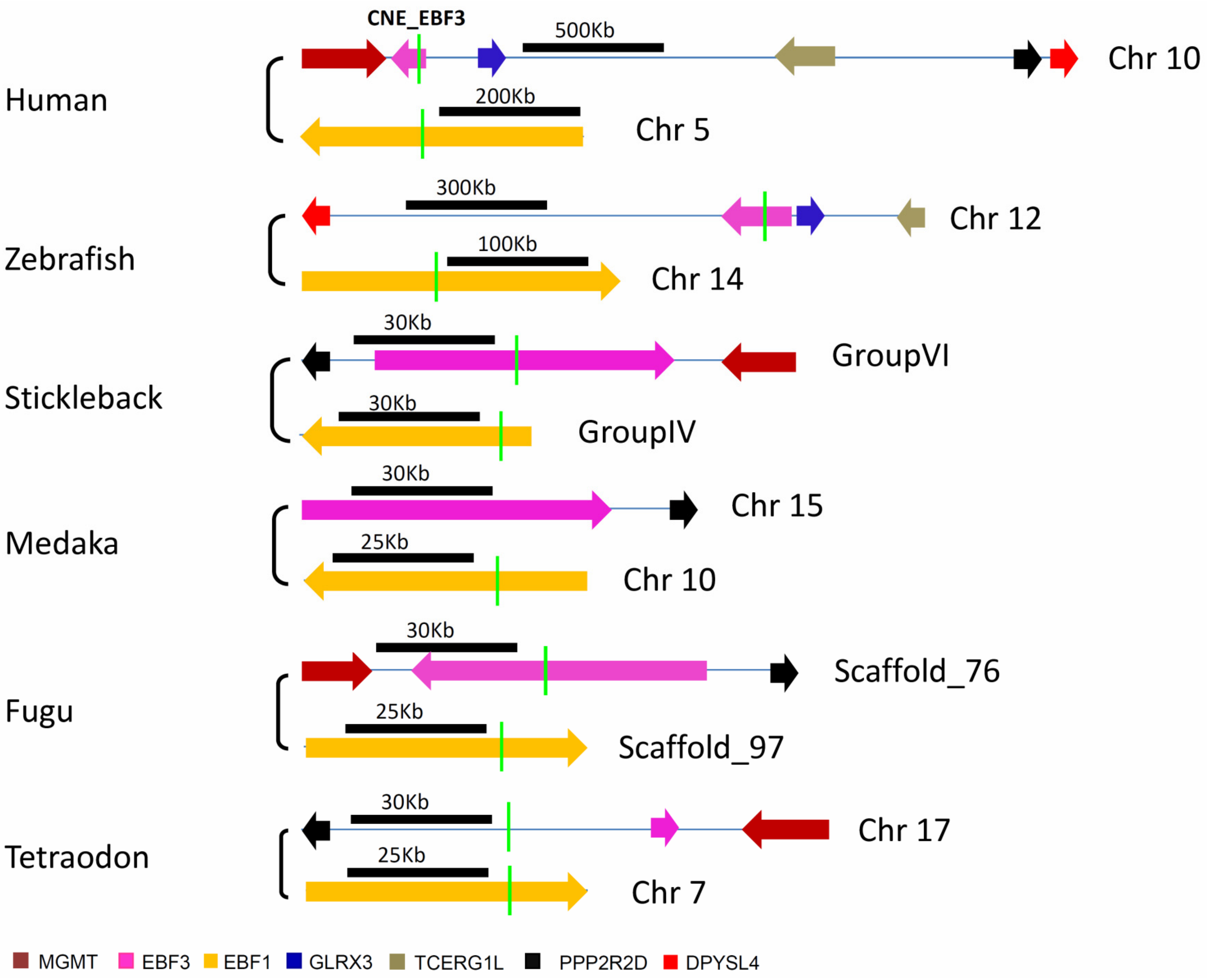
CNE_EBF3
500Kb
Chr 10
Human
200Kb
Chr 5
300Kb
Chr 12
Zebrafish
100Kb
Chr 14
30Kb
GroupVI
Stickleback
30Kb
GroupIV
30Kb
Chr 15
Medaka
25Kb
Chr 10
30Kb
Scaffold_76
Fugu
25Kb
Scaffold_97
30Kb
Chr 17
Tetraodon
25Kb
Chr 7
MGMT
EBF3
EBF1
GLRX3
TCERG1L
PPP2R2D
DPYSL4

Figure 71 . Assigning the target genes to CNE-enhancers through comparative genomic analysis (hs232).

Human enhancer-target gene duplicated early in vertebrate history, before tetrapod-teleost split. Conserved association between duplicated enhancers and *EBF* paralogs indicate that these duplicated human enhancers (*CNE-EBF3* and *CNE-EBF1*) are specific to *EBF3* and *EBF1*, respectively.

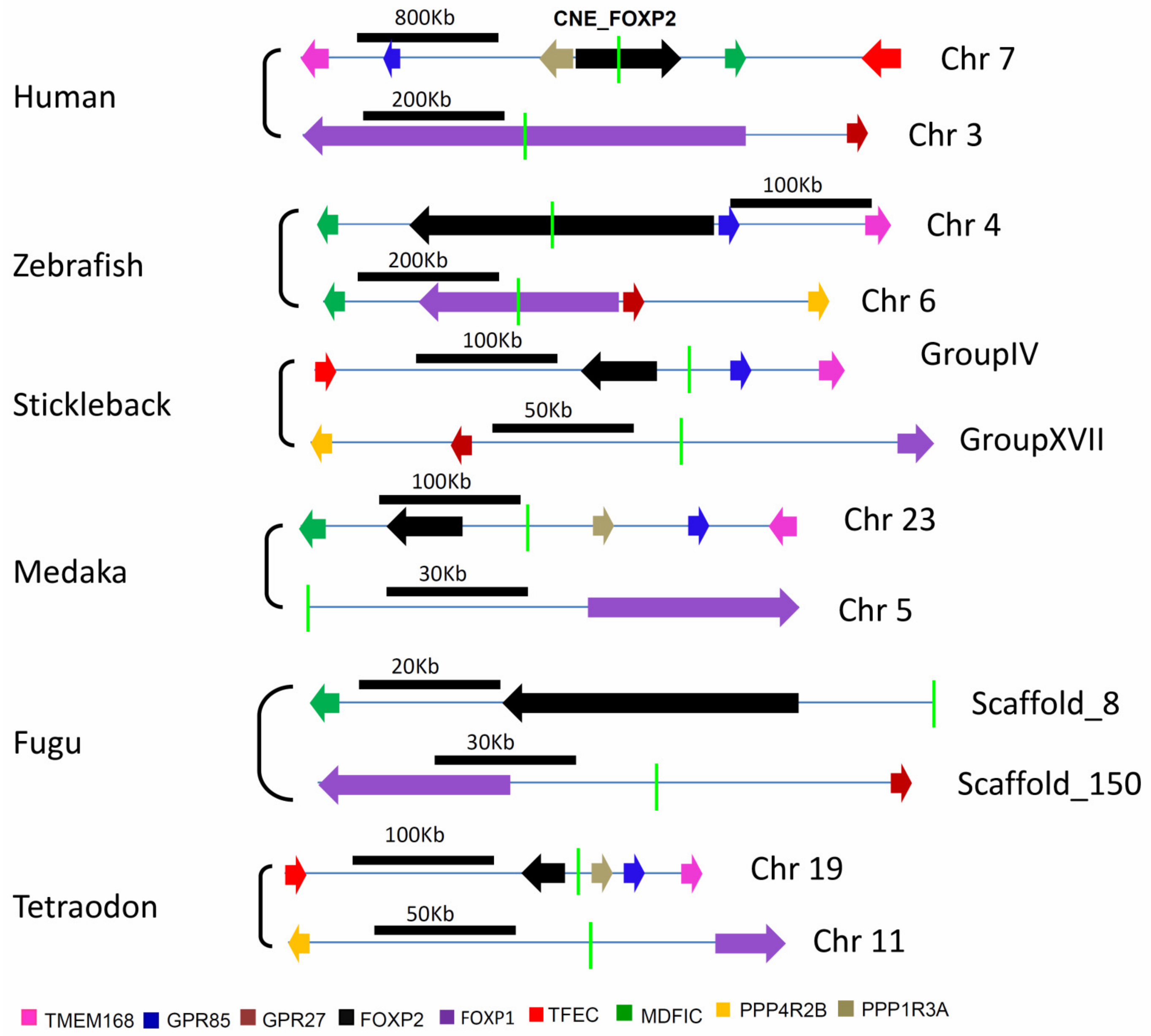

Human
Chr 7
Chr 3
800Kb
CNE_FOXP2
200Kb
Zebrafish
100Kb
Chr 4
200Kb
Chr 6
Stickleback
100Kb
GroupIV
50Kb
GroupXVII
Medaka
100Kb
Chr 23
30Kb
Chr 5
Fugu
20Kb
Scaffold_8
30Kb
Scaffold_150
Tetraodon
100Kb
Chr 19
50Kb
Chr 11
TMEM168
GPR85
GPR27
FOXP2
FOXP1
TFEC
MDFIC
PPP4R2B
PPP1R3A

Figure 72 . Assigning the target genes to CNE-enhancers through comparative genomic analysis (hs218)*.*

Duplication of human enhancer at the root of vertebrate lineage and preservation of linkage of duplicated enhancers with *FOXP* family members both within and across genomes, unmistakably suggest the specificity of these duplicated cis-regulatory regions (*CNE-FOXP2* and *CNE-FOXP1*) towards *FOXP2* and *FOXP1* promoters**.**

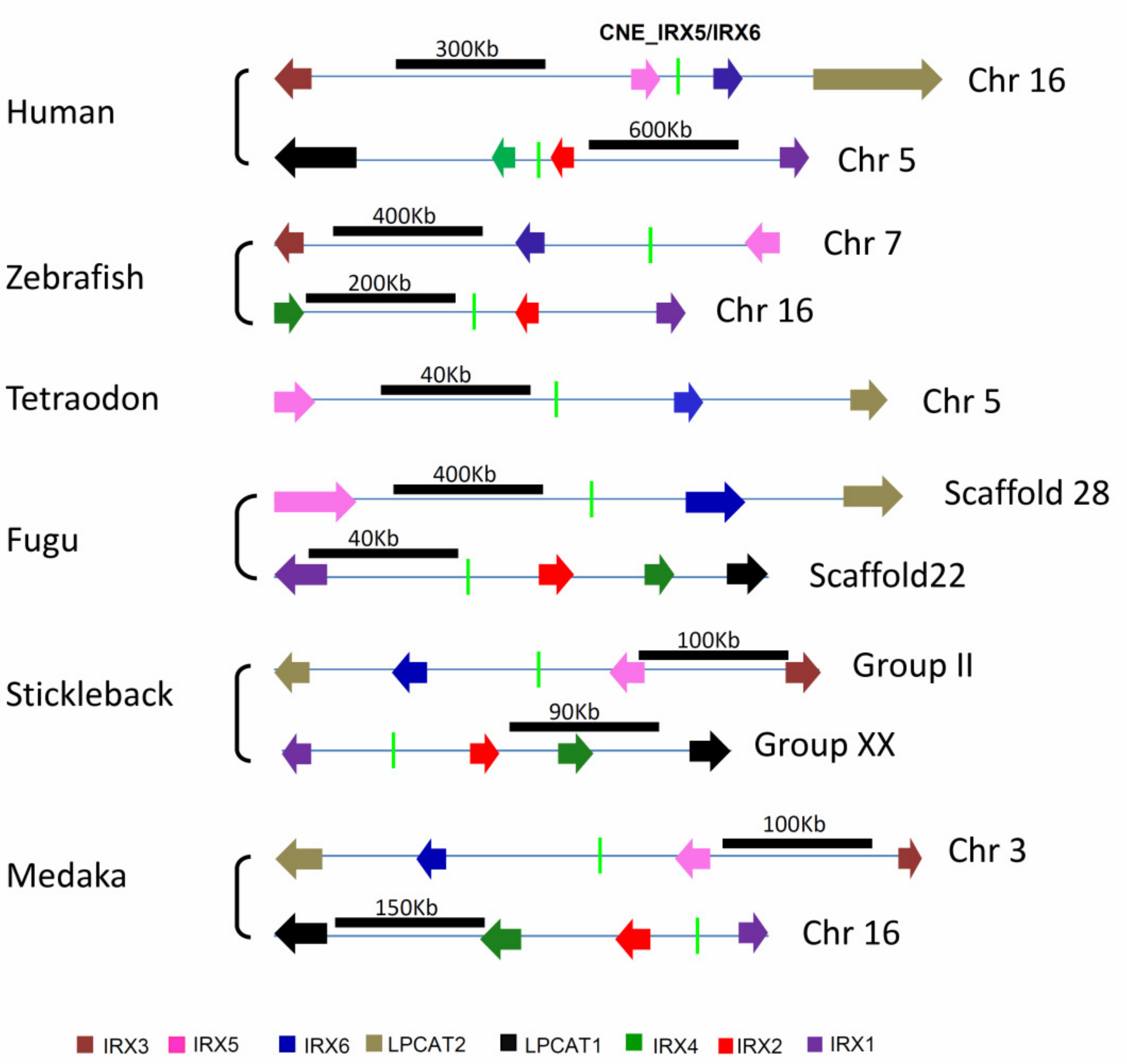

CNE_IRX5/IRX6
Human
300Kb
Chr 16
600Kb
Chr 5
Zebrafish
400Kb
Chr 7
200Kb
Chr 16
Tetraodon
40Kb
Chr 5
Fugu
400Kb
Scaffold 28
40Kb
Scaffold22
Stickleback
100Kb
Group II
90Kb
Group XX
Medaka
100Kb
Chr 3
150Kb
Chr 16
IRX3
IRX5
IRX6
LPCAT2
LPCAT1
IRX4
IRX2
IRX1

Figure 73 . Analyzing the genic architecture of human (hs26).

Human enhancer-target gene duplicated early in vertebrate history, before tetrapod-teleost split. Conserved association between duplicated enhancers and *IRX* paralogs indicate that these duplicated human enhancers (*CNE-IRX5, CNE-IRX6 and CNE-IRX2, CNE-IRX4*) are specific to *IRX5, IRX6, IRX2* and *IRX4* respectively.

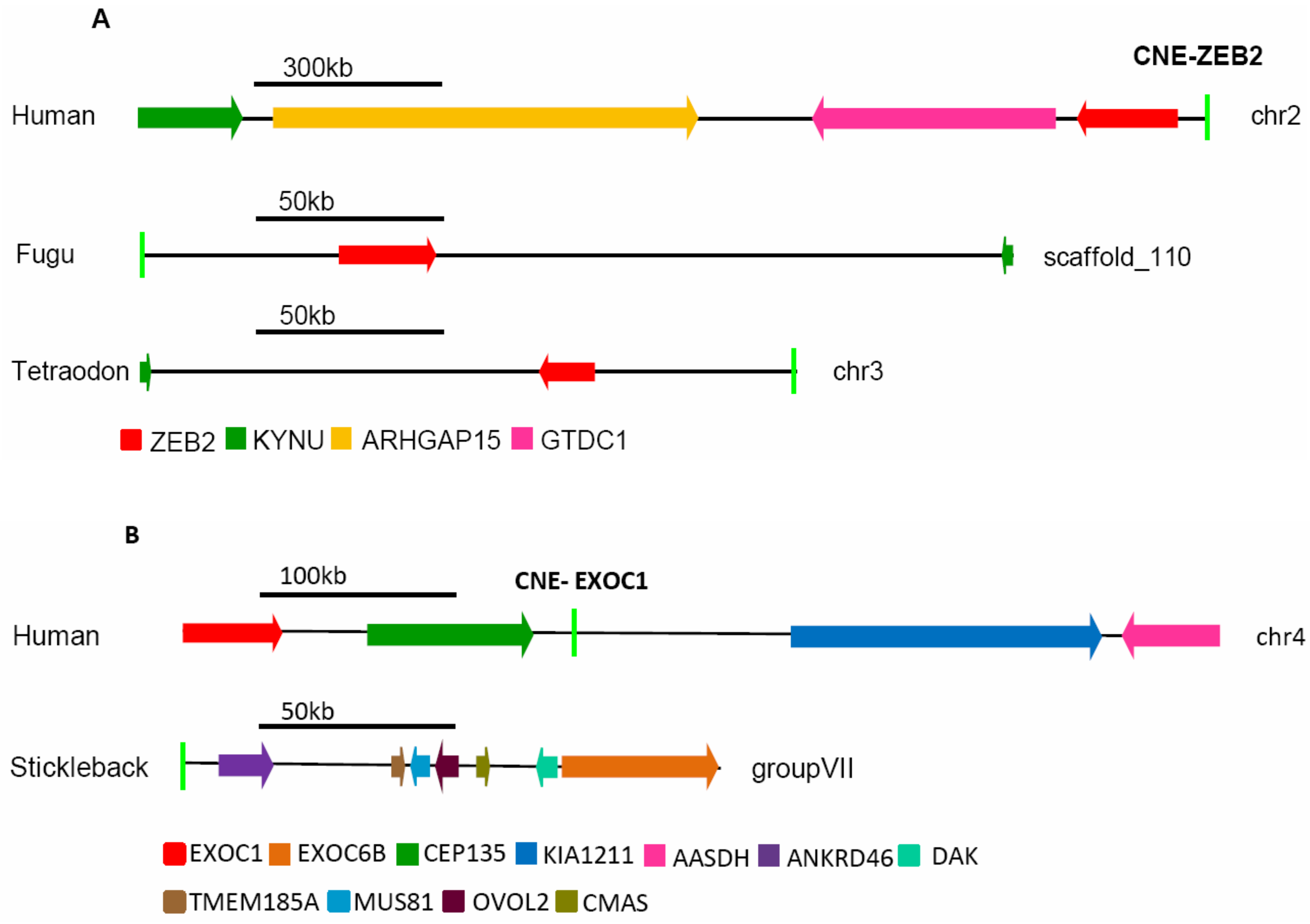
A
CNE-ZEB2
300kb
Human
chr2
50kb
Fugu
scaffold_110
50kb
Tetraodon
chr3
ZEB2 KYNU ARHGAP15 GTDC1
B
100kb
CNE- EXOC1
Human
chr4
50kb
Stickleback
groupVII
EXOC1 EXOC6B CEP135 KIA1211 AASDH ANKRD46 DAK
TMEM185A MUS81 OVOL2 CMAS

Figure 74. Analysis of genic environment of human CNE-enhancers in teleost fish orthologous genomic intervals and observing their expression patterns helps in identifying their target genes (hs1802/hs1651).

Analysis of CNE-enhancers in humans and its orthologous genomic intervals teleost fish lineage helps in associating CNE to their target genes. **(A)** Both synteny analysis and endogenous expression pattern clearly associate the CNE to human *ZEB2* gene. **(B)** CNE-enhancer within an intergenic space between human *CEP135* and *KIA1211* gene is associated with neighboring *EXOC1* gene.

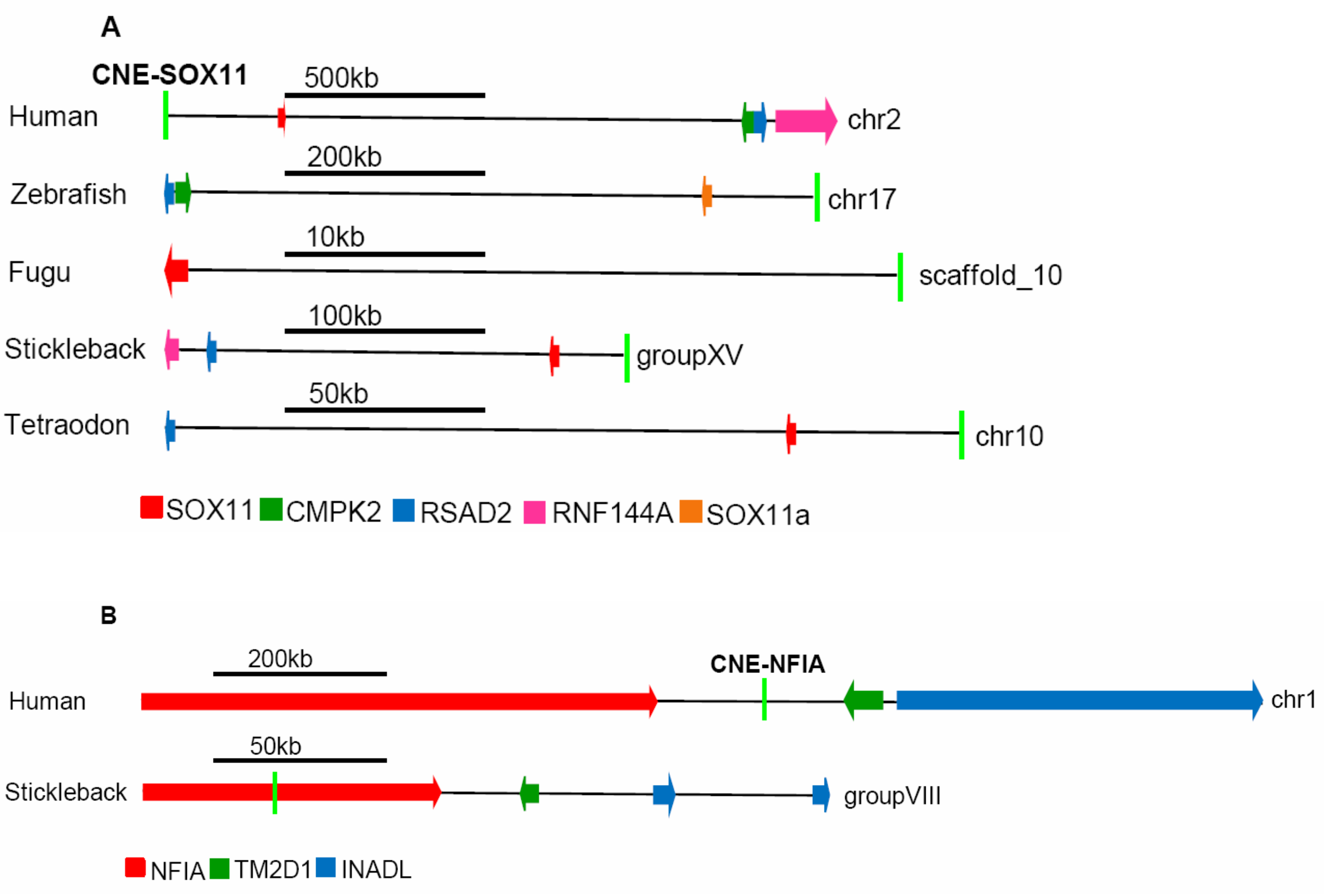

A
CNE-SOX11
500kb
Human
chr2
200kb
Zebrafish
chr17
10kb
Fugu
scaffold_10
100kb
Stickleback
groupXV
50kb
Tetraodon
chr10
SOX11
CMPK2
RSAD2
RNF144A
SOX11a
B
200kb
CNE-NFIA
Human
chr1
50kb
Stickleback
groupVIII
NFIA
TM2D1
INADL

Figure 75. Analysis of genic environment of human CNE-enhancers in teleost fish orthologous genomic intervals and observing their expression patterns helps in identifying their target genes (hs1496/hs1450).

Analysis of CNE-enhancers in humans and its orthologous genomic intervals teleost fish lineage helps in associating CNE to their target genes. **(A)** Both synteny analysis and endogenous expression pattern clearly associate the CNE to human *SOX11* gene. **(B)** CNE-enhancer within an intergenic space between human *NFIA* and *TM2D1* is associated with *NFIA* gene.

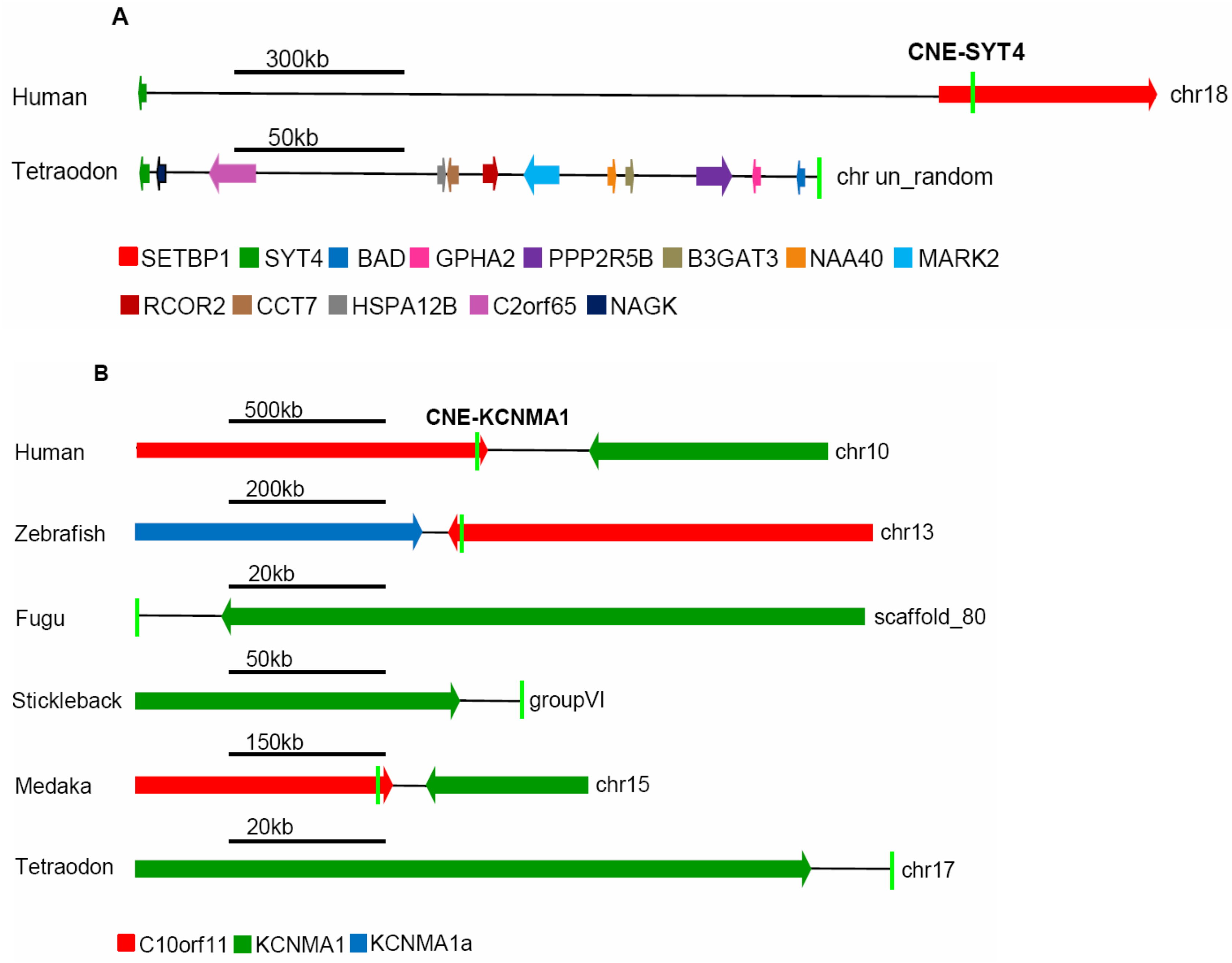
A
300kb
CNE-SYT4
Human
chr18
50kb
Tetraodon
chr un_random
SETBP1
SYT4
BAD
GPHA2
PPP2R5B
B3GAT3
NAA40
MARK2
RCOR2
CCT7
HSPA12B
C2orf65
NAGK
B
500kb
CNE-KCNMA1
Human
chr10
200kb
Zebrafish
chr13
20kb
Fugu
scaffold_80
50kb
Stickleback
groupVI
150kb
Medaka
chr15
20kb
Tetraodon
chr17
C10orf11
KCNMA1
KCNMA1a

Figure 76. Analysis of genic environment of human CNE-enhancers in teleost fish orthologous genomic intervals and observing their expression patterns helps in identifying their target genes (hs1362/hs1434).

Analysis of CNE-enhancers in humans and its orthologous genomic intervals teleost fish lineage helps in associating CNE to their target genes. **(A)** CNE-enhancer within an intron of human *SETBP1* gene is associated with neighboring *SYT4*. **(B)** CNE-enhancer within an intron of human *C10orf11* gene is associated with neighboring *KCNMA1*.

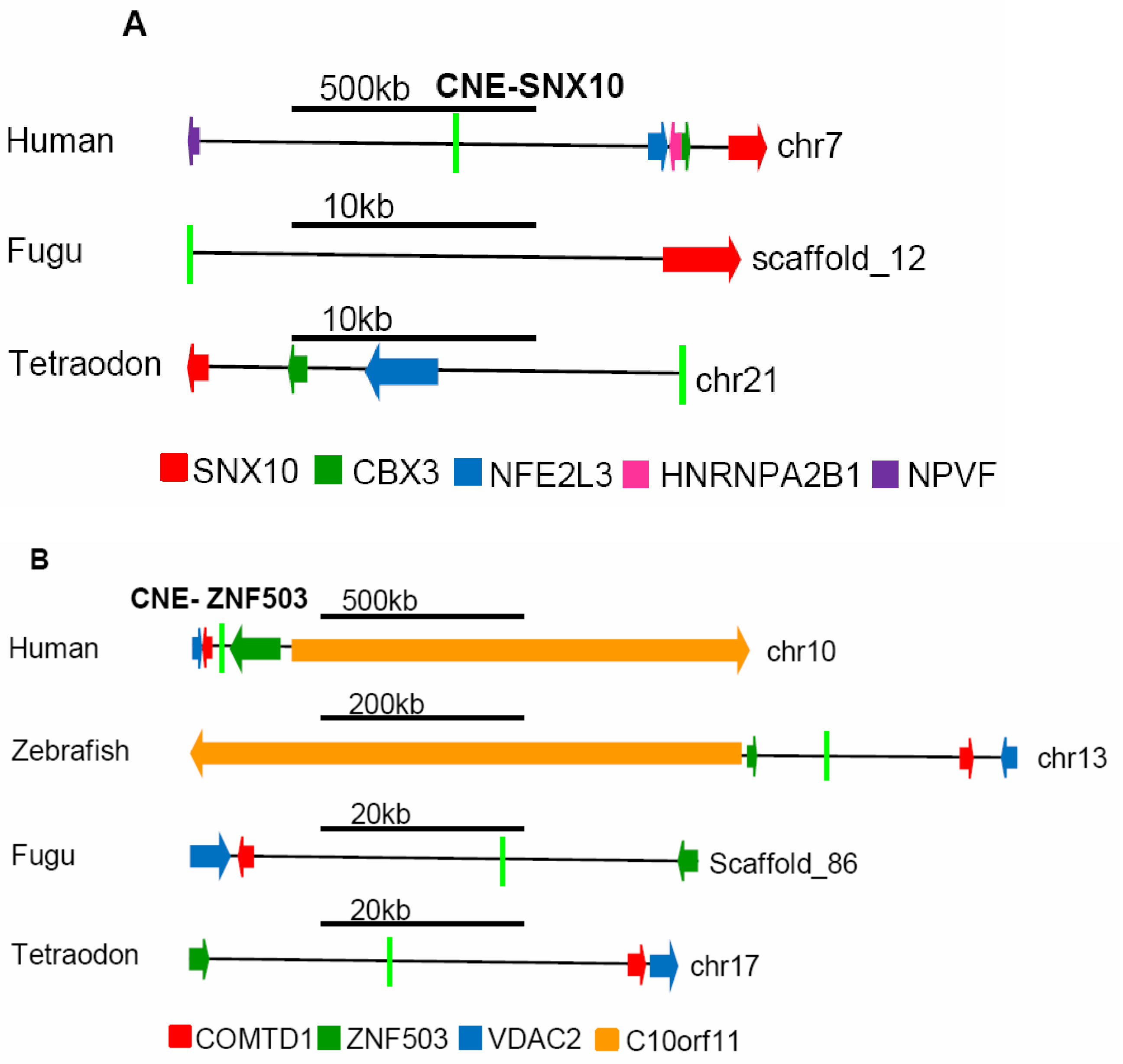
A
500kb
CNE-SNX10
Human
chr7
10kb
Fugu
scaffold_12
10kb
Tetraodon
chr21
SNX10
CBX3
NFE2L3
HNRNPA2B1
NPVF
B
CNE- ZNF503
500kb
Human
chr10
200kb
Zebrafish
chr13
20kb
Fugu
Scaffold_86
20kb
Tetraodon
chr17
COMTD1
ZNF503
VDAC2
C10orf11

Figure 77. Analysis of genic environment of human CNE-enhancers in teleost fish orthologous genomic intervals and observing their expression patterns helps in identifying their target genes (hs1325/hs1437).

Analysis of CNE-enhancers in humans and its orthologous genomic intervals teleost fish lineage helps in associating CNE to their target genes. **(A)** The localization of a human CNE-enhancer in the intergenic space between *NPVF* and *NFE2L3* genes suggests that this enhancer might be associated with one of these genes but not with *SNX10* that is present ~ 104.8 kb downstream of *NFE2L3*. However the careful analysis of genic context of orthologous regions of this element in teleost fish and human clearly associate the CNE to human *SNX10* gene. **(B)** CNE-enhancer within an intergenic space between human *COMTD1* and *ZNF503* genes. Careful analysis of genic context of orthologous regions of this element in teleost fish and human clearly associate the CNE to human *ZNF503*.

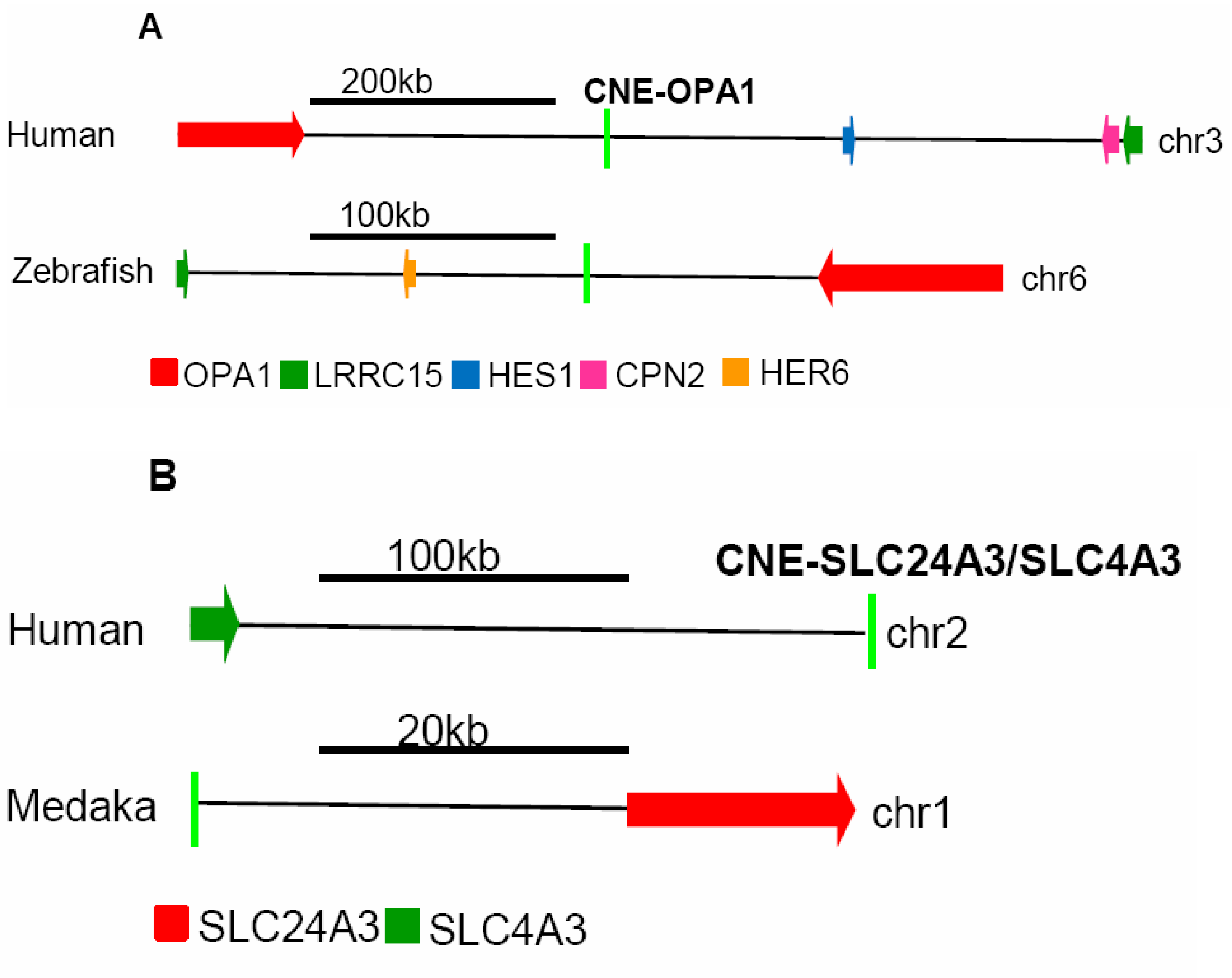
A
200kb
CNE-OPA1
Human
chr3
100kb
Zebrafish
chr6
OPA1
LRRC15
HES1
CPN2
HER6
B
100kb
CNE-SLC24A3/SLC4A3
Human
chr2
20kb
Medaka
chr1
SLC24A3
SLC4A3

Figure 78. Analysis of genic environment of human CNE-enhancers in teleost fish orthologous genomic intervals and observing their expression patterns helps in identifying their target genes (hs1344/hs1507).

Analysis of CNE-enhancers in humans and its orthologous genomic intervals teleost fish lineage helps in associating CNE to their target genes. **(A)** CNE-enhancer within an intergenic space between human *OPA1* and *HES1* is associated with *OPA1*. **(B)** Synteny analysis associate CNE-enhancer to *SLC4A3* gene but its expression pattern is not clearly defined but synteny analysis provides a strong evidence is favor of its association with *SLC4A3* gene.

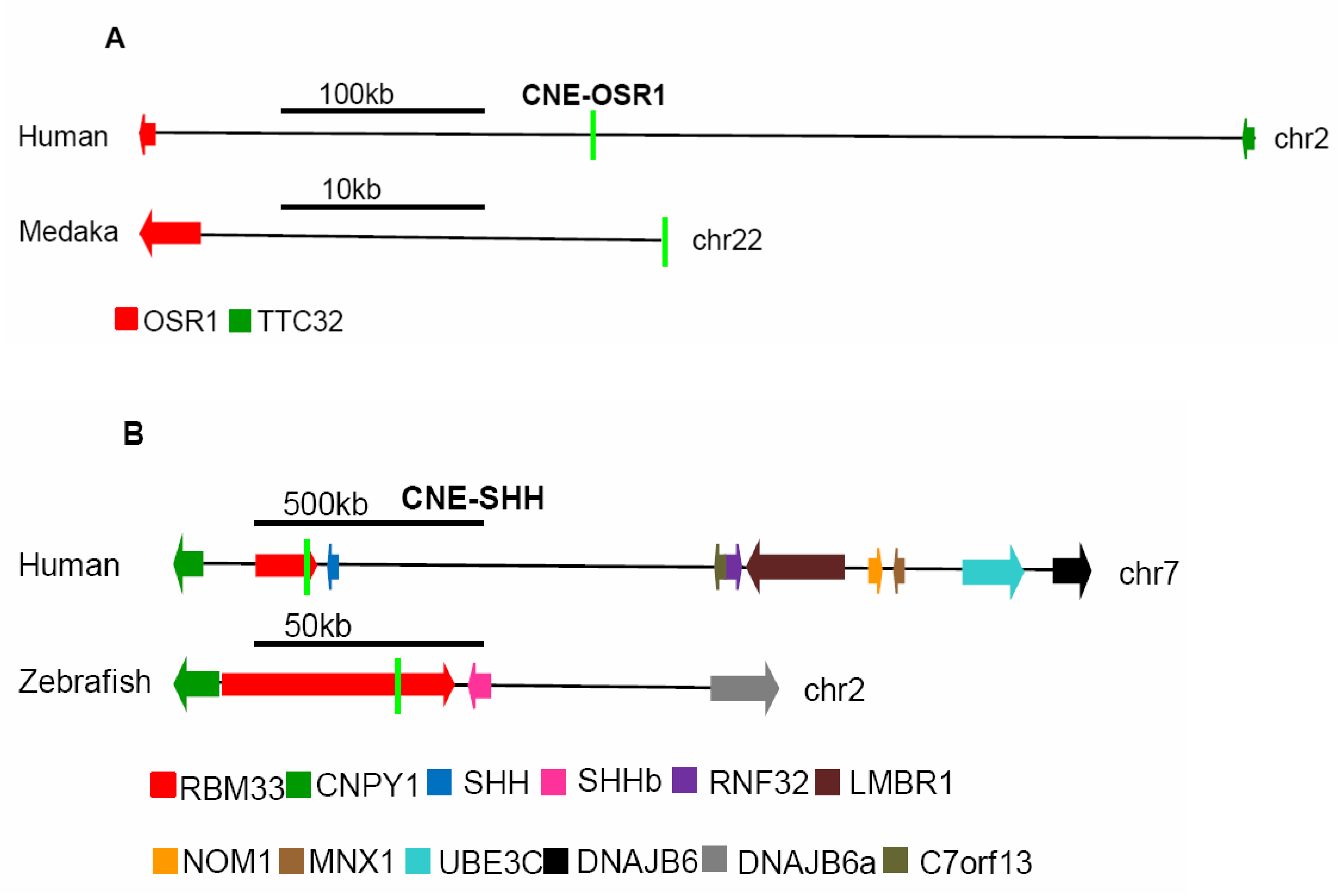
A
100kb
CNE-OSR1
Human
chr2
10kb
Medaka
chr22
OSR1
TTC32
B
500kb
CNE-SHH
Human
chr7
50kb
Zebrafish
chr2
RBM33
CNPY1
SHH
SHHb
RNF32
LMBR1
NOM1
MNX1
UBE3C
DNAJB6
DNAJB6a
C7orf13

Figure 79. Analysis of genic environment of human CNE-enhancers in teleost fish orthologous genomic intervals and observing their expression patterns helps in identifying their target genes (hs1273/hs1268).

Analysis of CNE-enhancers in humans and its orthologous genomic intervals teleost fish lineage helps in associating CNE to their target genes. **(A)** CNE-enhancer within an intergenic space between human *OSR1* and *TTC32* is associated with *OSR1*. **(B)** CNE-enhancer within an intron of human *RBM33* gene is associated with neighboring *SHH* gene.

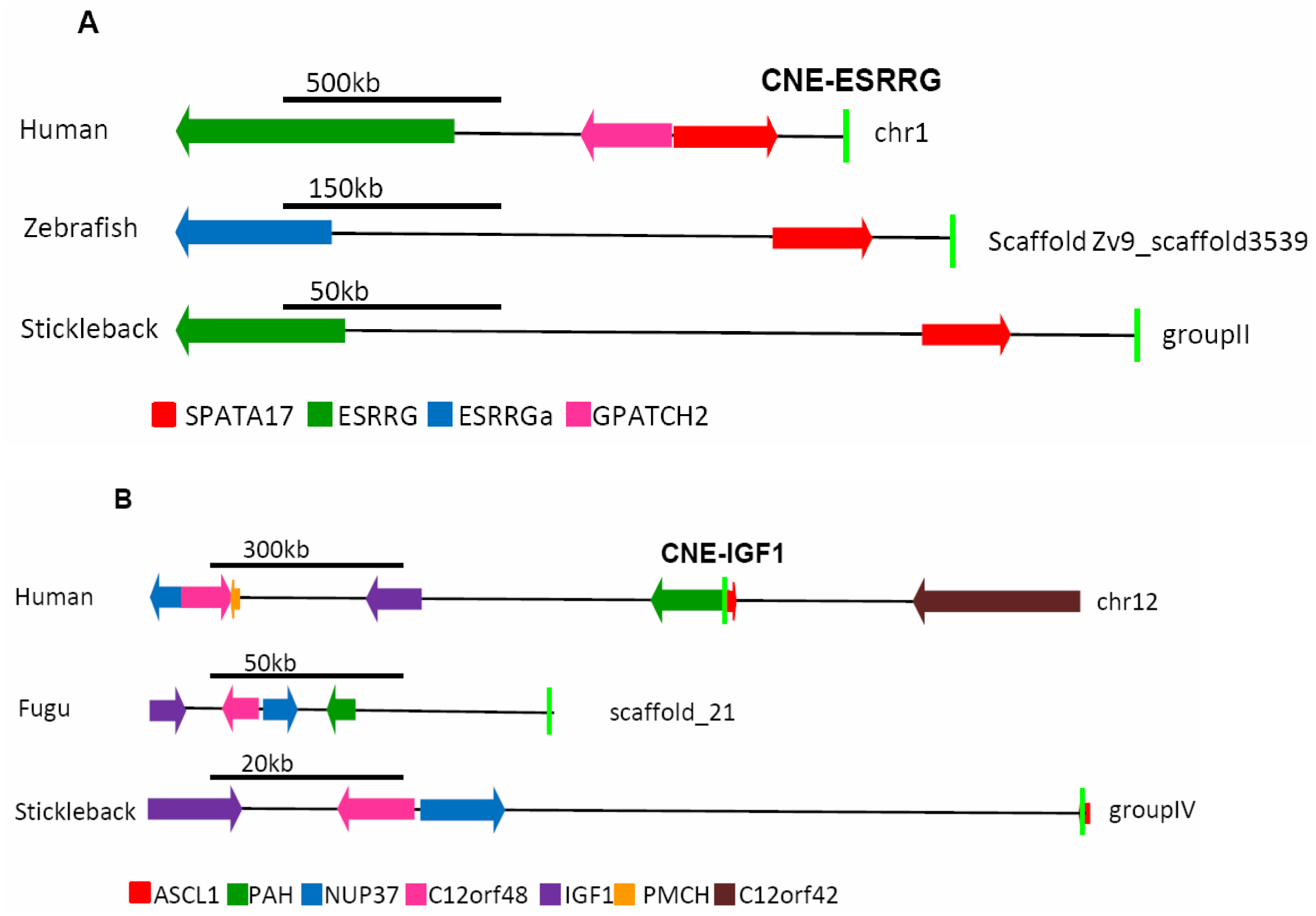
A
CNE-ESRRG
500kb
Human
chr1
150kb
Zebrafish
Scaffold Zv9_scaffold3539
50kb
Stickleback
groupII
SPATA17
ESRRG
ESRRGa
GPATCH2
B
CNE-IGF1
300kb
Human
chr12
50kb
Fugu
scaffold_21
20kb
Stickleback
groupIV
ASCL1
PAH
NUP37
C12orf48
IGF1
PMCH
C12orf42

Figure 80. Analysis of genic environment of human CNE-enhancers in teleost fish orthologous genomic intervals and observing their expression patterns helps in identifying their target genes (hs1257/hs1122).

Analysis of CNE-enhancers in humans and its orthologous genomic intervals teleost fish lineage helps in associating CNE to their target genes. **(A)** Both synteny analysis and endogenous expression pattern clearly associate the CNE to human *ESRRG* gene. **(B*)*** Both synteny analysis and endogenous expression pattern clearly associate CNE-enhancer within an intron of human *PAH* gene with neighboring *IGF1* gene.

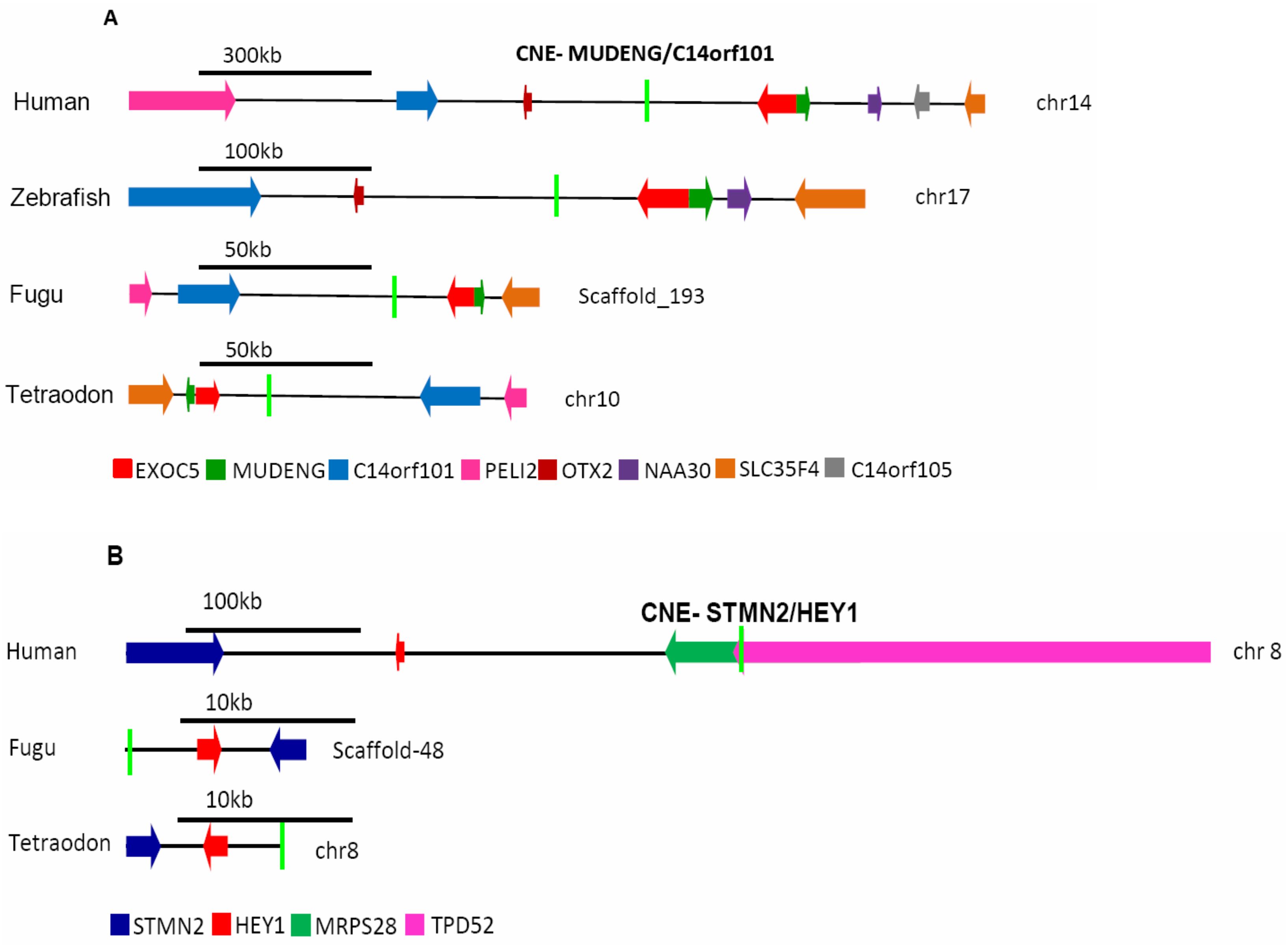
A
300kb
CNE- MUDENG/C14orf101
Human
chr14
100kb
Zebrafish
chr17
50kb
Fugu
Scaffold_193
50kb
Tetraodon
chr10
EXOC5 MUDENG C14orf101 PELI2 OTX2 NAA30 SLC35F4 C14orf105
B
100kb
CNE- STMN2/HEY1
Human
chr 8
10kb
Fugu
Scaffold-48
10kb
Tetraodon
chr8
STMN2 HEY1 MRPS28 TPD52

Figure 81. Analysis of genic environment of human CNE-enhancers in teleost fish orthologous genomic intervals and observing their expression patterns helps in identifying their target genes (hs1791/hs1305).

Analysis of CNE-enhancers in humans and its orthologous genomic intervals teleost fish lineage helps in associating CNE to their target genes. **(A)** CNE-enhancer is positioned within an intergenic space between human *OTX2* and *EXOC5* on chromosome 14 suggest its association with either of these genes. however the comparative syntenic analysis in fish lineage and expression studies of neighboring conserved genes infers that this CNE might be regulating the expression of downstream *MUDENG* and upstream *c14orf101* gene. **(B)** CNE-enhancer within an intron of human *MRPS28* gene is not associated with the promoter of the same gene, rather comparative syntenic analysis in fish lineage and expression studies of neighboring conserved genes infers that this CNE is acting the at a distance of ~150.8 kb downstream on *HEY1* and *STMN2* genes .

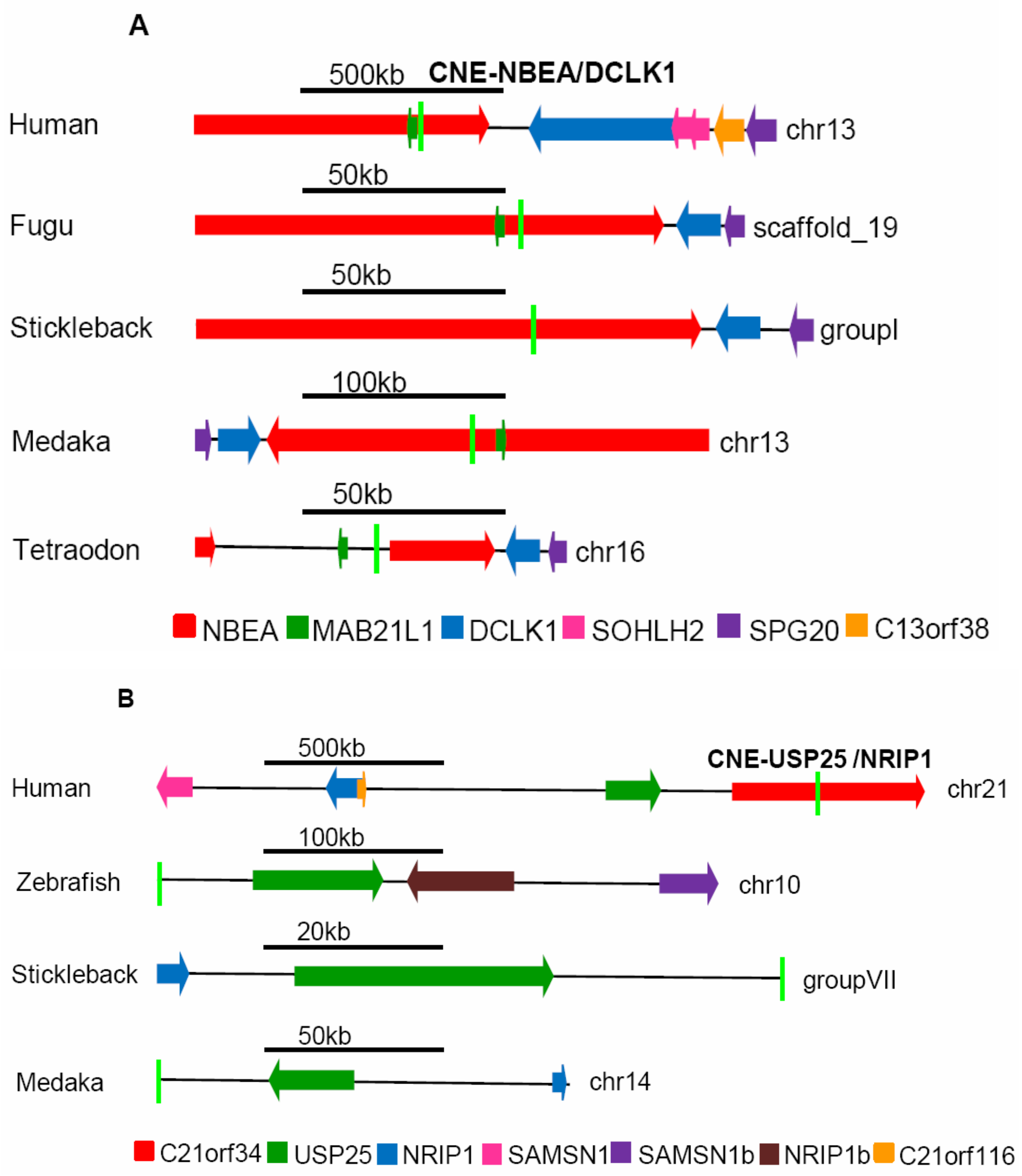
A
500kb
CNE-NBEA/DCLK1
Human
chr13
50kb
Fugu
scaffold_19
50kb
Stickleback
groupI
100kb
Medaka
chr13
50kb
Tetraodon
chr16
NBEA
MAB21L1
DCLK1
SOHLH2
SPG20
C13orf38
B
500kb
CNE-USP25 /NRIP1
Human
chr21
100kb
Zebrafish
chr10
20kb
Stickleback
groupVII
50kb
Medaka
chr14
C21orf34
USP25
NRIP1
SAMSN1
SAMSN1b
NRIP1b
C21orf116

Figure 82. Analysis of genic environment of human CNE-enhancers in teleost fish orthologous genomic intervals and observing their expression patterns helps in identifying their target genes (hs1333/hs1304).

Analysis of CNE-enhancers in humans and its orthologous genomic intervals teleost fish lineage helps in associating CNE to their target genes. **(A)** An CNE-enhancer within the intronic region of *NBEA* is conserved in its orthologous intervals in fish lineages, along with downstream *DCLK1* and other bystander genes. However the endogenous expression pattern of *NBEA* and *DCLK1* matches with that of reporter gene expression, clearly inferring the association of enhancer with *NBEA* and downstream *DCLK1* genes. **(B)** The conserved physical location of CNE-enhancer within intronic region of *C10orf34* gene suggest its association with *C10orf34* gene, however the expression studies of neighboring conserved genes infers that this CNE might be regulating the expression of *USP25* and *NRIP1* genes rather than *C10orf34*.

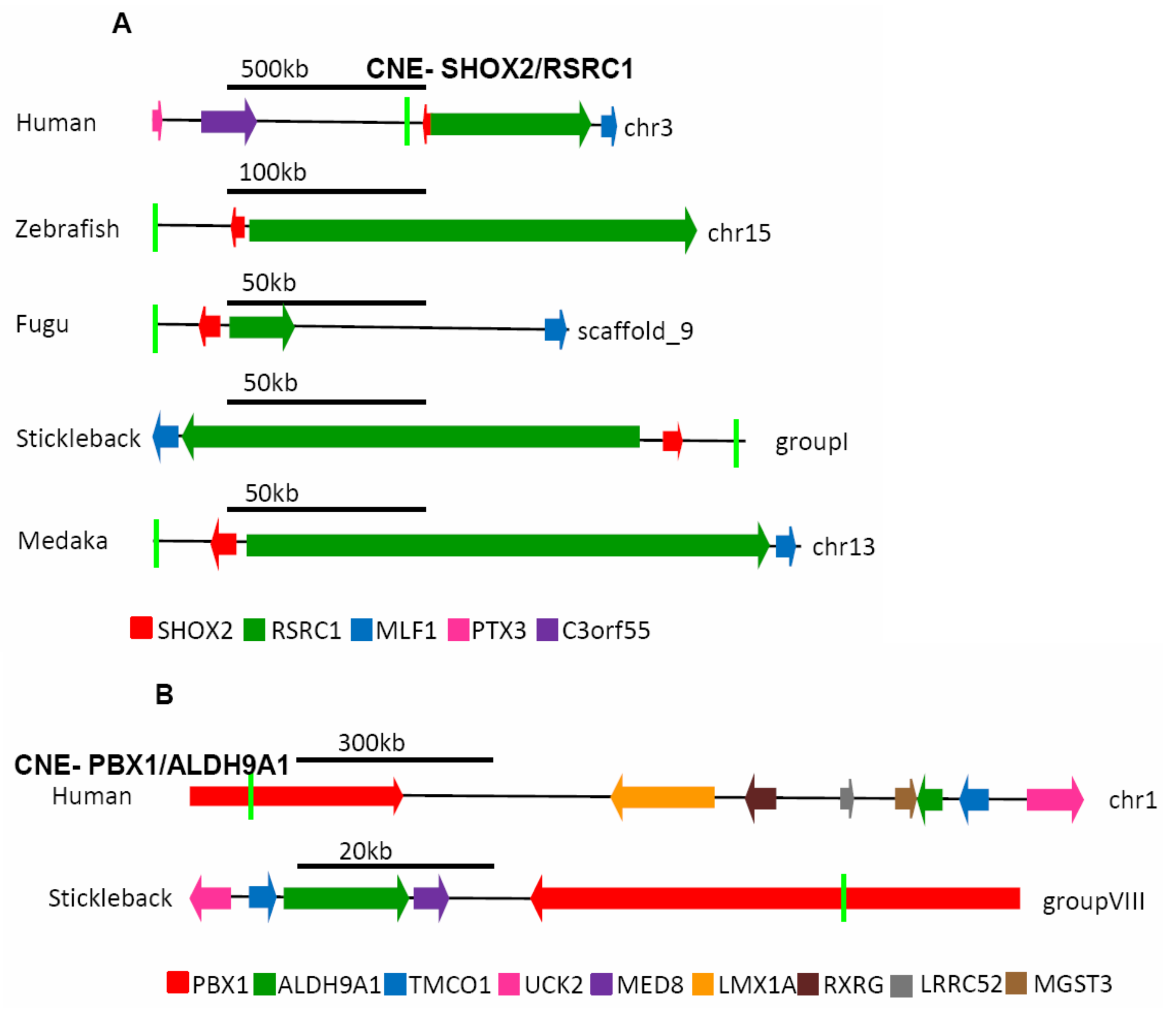
A
CNE- SHOX2/RSRC1
500kb
Human
chr3
100kb
Zebrafish
chr15
50kb
Fugu
scaffold_9
50kb
Stickleback
groupI
50kb
Medaka
chr13
SHOX2
RSRC1
MLF1
PTX3
C3orf55
B
CNE- PBX1/ALDH9A1
300kb
Human
chr1
20kb
Stickleback
groupVIII
PBX1
ALDH9A1
TMCO1
UCK2
MED8
LMX1A
RXRG
LRRC52
MGST3

Figure 83. Analysis of genic environment of human CNE-enhancers in teleost fish orthologous genomic intervals and observing their expression patterns helps in identifying their target genes (hs1251/hs1235).

Analysis of CNE-enhancers in humans and its orthologous genomic intervals teleost fish lineage helps in associating CNE to their target genes. **(A)** CNE-enhancer is positioned within an intergenic space between human *C3orf55* and *SHOX2* on chromosome 3 .Comparative syntenic analysis in fish lineage and expression pattern studies suggest the association of this CNE with *SHOX2* or *RSRC1*. **(B)** The CNE-enhancer located within intronic region of *PBX1* in human and some fish lineages is conserved along *ALDH9A1* and other bystander genes . The comparative syntenic analysis of human locus with multiple fish lineages suggest its association with *PBX1* and *ALDH9A1*, moreover expression pattern studies also favors this association.

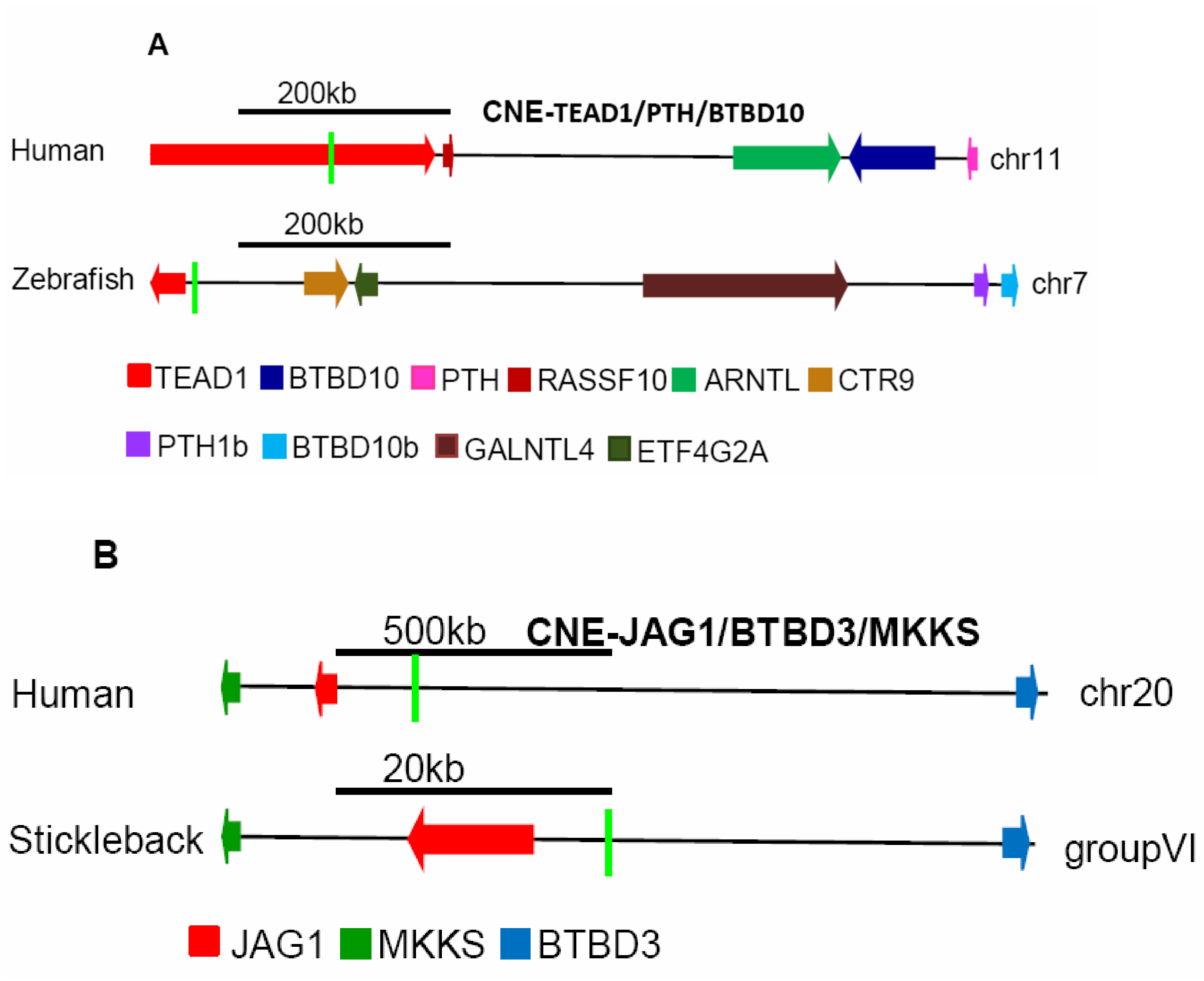

A
200kb
CNE-TEAD1/PTH/BTBD10
Human
chr11
200kb
Zebrafish
chr7
TEAD1 BTBD10 PTH RASSF10 ARNTL CTR9
PTH1b BTBD10b GALNTL4 ETF4G2A
B
500kb
CNE-JAG1/BTBD3/MKKS
Human
chr20
20kb
Stickleback
groupVI
JAG1 MKKS BTBD3

Figure 84. Analysis of genic environment of human CNE-enhancers in teleost fish orthologous genomic intervals and observing their expression patterns helps in identifying their target genes (hs1314/hs1278).

Analysis of CNE-enhancers in humans and its orthologous genomic intervals teleost fish lineage helps in associating CNE to their target genes. **(A)** The physical location of human CNE enhancer within the intronic region of *TEAD1* is conserved in its orthologous intervals in fish lineages, along with *PTH* and *BTBD10* genes. However the endogenous expression pattern is clearly inferring the association of enhancer with *TEAD1,PTH* and *BTBD10*. **(B)** An intergenic CNE-enhancer showed conserved positioning with respect to *JAG1* and *BTBD3* along with *MKKS* genes in human and teleost fish lineages. The expression pattern of these genes is highly redundant and in accordance with CNE-enhancer induced reporter expression, and suggests that this enhancer might be influencing the expression of all of these genes .

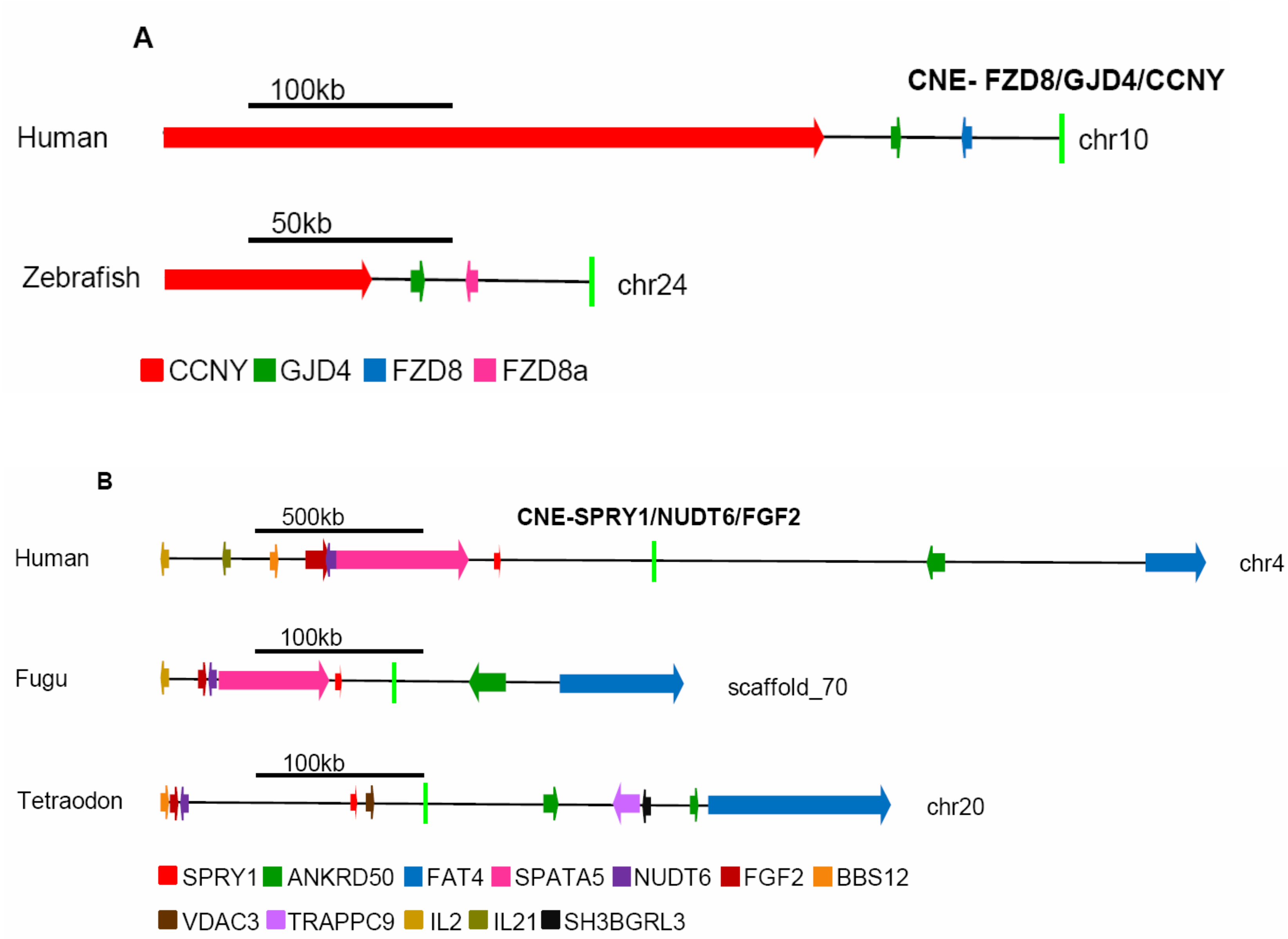
A
CNE- FZD8/GJD4/CCNY
100kb
Human
chr10
50kb
Zebrafish
chr24
CCNY
GJD4
FZD8
FZD8a
B
CNE-SPRY1/NUDT6/FGF2
500kb
Human
chr4
100kb
Fugu
scaffold_70
100kb
Tetraodon
chr20
SPRY1
ANKRD50
FAT4
SPATA5
NUDT6
FGF2
BBS12
VDAC3
TRAPPC9
IL2
IL21
SH3BGRL3

Figure 85. Analysis of genic environment of human CNE-enhancers in teleost fish orthologous genomic intervals and observing their expression patterns helps in identifying their target genes (hs1567/hs1525).

Analysis of CNE-enhancers in humans and its orthologous genomic intervals teleost fish lineage helps in associating CNE to their target genes. **(A)** Analyzing the expression pattern and conservation in fish lineage shows that CNE-enhancer is regulating the expression of *FZD8,GJD4,CCNY*. **(B)** Analyzing the expression pattern and conservation in fish lineage shows that human CNE-enhancer positioned in the intergenic space between human *SPRY1* and *ANKRD50* gene is regulating the expression of flanking *SPRY1* and downstream *NUDT6* and *FGF2* genes.

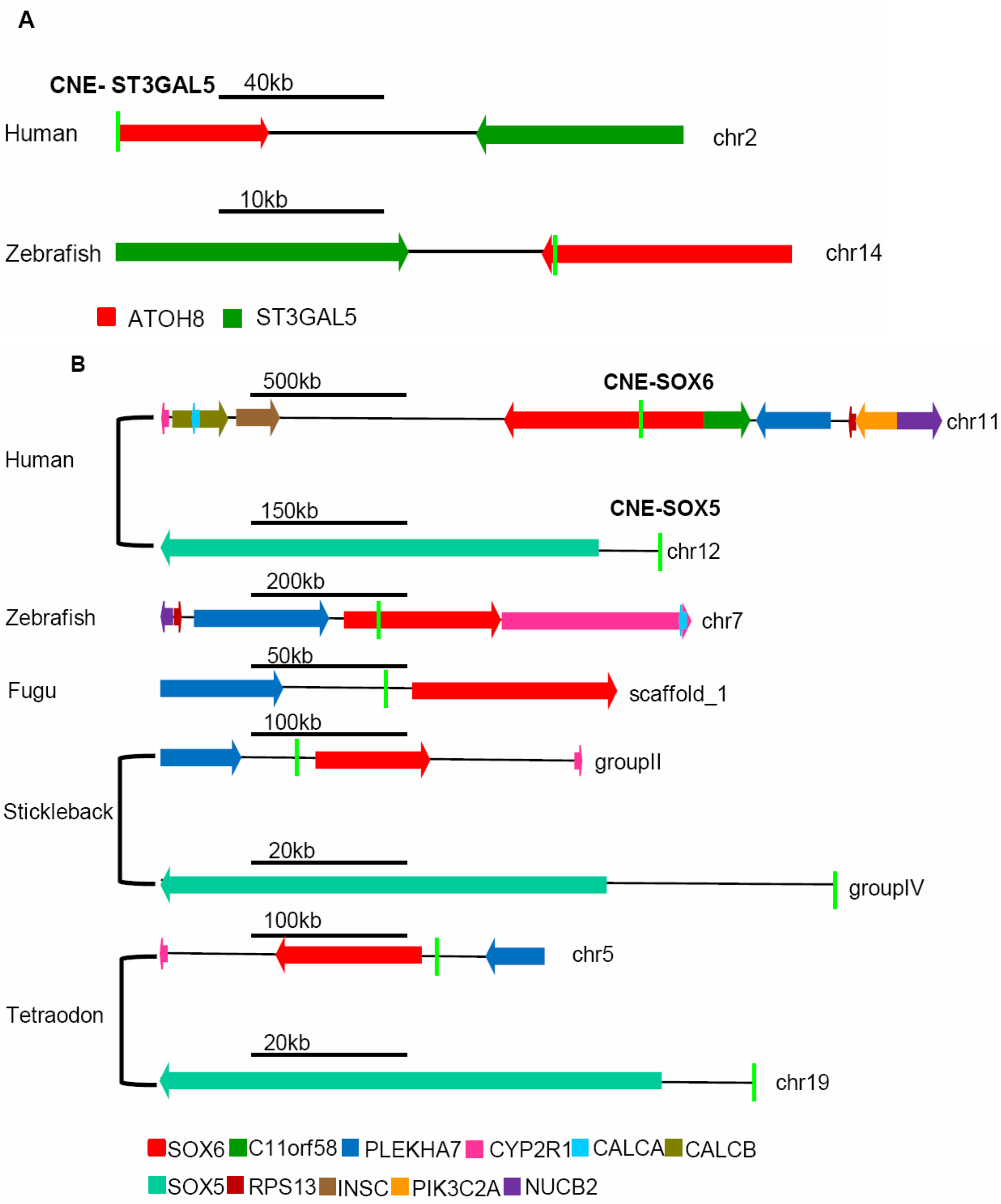
A
CNE- ST3GAL5
40kb
Human
chr2
10kb
Zebrafish
chr14
ATOH8
ST3GAL5
B
500kb
CNE-SOX6
Human
chr11
150kb
CNE-SOX5
chr12
200kb
Zebrafish
chr7
50kb
Fugu
scaffold_1
100kb
groupII
Stickleback
20kb
groupIV
100kb
chr5
Tetraodon
20kb
chr19
SOX6
C11orf58
PLEKHA7
CYP2R1
CALCA
CALCB
SOX5
RPS13
INSC
PIK3C2A
NUCB2

Figure 86. Analysis of genic environment of human CNE-enhancers in teleost fish orthologous genomic intervals and observing their expression patterns helps in identifying their target genes (hs1652/hs1301).

Analysis of CNE-enhancers in humans and its orthologous genomic intervals teleost fish lineage helps in associating CNE to their target genes. **(A)** The conserved physical location of CNE-enhancer within intronic region of *ATOH8* in fish and human lineages suggest its association with *ATOH8* gene, however the expression studies of neighboring conserved genes infers that this CNE might be regulating the expression of *ST3GAL5* rather than *ATOH8*. **(B)** Human CNE-enhancer and target gene duplicated before tetrapod-teleost divergence. Comparative syntenic analysis of human duplicated loci in multiple fish lineages clearly suggest that these duplicated human enhancers are associated with the regulation of human *SOX* paralogs(*SOX6,SOX5*) residing on human chromosome 11 and 12.

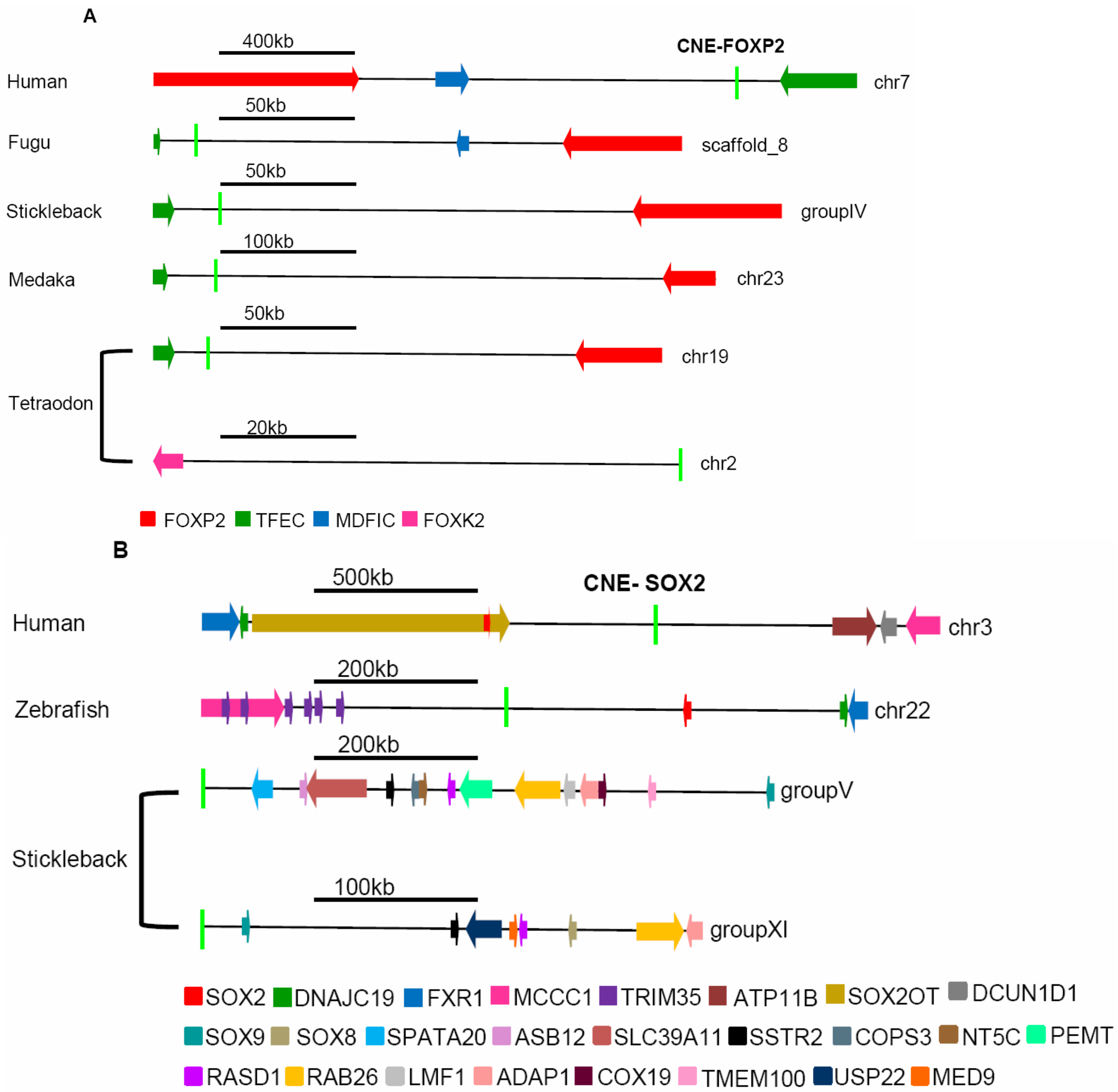
A
400kb
CNE-FOXP2
Human
chr7
50kb
Fugu
scaffold_8
50kb
Stickleback
groupIV
100kb
Medaka
chr23
50kb
chr19
Tetraodon
20kb
chr2
FOXP2 TFEC MDFIC FOXK2
B
500kb
CNE- SOX2
Human
chr3
200kb
Zebrafish
chr22
200kb
groupV
Stickleback
100kb
groupXI
SOX2 DNAJC19 FXR1 MCCC1 TRIM35 ATP11B SOX2OT DCUN1D1
SOX9 SOX8 SPATA20 ASB12 SLC39A11 SSTR2 COPS3 NT5C PEMT
RASD1 RAB26 LMF1 ADAP1 COX19 TMEM100 USP22 MED9

Figure 87. Analysis of genic environment of human CNE-enhancers in teleost fish orthologous genomic intervals and observing their expression patterns helps in identifying their target genes (hs1798/hs1332).

Analysis of CNE-enhancers in humans and its orthologous genomic intervals teleost fish lineage helps in associating CNE to their target genes. **(A)** Human cis-regulatory element positioned in the intergenic space between *MDFIC* and *TFEC*, suggesting that this enhancer might be associated with one of these genes but not with *FOXP2* that is present ~ 1.11 Mb upstream of *MDFIC*. However the careful analysis of genic context of orthologous regions of this element in teleost fish and its duplicated paralogous copy on Tetraodon chromosome 2 clearly indicates that the only gene in the human locus that preserved its association with this CNE-enhancer in all teleost fish analyzed (even in duplicated loci of tetraodon) is *FOXP2*.Therefore this human enhancer is unambiguously associated with the regulation of human *FOXP2* gene. **(B)** CNE-enhancer within an intergenic space between *SOX2OT* and *ATP11B* genes, suggesting that this enhancer might be associated with one of these genes but careful analysis of genic context of orthologous regions of this element in teleost fish and its duplicated paralogous (*SOX9,SOX8*) in Stickleback clearly indicates that the only gene in the human locus that preserved its association with this CNE-enhancer in all teleost fish analyzed (even in duplicated loci of Stickleback) is *SOX2* within intron of human *SOX2OT* gene. Therefore this human enhancer is unambiguously associated with the regulation of human *SOX2* gene.

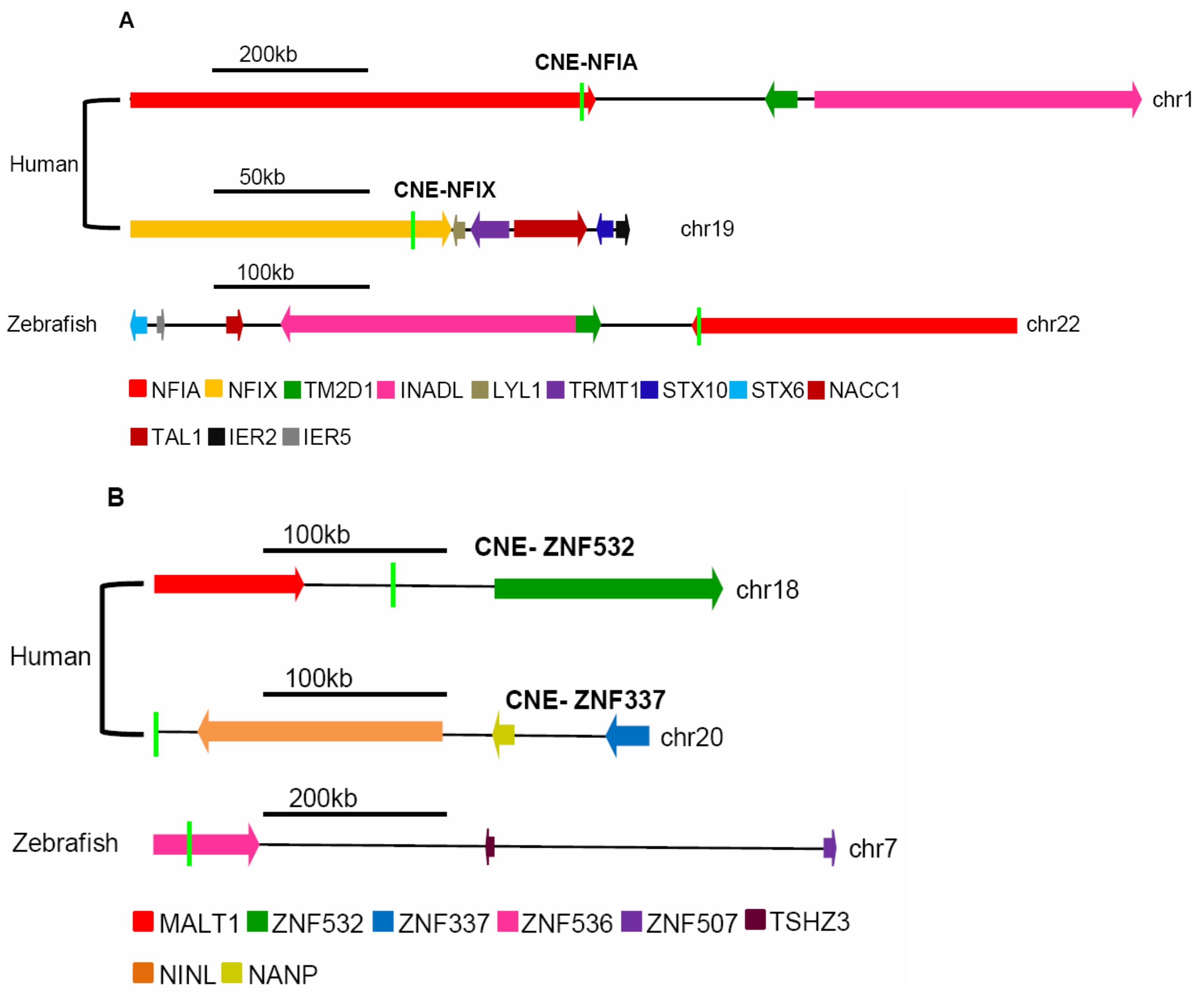
A
200kb
CNE-NFIA
chr1
Human
50kb
CNE-NFIX
chr19
100kb
Zebrafish
chr22
NFIA NFIX TM2D1 INADL LYL1 TRMT1 STX10 STX6 NACC1
TAL1 IER2 IER5
B
100kb
CNE- ZNF532
chr18
Human
100kb
CNE- ZNF337
chr20
200kb
Zebrafish
chr7
MALT1 ZNF532 ZNF337 ZNF536 ZNF507 TSHZ3
NINL NANP

Figure 88. Analysis of genic environment of human CNE-enhancers in teleost fish orthologous genomic intervals and observing their expression patterns helps in identifying their target genes (hs1309/hs1397).

Analysis of CNE-enhancers in humans and its orthologous genomic intervals teleost fish lineage helps in associating CNE to their target genes. **(A)** Human CNE-enhancer duplicated in human lineage is positioned within the intronic interval of *NFIA* gene on chromosome 1. Careful analysis of genic context of orthologous regions of this element in teleost fish and its duplicated paralogous copy on human chromosome suggests the association of this enhancer with *NFIA* gene. **(B)** Duplicated human CNE enhancer within an intergenic space between *MALT1* and *ZNF532* is associated with either of these genes where as careful analysis of genic context of orthologous regions of this element in teleost fish reveal that it is associated with *ZNF532*.

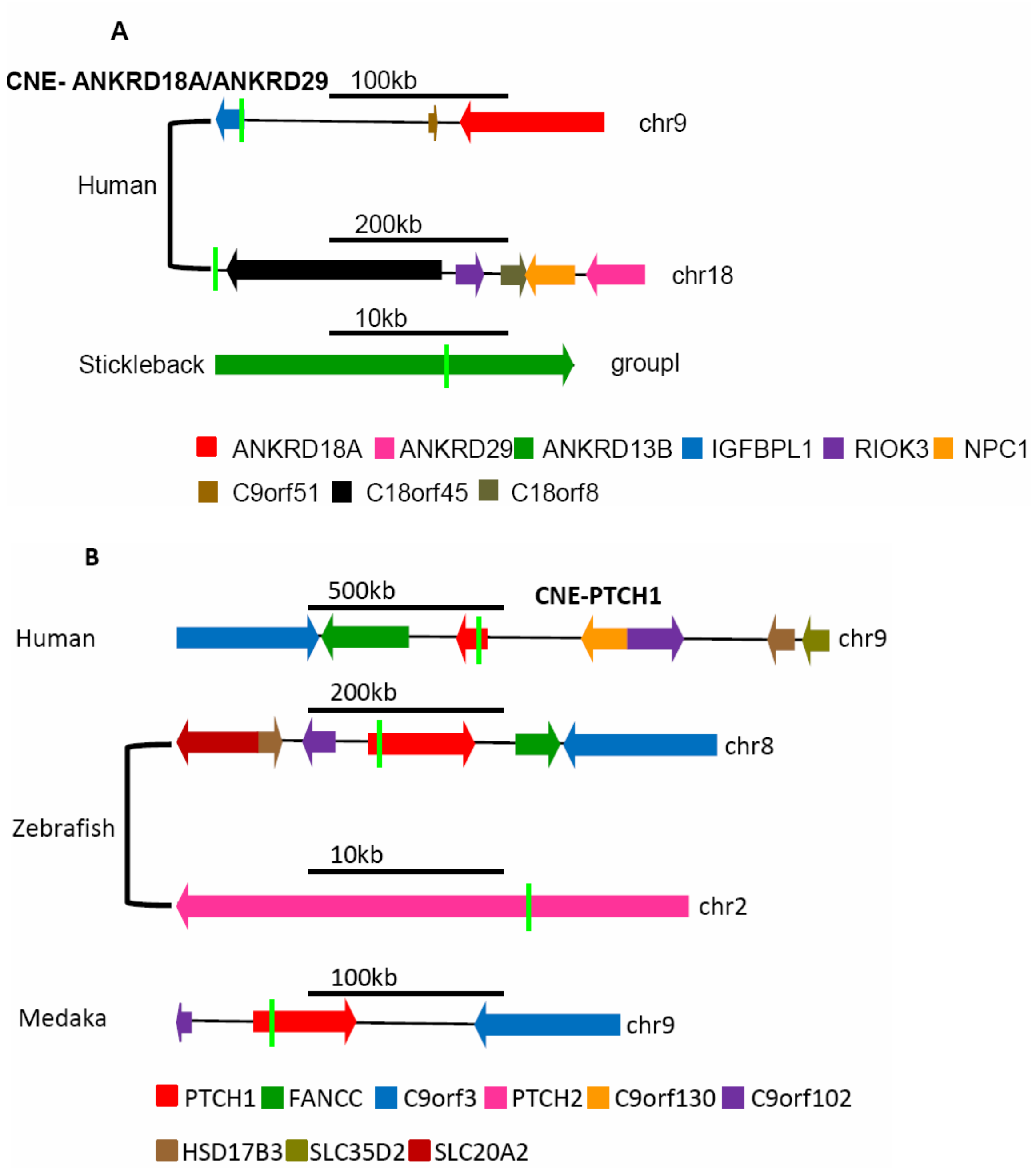
A
CNE- ANKRD18A/ANKRD29
100kb
chr9
Human
200kb
chr18
10kb
Stickleback
groupI
ANKRD18A
ANKRD29
ANKRD13B
IGFBPL1
RIOK3
NPC1
C9orf51
C18orf45
C18orf8
B
500kb
CNE-PTCH1
Human
chr9
200kb
chr8
Zebrafish
10kb
chr2
100kb
Medaka
chr9
PTCH1
FANCC
C9orf3
PTCH2
C9orf130
C9orf102
HSD17B3
SLC35D2
SLC20A2

Figure 89. Analysis of genic environment of human CNE-enhancers in teleost fish orthologous genomic intervals and observing their expression patterns helps in identifying their target genes (hs1390/hs1258).

Analysis of CNE-enhancers in humans and its orthologous genomic intervals teleost fish lineage helps in associating CNE to their target genes. **(A)** CNE-enhancer within an intron of human *IGFBPL1* gene is duplicated in Human .Comparative syntenic analysis of this locus revealed that genomic interval encompassing this conserved enhancer in all lineages links this CNE with the regulation of neighboring *ANKRD18A & ANKRD29* gene. Although its expression pattern is not clearly defined but syntenic analysis provide a strong evidence in favor of its association with *ANKRD18A & ANKRD29* gene. **(B)** Human CNE-enhancer is positioned within the intronic interval of *PTCH1* gene. Comparative analysis of this locus in teleost fish revealed that genomic interval encompassing this conserved enhancer is duplicated in zebrafish (*PTCH1,PTCH2*). The conserved linkage of CNE with *PTCH1* in all lineages and its endogenous expression unmistakably links this CNE with the regulation of *PTCH1* gene.

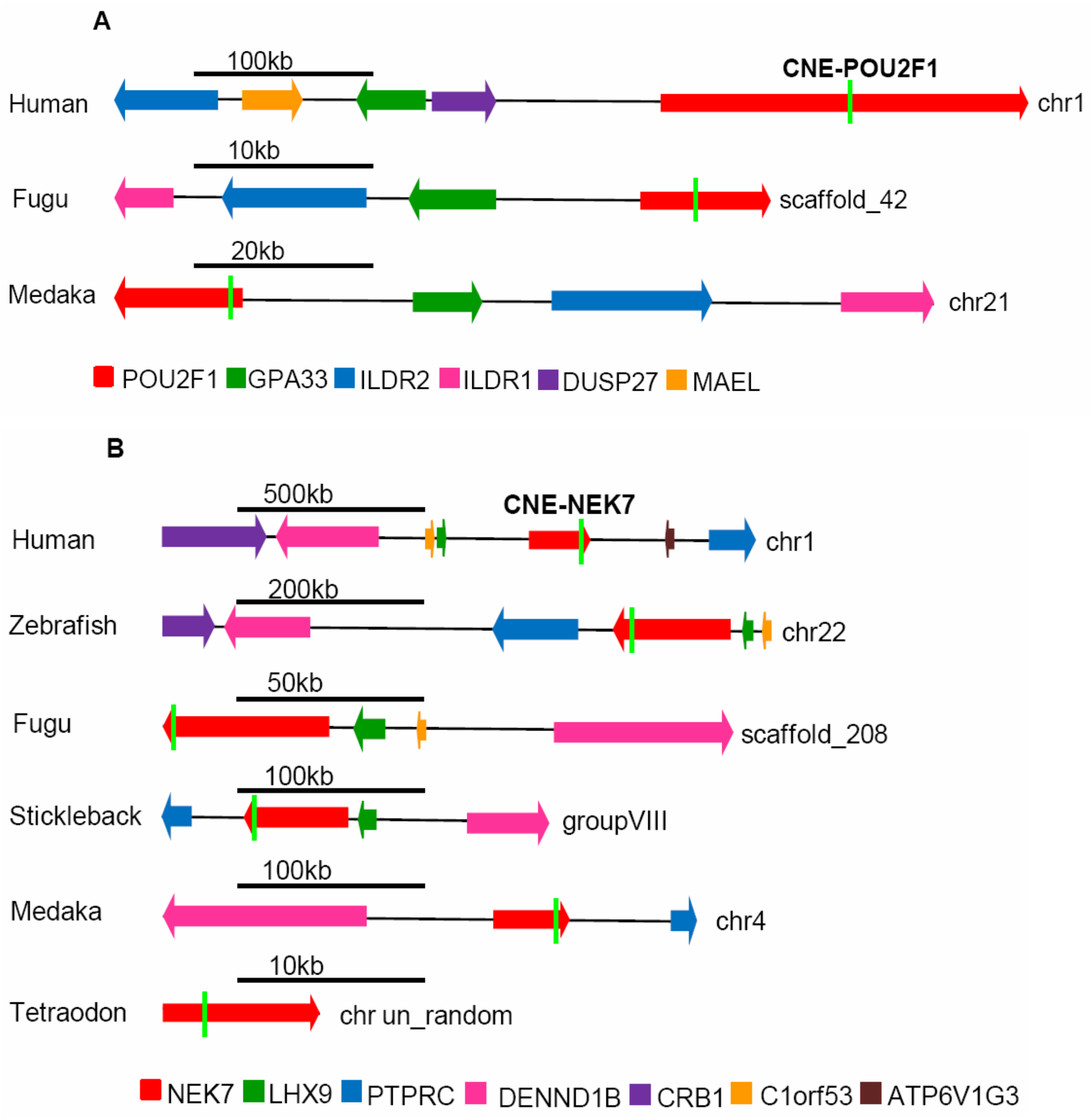
A
100kb
CNE-POU2F1
Human
chr1
10kb
Fugu
scaffold_42
20kb
Medaka
chr21
POU2F1
GPA33
ILDR2
ILDR1
DUSP27
MAEL
B
500kb
CNE-NEK7
Human
chr1
200kb
Zebrafish
chr22
50kb
Fugu
scaffold_208
100kb
Stickleback
groupVIII
100kb
Medaka
chr4
10kb
Tetraodon
chr un_random
NEK7
LHX9
PTPRC
DENND1B
CRB1
C1orf53
ATP6V1G3

Figure 90. Analysis of genic environment of human CNE-enhancers in teleost fish orthologous genomic intervals and observing their expression patterns helps in identifying their target genes (hs1331/hs1322).

Analysis of CNE-enhancers in humans and its orthologous genomic intervals teleost fish lineage helps in associating CNE to their target genes. **(A)** CNE-enhancer within an intron of human *POU2F1* gene is associated with the promoter of the same gene. Other human-fish conserved genes within this locus appeared to be bystanders as their expression pattern is unrelated to the activity of this cis-acting region. **(B)** CNE-enhancer within an intron of human *NEK7* gene is associated with the promoter of the same gene. Other human-fish conserved genes within this locus appeared to be bystanders as their expression pattern is unrelated to the activity of this cis-acting region.

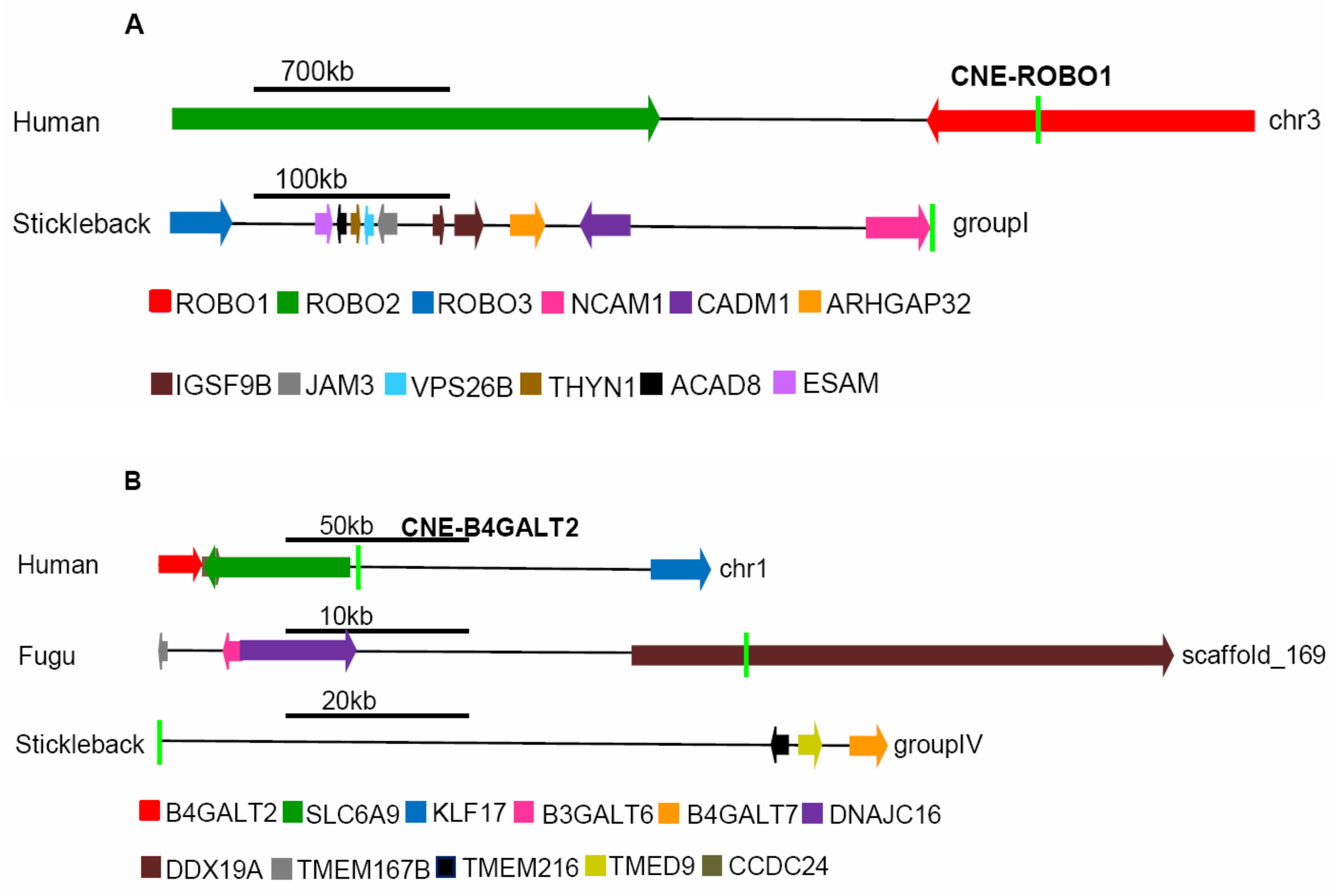
A
700kb
CNE-ROBO1
Human
chr3
100kb
Stickleback
groupI
ROBO1 ROBO2 ROBO3 NCAM1 CADM1 ARHGAP32
IGSF9B JAM3 VPS26B THYN1 ACAD8 ESAM
B
50kb
CNE-B4GALT2
Human
chr1
10kb
Fugu
scaffold_169
20kb
Stickleback
groupIV
B4GALT2 SLC6A9 KLF17 B3GALT6 B4GALT7 DNAJC16
DDX19A TMEM167B TMEM216 TMED9 CCDC24

Figure 91. Analysis of genic environment of human CNE-enhancers in teleost fish orthologous genomic intervals and observing their expression patterns helps in identifying their target genes (hs1475/hs1857).

Analysis of CNE-enhancers in humans and its orthologous genomic intervals teleost fish lineage helps in associating CNE to their target genes. **(A)** CNE-enhancer within an intron of human *ROBO1* gene is associated with the promoter of same gene. **(B)** CNE-enhancer within an intergenic space between human *SLC6A9* and *KLF17*gene is associated with neighboring *B4GALT2* gene.
