# Supplementary material for: Comparative genomics using teleost fish helps to systematically identify target gene bodies of functionally defined human enhancers
Source: BMC Genomics. 2013 Feb 23;14:122. doi: 10.1186/1471-2164-14-122 (PMC3599049; doi:10.1186/1471-2164-14-122)
Supplement: Additional file 4: Table S3 — List of CNE-enhancers which reside >1.5 Mb apart from their target gene. [file 1471-2164-14-122-S4.doc]

| **Supplementary Table S3: List of CNE-enhancers which reside >1.5 Mb apart from their target gene.** | | | |
| --- | --- | --- | --- |
|  | **CNE-enhancers** | **Enhancer ID** | **Radius** |
| 1 | CNE_FANCL-BCL11A | hs1067 | 1.9Mb |
| 2 | CNE_MAF-DNCL2B(WWOX) | hs4 | 1.75Mb |
| 3 | CNE_WWOX (MAF) | hs12 | 2.04Mb |
| 4 | CNE_ARRDC3-NR2F1 | hs191 | 1.9mb |
| 5 | CNE_TBC1D5-SATB1 | hs250 | 1.8mb |
| 6 | CNE_ KIAA1900 | hs676 | 1.7mb |
| 7 | CNE_GTPBP9 - FLJ46347 | hs860 | 1.7mb |
| 8 | CNE_PXMP3-PKIA(ZFHX4,HNF4G) | hs742 | 1.7Mb |
| 9 | CNE_MGMT(intragenic)(PPP2R2D) | hs656 | 2.16Mb |
