# Supplementary material for: Comparative genomics using teleost fish helps to systematically identify target gene bodies of functionally defined human enhancers
Source: BMC Genomics. 2013 Feb 23;14:122. doi: 10.1186/1471-2164-14-122 (PMC3599049; doi:10.1186/1471-2164-14-122)
Supplement: Additional file 7: Table S6 — R = [rij2] A Correlation Matrix with Lower Diagonal for Training and Upper Diagonal for the Control data set. [file 1471-2164-14-122-S7.doc]

| **Table S6**  $R=[r_{ij}^{2}]$**: A Correlation Matrix with Lower Diagonal for Training and Upper Diagonal for the Control data set.** | | | | | | | | | | | | | | |
| --- | --- | --- | --- | --- | --- | --- | --- | --- | --- | --- | --- | --- | --- | --- |
|  | **Tal1β** | **NFkapaB** | **nMYC** | **ARNT** | **USF** | **c-REL** | **MEF** | **FREAC** | **AML-1** | **HFH** | **c-FOS** | **HNFβ** | **SOX17** | **SOX5** |
| **TaI1β** |  | *0.00* | *0.00* | *0.01* | *0.00* | *0.00* | *0.01* | *0.00* | *0.01* | *0.00* | *0.07* | *0.02* | *0.00* | *0.01* |
| **NFkapaB** | 0.04 |  | *0.01* | *0.01* | *0.01* | ***0.12*** | *0.00* | *0.00* | *0.00* | *0.00* | *0.06* | *0.00* | *0.00* | *0.01* |
| **n-MYC** | 0.01 | 0.00 |  | ***0.71*** | ***1.00*** | *0.00* | *0.00* | *0.00* | *0.07* | *0.01* | *0.00* | *0.00* | *0.01* | *0.03* |
| **ARNT** | 0.03 | 0.00 | **0.69** |  | ***0.71*** | *0.01* | *0.01* | *0.00* | *0.04* | *0.00* | *0.00* | *0.00* | *0.00* | *0.02* |
| **USF** | 0.02 | 0.02 | **0.68** | **0.78** |  | *0.00* | *0.00* | *0.00* | *0.07* | *0.01* | *0.00* | *0.00* | *0.01* | *0.03* |
| **c-REL** | 0.02 | **0.31** | 0.00 | 0.00 | 0.00 |  | *0.02* | *0.00* | *0.01* | *0.01* | *0.01* | *0.00* | *0.01* | *0.00* |
| **MEF** | 0.01 | 0.01 | 0.00 | 0.00 | 0.00 | 0.02 |  | *0.01* | *0.00* | *0.00* | *0.00* | *0.03* | *0.00* | *0.01* |
| **FREAC** | 0.07 | 0.00 | 0.00 | 0.00 | 0.00 | 0.00 | 0.04 |  | *0.02* | *0.01* | *0.00* | *0.00* | *0.00* | *0.01* |
| **AML-1** | 0.01 | 0.00 | 0.00 | 0.00 | 0.00 | 0.00 | 0.03 | 0.08 |  | *0.00* | *0.00* | *0.00* | *0.04* | *0.00* |
| **HFH** | 0.03 | 0.00 | 0.00 | 0.00 | 0.00 | 0.03 | 0.07 | **0.22** | ***0.14*** |  | *0.00* | ***0.30*** | *0.05* | *0.02* |
| **c-FOS** | 0.05 | 0.00 | 0.00 | 0.01 | 0.01 | 0.03 | 0.00 | 0.08 | 0.09 | **0.19** |  | *0.00* | *0.01* | *0.00* |
| **HNF3β** | 0.03 | 0.00 | 0.01 | 0.00 | 0.00 | 0.02 | 0.08 | **0.28** | ***0.12*** | **0.54** | ***0.14*** |  | *0.00* | *0.00* |
| **SOX17** | 0.04 | 0.00 | 0.00 | 0.00 | 0.00 | 0.00 | 0.05 | ***0.16*** | 0.09 | **0.21** | ***0.15*** | ***0.15*** |  | ***0.13*** |
| **SOX5** | 0.03 | 0.00 | 0.00 | 0.01 | 0.00 | 0.05 | **0.11** | ***0.17*** | **0.20** | **0.46** | **0.22** | **0.36** | **0.36** |  |

The yellow highlighted and bold cells in the lower diagonal matrix represent high correlation in the pairs of TFs. The Cells highlighted Turquoise and bold but Italics are with less than 20 % covariance relationship. But in the context more than 10% may be taken as a degree of relationship compared to zero correlation. The three groups in the training data set are

1. Group-1: nMYC, ARNT, USF with $r^{2}$ as high as 70%
2. Group-2: c-REL, NF-Kappaβ with $r^{2}$ 31%
3. Group-3: HFH, HNF3-β, SOX5, SOX17, FREAC, c-FOS with $12\%\leq r^{2}\leq56\%$

The three groups defined are strongly interactive internally, with no correlation among, furnishing the groups as non overlapping and distinctively exclusive in contrast to the upper diagonal matrix (the control data set) which is deficit of any similar co varying group of TFs. Only Group-1 is seen in the control data set (cells in bold and green) with highest internal correlation within and no correlation with the other TFs.
